# Supplementary figures and images for: Brown remodeling of white adipose tissue protects against abdominal aortic aneurysm via batokine FSTL1 (part 1 of 2)
Source: EMBO Mol Med. 2025 Oct 9;17(11):3080–109. doi: 10.1038/s44321-025-00318-z (PMC12603302; doi:10.1038/s44321-025-00318-z)

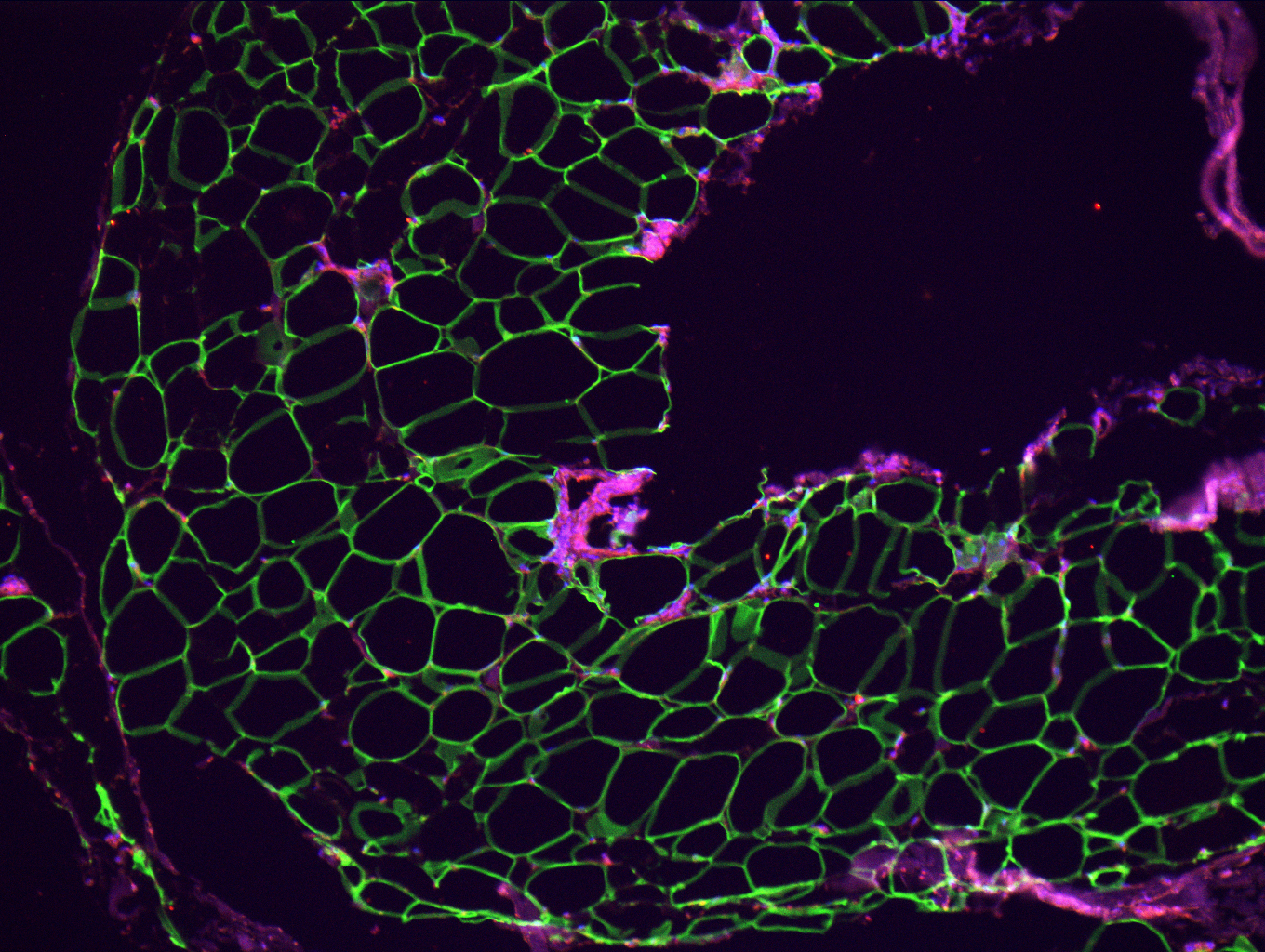

Supplement: Supplementary file 2 — Source data Fig. 1 [file 44321_2025_318_MOESM2_ESM.zip › Figure 1/Figure 1A/AAA Merge.tif]

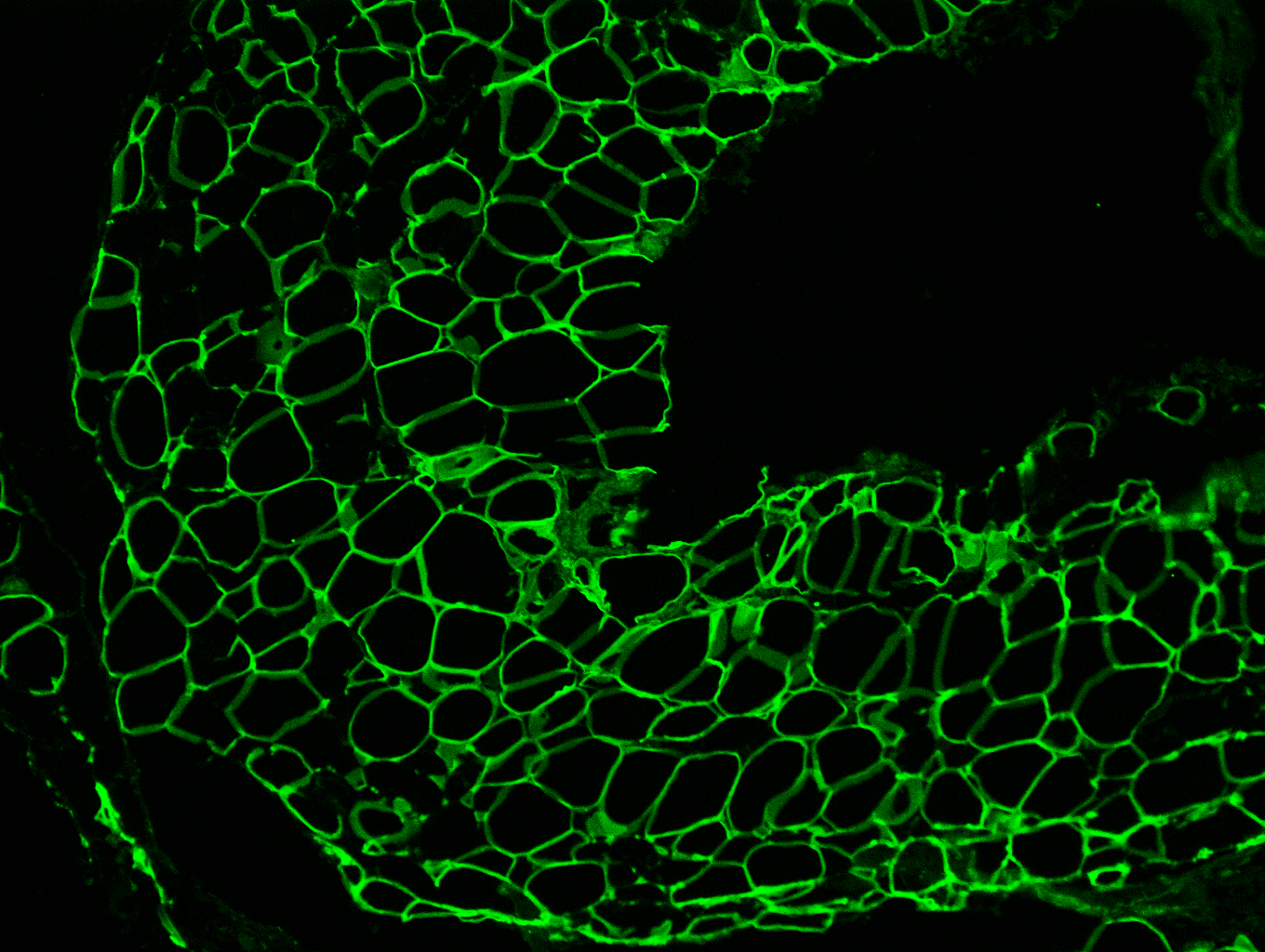

Supplement: Supplementary file 2 — Source data Fig. 1 [file 44321_2025_318_MOESM2_ESM.zip › Figure 1/Figure 1A/AAA Perilipin 1.tif]

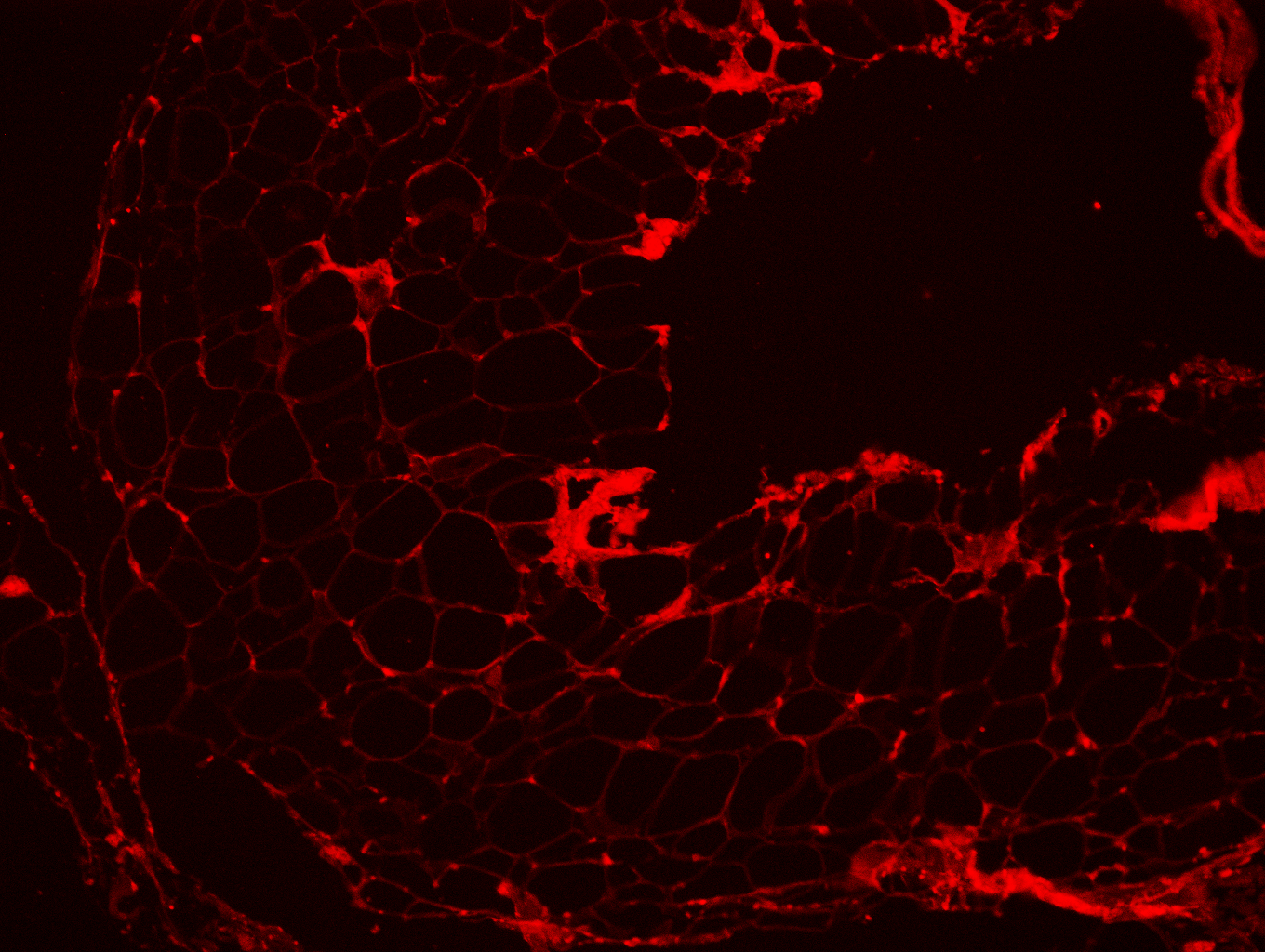

Supplement: Supplementary file 2 — Source data Fig. 1 [file 44321_2025_318_MOESM2_ESM.zip › Figure 1/Figure 1A/AAA UCP-1.tif]

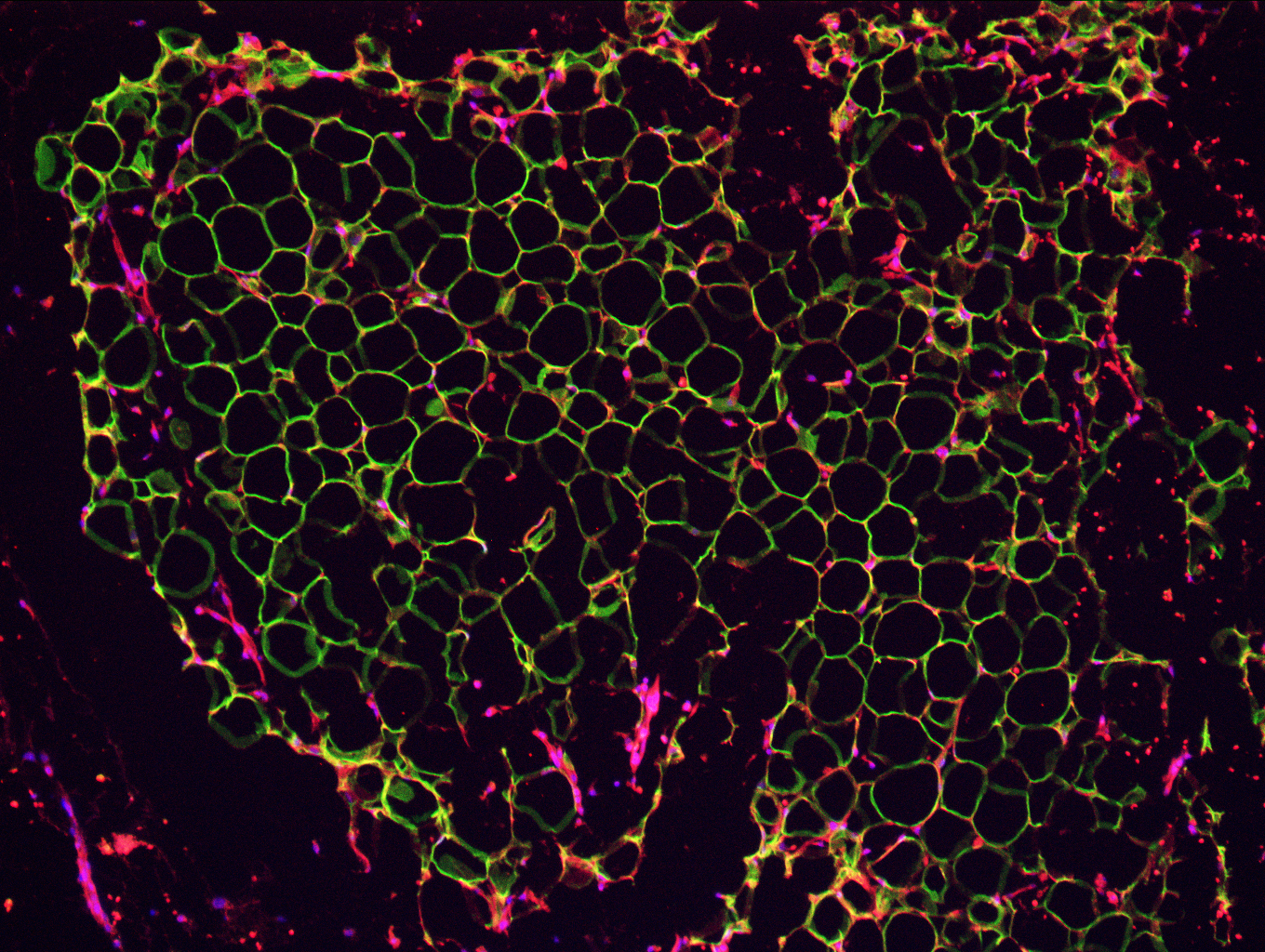

Supplement: Supplementary file 2 — Source data Fig. 1 [file 44321_2025_318_MOESM2_ESM.zip › Figure 1/Figure 1A/non AAA Merge.tif]

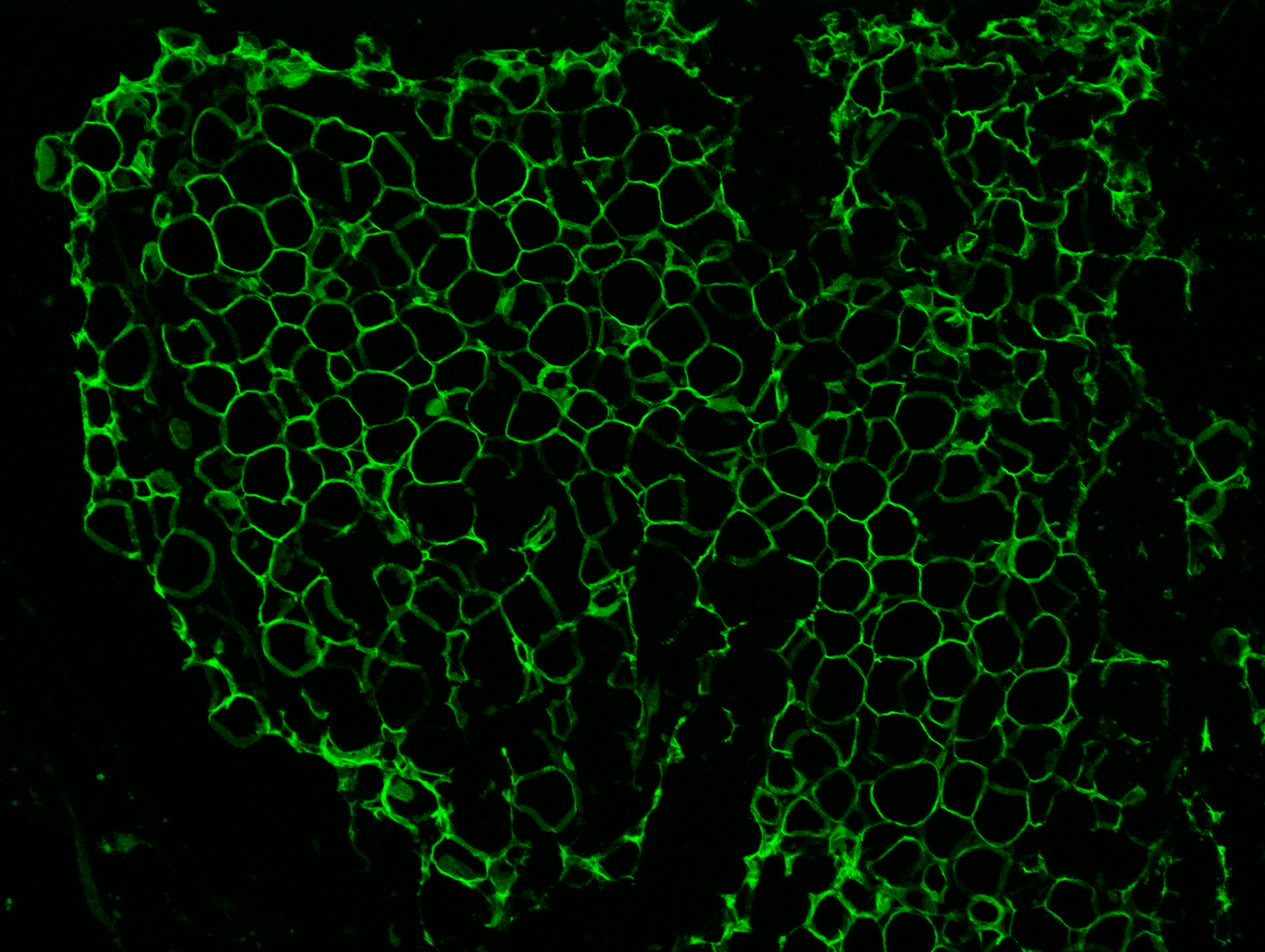

Supplement: Supplementary file 2 — Source data Fig. 1 [file 44321_2025_318_MOESM2_ESM.zip › Figure 1/Figure 1A/non AAA Perilipin 1.tif]

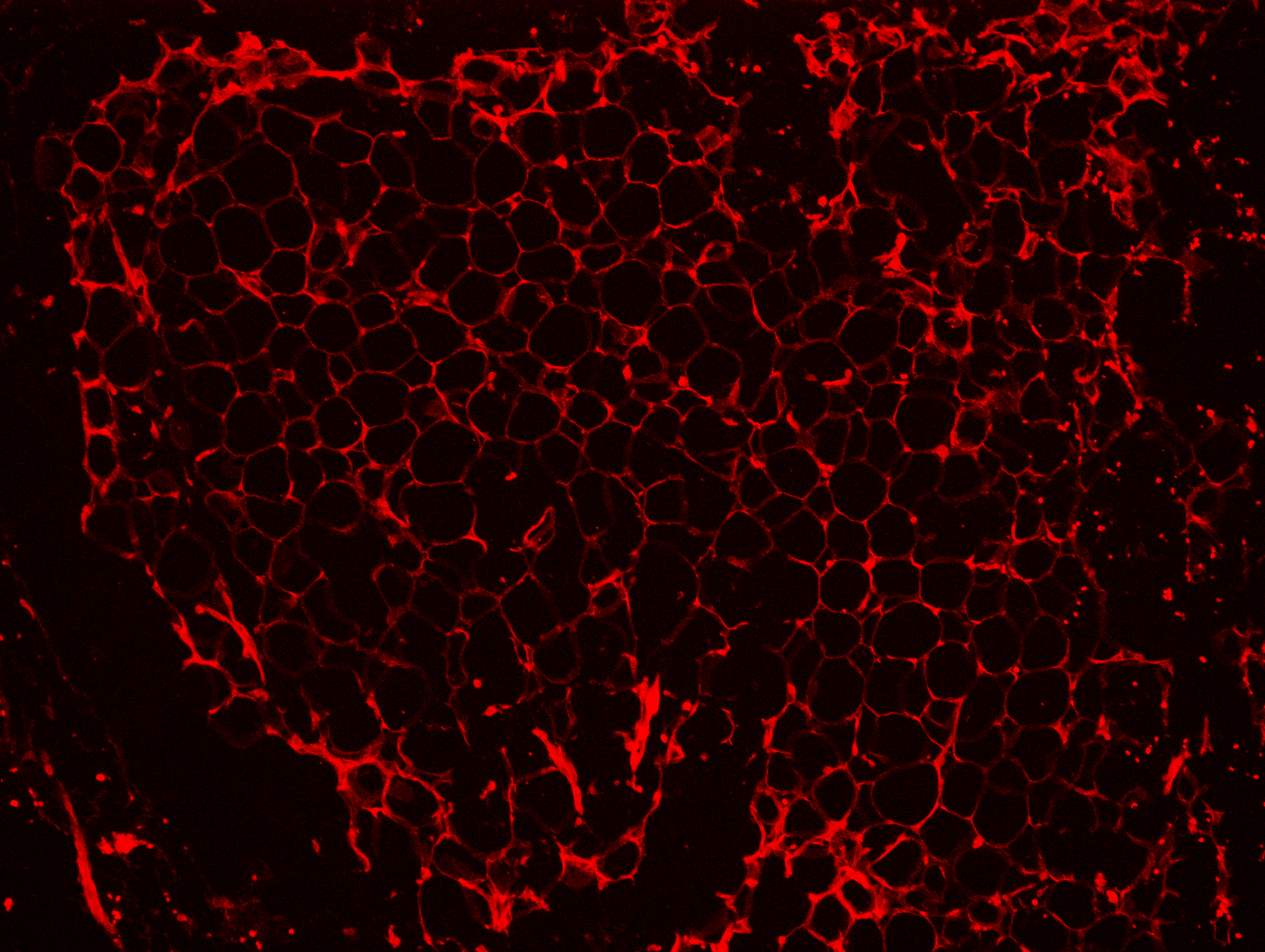

Supplement: Supplementary file 2 — Source data Fig. 1 [file 44321_2025_318_MOESM2_ESM.zip › Figure 1/Figure 1A/non AAA UCP-1.tif]

## Slide 1
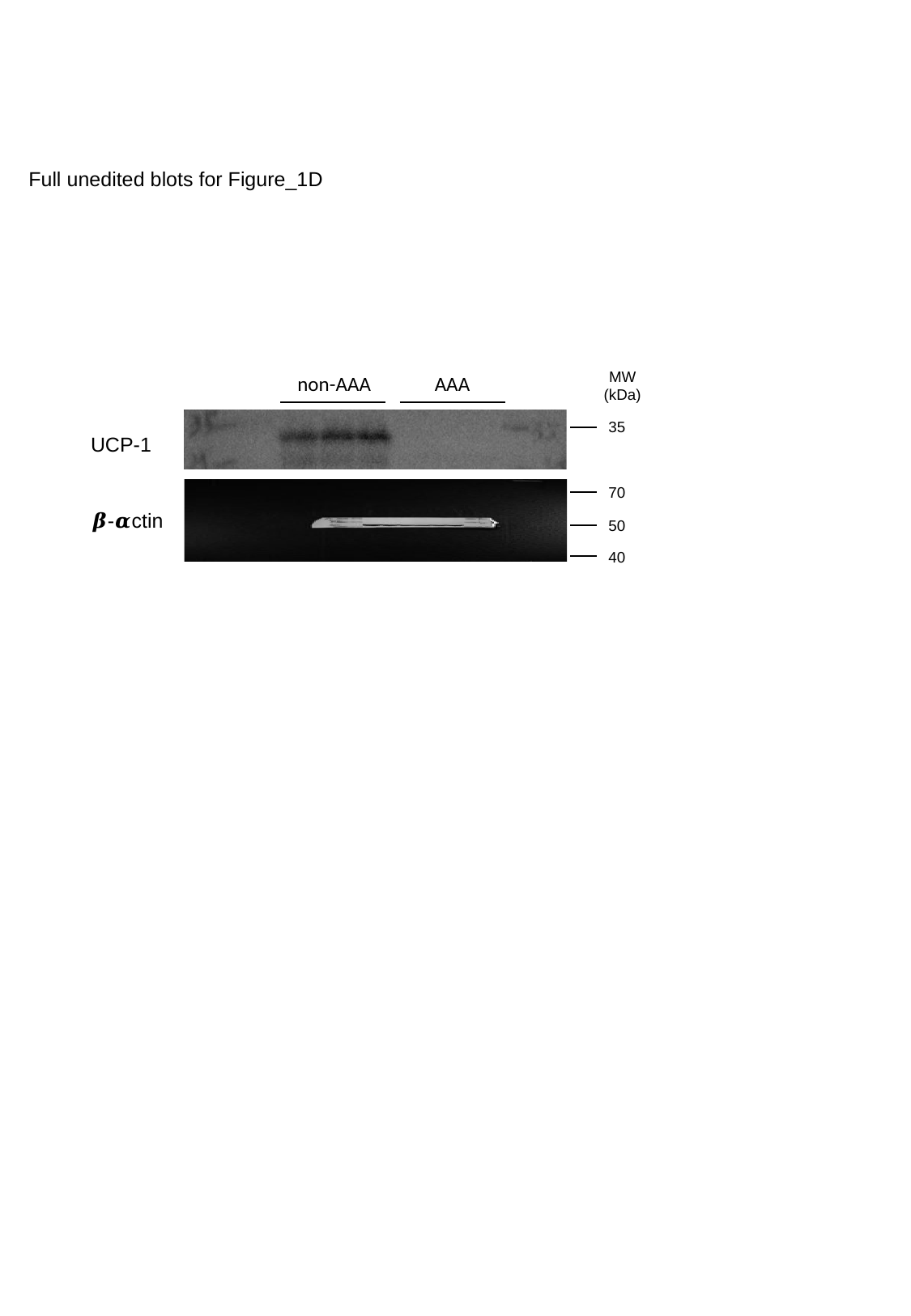

Full unedited blots for Figure_1D
MW
(kDa)
non-AAA
AAA
35
UCP-1
70
𝜷-𝜶ctin
50
40

Supplement: Supplementary file 2 — Source data Fig. 1 [file 44321_2025_318_MOESM2_ESM.zip › Figure 1/Figure 1D/README.pptx]

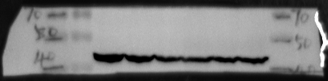

Supplement: Supplementary file 2 — Source data Fig. 1 [file 44321_2025_318_MOESM2_ESM.zip › Figure 1/Figure 1D/Western GAPDH.tif]

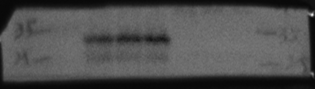

Supplement: Supplementary file 2 — Source data Fig. 1 [file 44321_2025_318_MOESM2_ESM.zip › Figure 1/Figure 1D/Western Ucp-1.tif]

## Slide 1
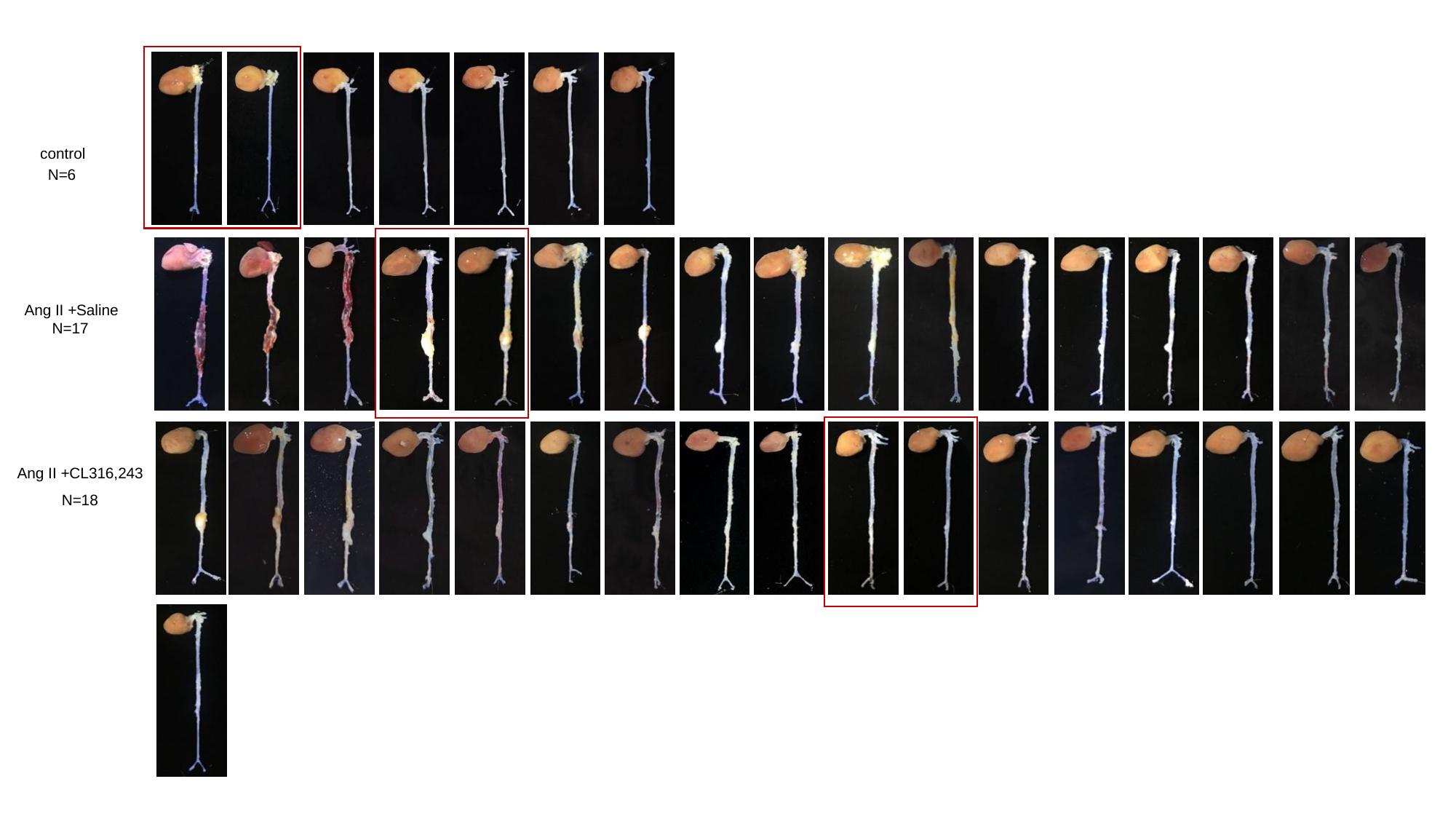

control
N=6
Ang II +Saline
N=17
Ang II +CL316,243
N=18

Supplement: Supplementary file 2 — Source data Fig. 1 [file 44321_2025_318_MOESM2_ESM.zip › Figure 1/Figure 1K/Whole mount.pptx]

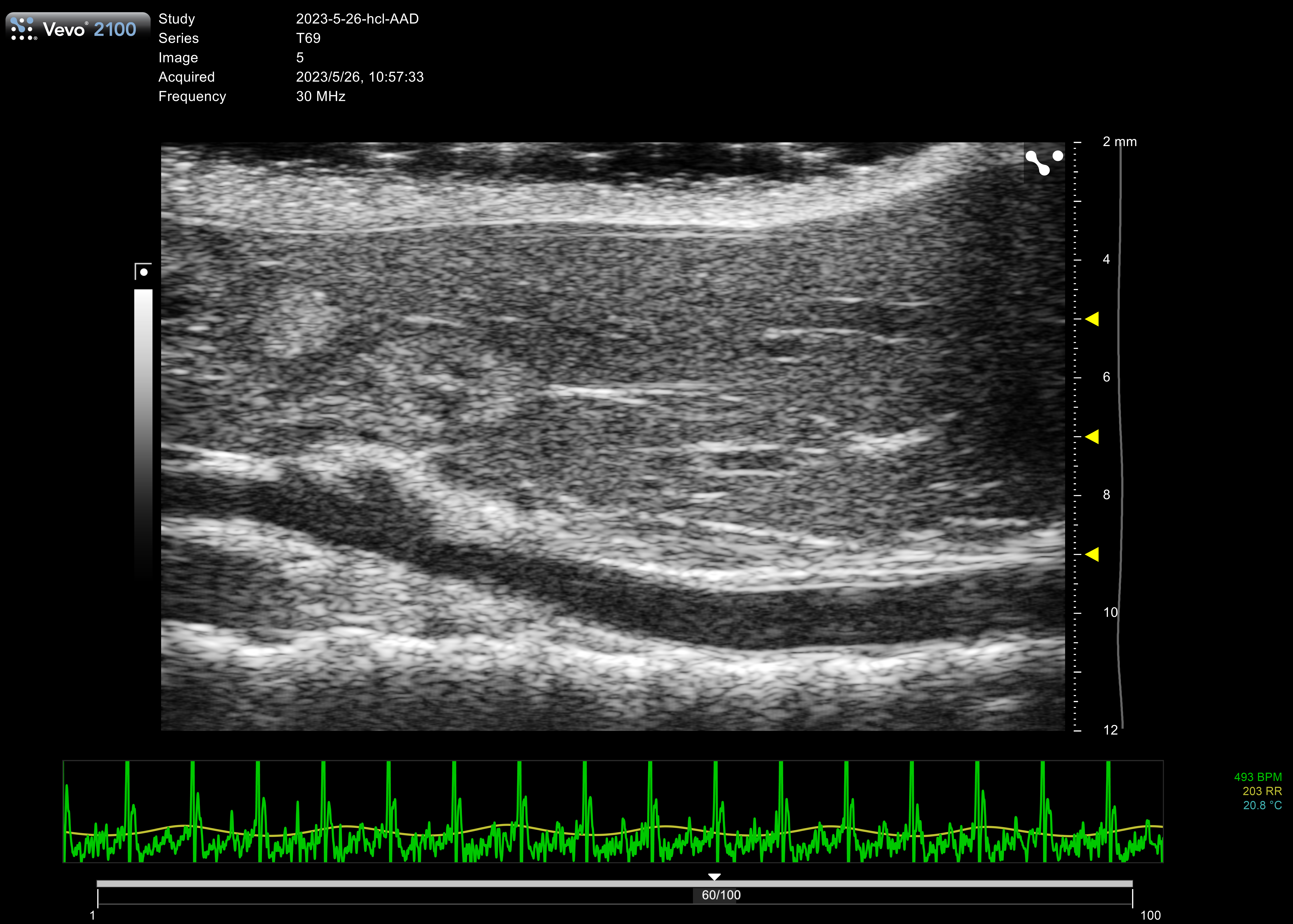

Supplement: Supplementary file 2 — Source data Fig. 1 [file 44321_2025_318_MOESM2_ESM.zip › Figure 1/Figure 1L/Figure 1L Ang II+CL316,243.tif]

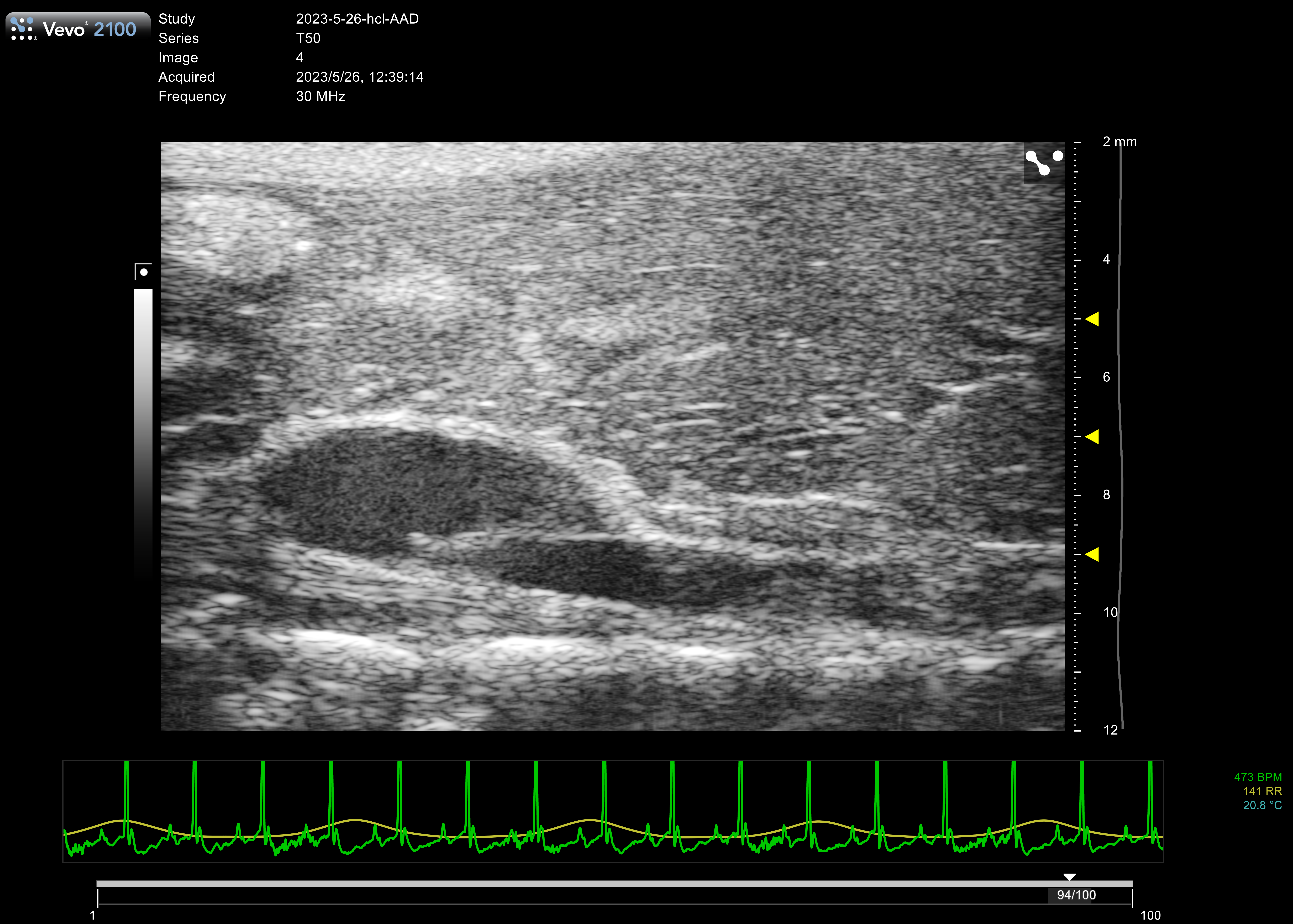

Supplement: Supplementary file 2 — Source data Fig. 1 [file 44321_2025_318_MOESM2_ESM.zip › Figure 1/Figure 1L/Figure 1L Ang II+Saline.tif]

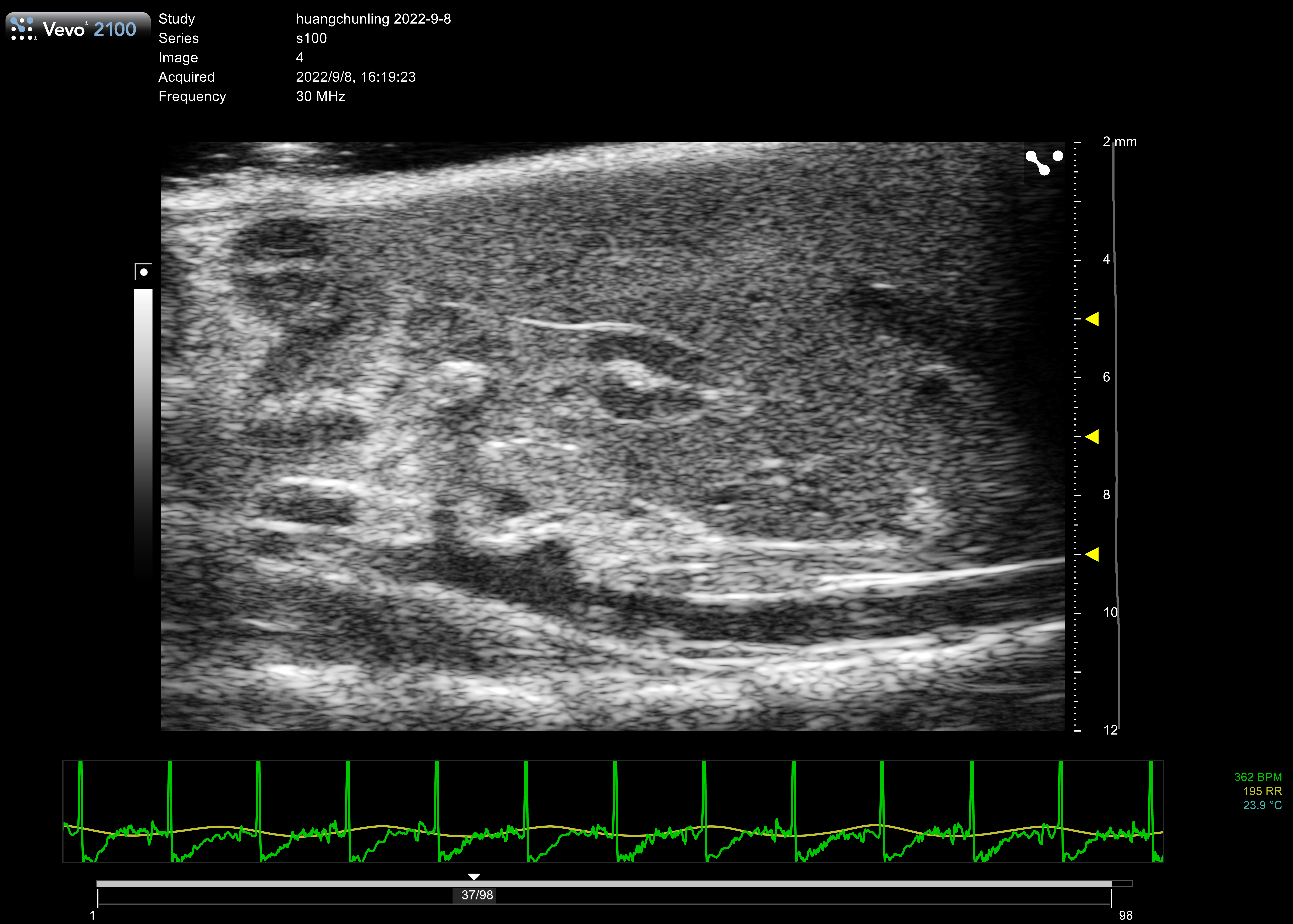

Supplement: Supplementary file 2 — Source data Fig. 1 [file 44321_2025_318_MOESM2_ESM.zip › Figure 1/Figure 1L/Figure 1L Control.tif]

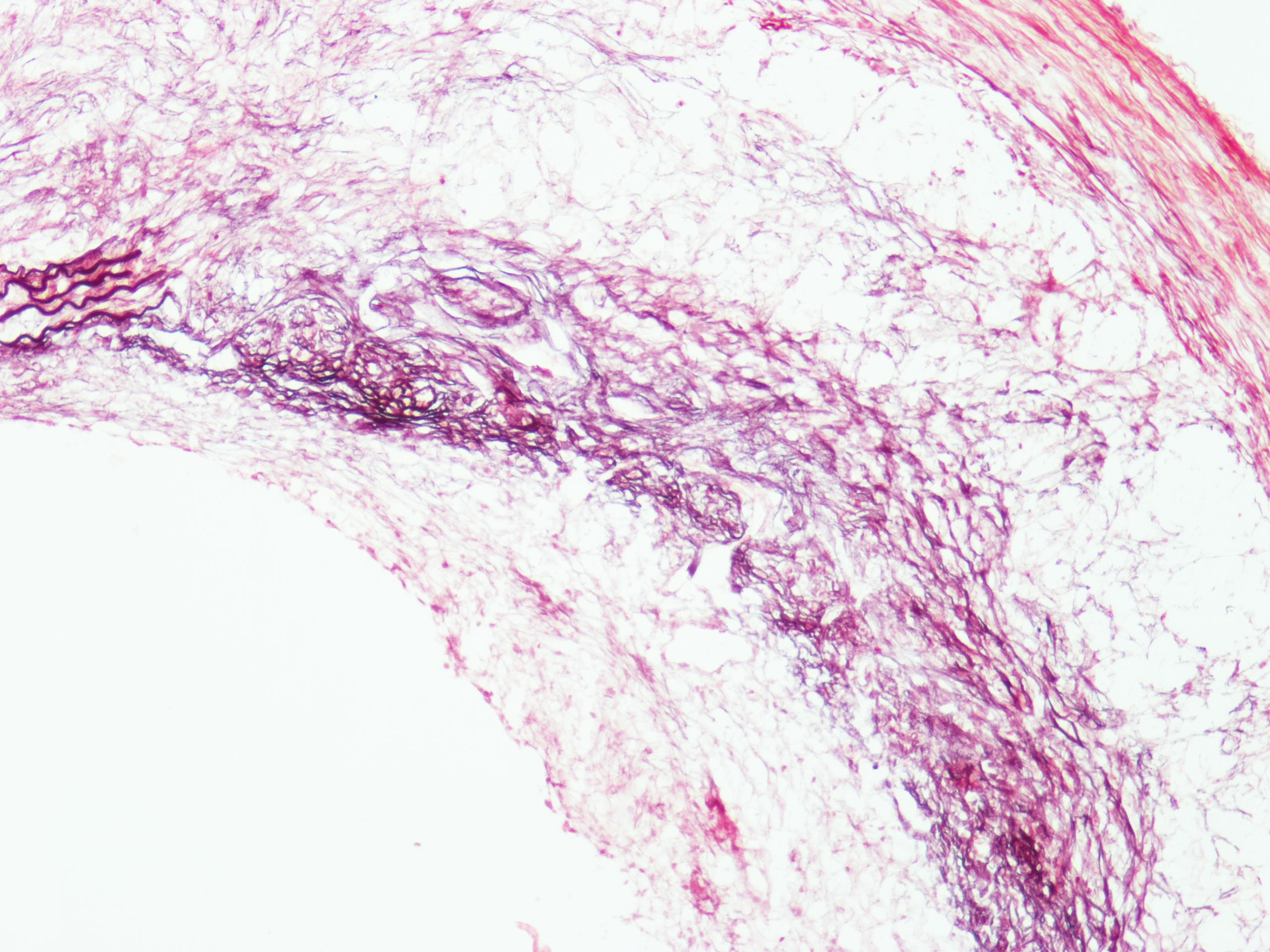

Supplement: Supplementary file 2 — Source data Fig. 1 [file 44321_2025_318_MOESM2_ESM.zip › Figure 1/Figure 1N/EVG staining/Ang II + Saline 100um.tif]

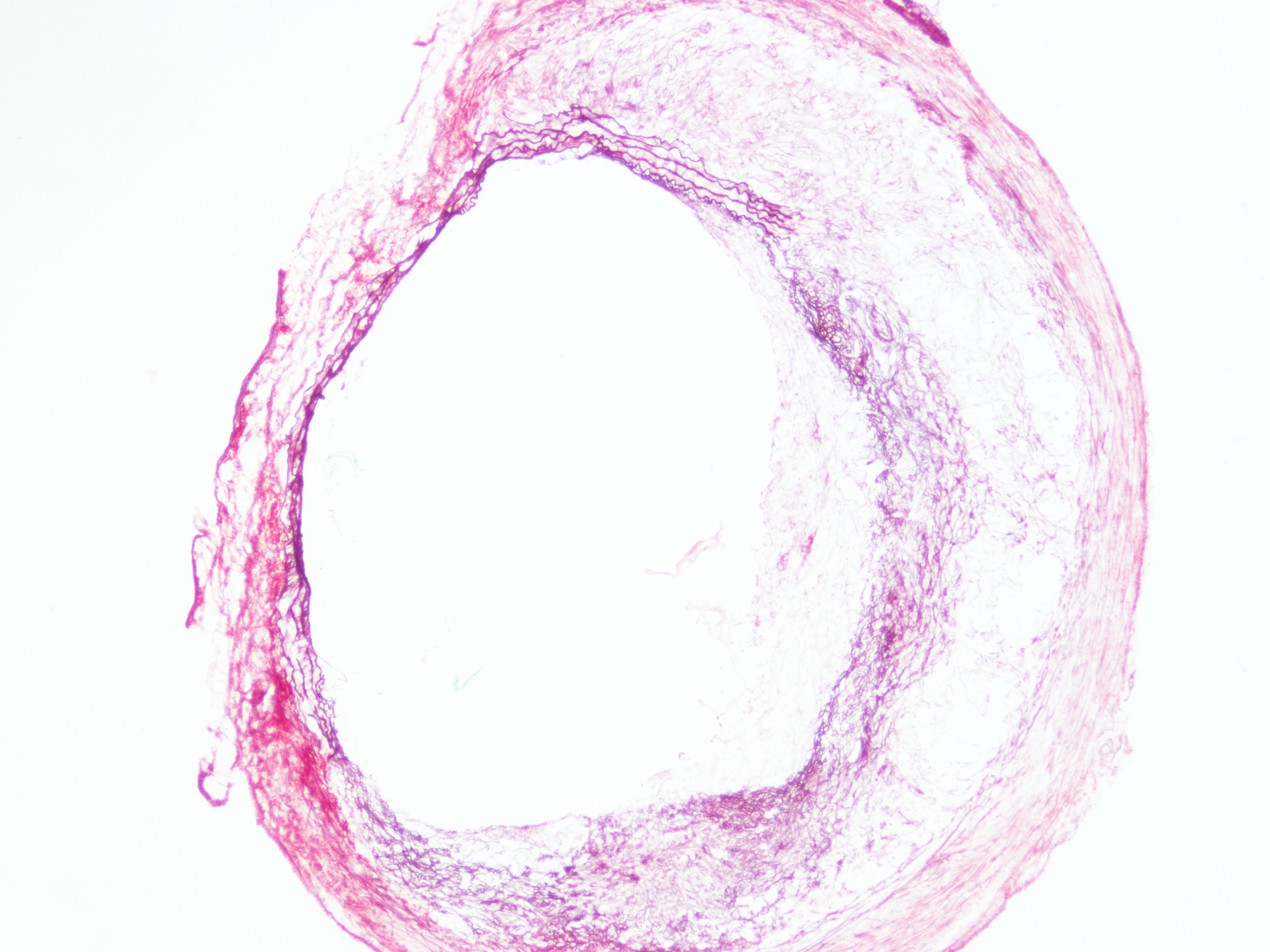

Supplement: Supplementary file 2 — Source data Fig. 1 [file 44321_2025_318_MOESM2_ESM.zip › Figure 1/Figure 1N/EVG staining/Ang II + Saline 200um.tif]

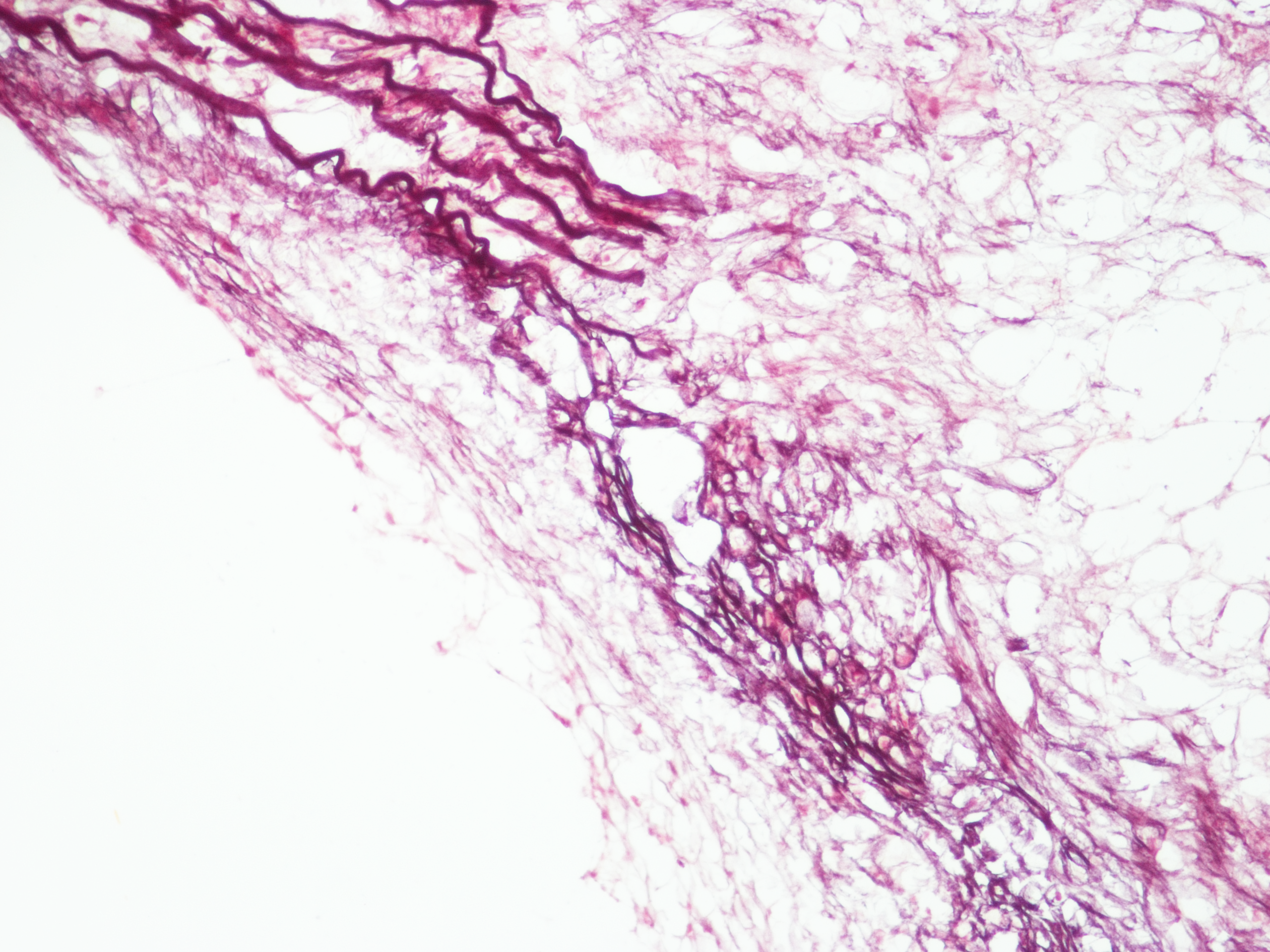

Supplement: Supplementary file 2 — Source data Fig. 1 [file 44321_2025_318_MOESM2_ESM.zip › Figure 1/Figure 1N/EVG staining/Ang II + Saline 50um.tif]

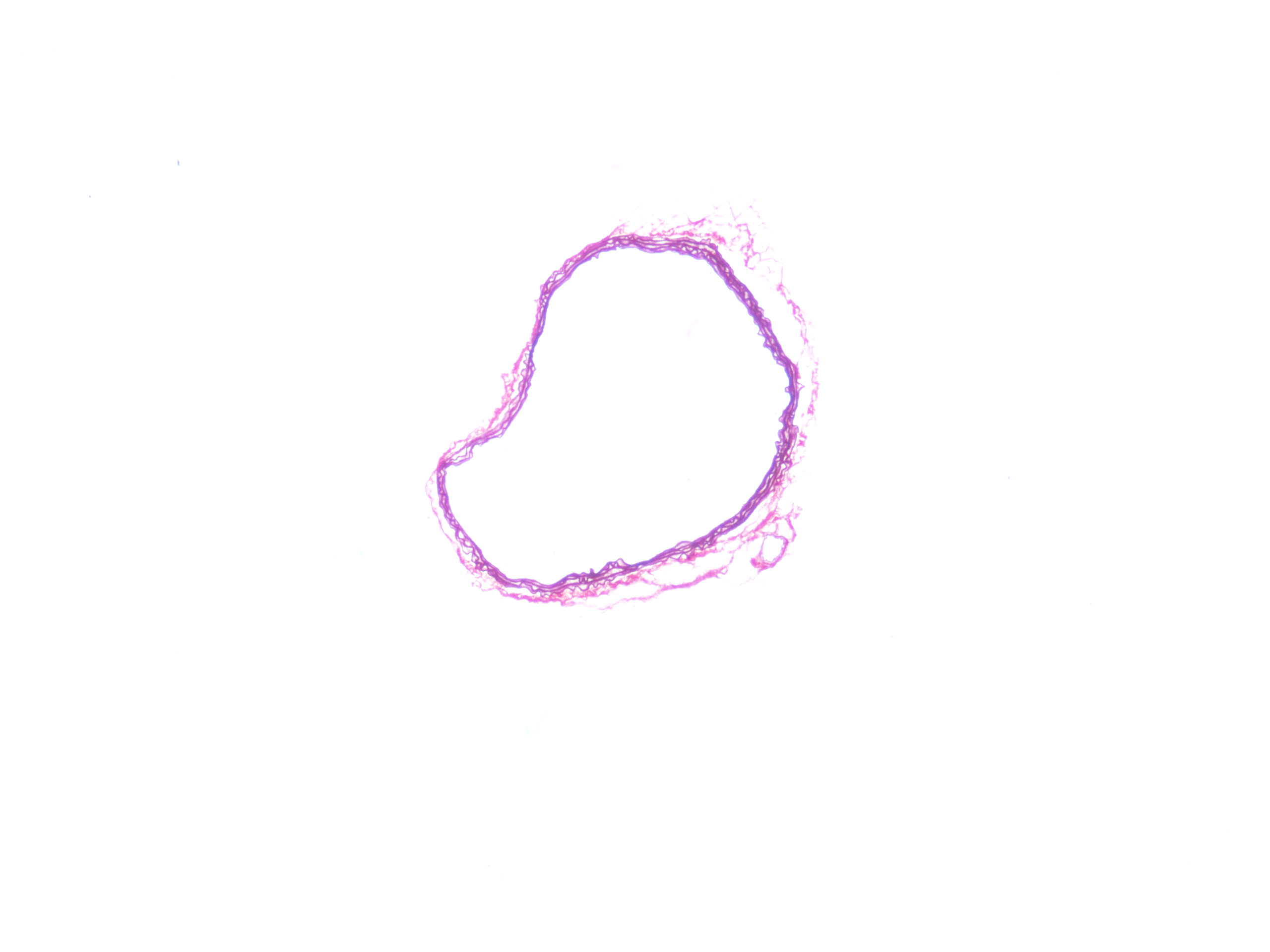

Supplement: Supplementary file 2 — Source data Fig. 1 [file 44321_2025_318_MOESM2_ESM.zip › Figure 1/Figure 1N/EVG staining/Ang II +CL316,423 200um.tiff]

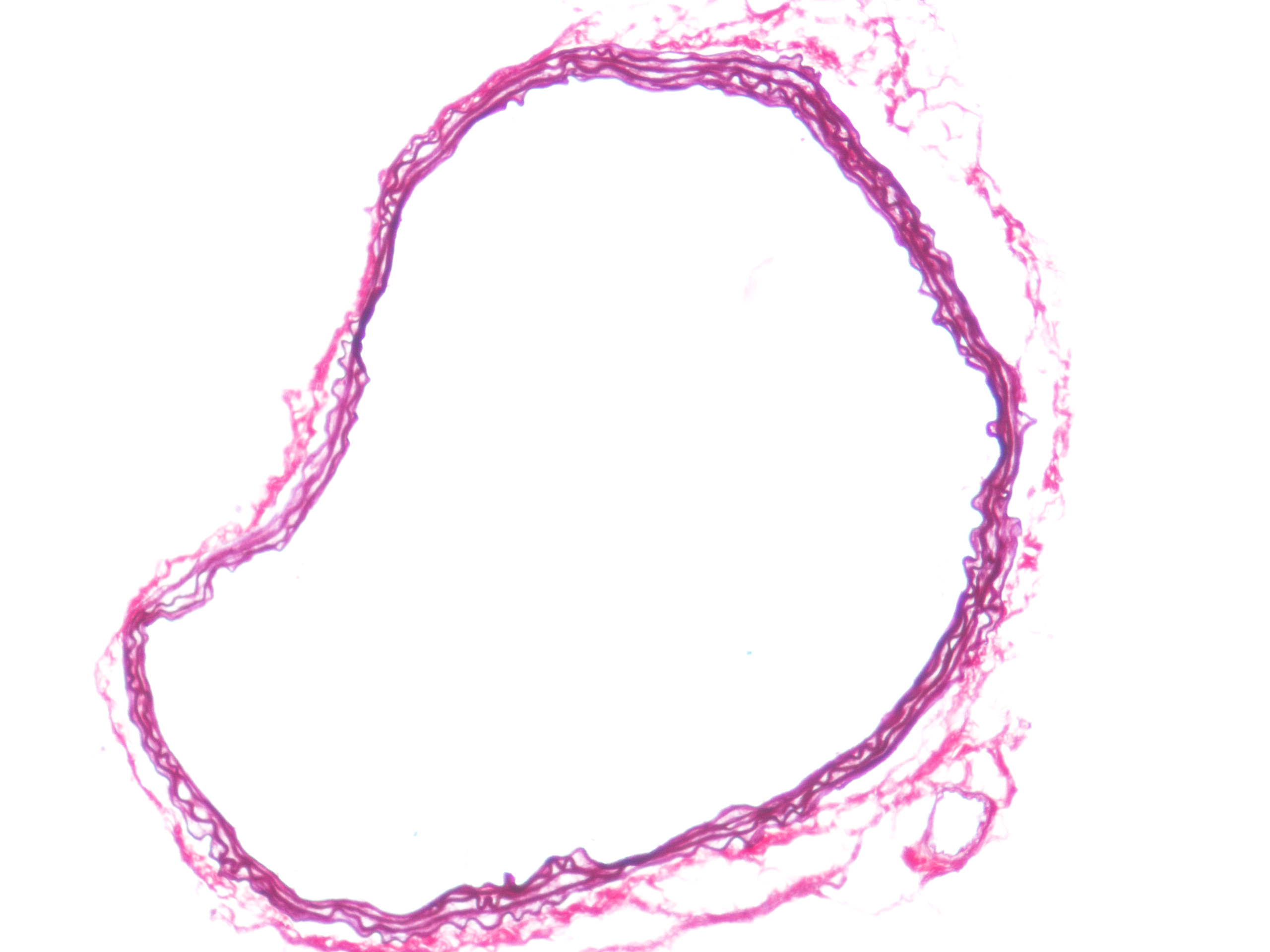

Supplement: Supplementary file 2 — Source data Fig. 1 [file 44321_2025_318_MOESM2_ESM.zip › Figure 1/Figure 1N/EVG staining/AngII+CL316,243 100um.tiff]

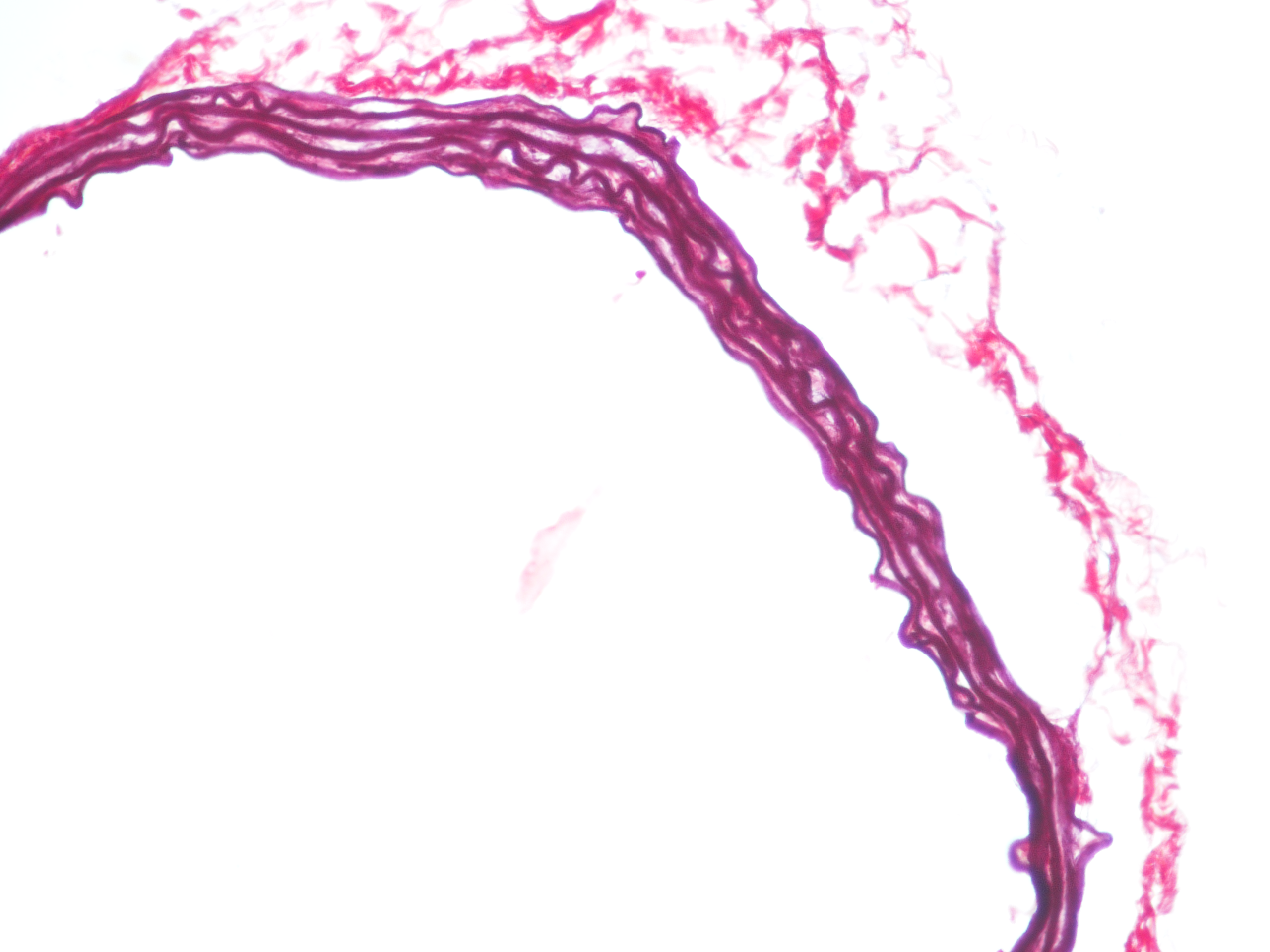

Supplement: Supplementary file 2 — Source data Fig. 1 [file 44321_2025_318_MOESM2_ESM.zip › Figure 1/Figure 1N/EVG staining/AngII+CL316,243 50um.tiff]

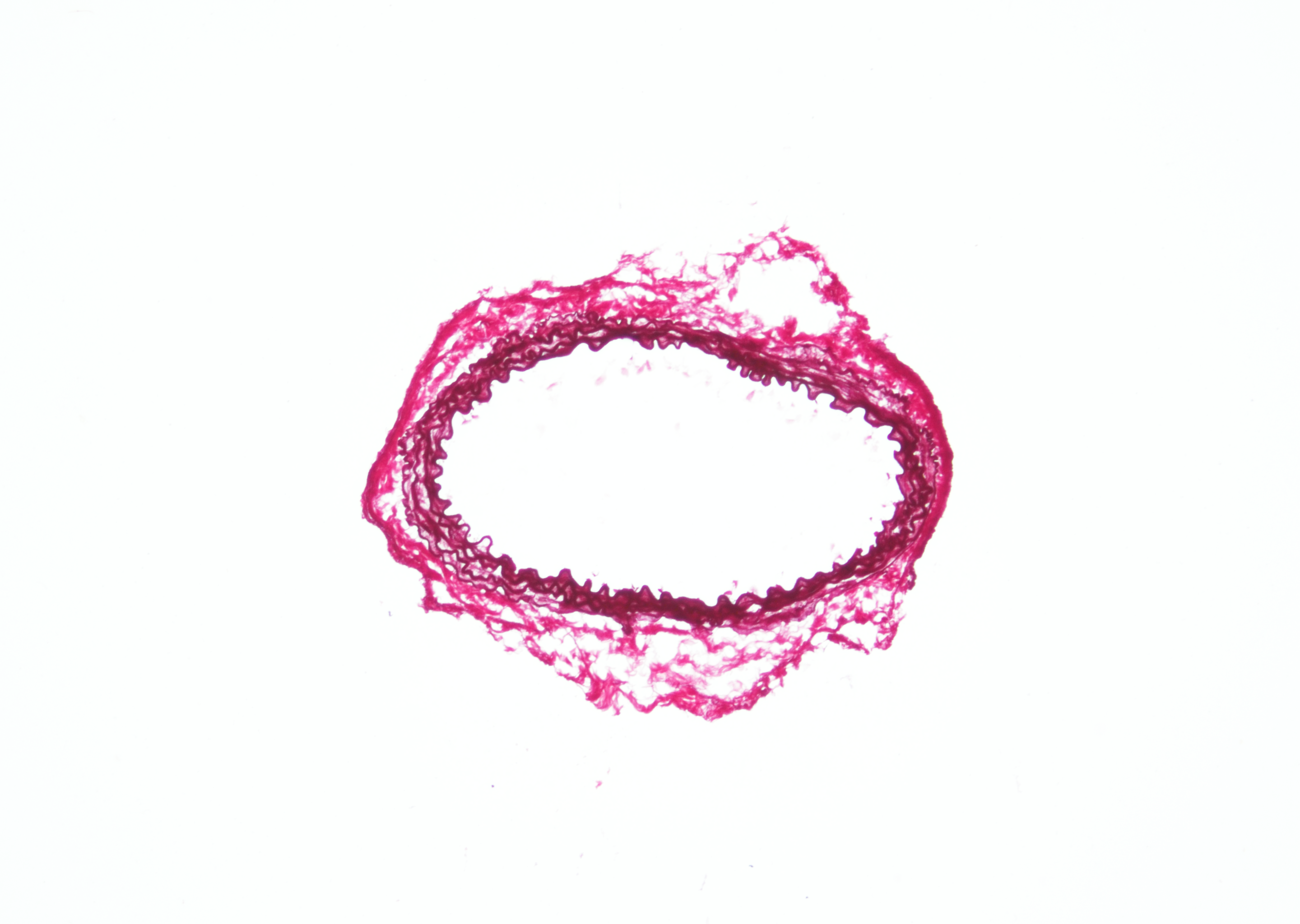

Supplement: Supplementary file 2 — Source data Fig. 1 [file 44321_2025_318_MOESM2_ESM.zip › Figure 1/Figure 1N/EVG staining/Control 100um.tif]

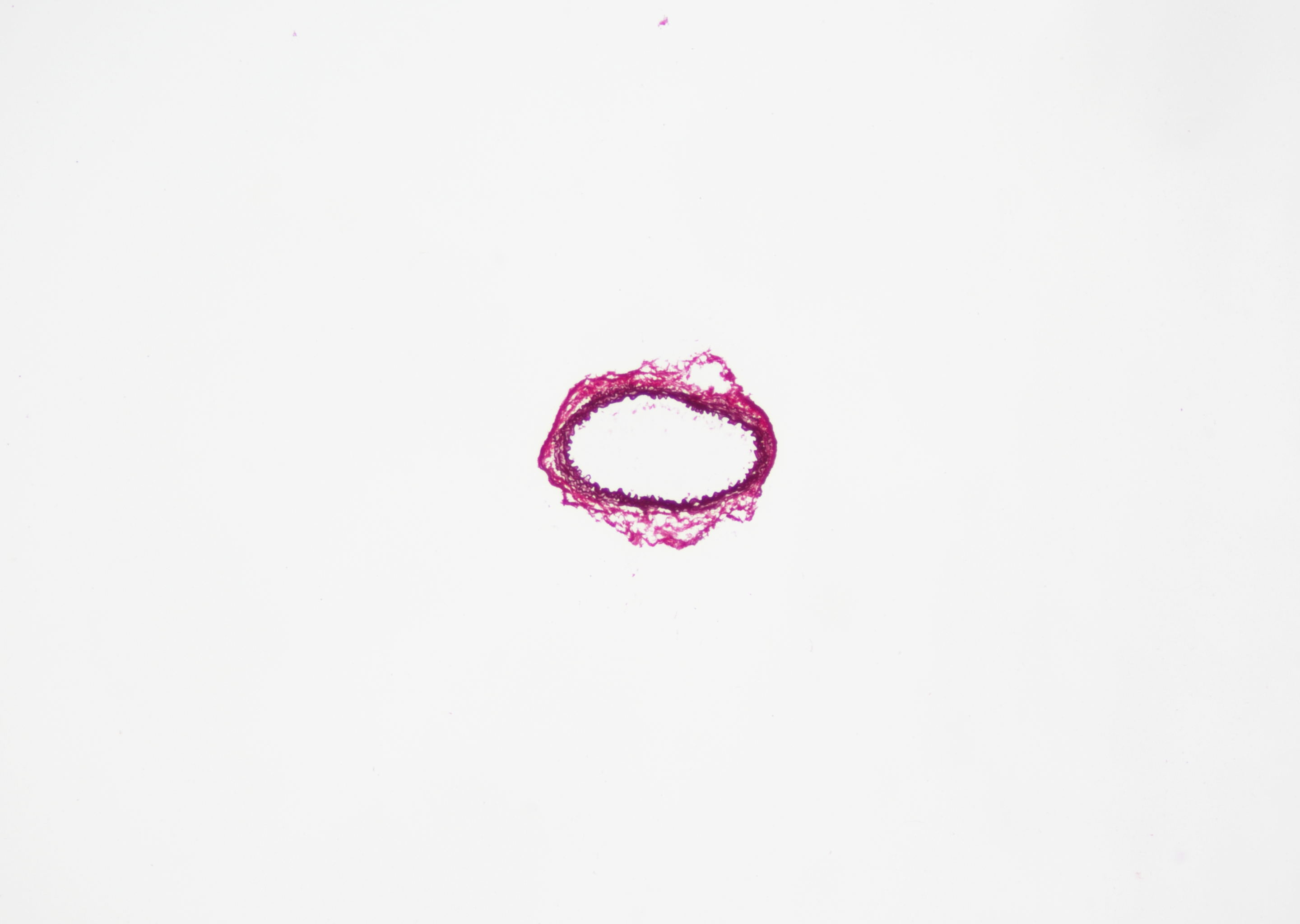

Supplement: Supplementary file 2 — Source data Fig. 1 [file 44321_2025_318_MOESM2_ESM.zip › Figure 1/Figure 1N/EVG staining/Control 200um.tif]

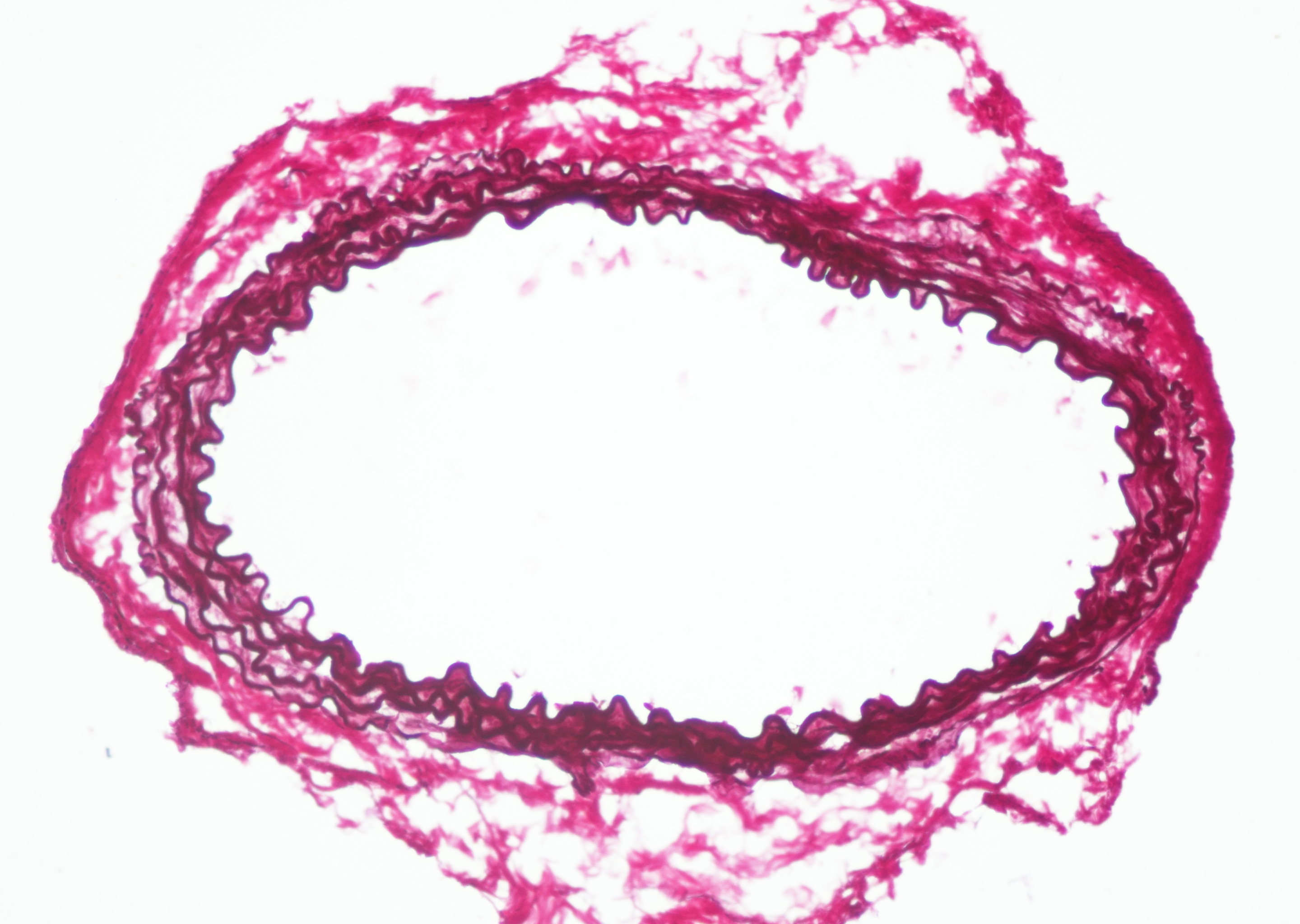

Supplement: Supplementary file 2 — Source data Fig. 1 [file 44321_2025_318_MOESM2_ESM.zip › Figure 1/Figure 1N/EVG staining/Control 50um.tif]

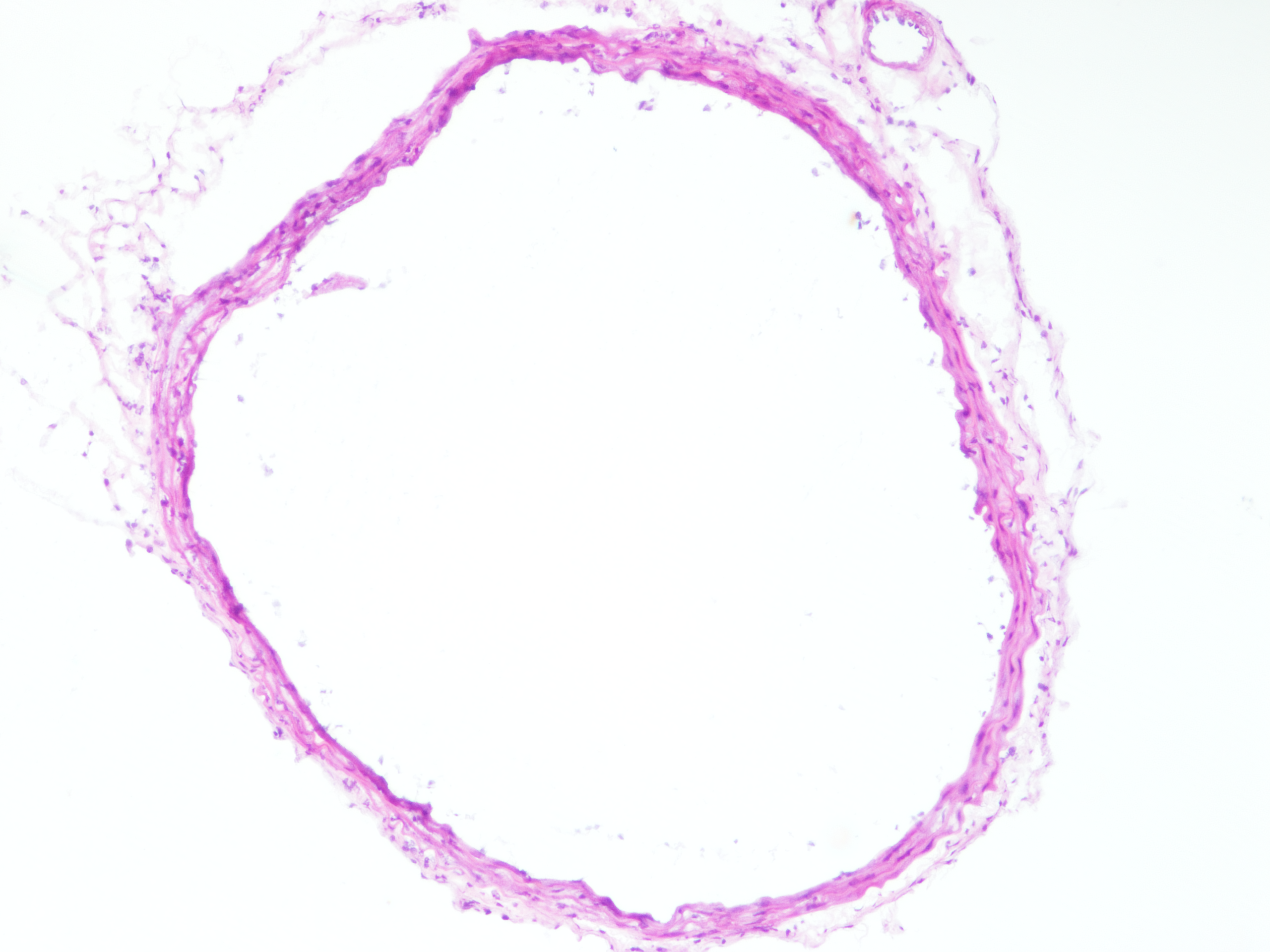

Supplement: Supplementary file 2 — Source data Fig. 1 [file 44321_2025_318_MOESM2_ESM.zip › Figure 1/Figure 1N/HE staining/Ang II+CL316,243 100um.tif]

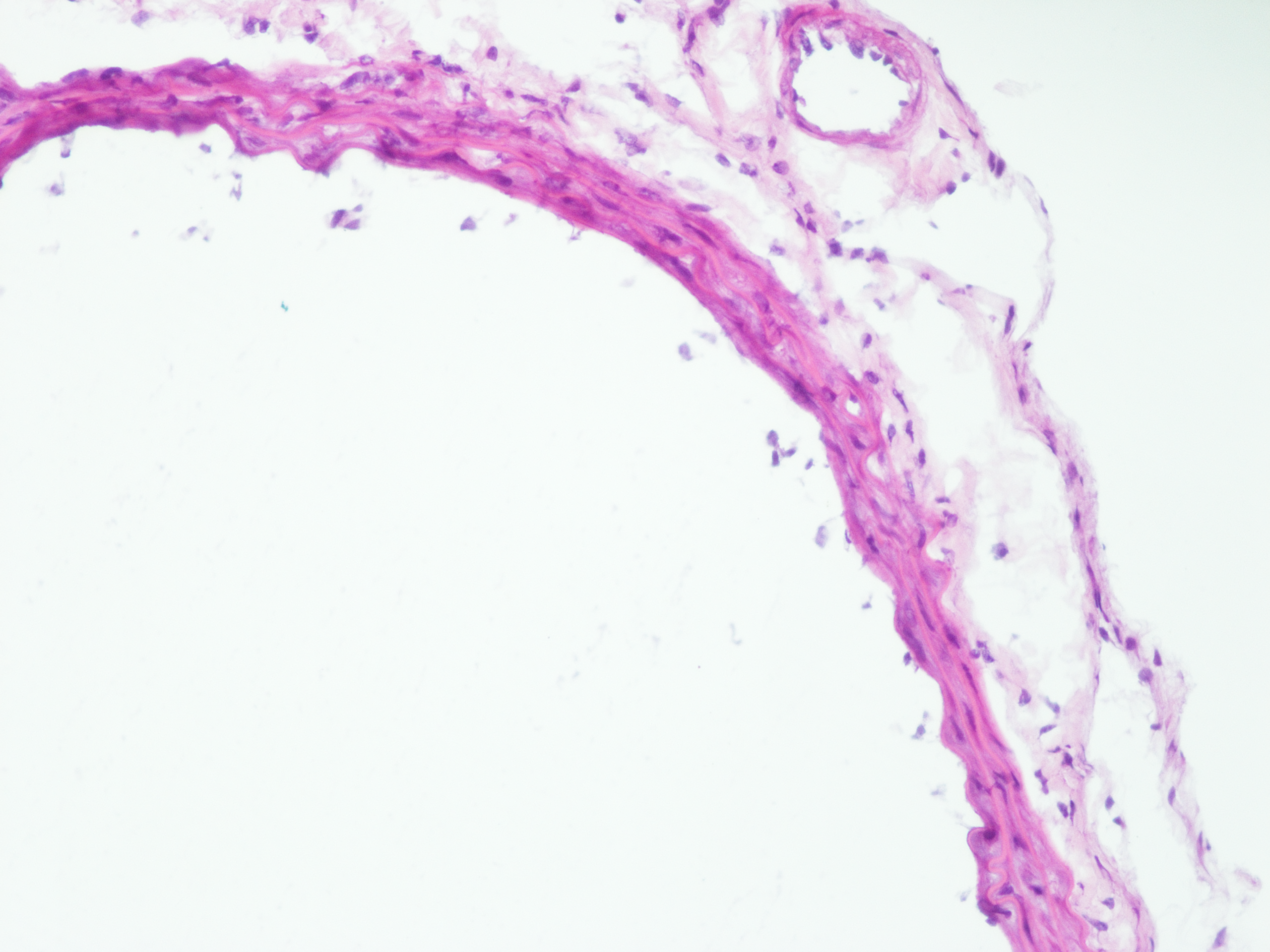

Supplement: Supplementary file 2 — Source data Fig. 1 [file 44321_2025_318_MOESM2_ESM.zip › Figure 1/Figure 1N/HE staining/Ang II+CL316,243 50um.tif]

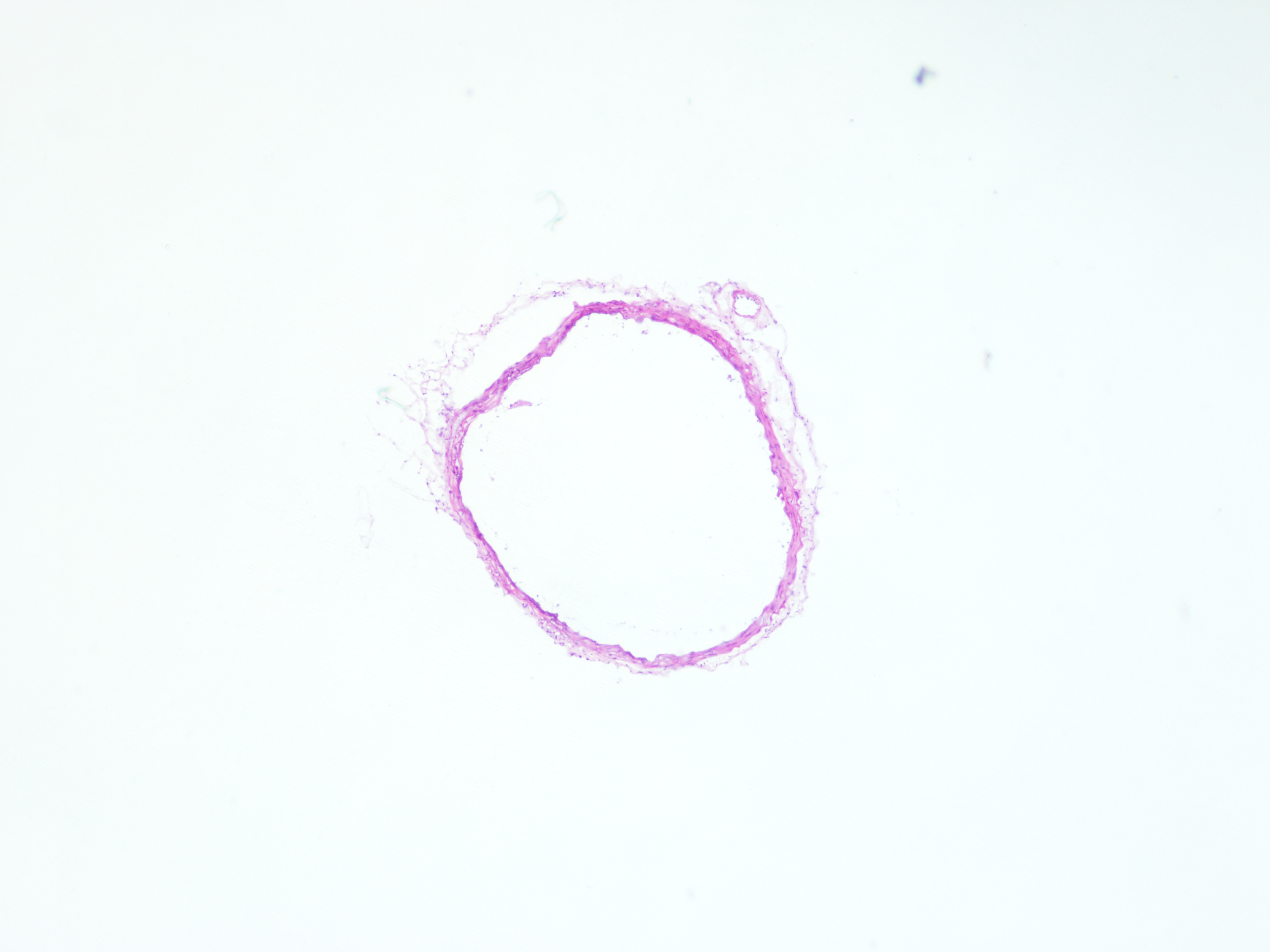

Supplement: Supplementary file 2 — Source data Fig. 1 [file 44321_2025_318_MOESM2_ESM.zip › Figure 1/Figure 1N/HE staining/AngII+CL316,243 200um.tif]

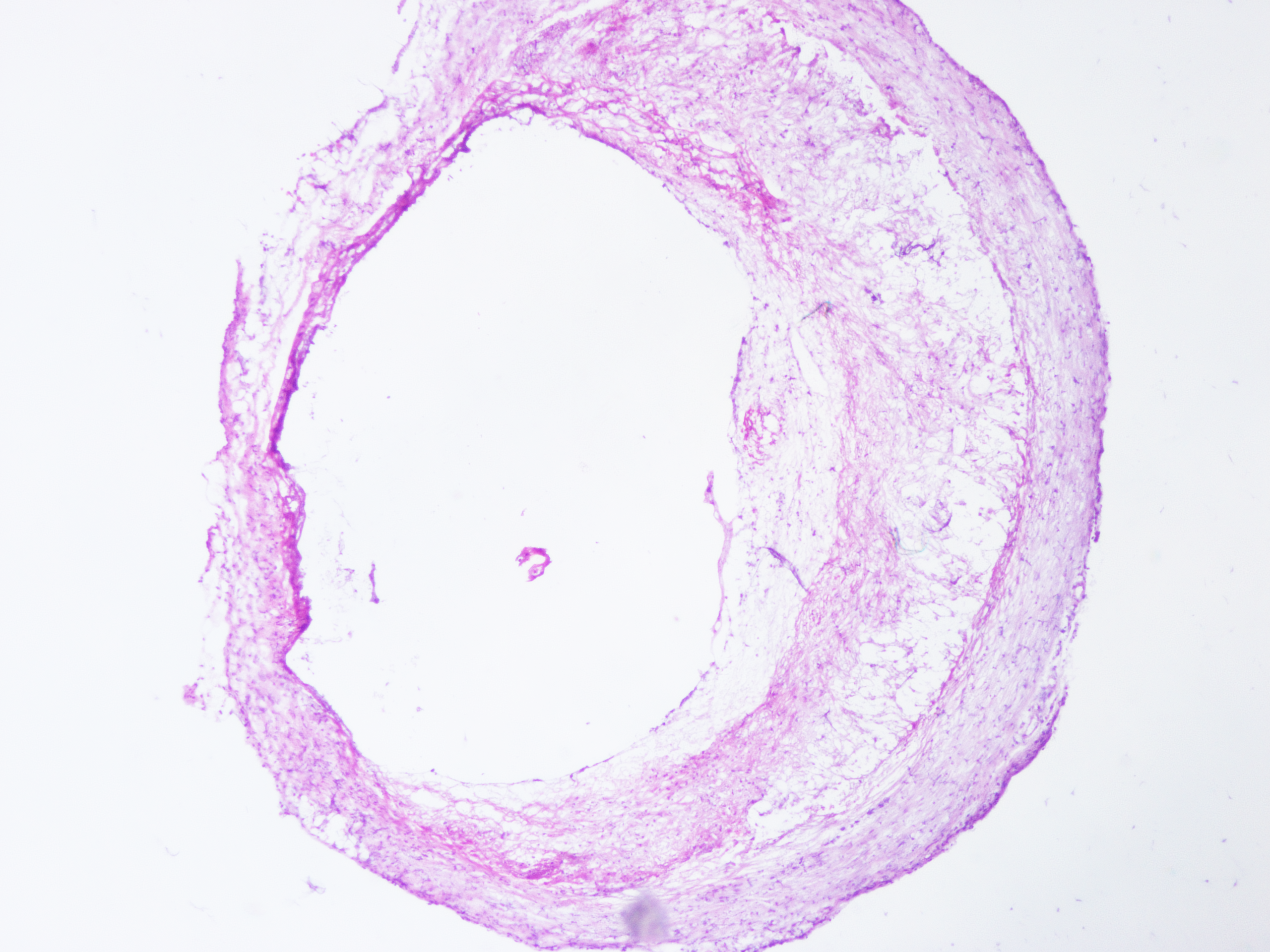

Supplement: Supplementary file 2 — Source data Fig. 1 [file 44321_2025_318_MOESM2_ESM.zip › Figure 1/Figure 1N/HE staining/AngII+Saline 200um.tif]

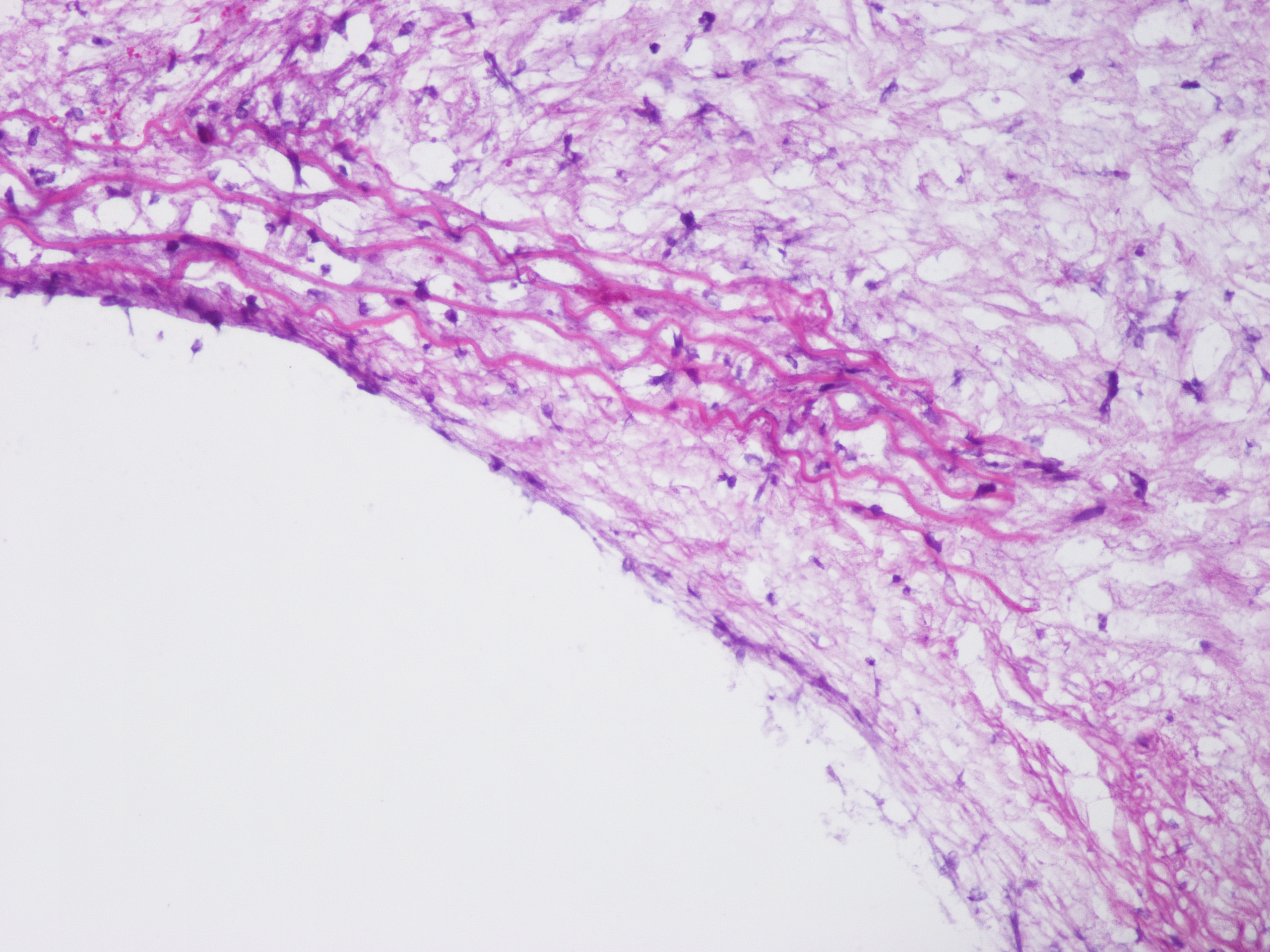

Supplement: Supplementary file 2 — Source data Fig. 1 [file 44321_2025_318_MOESM2_ESM.zip › Figure 1/Figure 1N/HE staining/AngII+Saline 50um.tif]

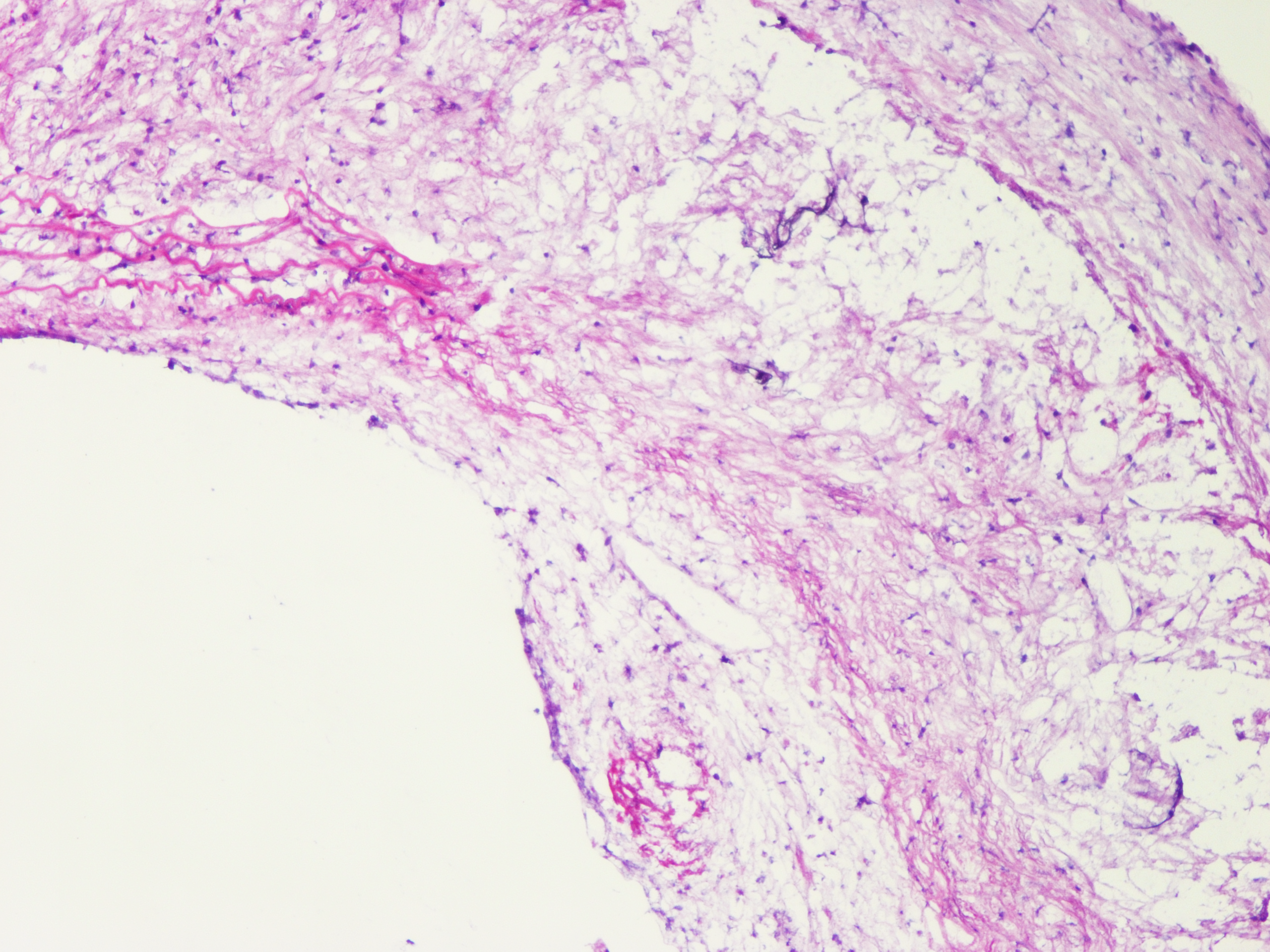

Supplement: Supplementary file 2 — Source data Fig. 1 [file 44321_2025_318_MOESM2_ESM.zip › Figure 1/Figure 1N/HE staining/AngII+Saline 100um.tif]

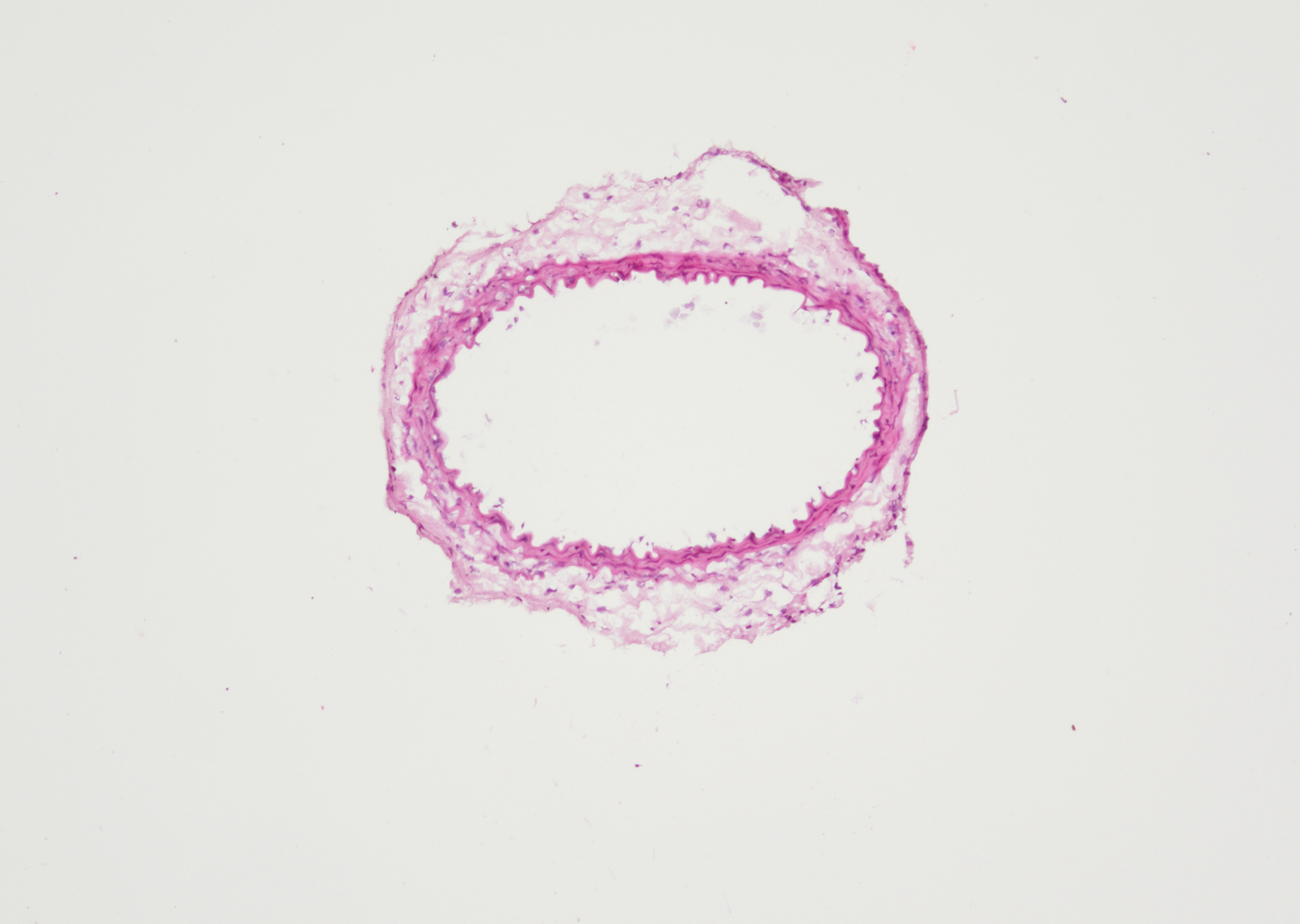

Supplement: Supplementary file 2 — Source data Fig. 1 [file 44321_2025_318_MOESM2_ESM.zip › Figure 1/Figure 1N/HE staining/Control 100um.tif]

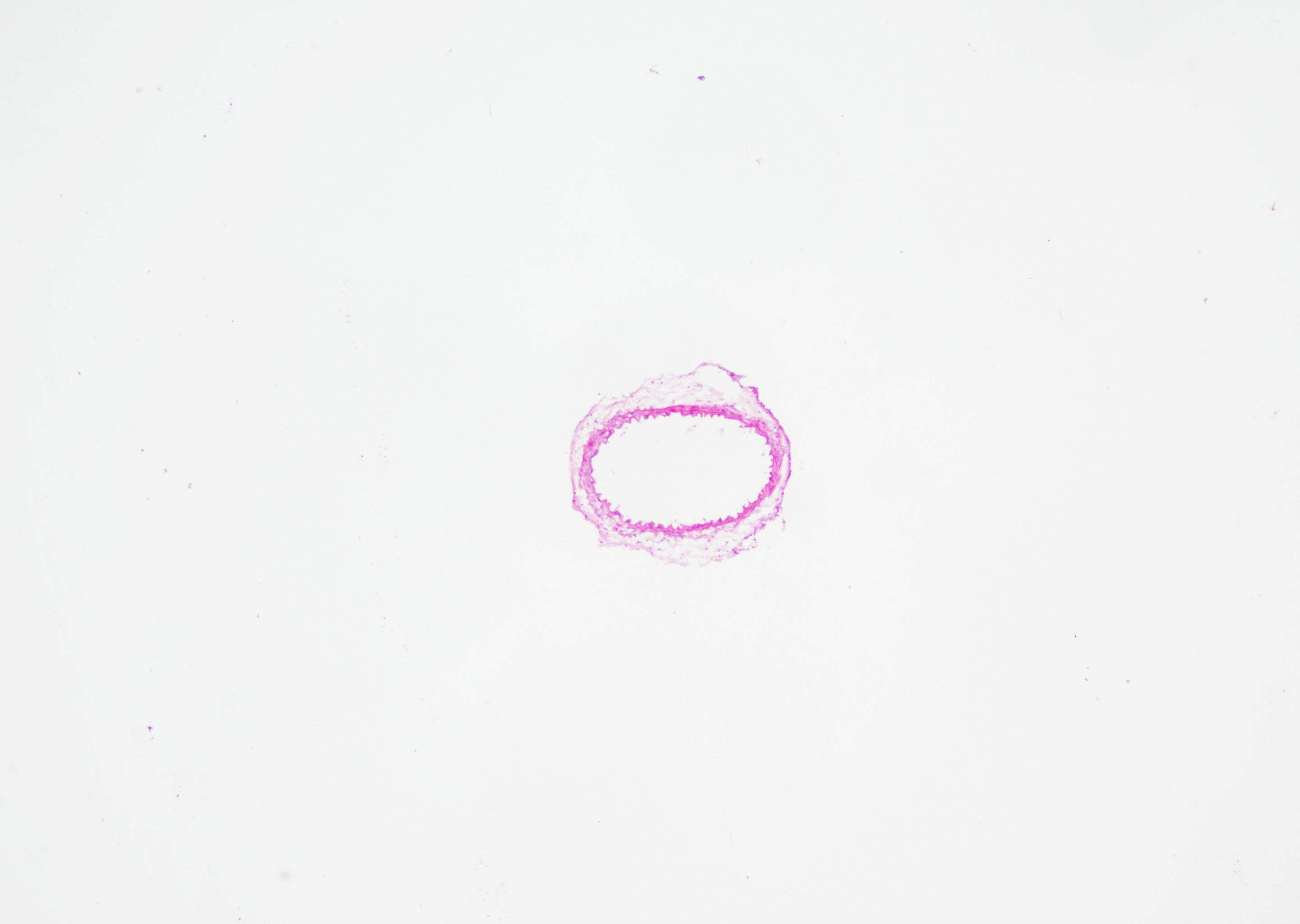

Supplement: Supplementary file 2 — Source data Fig. 1 [file 44321_2025_318_MOESM2_ESM.zip › Figure 1/Figure 1N/HE staining/Control 200um.tif]

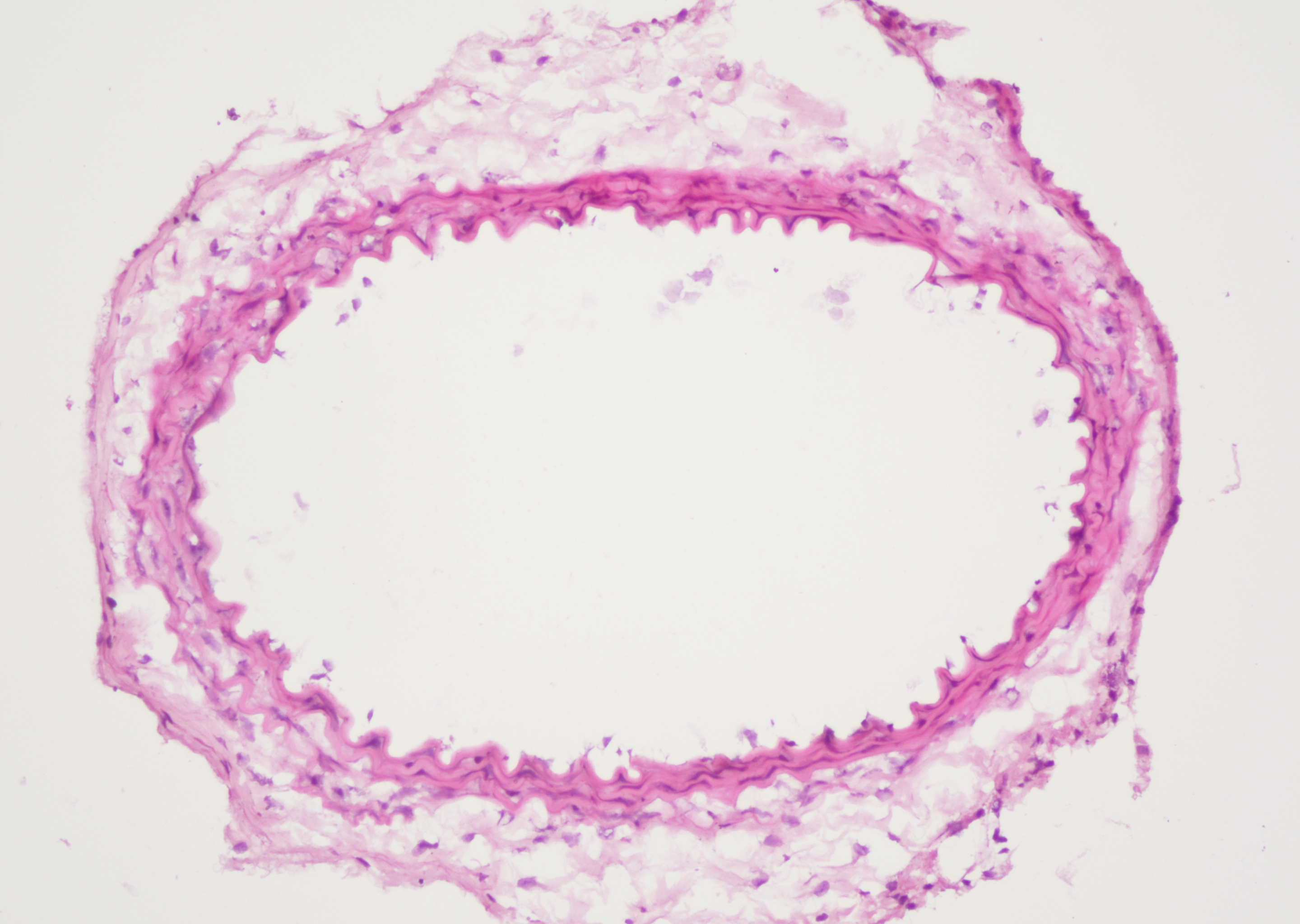

Supplement: Supplementary file 2 — Source data Fig. 1 [file 44321_2025_318_MOESM2_ESM.zip › Figure 1/Figure 1N/HE staining/Control 50um.tif]

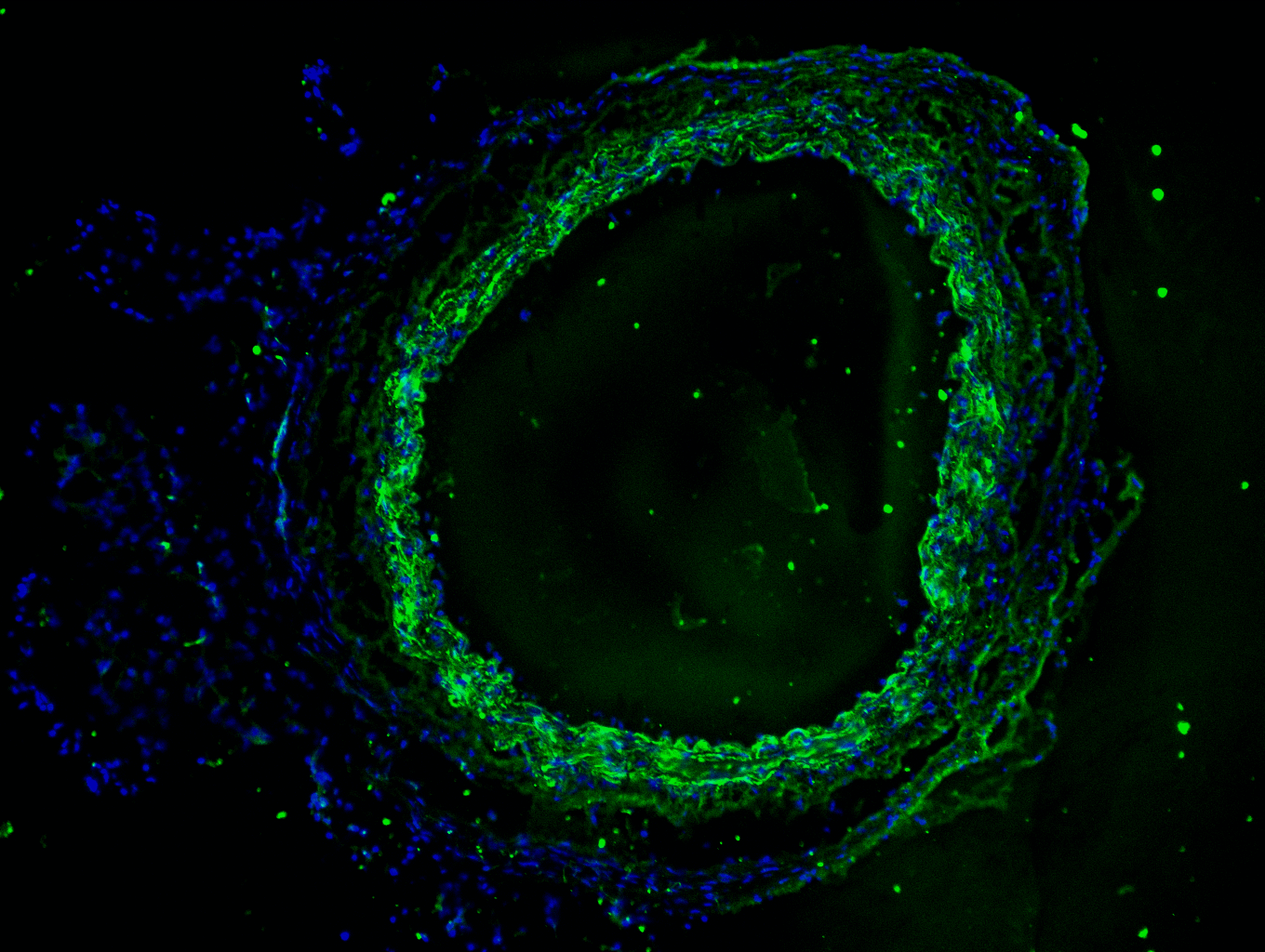

Supplement: Supplementary file 3 — Source data Fig. 2 [file 44321_2025_318_MOESM3_ESM.zip › Figure 2/Figure 2C/aSMA-AngII + CL316,243-1.tif]

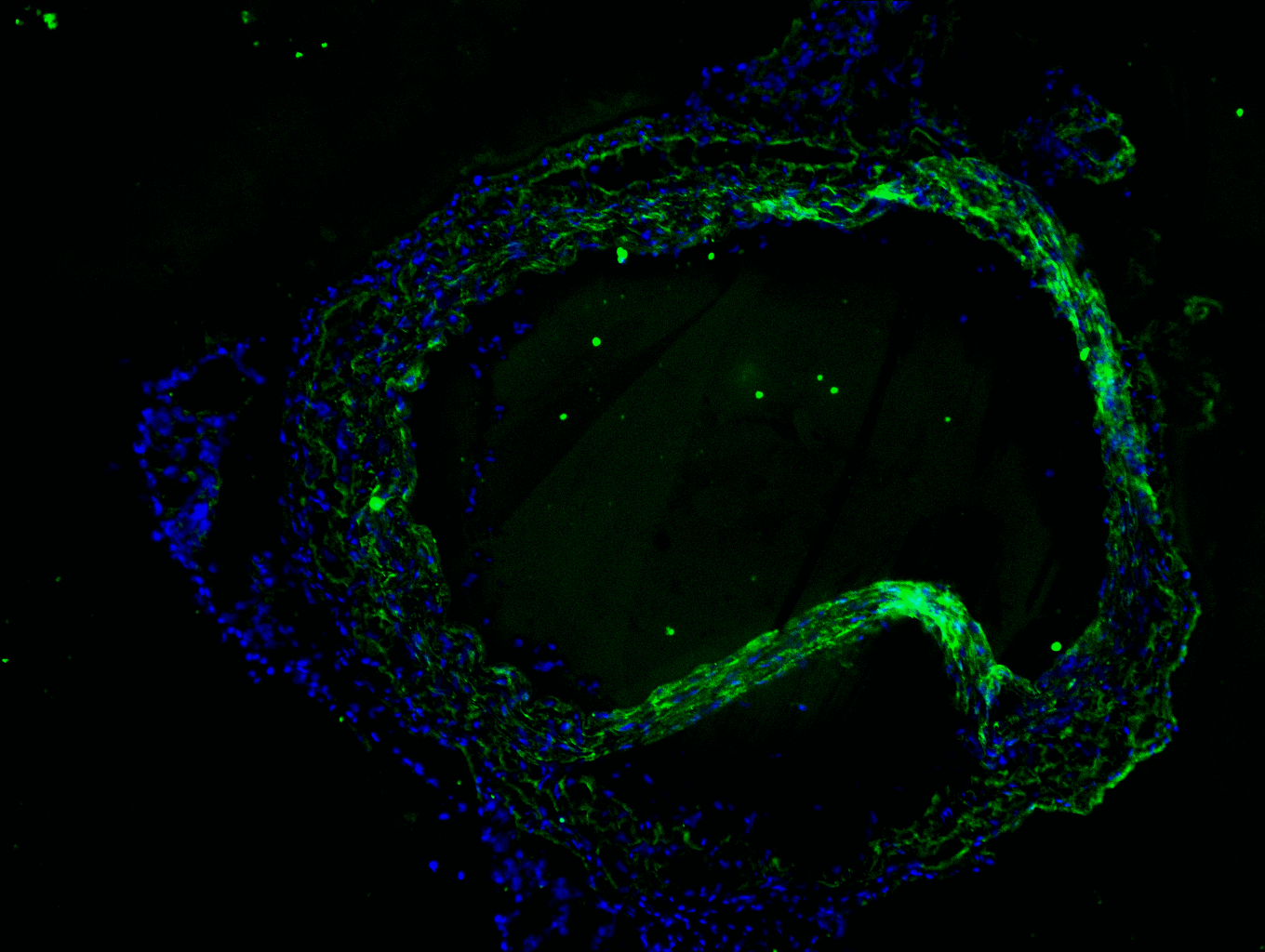

Supplement: Supplementary file 3 — Source data Fig. 2 [file 44321_2025_318_MOESM3_ESM.zip › Figure 2/Figure 2C/aSMA-AngII + Saline.tif]

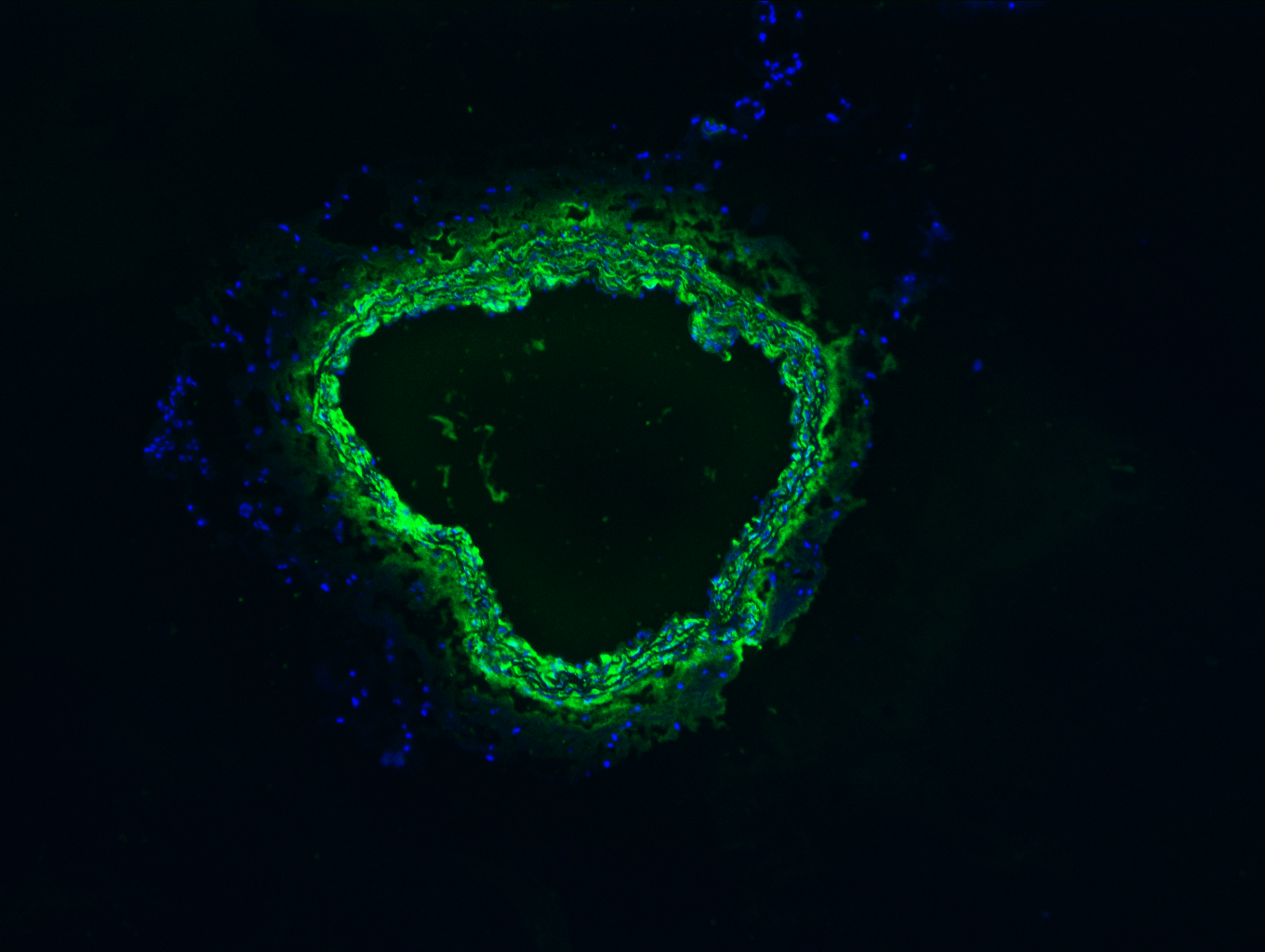

Supplement: Supplementary file 3 — Source data Fig. 2 [file 44321_2025_318_MOESM3_ESM.zip › Figure 2/Figure 2C/aSMA-Control.tif]

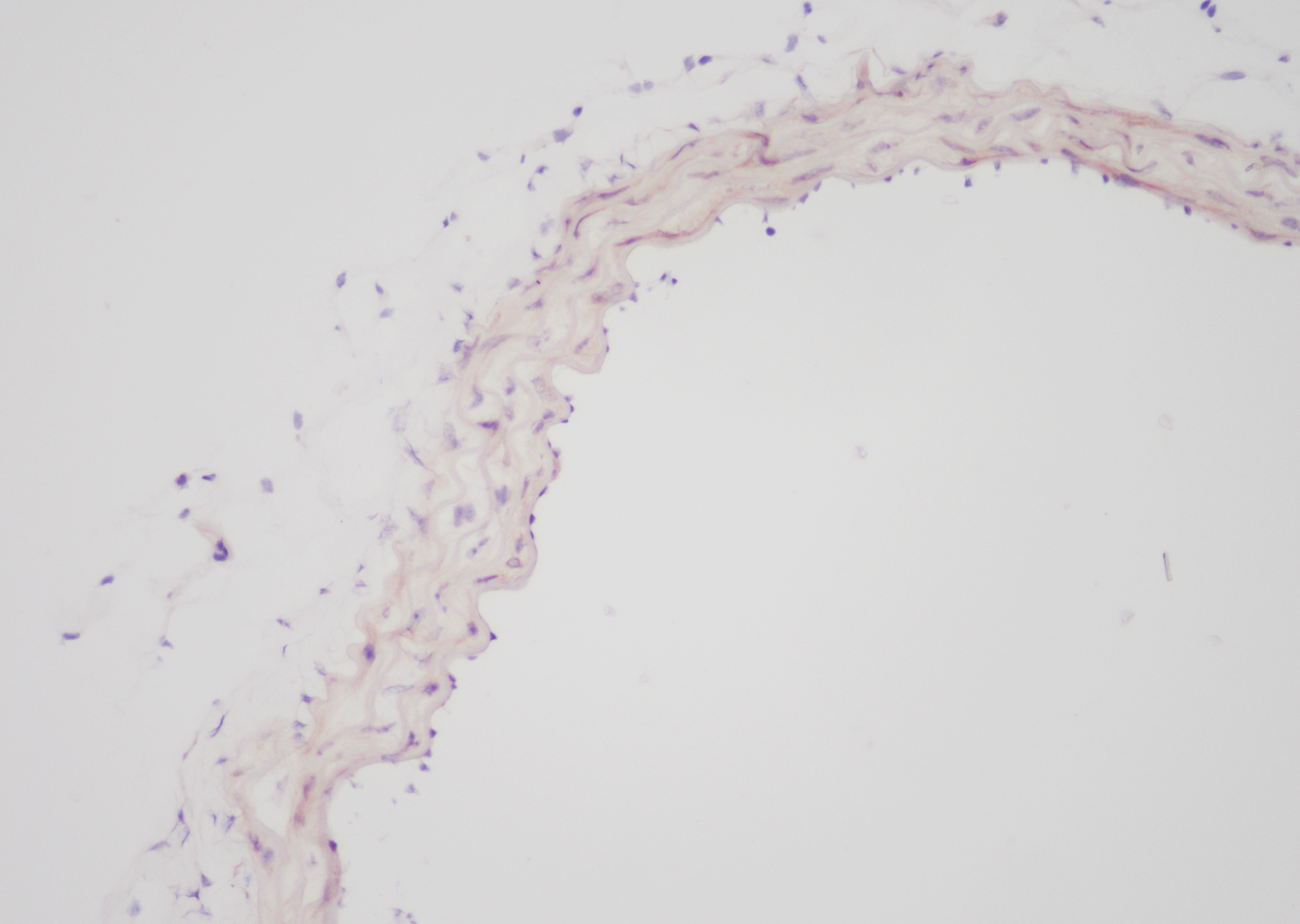

Supplement: Supplementary file 3 — Source data Fig. 2 [file 44321_2025_318_MOESM3_ESM.zip › Figure 2/Figure 2E/AngII+CL316,243.tif]

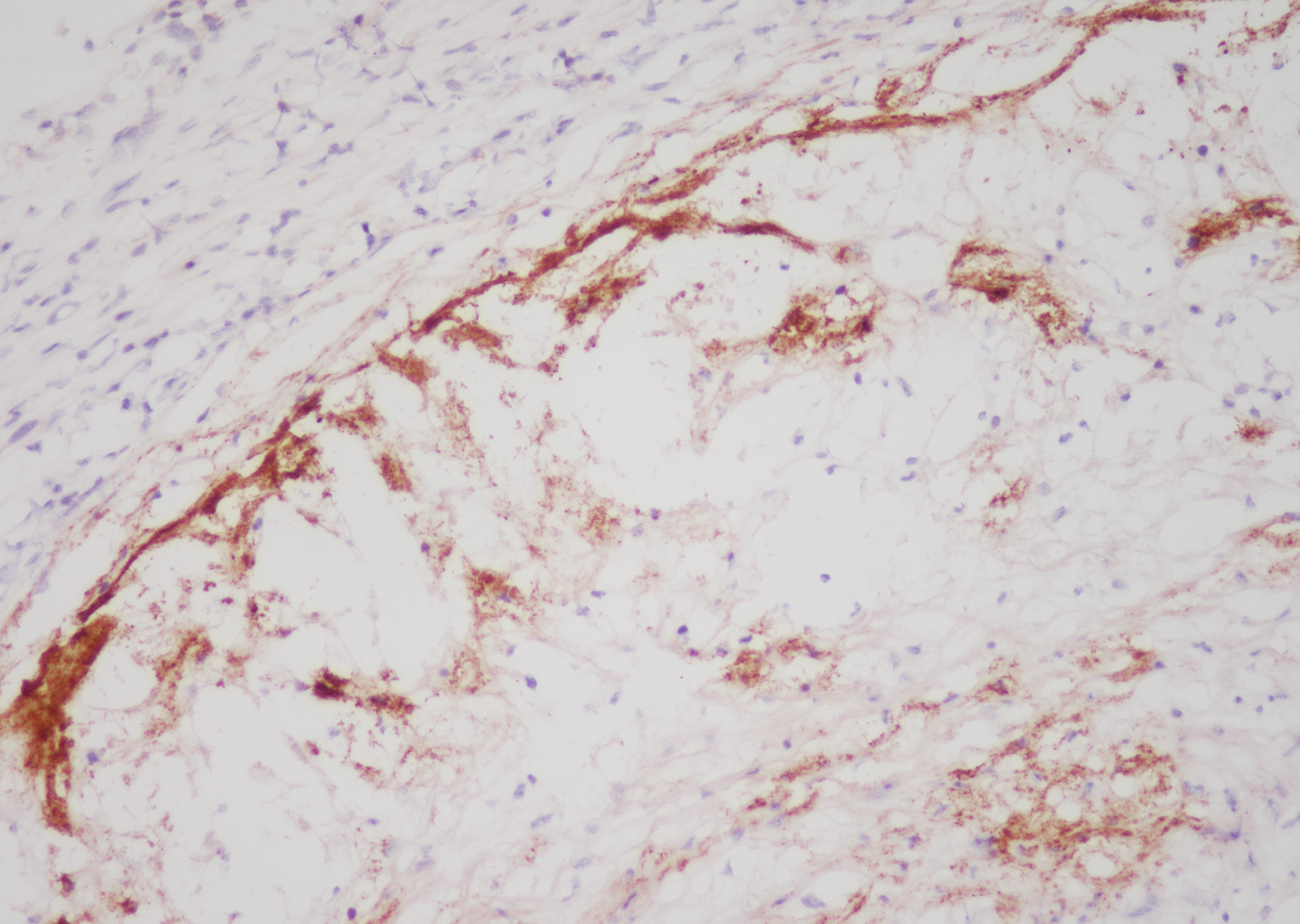

Supplement: Supplementary file 3 — Source data Fig. 2 [file 44321_2025_318_MOESM3_ESM.zip › Figure 2/Figure 2E/AngII+Saline.tif]

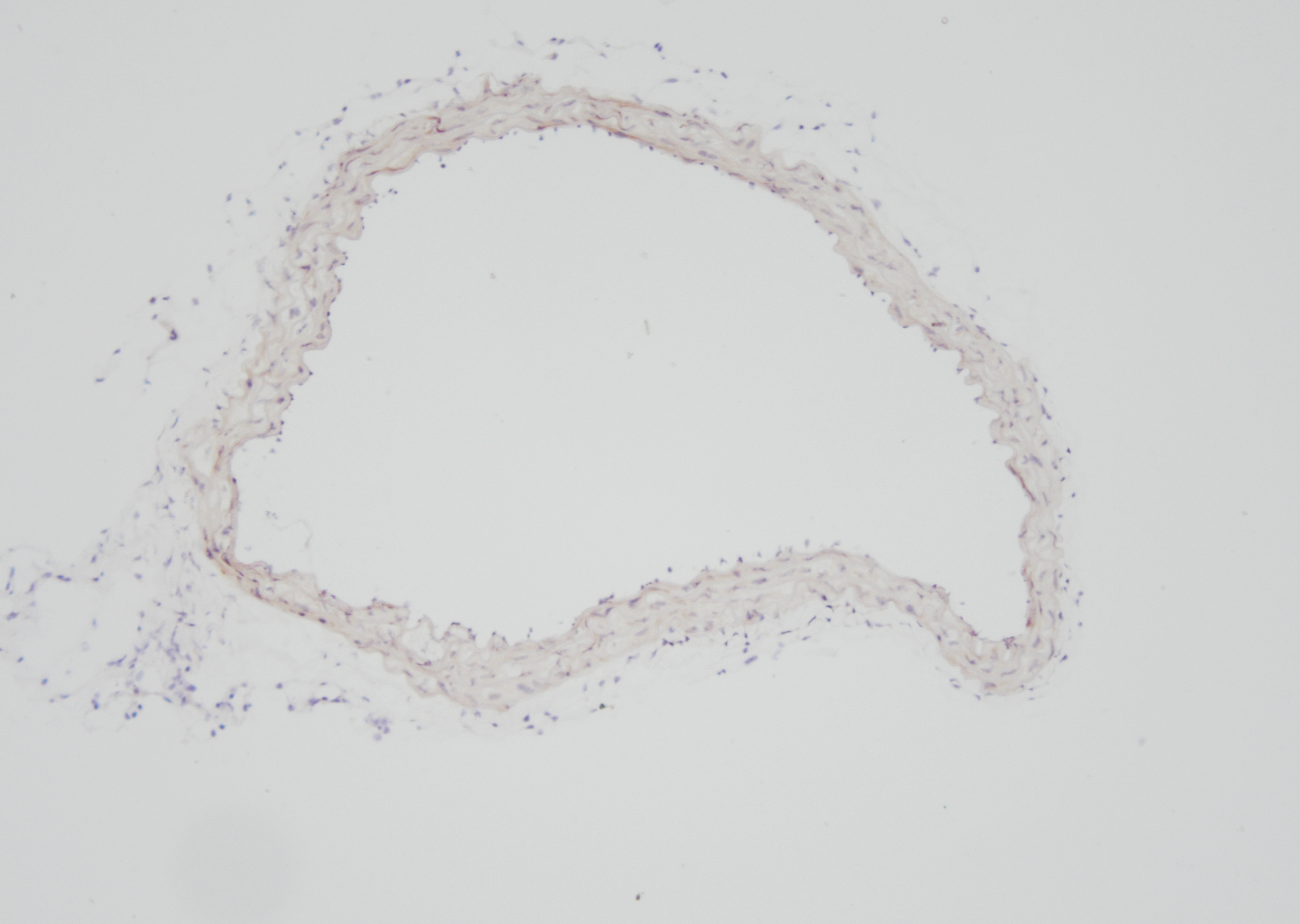

Supplement: Supplementary file 3 — Source data Fig. 2 [file 44321_2025_318_MOESM3_ESM.zip › Figure 2/Figure 2E/Cleaved-caspase3 AngII +CL316,243.tif]

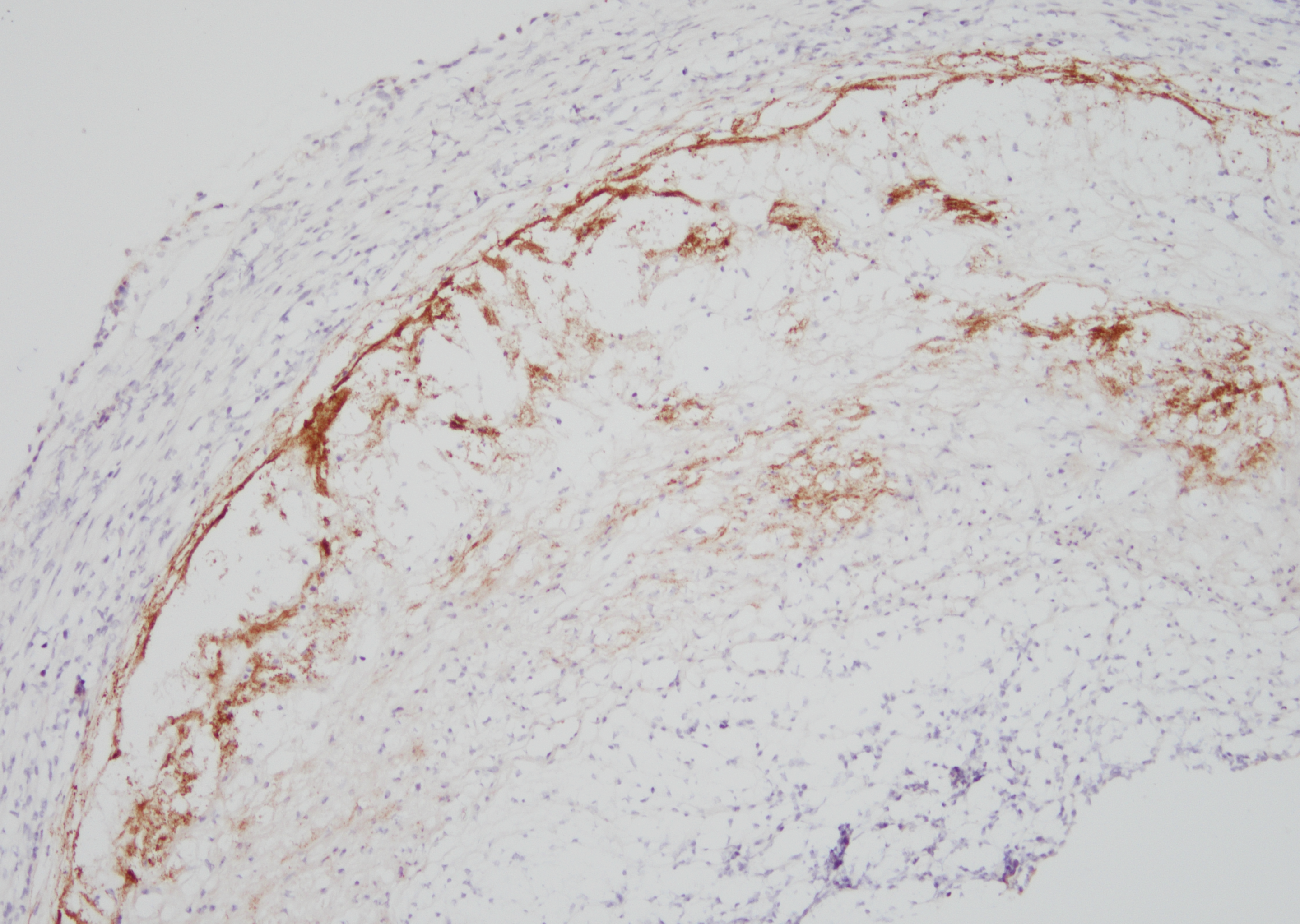

Supplement: Supplementary file 3 — Source data Fig. 2 [file 44321_2025_318_MOESM3_ESM.zip › Figure 2/Figure 2E/Cleaved-caspase3 AngII +Saline.tif]

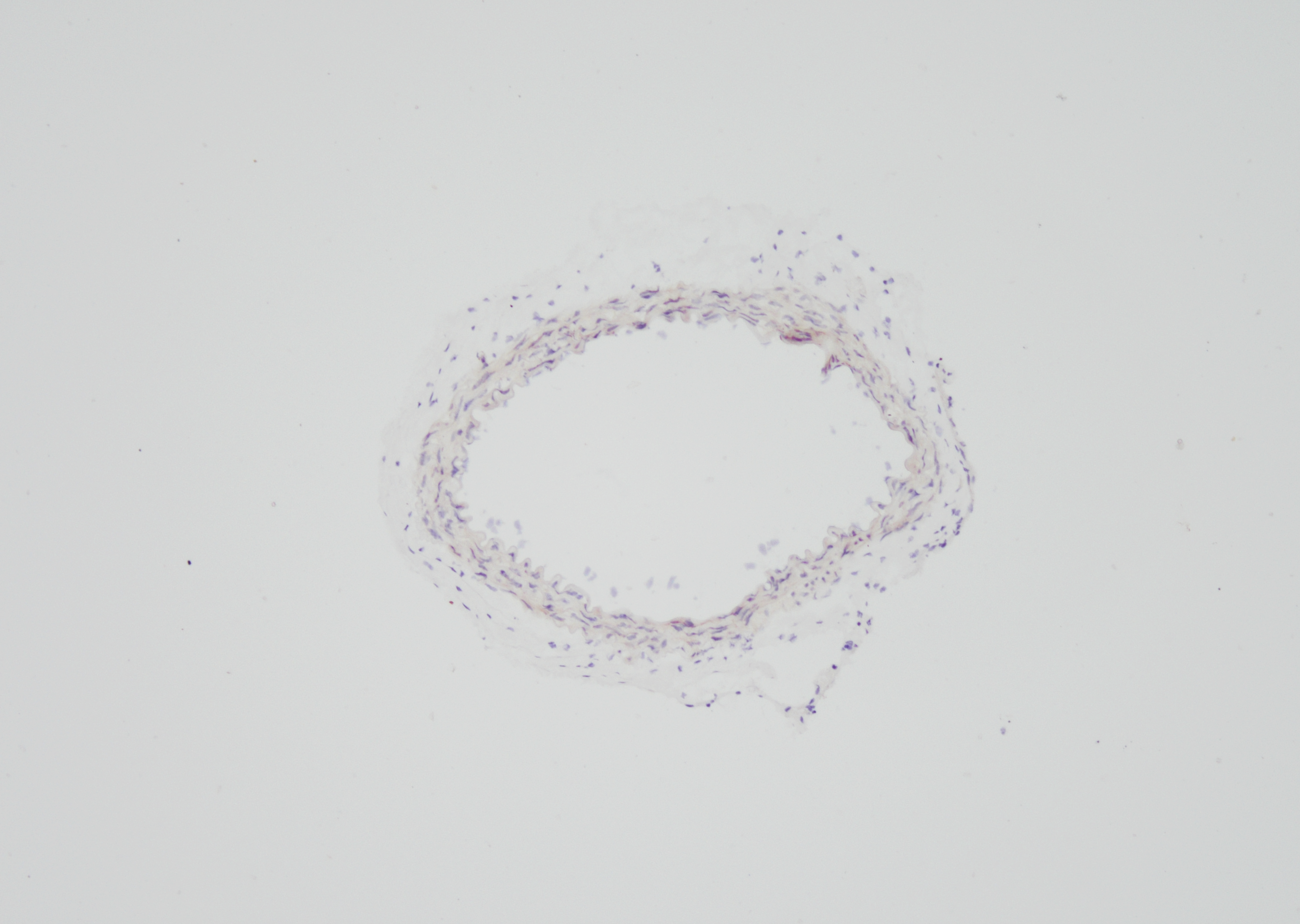

Supplement: Supplementary file 3 — Source data Fig. 2 [file 44321_2025_318_MOESM3_ESM.zip › Figure 2/Figure 2E/Cleaved-Caspase3 Control.tif]

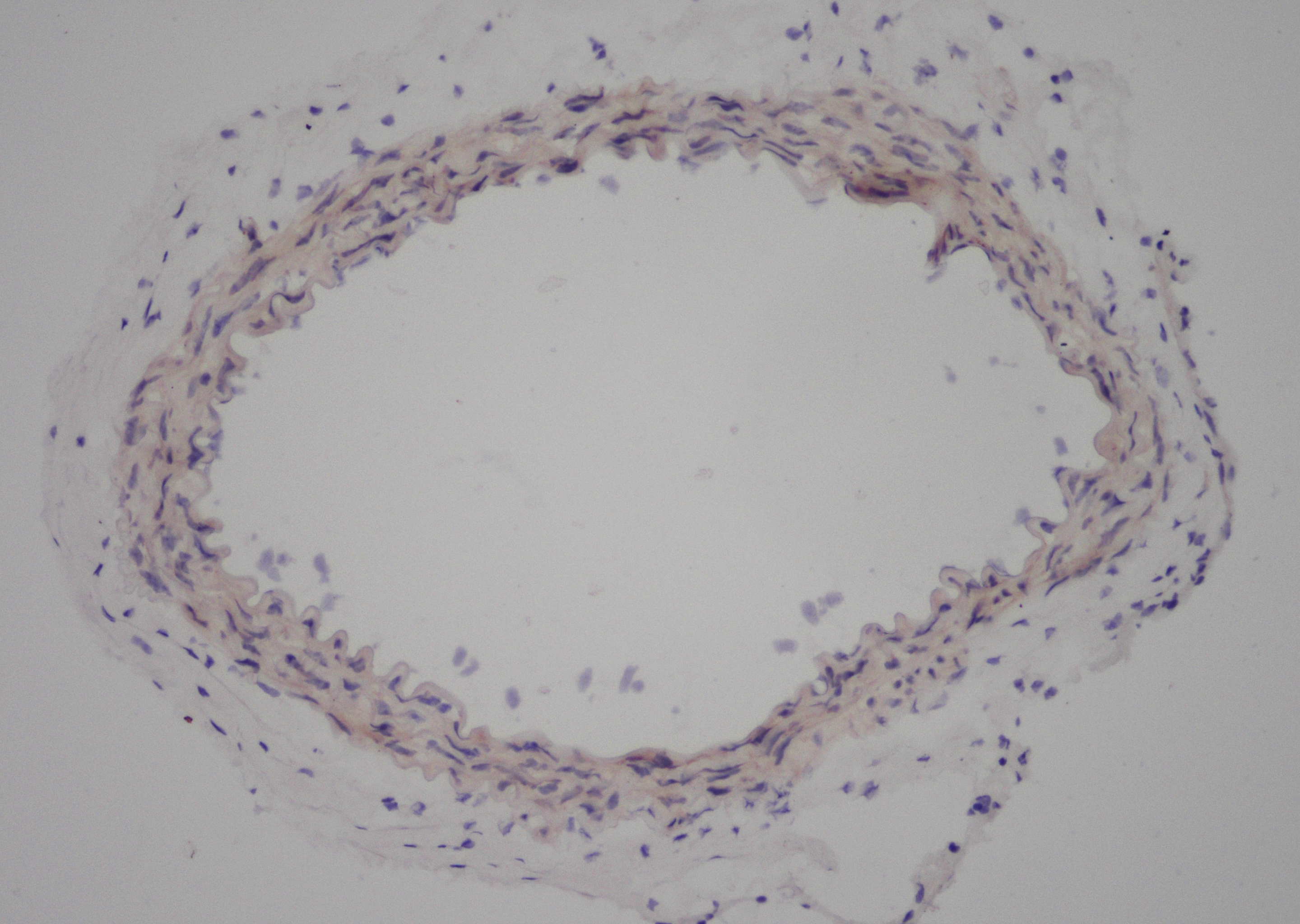

Supplement: Supplementary file 3 — Source data Fig. 2 [file 44321_2025_318_MOESM3_ESM.zip › Figure 2/Figure 2E/Control.tif]

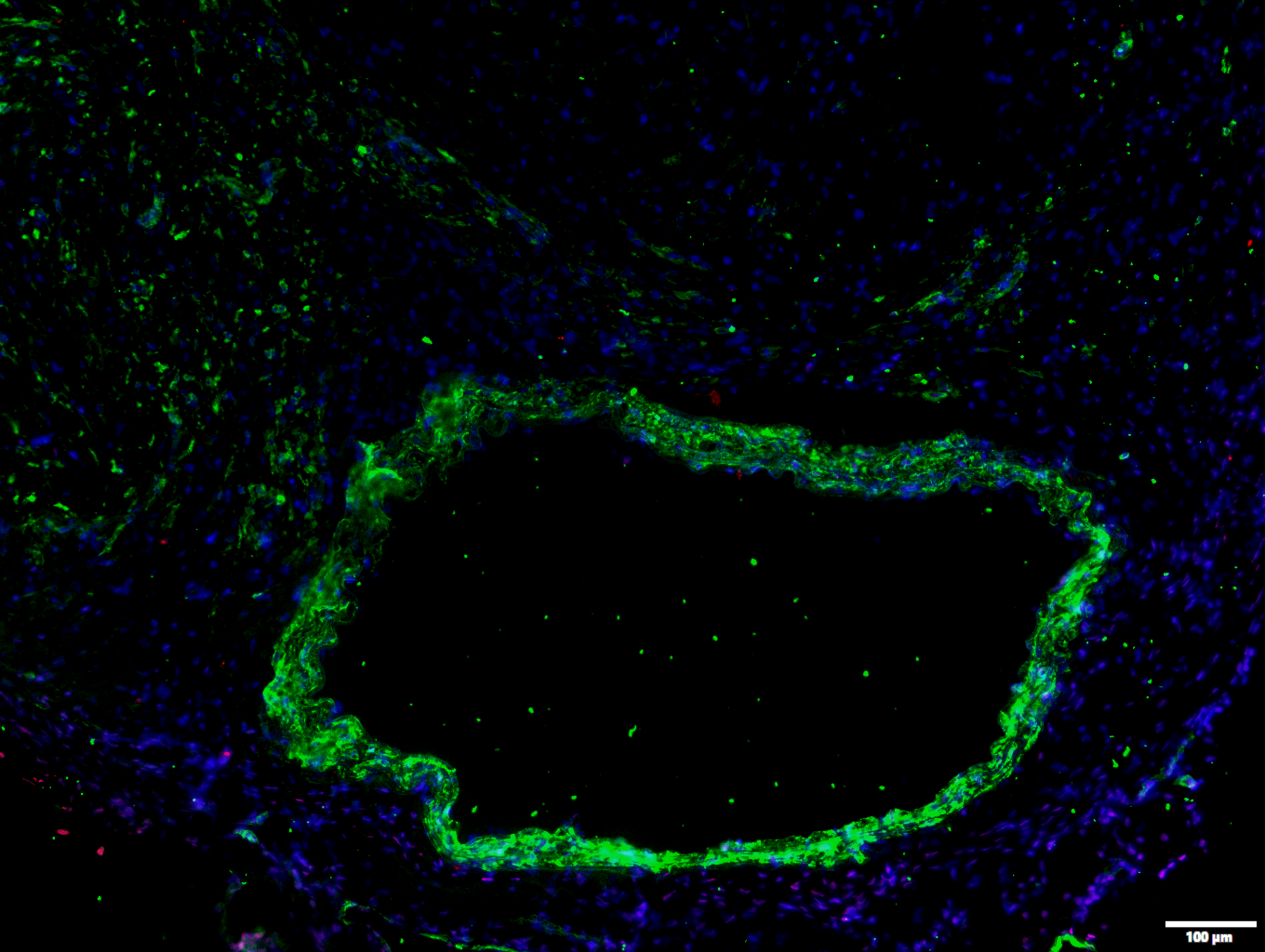

Supplement: Supplementary file 3 — Source data Fig. 2 [file 44321_2025_318_MOESM3_ESM.zip › Figure 2/Figure 2G/AngII + CL316,243.tif]

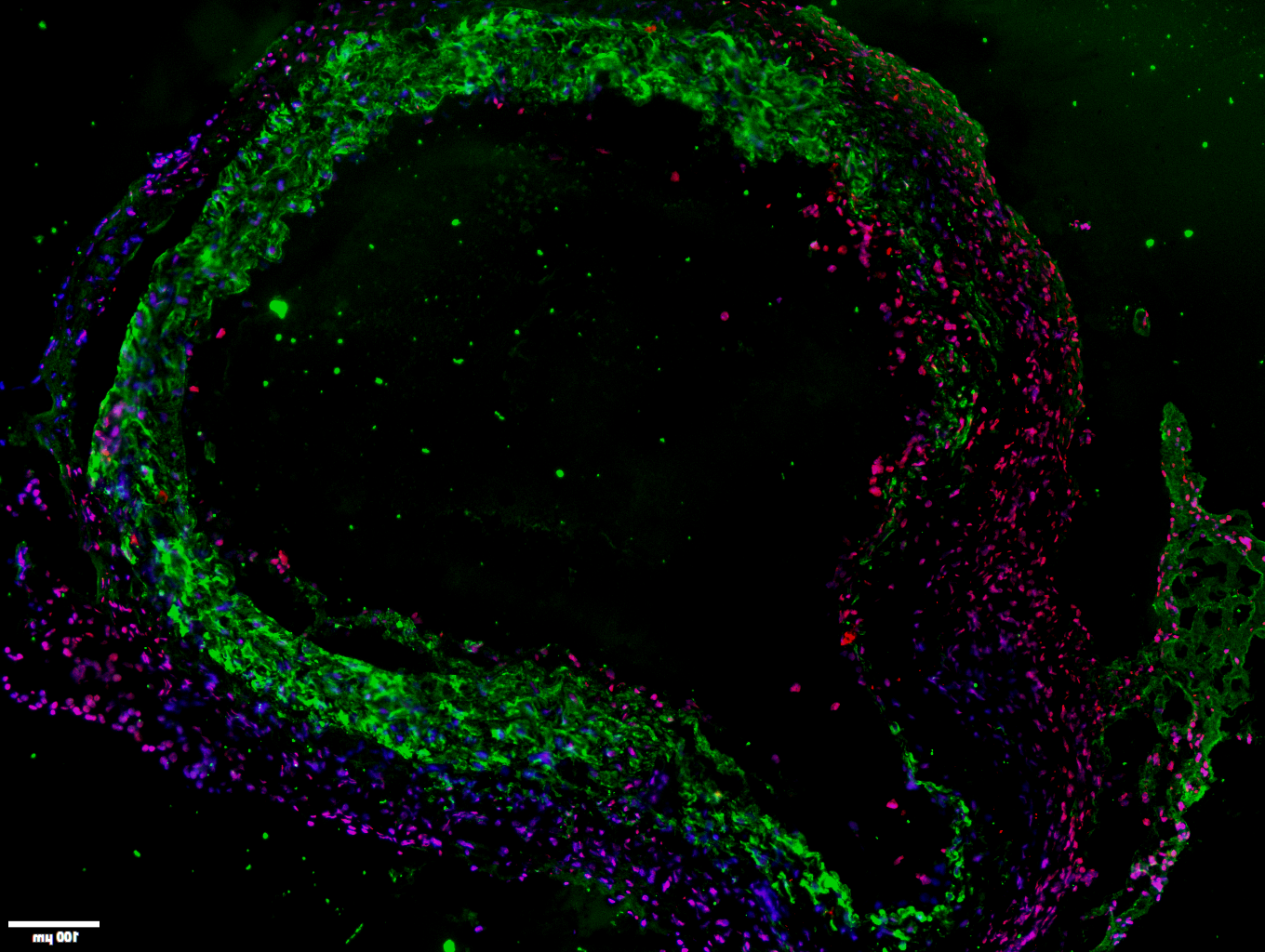

Supplement: Supplementary file 3 — Source data Fig. 2 [file 44321_2025_318_MOESM3_ESM.zip › Figure 2/Figure 2G/AngII + Saline.tif]

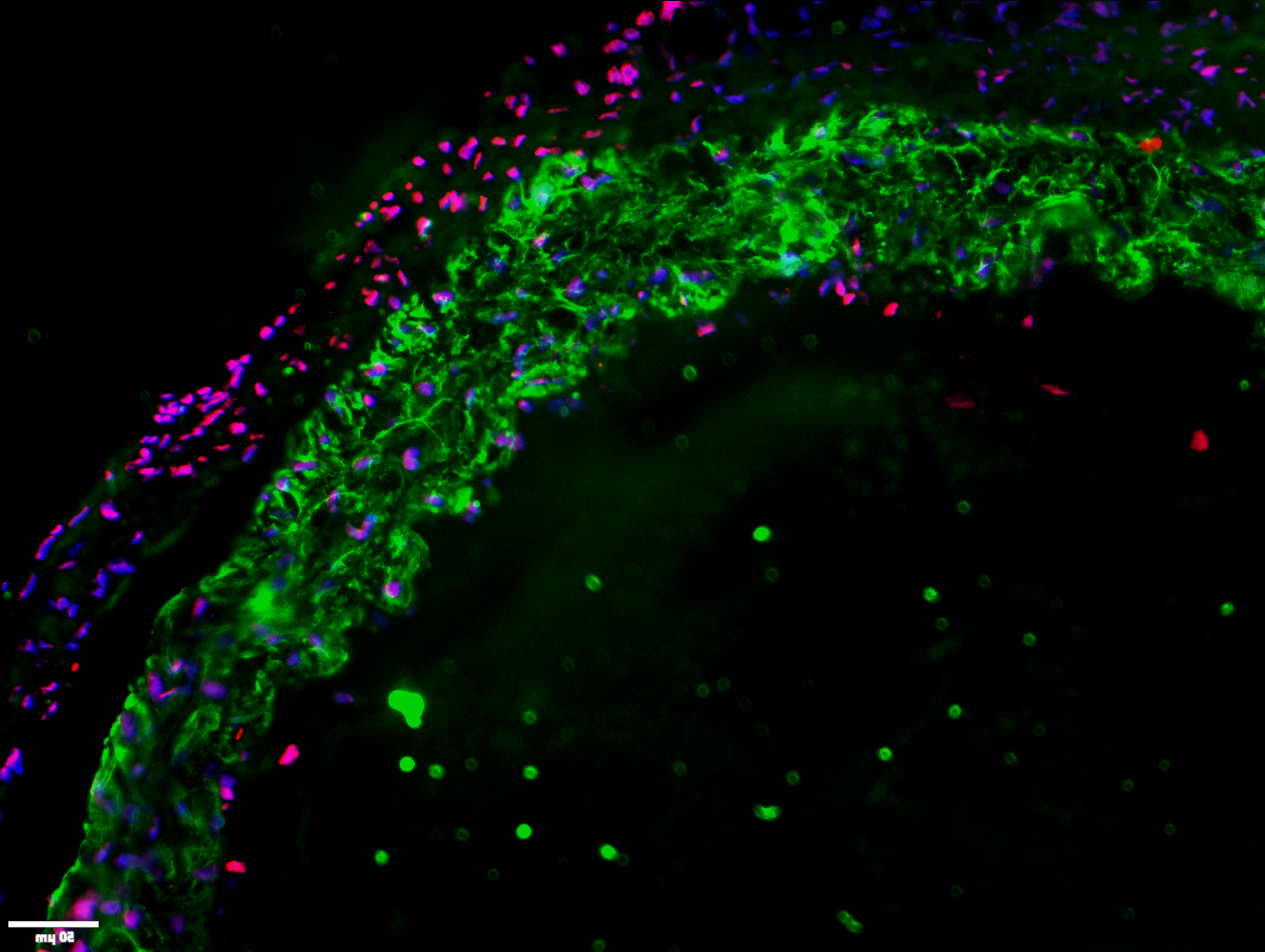

Supplement: Supplementary file 3 — Source data Fig. 2 [file 44321_2025_318_MOESM3_ESM.zip › Figure 2/Figure 2G/AngII + Saline 50um.tif]

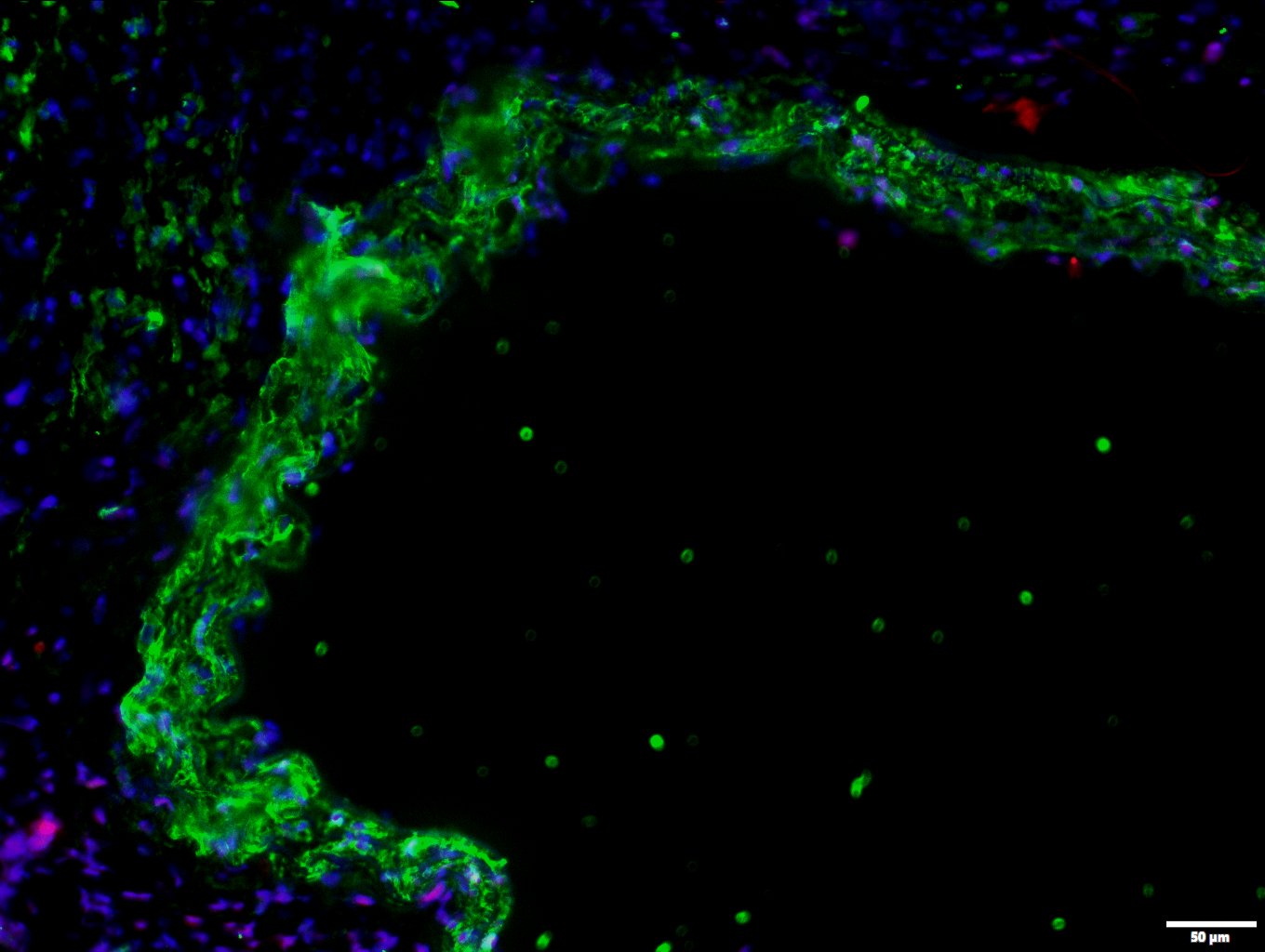

Supplement: Supplementary file 3 — Source data Fig. 2 [file 44321_2025_318_MOESM3_ESM.zip › Figure 2/Figure 2G/AngII+CL316,243 50um.tif]

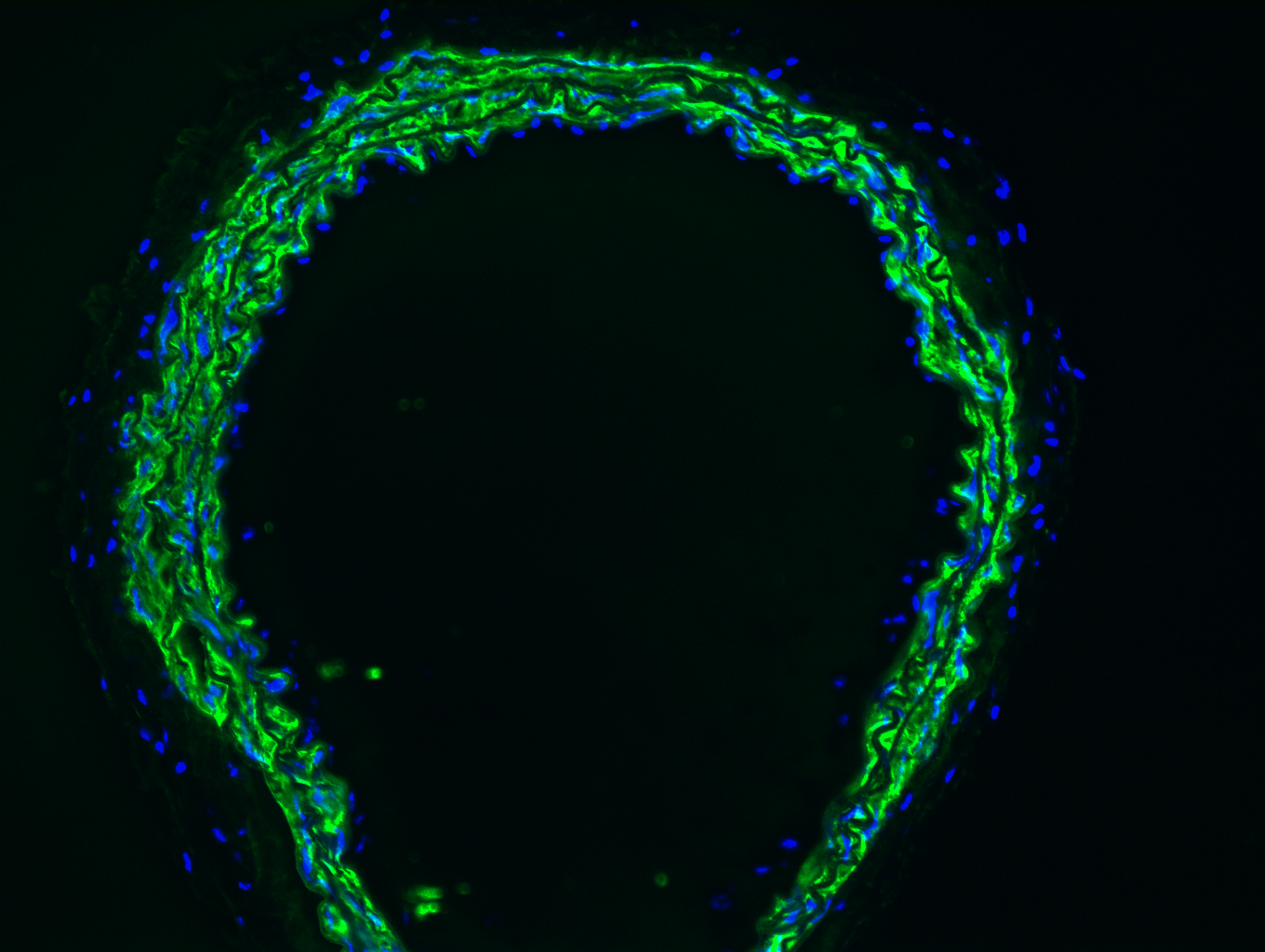

Supplement: Supplementary file 3 — Source data Fig. 2 [file 44321_2025_318_MOESM3_ESM.zip › Figure 2/Figure 2G/Control 50um.tif]

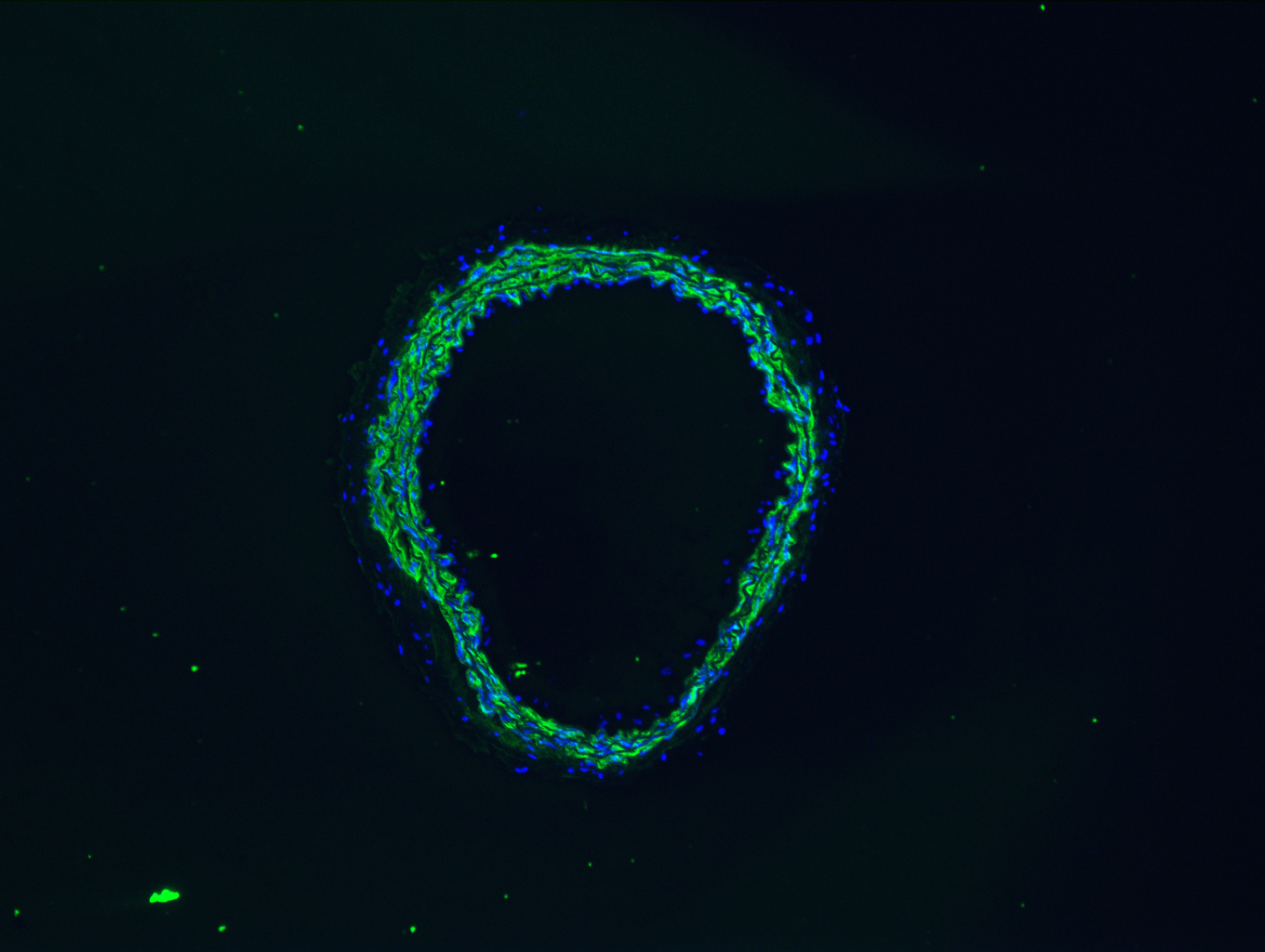

Supplement: Supplementary file 3 — Source data Fig. 2 [file 44321_2025_318_MOESM3_ESM.zip › Figure 2/Figure 2G/Control.tif]

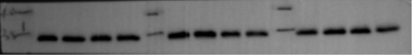

Supplement: Supplementary file 4 — Source data Fig. 3 [file 44321_2025_318_MOESM4_ESM.zip › Figure 3/Figure 3F/Caspase3.tif]

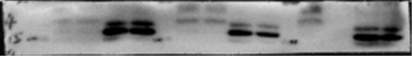

Supplement: Supplementary file 4 — Source data Fig. 3 [file 44321_2025_318_MOESM4_ESM.zip › Figure 3/Figure 3F/Cleaved-Caspase3.tif]

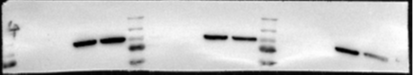

Supplement: Supplementary file 4 — Source data Fig. 3 [file 44321_2025_318_MOESM4_ESM.zip › Figure 3/Figure 3F/Cleaved-PARP.tif]

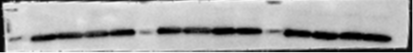

Supplement: Supplementary file 4 — Source data Fig. 3 [file 44321_2025_318_MOESM4_ESM.zip › Figure 3/Figure 3F/GAPDH.tif]

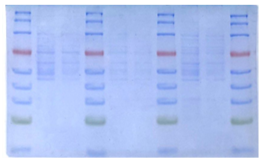

Supplement: Supplementary file 5 — Source data Fig. 4 [file 44321_2025_318_MOESM5_ESM.zip › Figure 4/Figure 4E/CBB Staining.tif]

## Slide 1
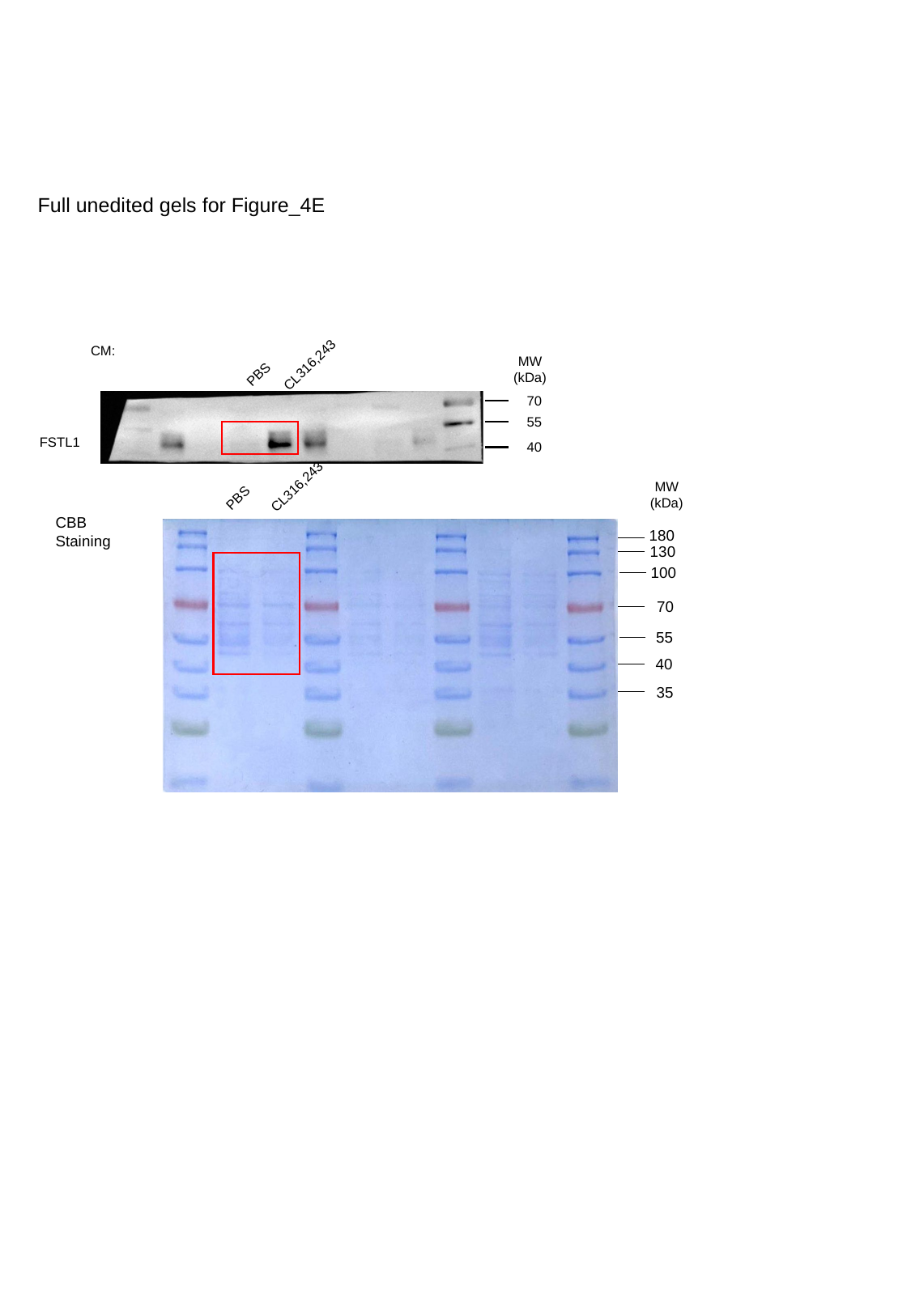

Full unedited gels for Figure_4E
CM:
MW
(kDa)
CL316,243
PBS
70
55
FSTL1
40
MW
(kDa)
CL316,243
PBS
CBB
Staining
180
130
100
70
55
40
35

Supplement: Supplementary file 5 — Source data Fig. 4 [file 44321_2025_318_MOESM5_ESM.zip › Figure 4/Figure 4E/README.pptx]

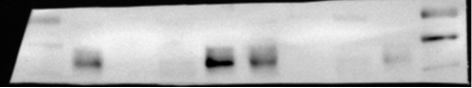

Supplement: Supplementary file 5 — Source data Fig. 4 [file 44321_2025_318_MOESM5_ESM.zip › Figure 4/Figure 4E/Western FSTL1.tif]

## Slide 1
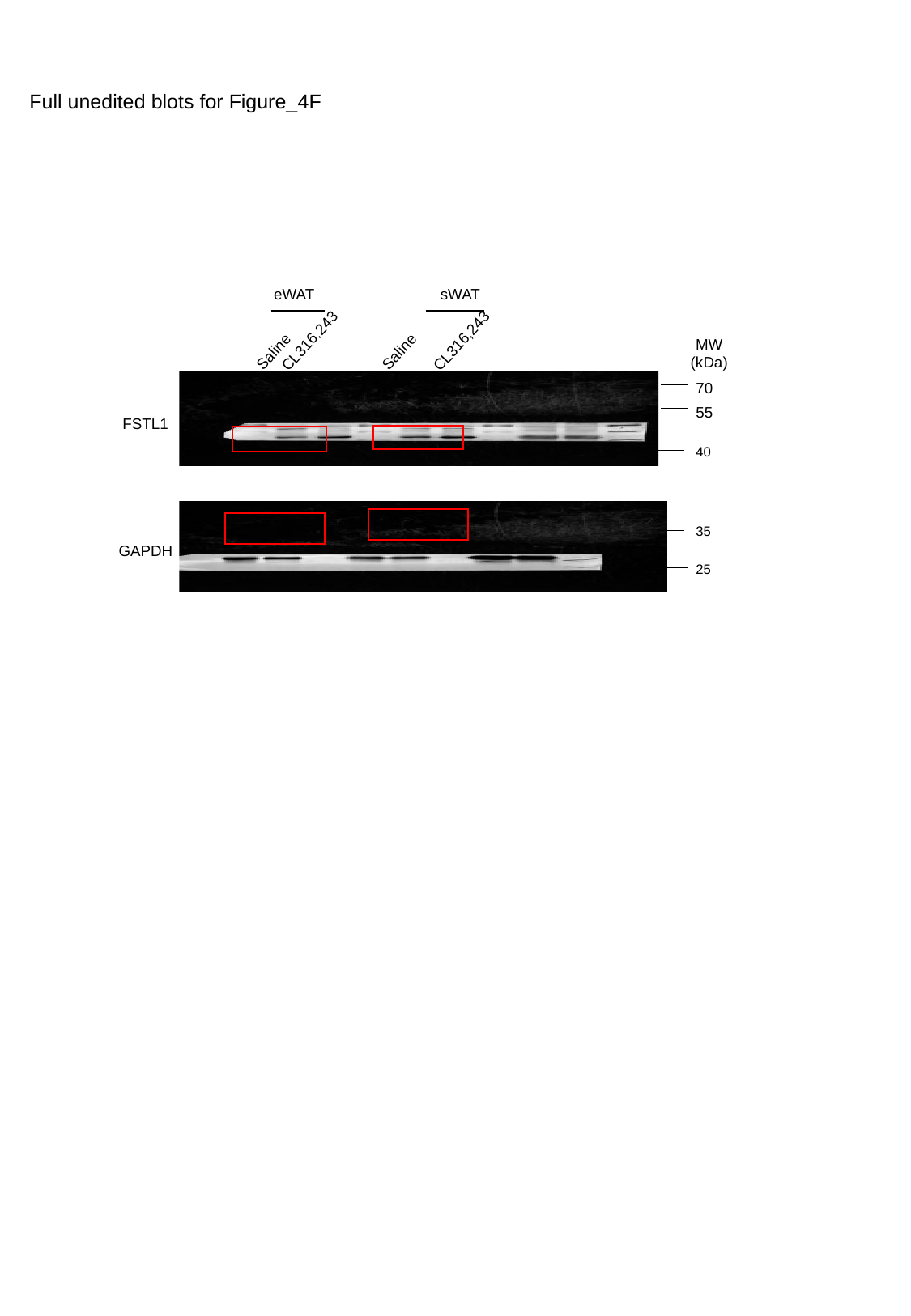

Full unedited blots for Figure_4F
eWAT
sWAT
CL316,243
CL316,243
MW
(kDa)
Saline
Saline
70
55
FSTL1
40
35
GAPDH
25

Supplement: Supplementary file 5 — Source data Fig. 4 [file 44321_2025_318_MOESM5_ESM.zip › Figure 4/Figure 4F/README.pptx]

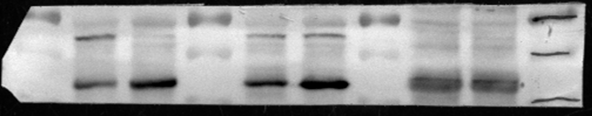

Supplement: Supplementary file 5 — Source data Fig. 4 [file 44321_2025_318_MOESM5_ESM.zip › Figure 4/Figure 4F/Western blot FSTL1.tif]

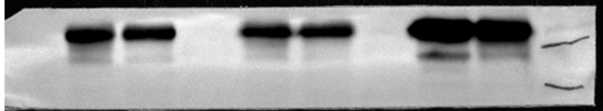

Supplement: Supplementary file 5 — Source data Fig. 4 [file 44321_2025_318_MOESM5_ESM.zip › Figure 4/Figure 4F/Western blot GAPDH.tif]

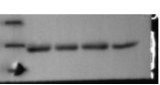

Supplement: Supplementary file 5 — Source data Fig. 4 [file 44321_2025_318_MOESM5_ESM.zip › Figure 4/Figure 4J/Western Caspase3.tif]

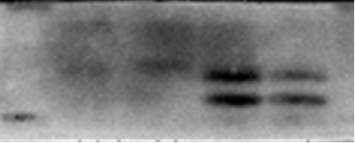

Supplement: Supplementary file 5 — Source data Fig. 4 [file 44321_2025_318_MOESM5_ESM.zip › Figure 4/Figure 4J/Western Cleaved-Caspase3.tif]

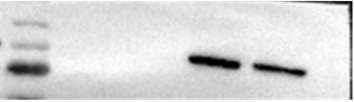

Supplement: Supplementary file 5 — Source data Fig. 4 [file 44321_2025_318_MOESM5_ESM.zip › Figure 4/Figure 4J/Western Cleaved-PARP.tif]

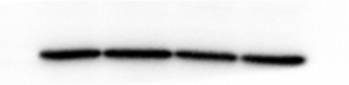

Supplement: Supplementary file 5 — Source data Fig. 4 [file 44321_2025_318_MOESM5_ESM.zip › Figure 4/Figure 4J/Western GAPDH.tif]

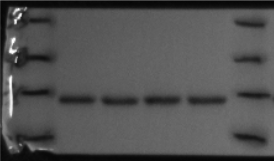

Supplement: Supplementary file 5 — Source data Fig. 4 [file 44321_2025_318_MOESM5_ESM.zip › Figure 4/Figure 4K/Western caspase3.tif]

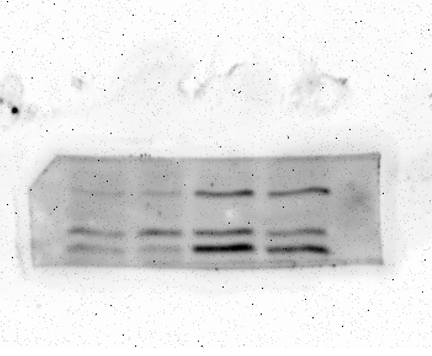

Supplement: Supplementary file 5 — Source data Fig. 4 [file 44321_2025_318_MOESM5_ESM.zip › Figure 4/Figure 4K/Western Cleaved-caspase3.tif]

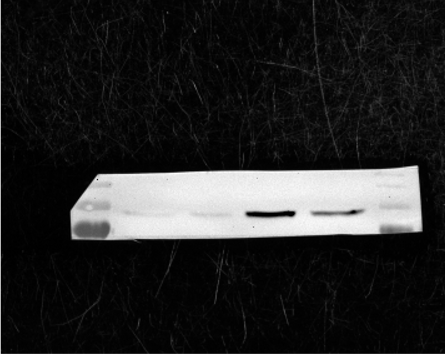

Supplement: Supplementary file 5 — Source data Fig. 4 [file 44321_2025_318_MOESM5_ESM.zip › Figure 4/Figure 4K/Western Cleaved-PARP.tif]

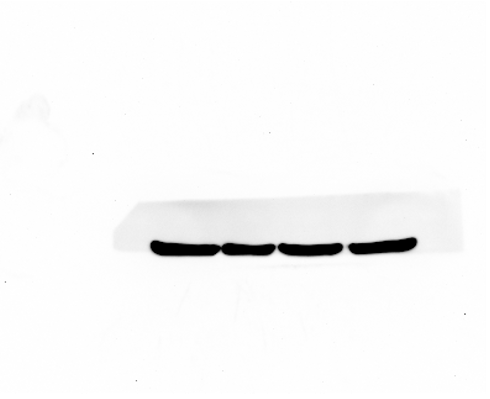

Supplement: Supplementary file 5 — Source data Fig. 4 [file 44321_2025_318_MOESM5_ESM.zip › Figure 4/Figure 4K/Western GAPDH.tif]

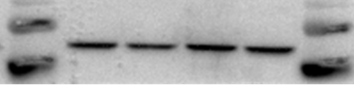

Supplement: Supplementary file 6 — Source data Fig. 5 [file 44321_2025_318_MOESM6_ESM.zip › Figure 5/Figure 5I/Western AKT.tif]

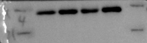

Supplement: Supplementary file 6 — Source data Fig. 5 [file 44321_2025_318_MOESM6_ESM.zip › Figure 5/Figure 5I/Western Caspase3.tif]

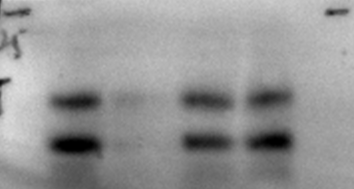

Supplement: Supplementary file 6 — Source data Fig. 5 [file 44321_2025_318_MOESM6_ESM.zip › Figure 5/Figure 5I/Western Cleaved-caspase3.tif]

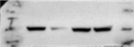

Supplement: Supplementary file 6 — Source data Fig. 5 [file 44321_2025_318_MOESM6_ESM.zip › Figure 5/Figure 5I/Western Cleaved-PARP.tif]

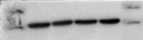

Supplement: Supplementary file 6 — Source data Fig. 5 [file 44321_2025_318_MOESM6_ESM.zip › Figure 5/Figure 5I/Western GAPDH.tif]

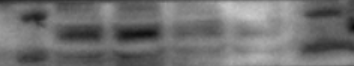

Supplement: Supplementary file 6 — Source data Fig. 5 [file 44321_2025_318_MOESM6_ESM.zip › Figure 5/Figure 5I/Western pAKT.tif]

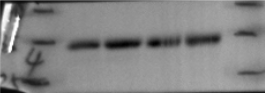

Supplement: Supplementary file 6 — Source data Fig. 5 [file 44321_2025_318_MOESM6_ESM.zip › Figure 5/Figure 5C/Western Caspase3.tif]

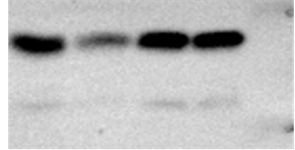

Supplement: Supplementary file 6 — Source data Fig. 5 [file 44321_2025_318_MOESM6_ESM.zip › Figure 5/Figure 5C/Western Cleaved-Caspase3.tif]

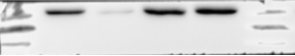

Supplement: Supplementary file 6 — Source data Fig. 5 [file 44321_2025_318_MOESM6_ESM.zip › Figure 5/Figure 5C/Western Cleaved-PARP.tif]

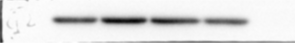

Supplement: Supplementary file 6 — Source data Fig. 5 [file 44321_2025_318_MOESM6_ESM.zip › Figure 5/Figure 5C/Western GAPDH.tif]

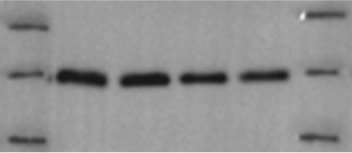

Supplement: Supplementary file 6 — Source data Fig. 5 [file 44321_2025_318_MOESM6_ESM.zip › Figure 5/Figure 5D/Western Caspase3.tif]

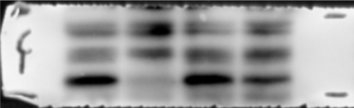

Supplement: Supplementary file 6 — Source data Fig. 5 [file 44321_2025_318_MOESM6_ESM.zip › Figure 5/Figure 5D/Western Cleaved-caspase3.tif]

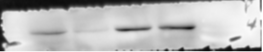

Supplement: Supplementary file 6 — Source data Fig. 5 [file 44321_2025_318_MOESM6_ESM.zip › Figure 5/Figure 5D/Western Cleaved-PARP.tif]

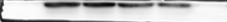

Supplement: Supplementary file 6 — Source data Fig. 5 [file 44321_2025_318_MOESM6_ESM.zip › Figure 5/Figure 5D/Western GAPDH.tif]

## Slide 1
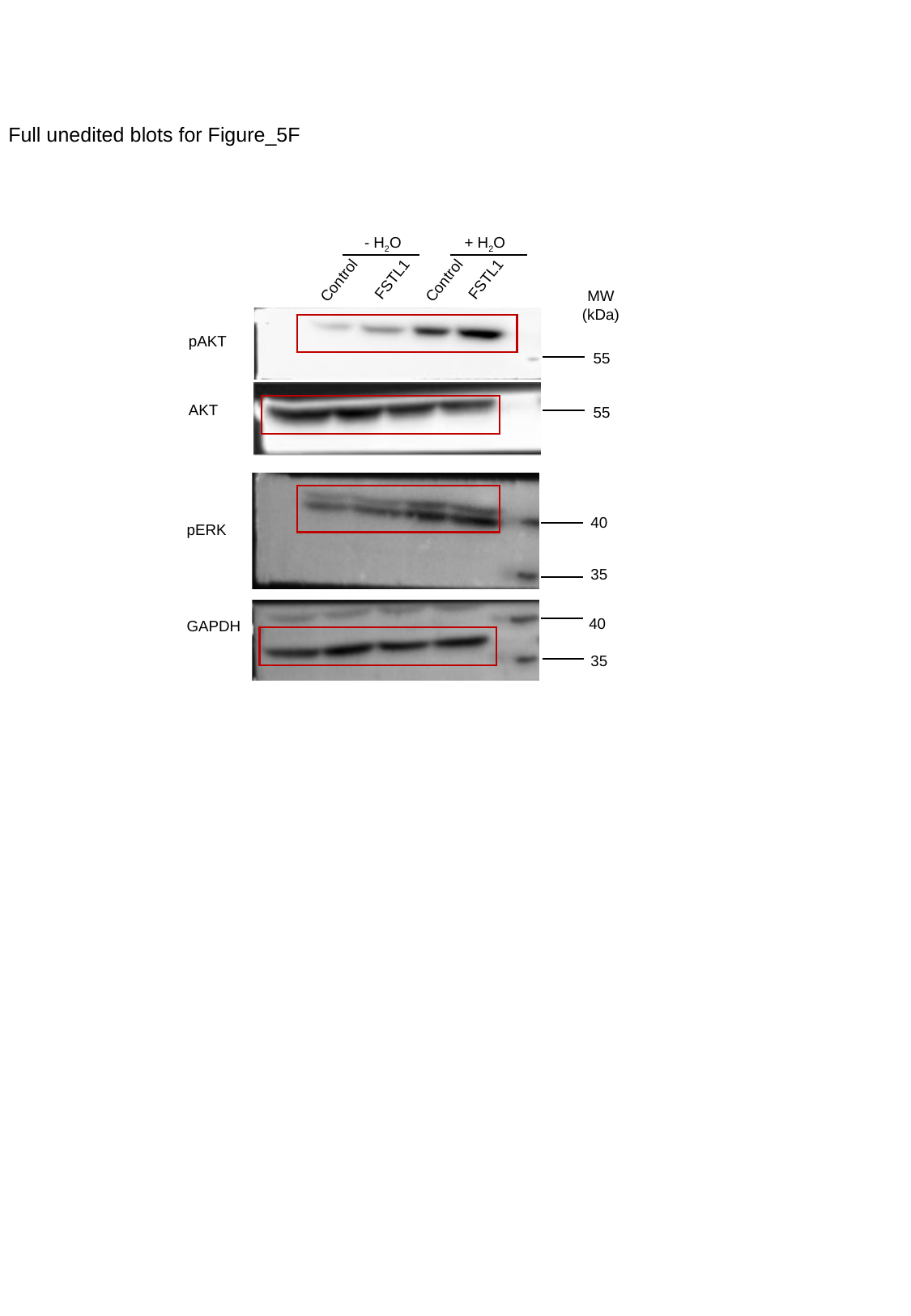

Full unedited blots for Figure_5F
- H2O
+ H2O
FSTL1
FSTL1
Control
Control
MW
(kDa)
pAKT
55
AKT
55
40
pERK
35
40
GAPDH
35

Supplement: Supplementary file 6 — Source data Fig. 5 [file 44321_2025_318_MOESM6_ESM.zip › Figure 5/Figure 5F/README.pptx]

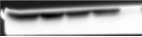

Supplement: Supplementary file 6 — Source data Fig. 5 [file 44321_2025_318_MOESM6_ESM.zip › Figure 5/Figure 5F/Western AKT.tif]

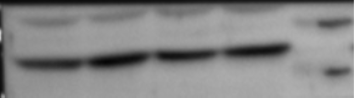

Supplement: Supplementary file 6 — Source data Fig. 5 [file 44321_2025_318_MOESM6_ESM.zip › Figure 5/Figure 5F/Western GAPDH.tif]

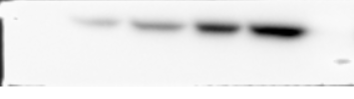

Supplement: Supplementary file 6 — Source data Fig. 5 [file 44321_2025_318_MOESM6_ESM.zip › Figure 5/Figure 5F/Western pAKT.tif]

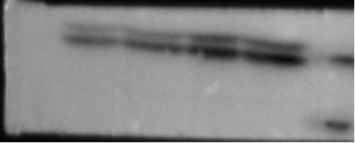

Supplement: Supplementary file 6 — Source data Fig. 5 [file 44321_2025_318_MOESM6_ESM.zip › Figure 5/Figure 5F/Western pERK.tif]

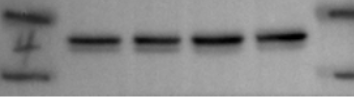

Supplement: Supplementary file 6 — Source data Fig. 5 [file 44321_2025_318_MOESM6_ESM.zip › Figure 5/Figure 5G/Western AKT.tif]

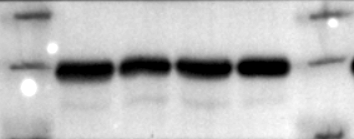

Supplement: Supplementary file 6 — Source data Fig. 5 [file 44321_2025_318_MOESM6_ESM.zip › Figure 5/Figure 5G/Western GAPDH.tif]

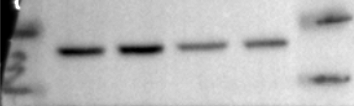

Supplement: Supplementary file 6 — Source data Fig. 5 [file 44321_2025_318_MOESM6_ESM.zip › Figure 5/Figure 5G/Western pAKT.tif]

## Slide 1
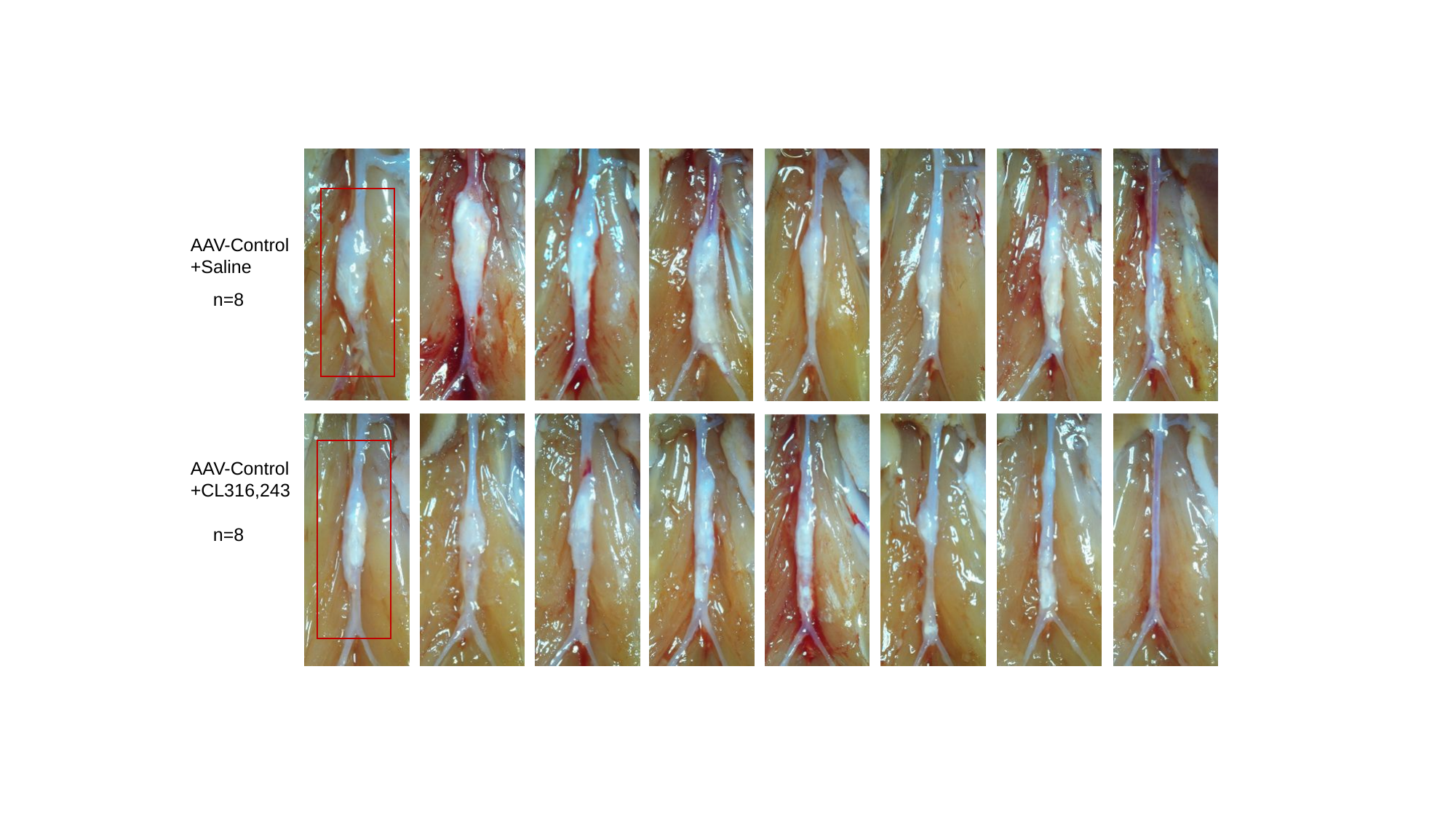

AAV-Control
+Saline
n=8
AAV-Control
+CL316,243
n=8

## Slide 2
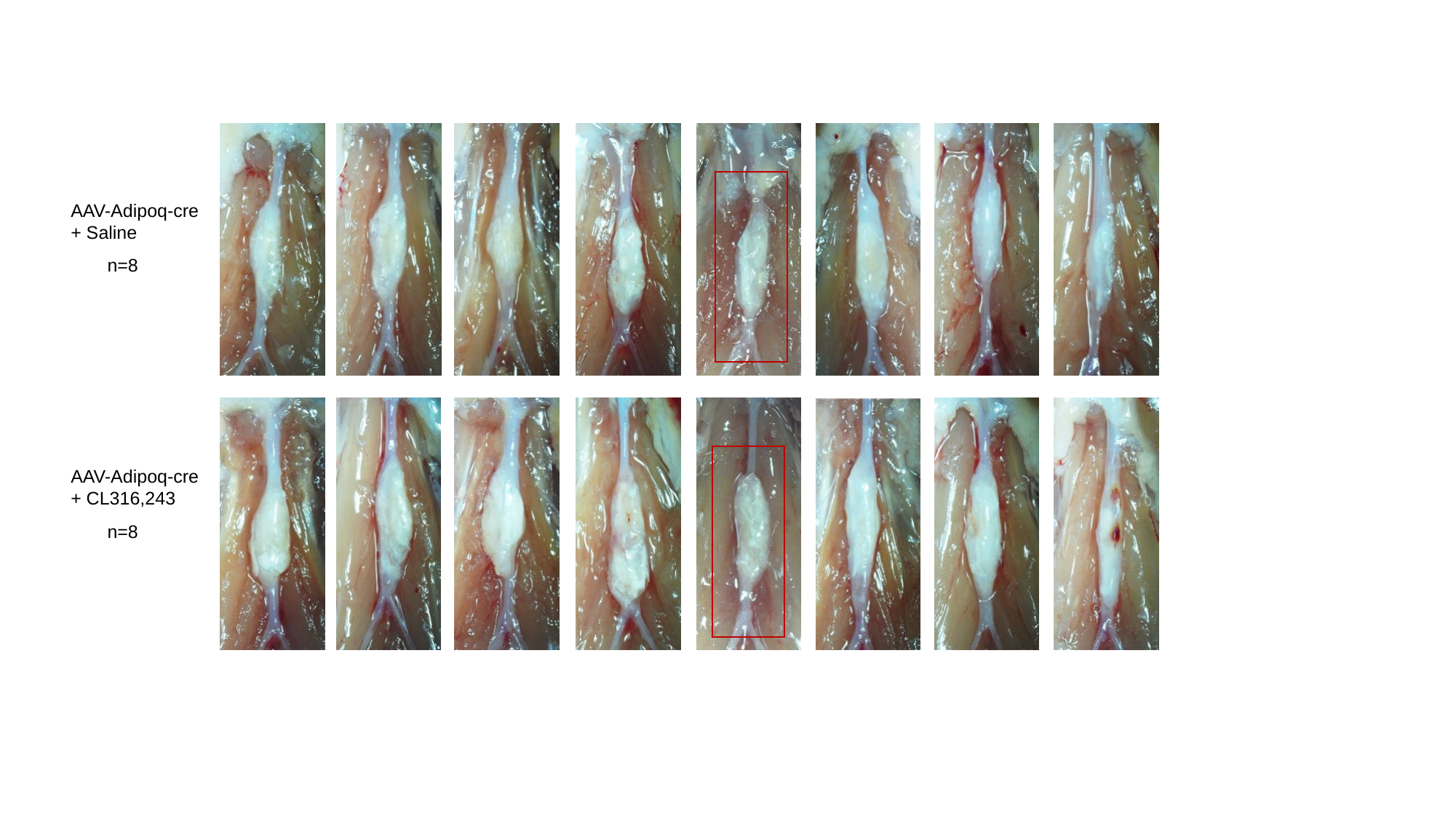

AAV-Adipoq-cre
+ Saline
n=8
AAV-Adipoq-cre
+ CL316,243
n=8

Supplement: Supplementary file 7 — Source data Fig. 6 [file 44321_2025_318_MOESM7_ESM.zip › Figure 6/Figure 6B/Whole mount.pptx]

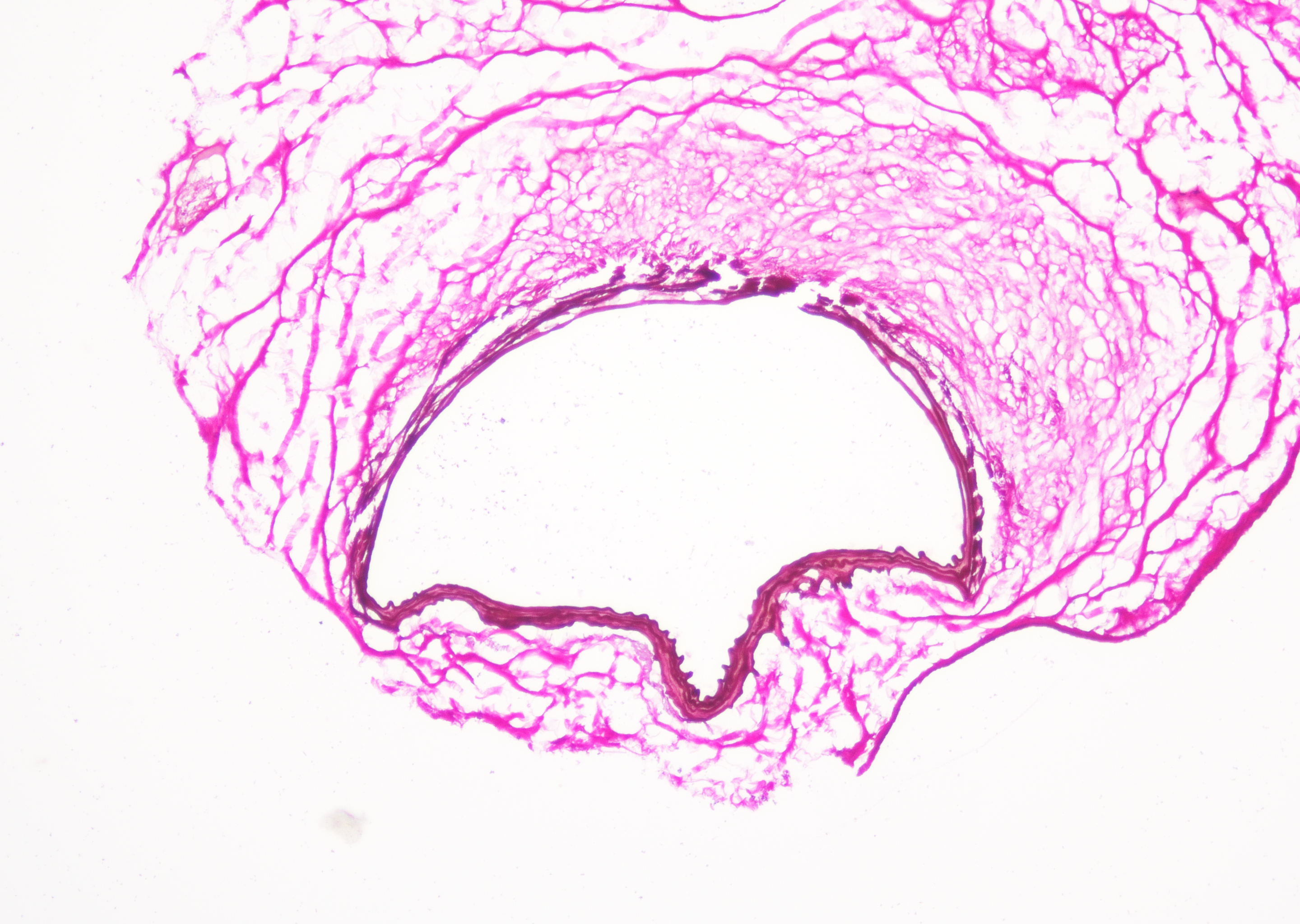

Supplement: Supplementary file 7 — Source data Fig. 6 [file 44321_2025_318_MOESM7_ESM.zip › Figure 6/Figure 6E/EVG Staining/CL316,243 AAV-Adipoq-cre 100um.tif]

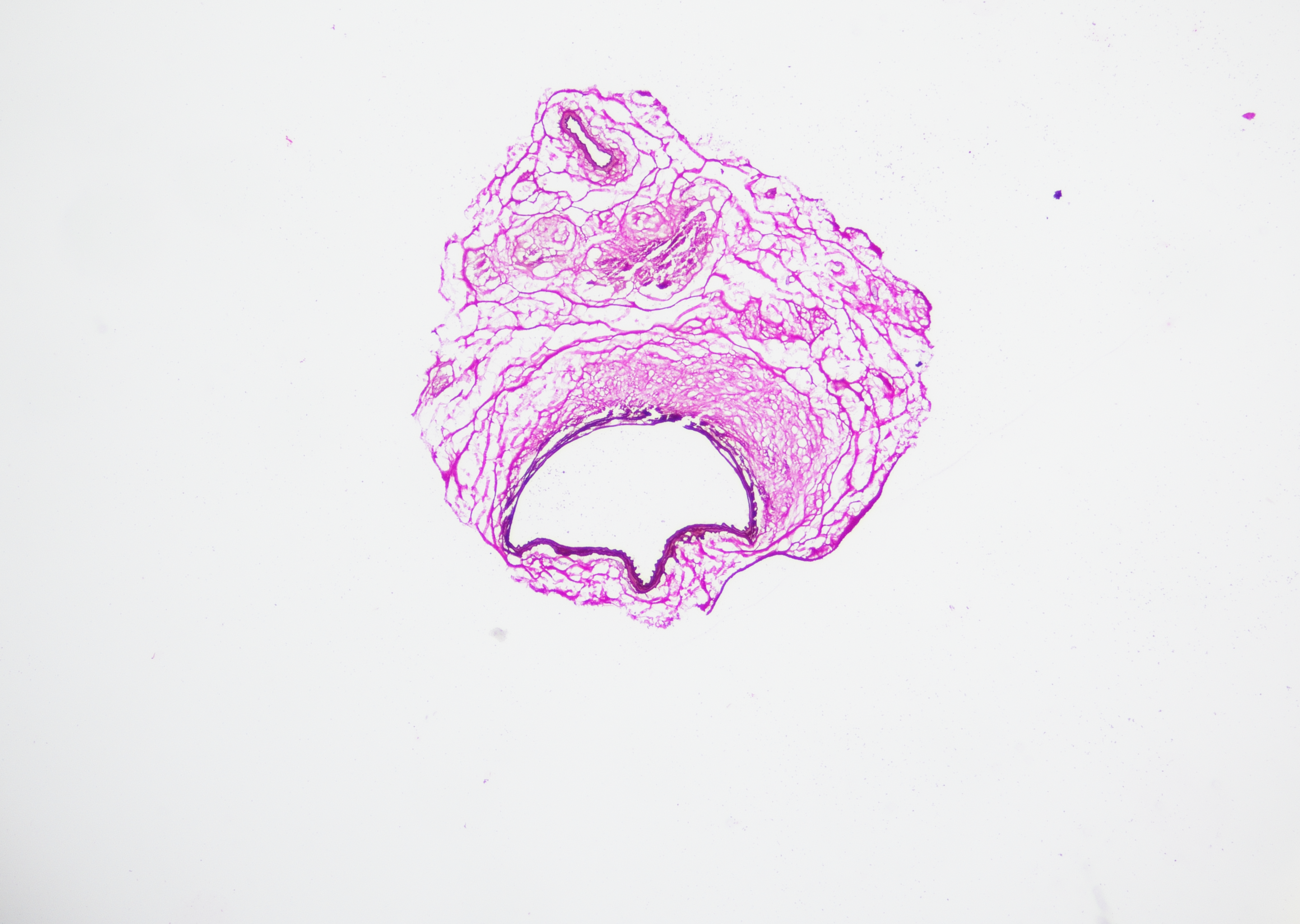

Supplement: Supplementary file 7 — Source data Fig. 6 [file 44321_2025_318_MOESM7_ESM.zip › Figure 6/Figure 6E/EVG Staining/CL316,243 AAV-Adipoq-cre 200um.tif]

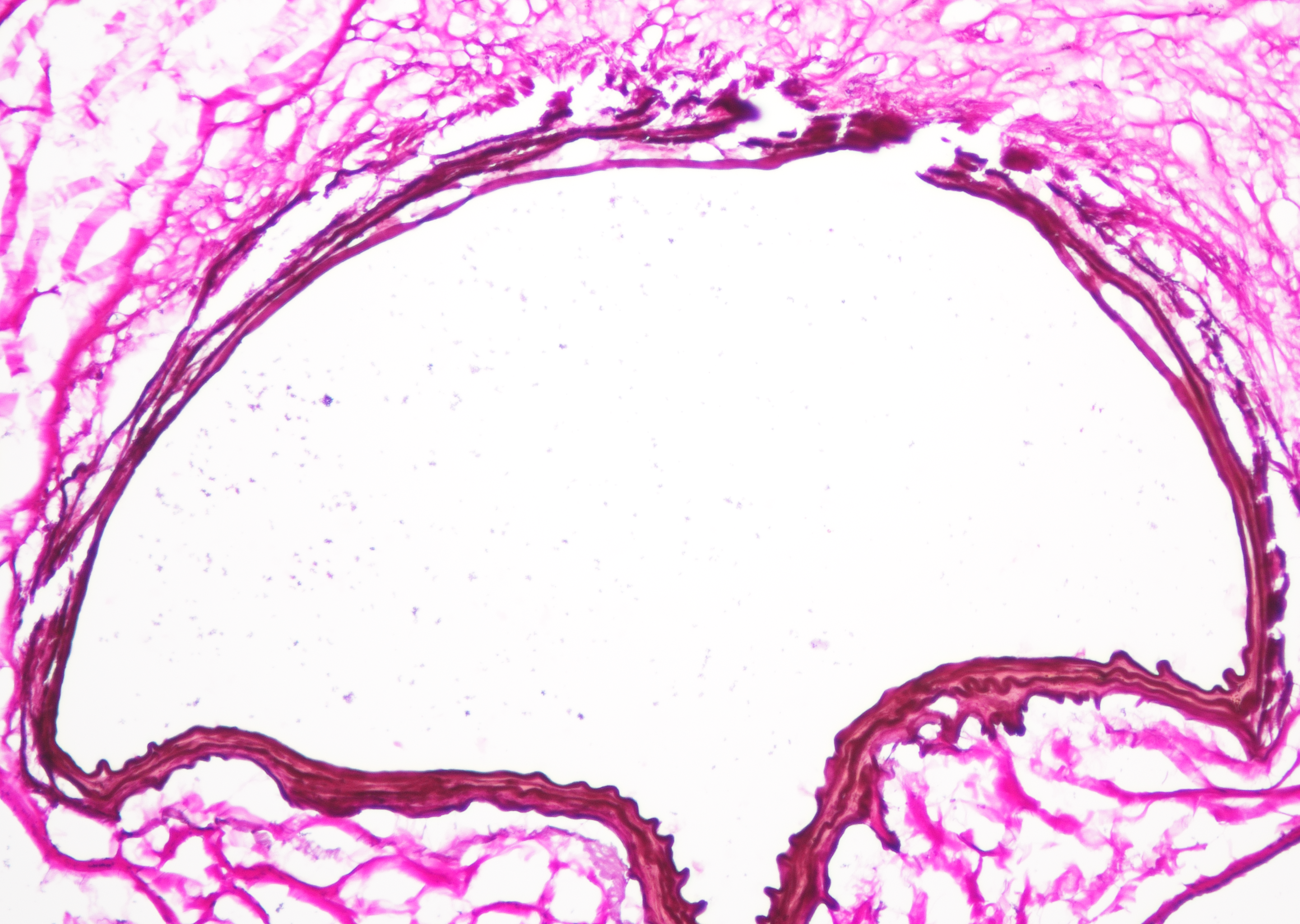

Supplement: Supplementary file 7 — Source data Fig. 6 [file 44321_2025_318_MOESM7_ESM.zip › Figure 6/Figure 6E/EVG Staining/CL316,243 AAV-Adipoq-cre 50um.tif]

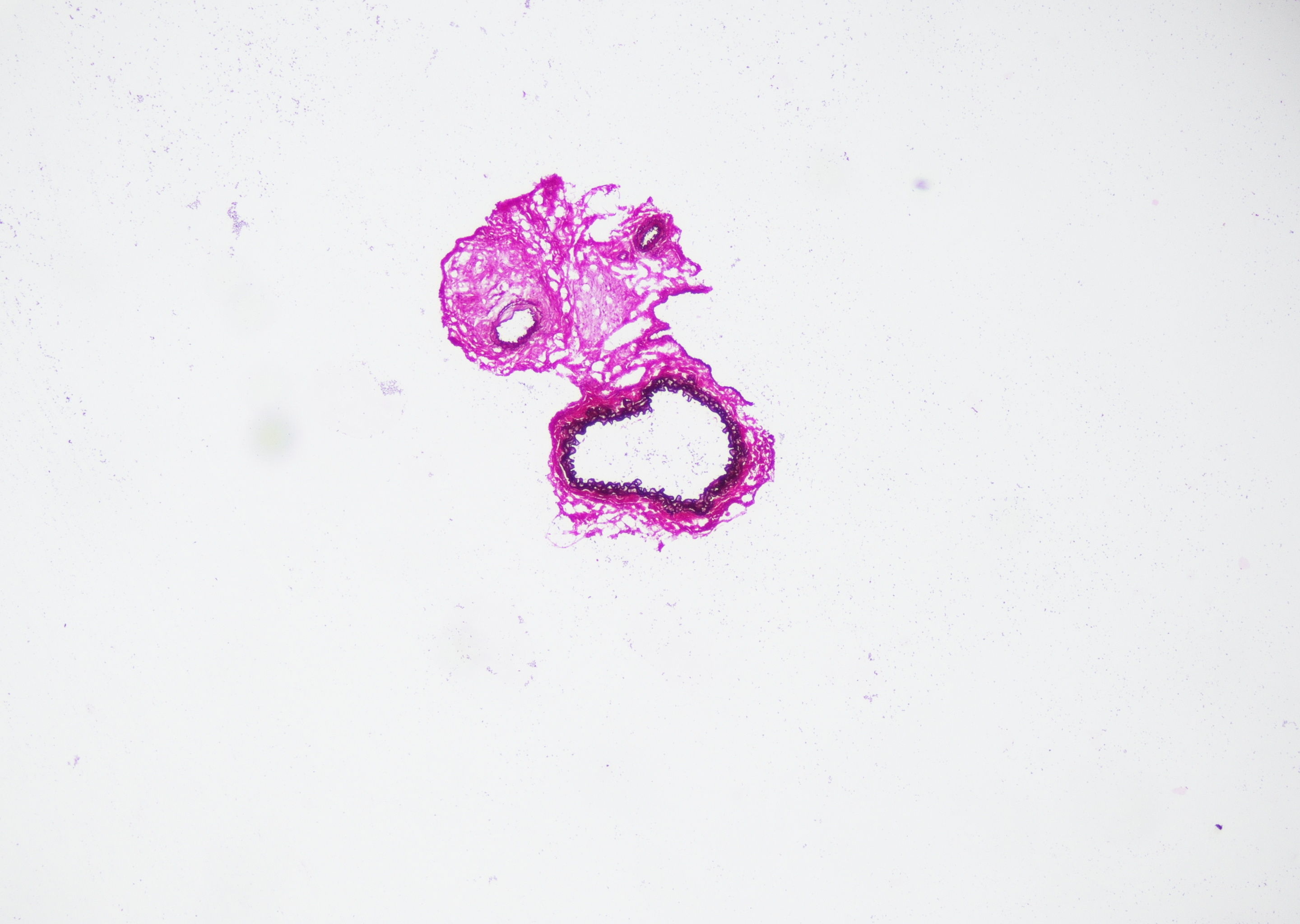

Supplement: Supplementary file 7 — Source data Fig. 6 [file 44321_2025_318_MOESM7_ESM.zip › Figure 6/Figure 6E/EVG Staining/CL316,243 AAV-Control 200um.tif]

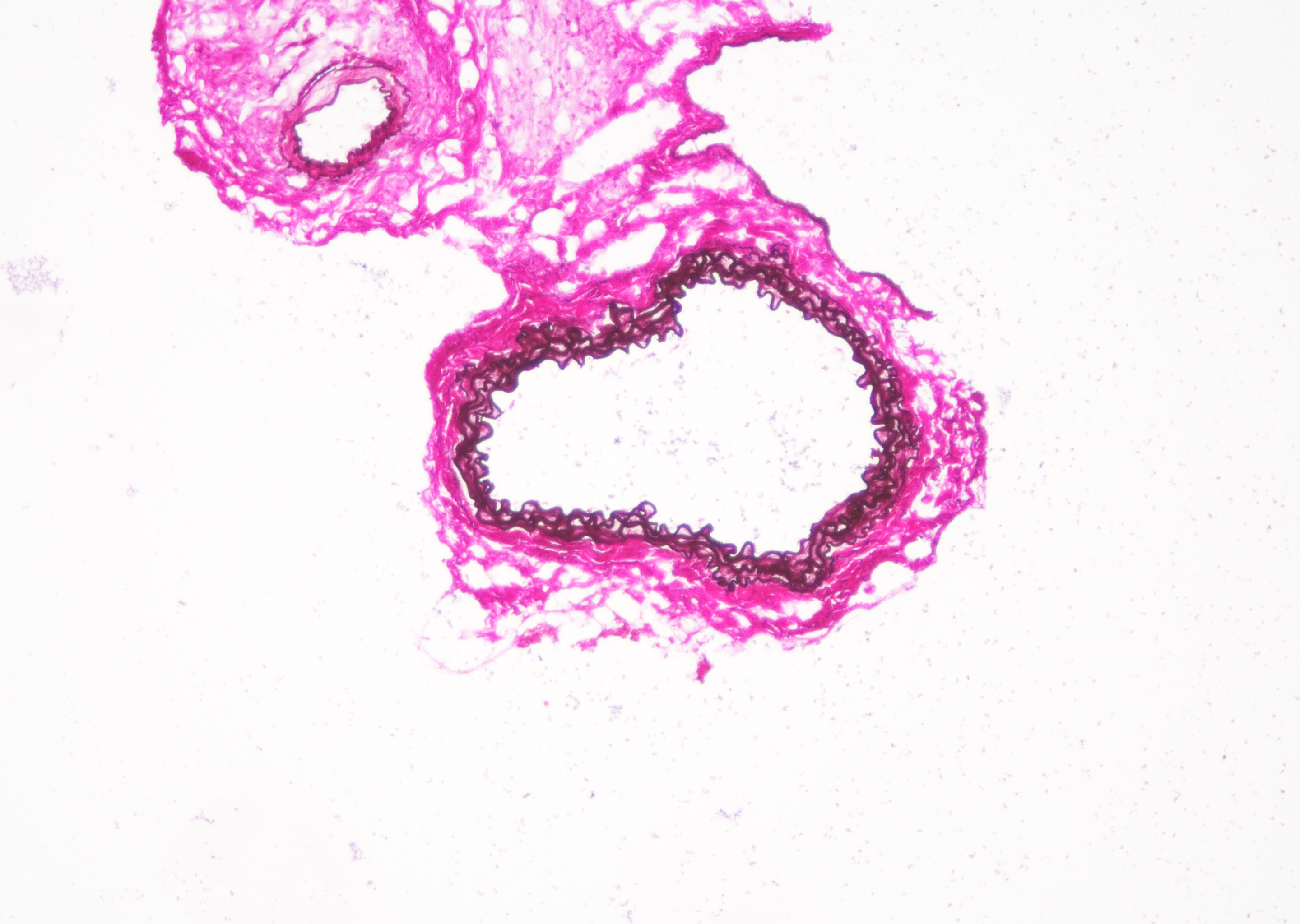

Supplement: Supplementary file 7 — Source data Fig. 6 [file 44321_2025_318_MOESM7_ESM.zip › Figure 6/Figure 6E/EVG Staining/CL316,243 AAV- Control 100um.tif]

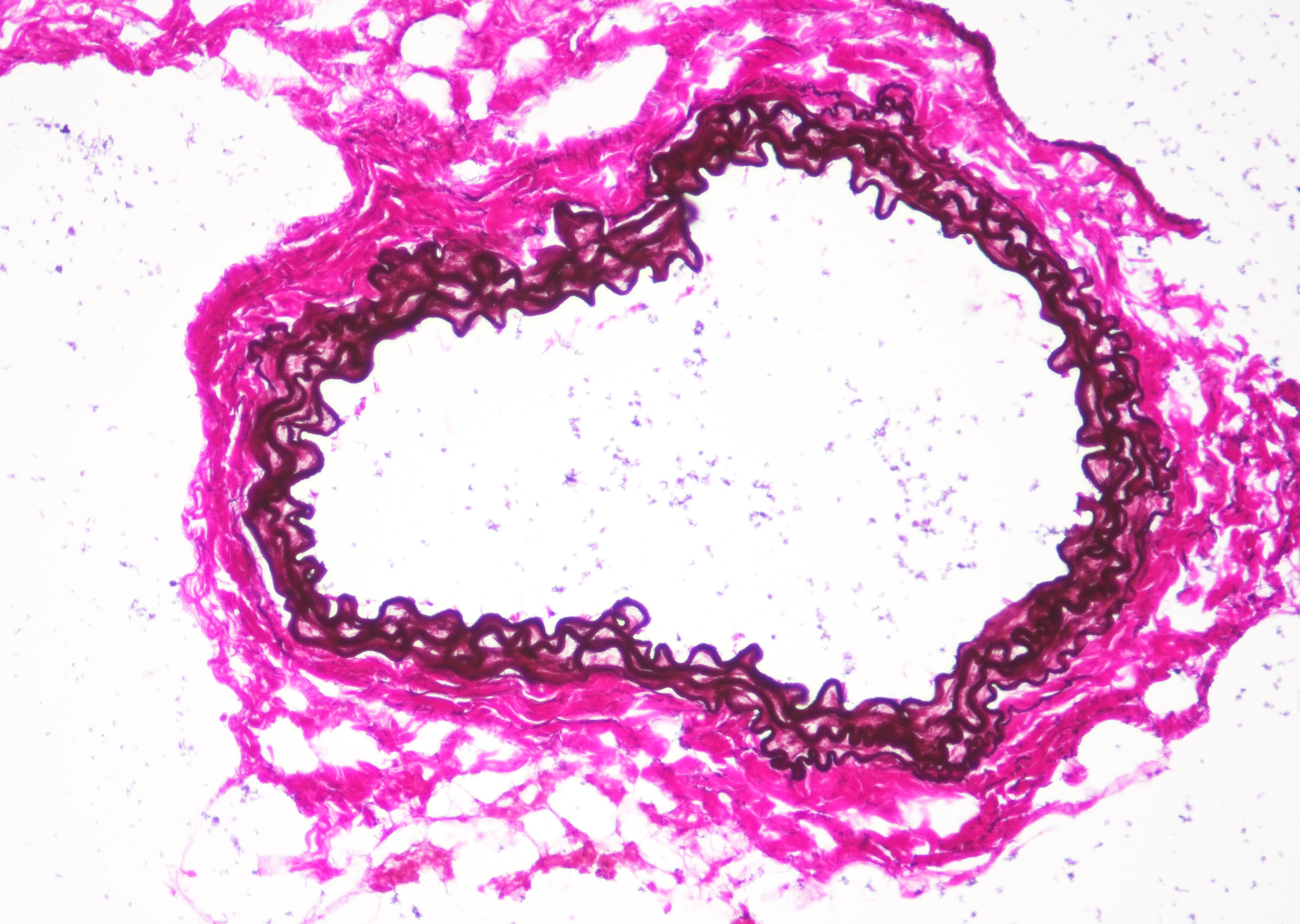

Supplement: Supplementary file 7 — Source data Fig. 6 [file 44321_2025_318_MOESM7_ESM.zip › Figure 6/Figure 6E/EVG Staining/CL316,243 AAV- Control 50um.tif]

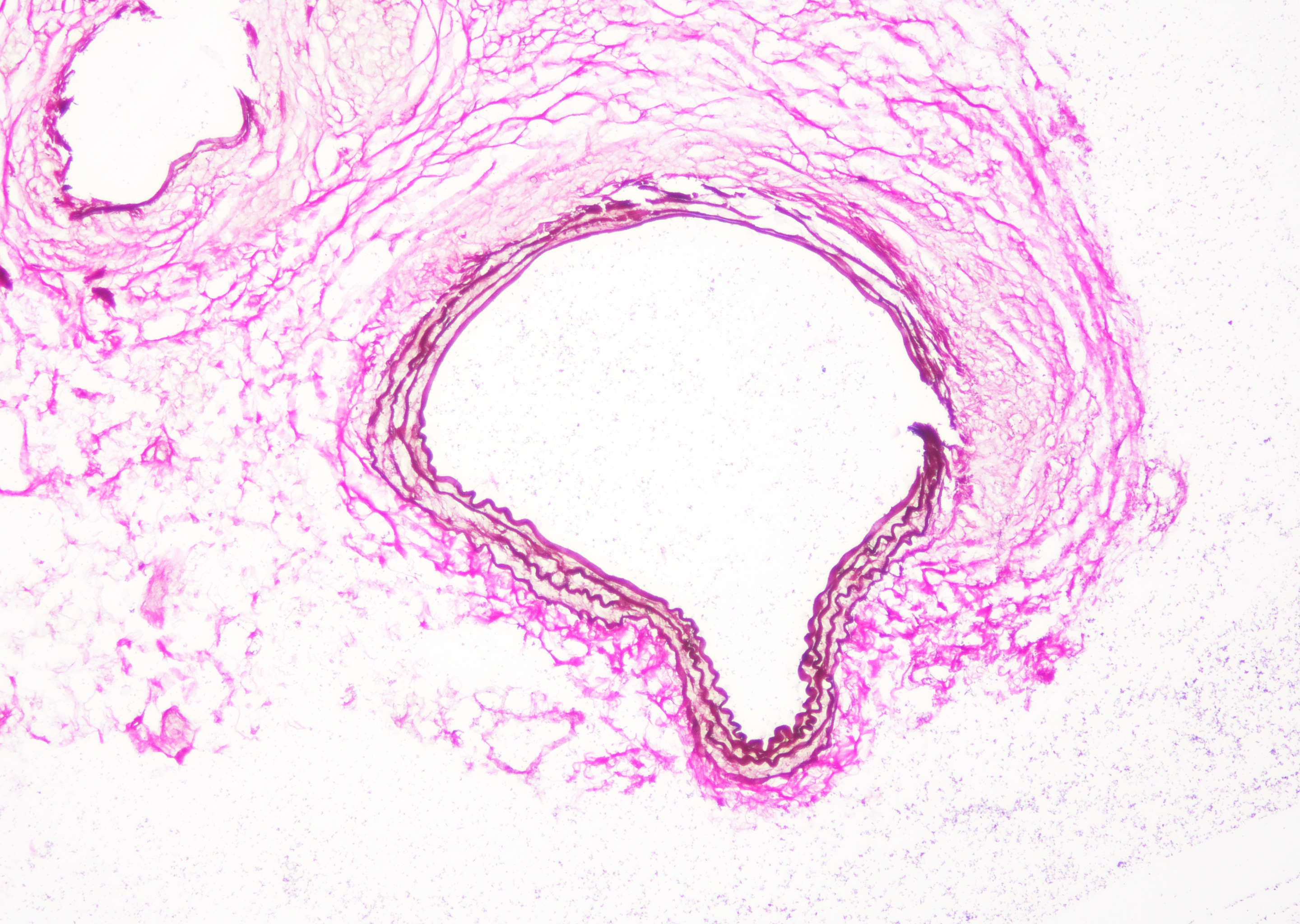

Supplement: Supplementary file 7 — Source data Fig. 6 [file 44321_2025_318_MOESM7_ESM.zip › Figure 6/Figure 6E/EVG Staining/Saline AAV-Adipoq-cre 100um.tif]

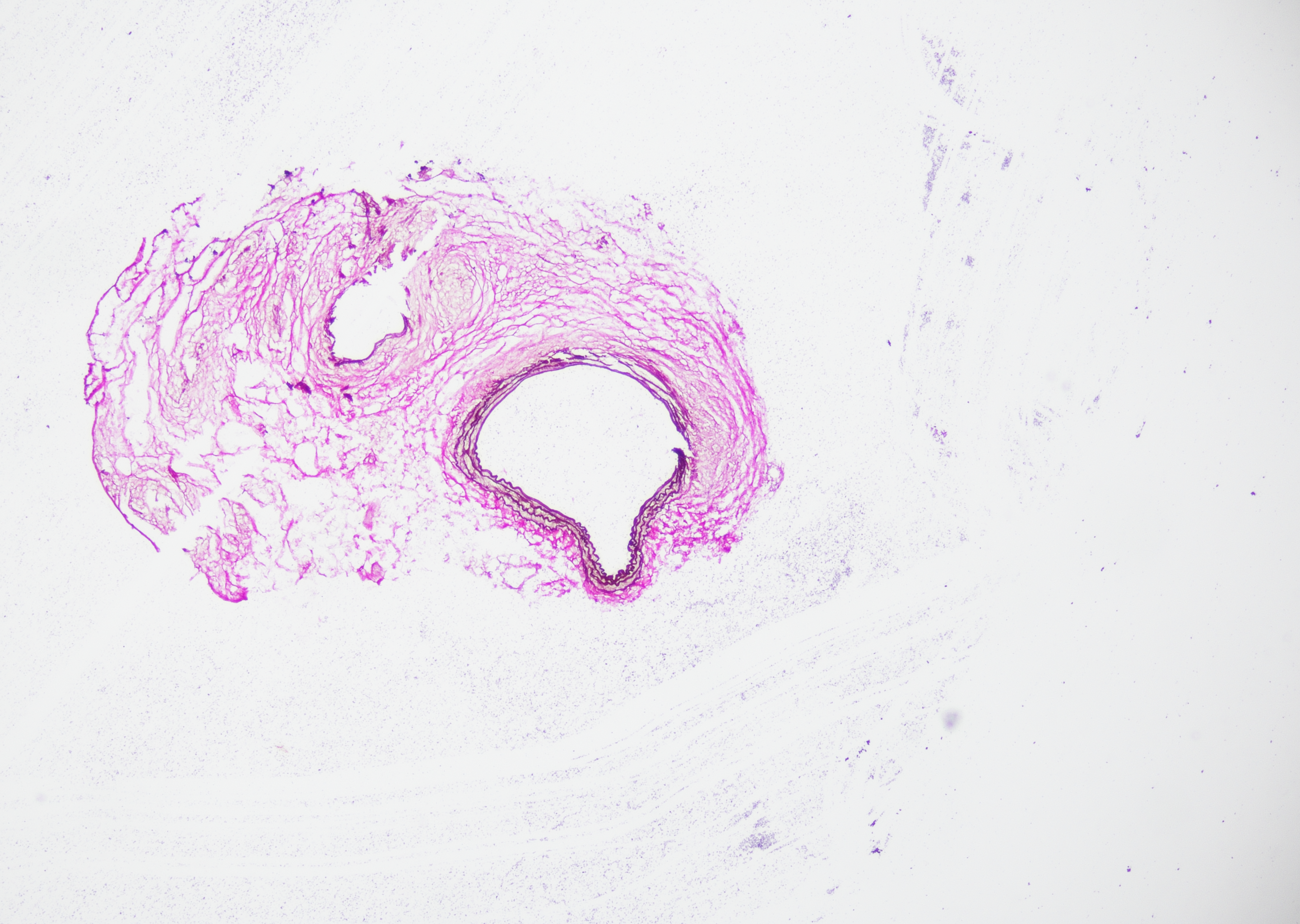

Supplement: Supplementary file 7 — Source data Fig. 6 [file 44321_2025_318_MOESM7_ESM.zip › Figure 6/Figure 6E/EVG Staining/Saline AAV-Adipoq-cre 200um.tif]

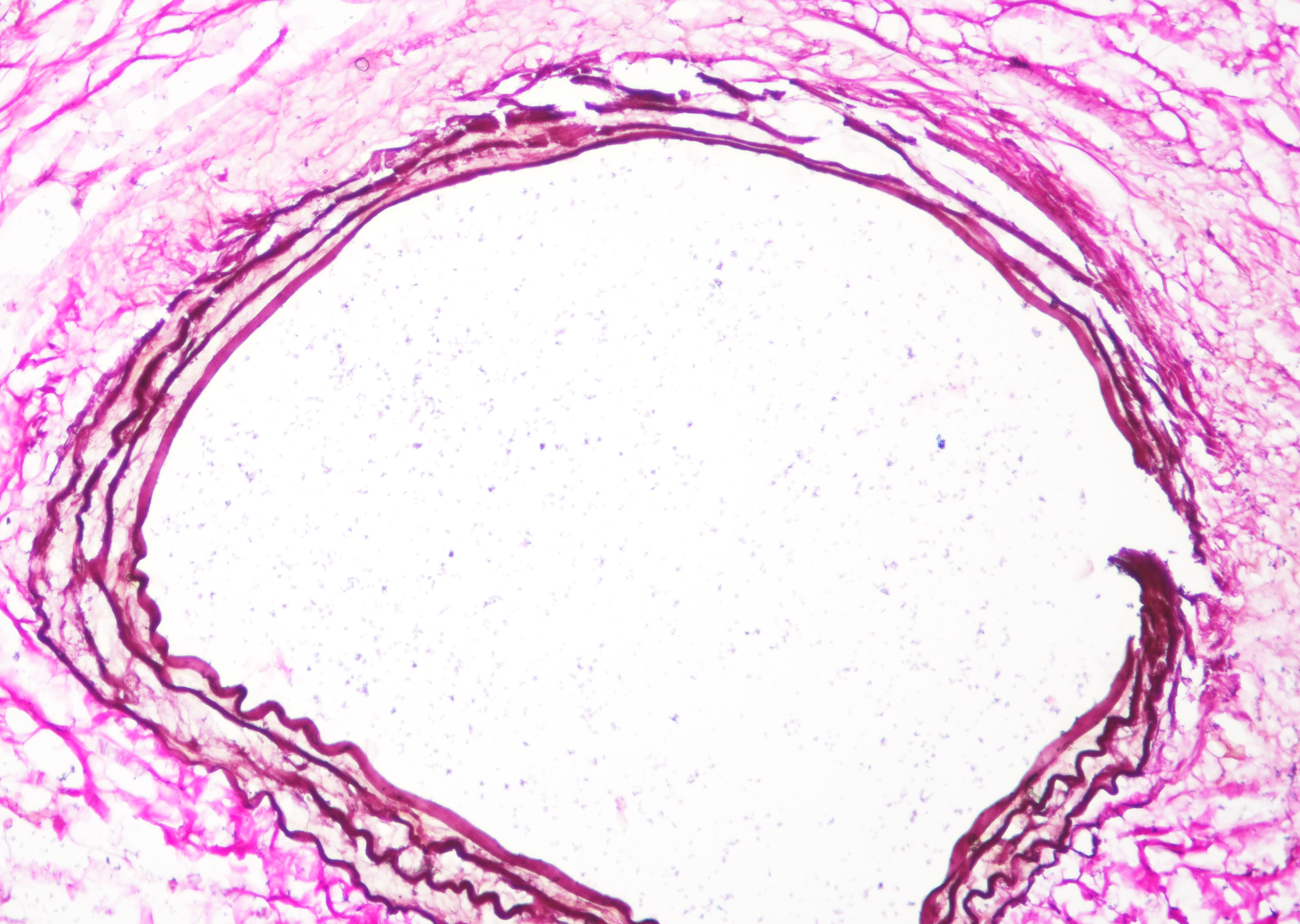

Supplement: Supplementary file 7 — Source data Fig. 6 [file 44321_2025_318_MOESM7_ESM.zip › Figure 6/Figure 6E/EVG Staining/Saline AAV-Adipoq-cre 50um.tif]

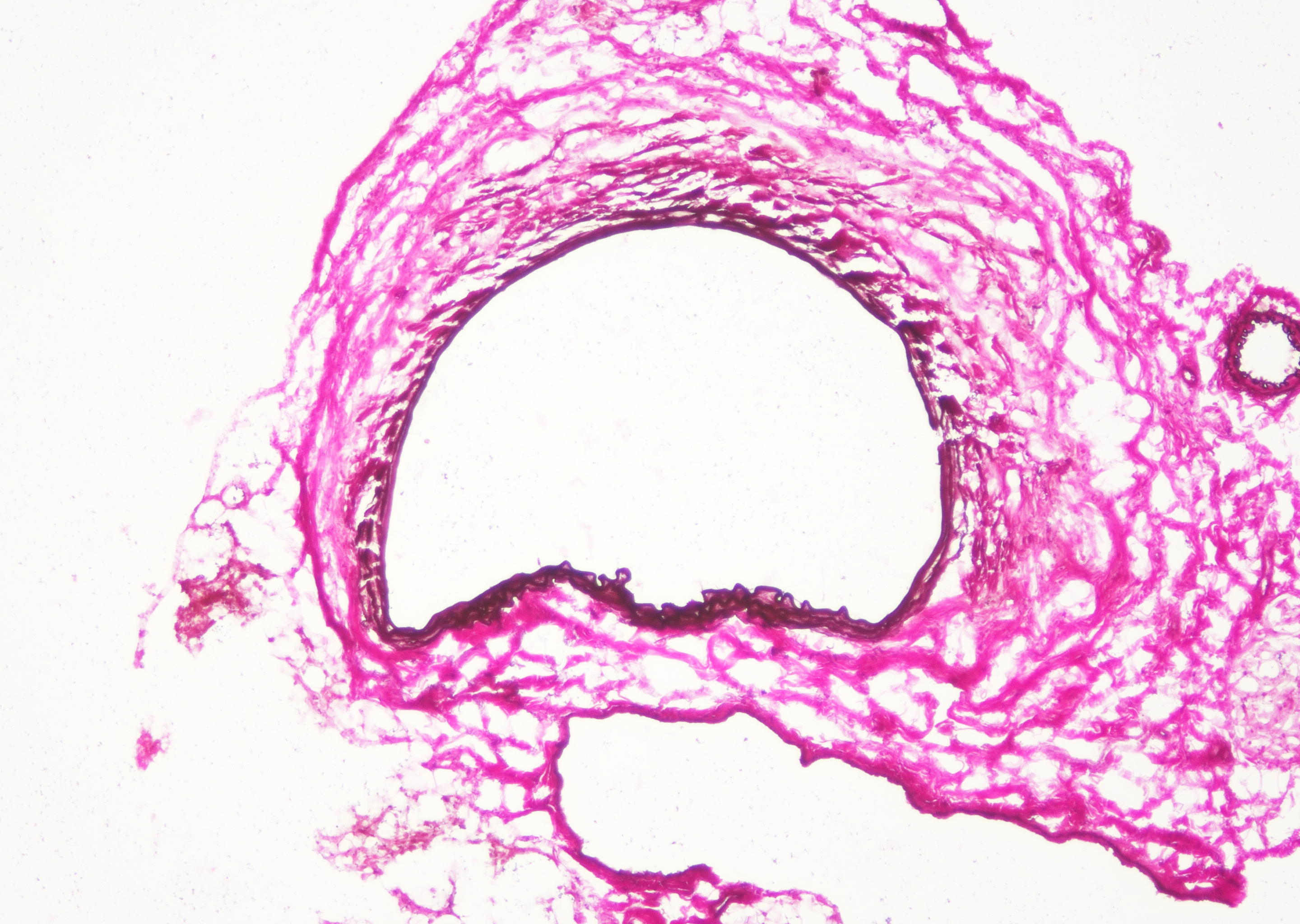

Supplement: Supplementary file 7 — Source data Fig. 6 [file 44321_2025_318_MOESM7_ESM.zip › Figure 6/Figure 6E/EVG Staining/Saline AAV-Control 100um.tif]

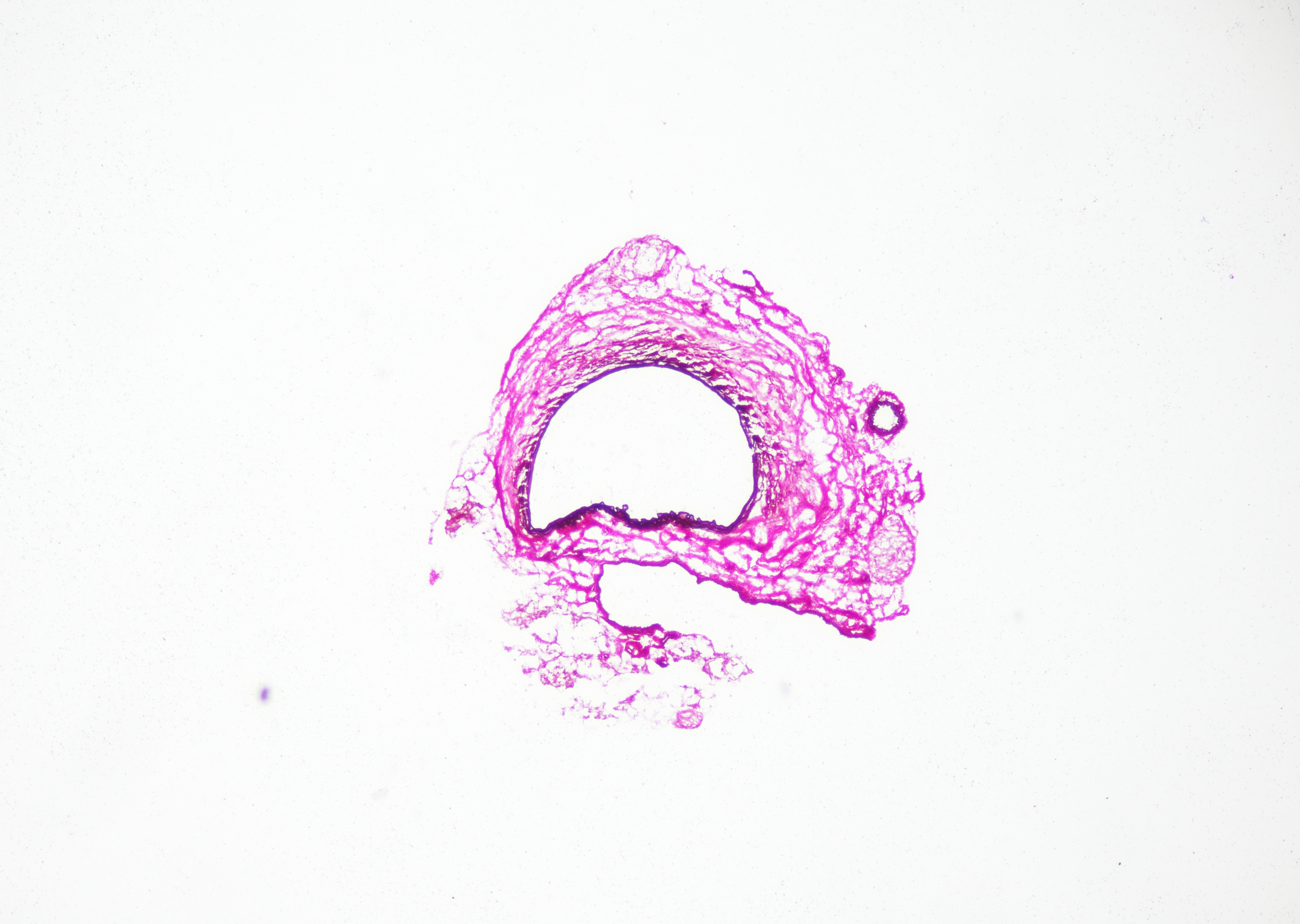

Supplement: Supplementary file 7 — Source data Fig. 6 [file 44321_2025_318_MOESM7_ESM.zip › Figure 6/Figure 6E/EVG Staining/Saline AAV-Control 200um.tif]

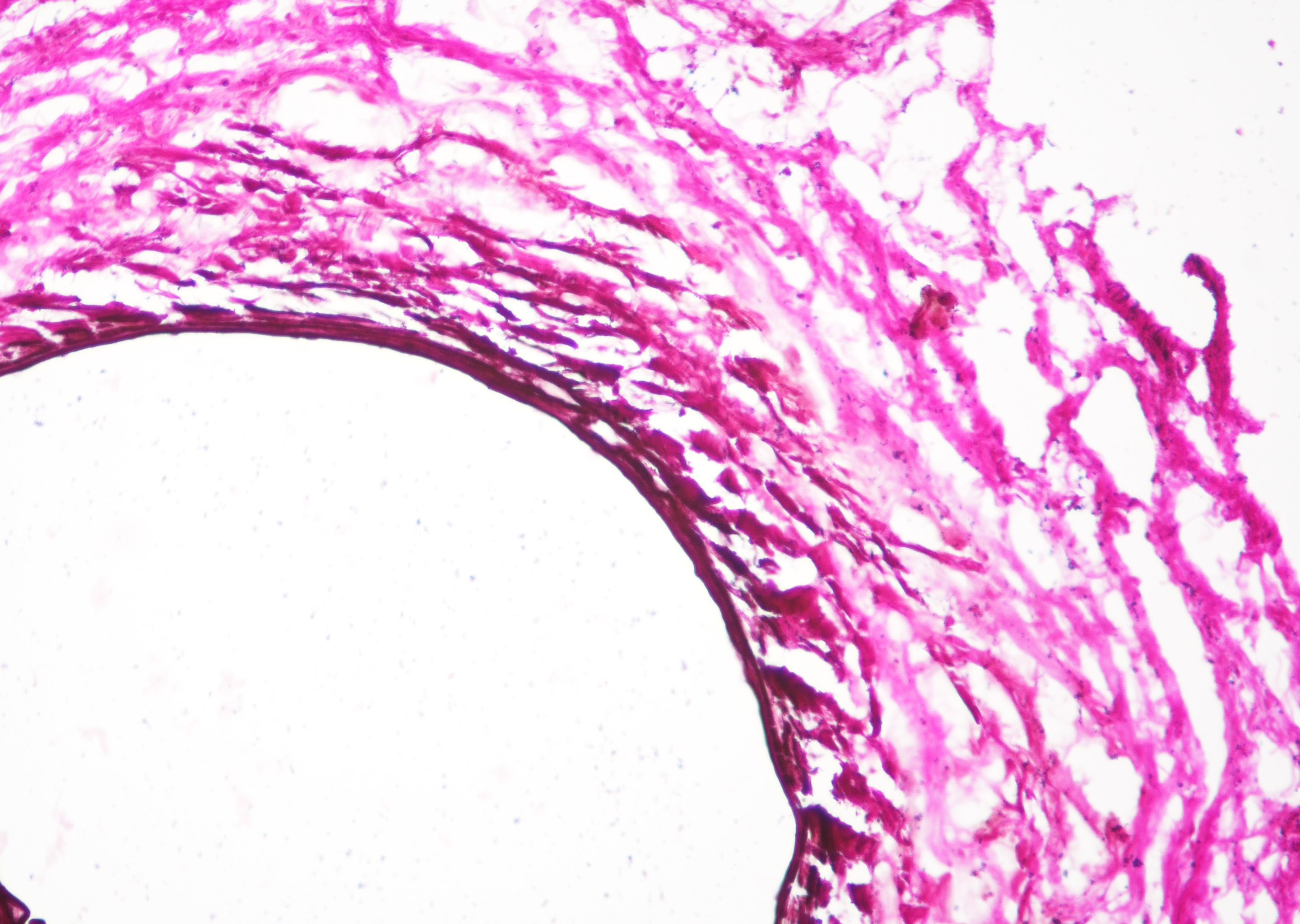

Supplement: Supplementary file 7 — Source data Fig. 6 [file 44321_2025_318_MOESM7_ESM.zip › Figure 6/Figure 6E/EVG Staining/Saline AAV-Control 50um.tif]

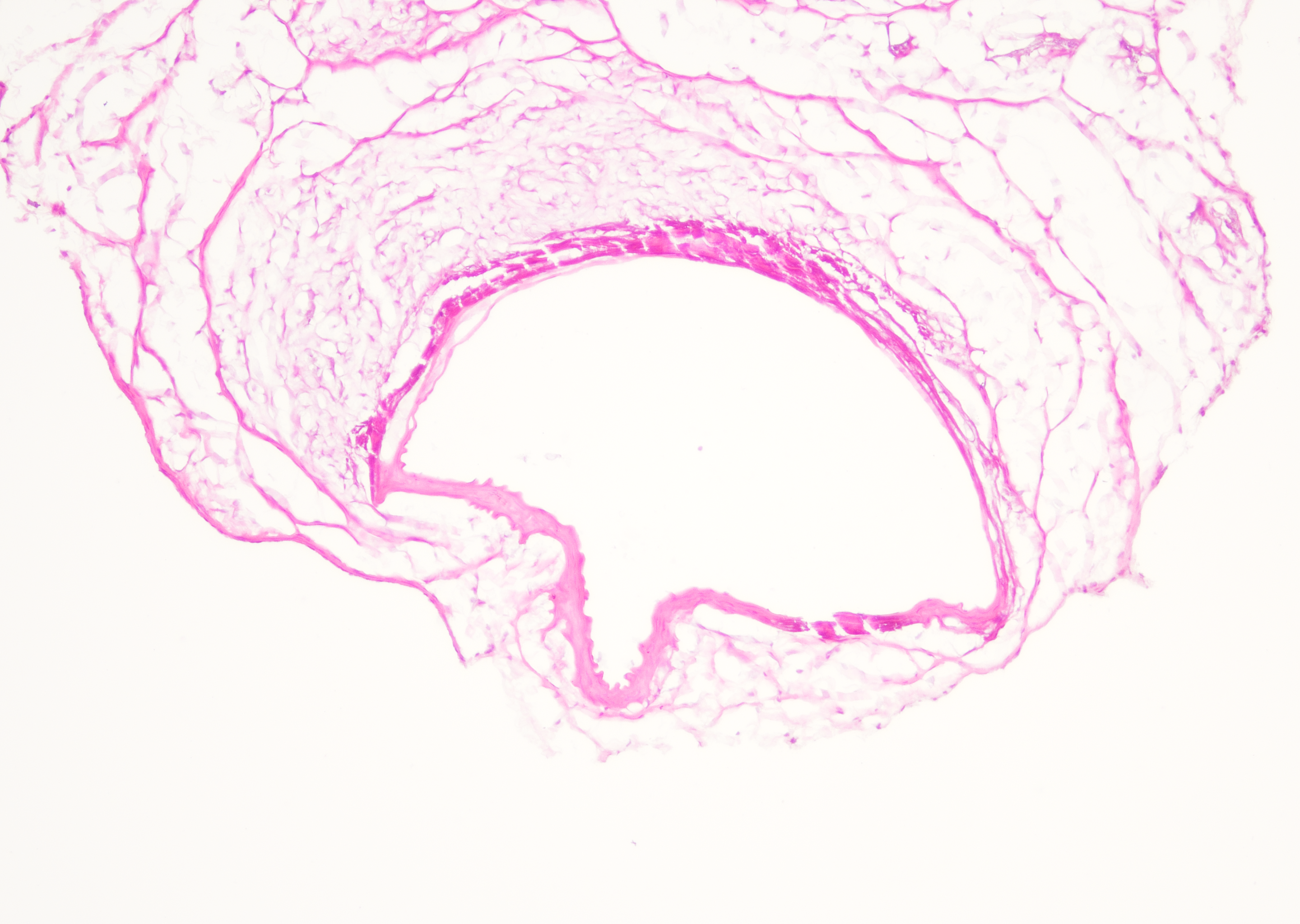

Supplement: Supplementary file 7 — Source data Fig. 6 [file 44321_2025_318_MOESM7_ESM.zip › Figure 6/Figure 6E/HE Staining/CL316,243 AAV-Adipoq-cre 100um.tif]
