# Supplementary material for: Brown remodeling of white adipose tissue protects against abdominal aortic aneurysm via batokine FSTL1
Source: EMBO Mol Med. 2025 Oct 9;17(11):3080–109. doi: 10.1038/s44321-025-00318-z (PMC12603302; doi:10.1038/s44321-025-00318-z)
Supplement: Supplementary file 4 — Source data Fig. 3 [file 44321_2025_318_MOESM4_ESM.zip › Figure 3/Figure 3F/README.pptx]

## Slide 1
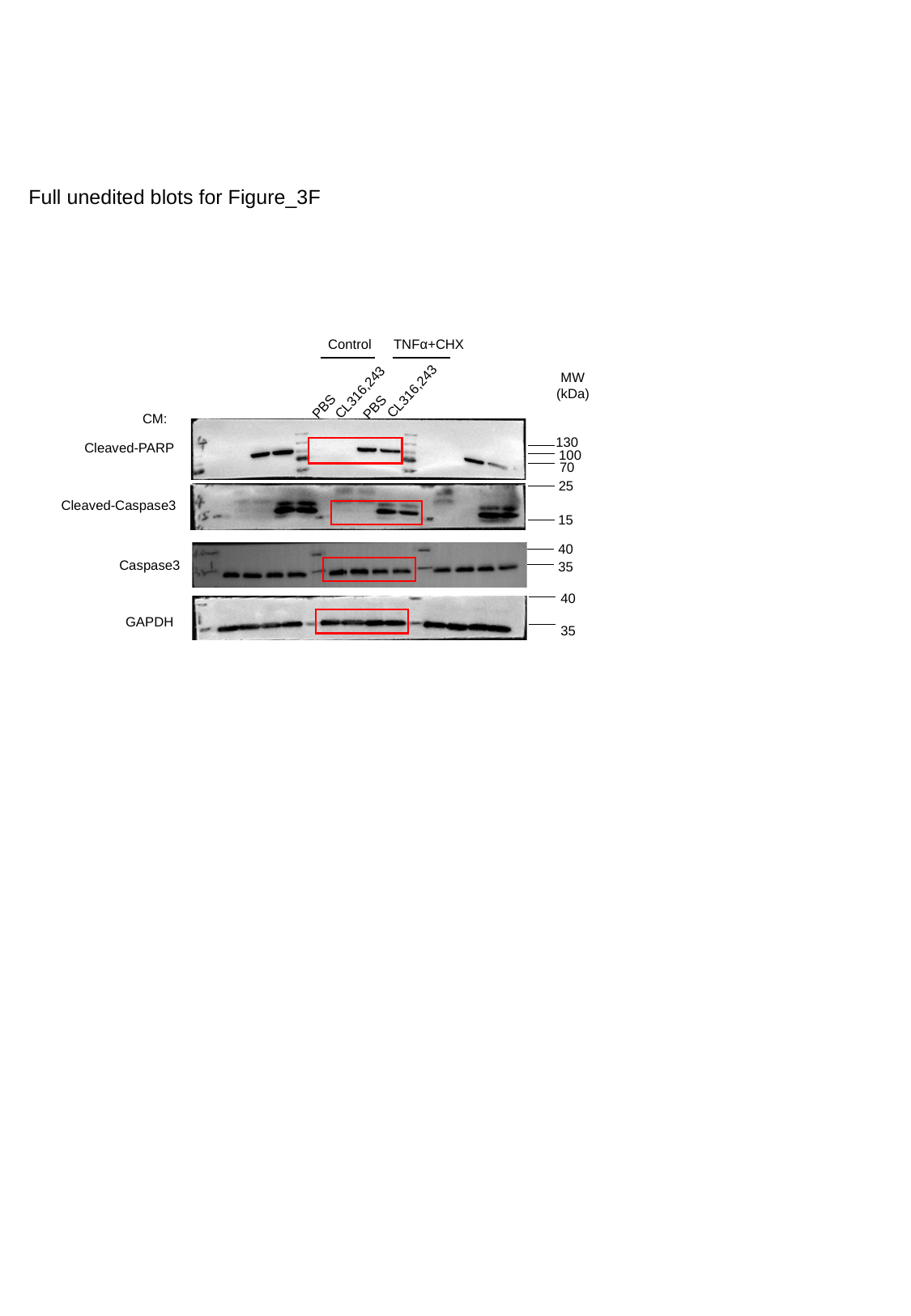

Full unedited blots for Figure_3F
Control
TNFα+CHX
MW
(kDa)
CL316,243
CL316,243
PBS
PBS
CM:
130
Cleaved-PARP
100
70
25
Cleaved-Caspase3
15
40
Caspase3
35
40
GAPDH
35
