# Supplementary material for: Brown remodeling of white adipose tissue protects against abdominal aortic aneurysm via batokine FSTL1
Source: EMBO Mol Med. 2025 Oct 9;17(11):3080–109. doi: 10.1038/s44321-025-00318-z (PMC12603302; doi:10.1038/s44321-025-00318-z)
Supplement: Supplementary file 5 — Source data Fig. 4 [file 44321_2025_318_MOESM5_ESM.zip › Figure 4/Figure 4K/README.pptx]

## Slide 1
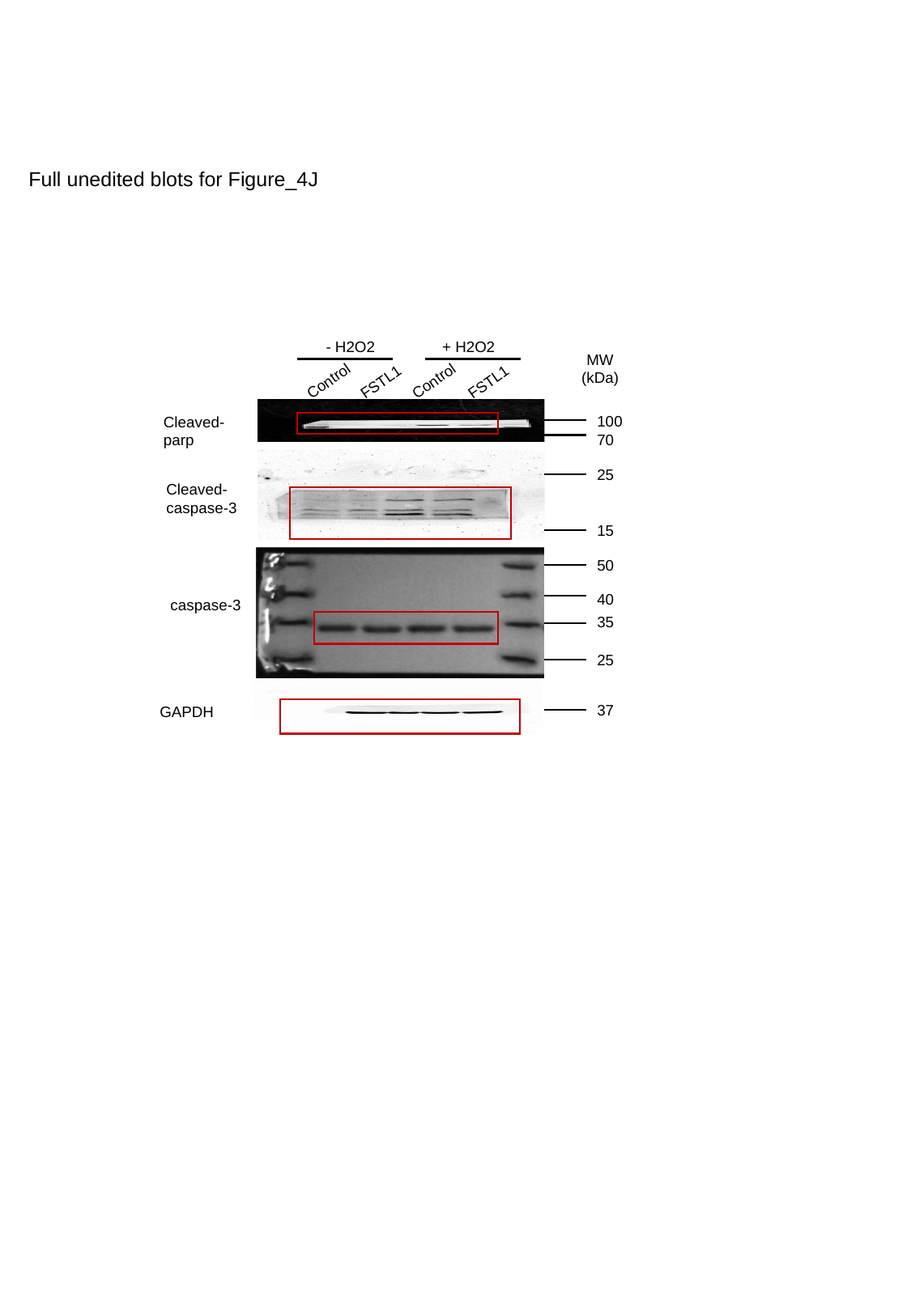

Full unedited blots for Figure_4J
- H2O2
+ H2O2
MW
(kDa)
FSTL1
Control
FSTL1
Control
100
Cleaved-parp
70
25
Cleaved-
caspase-3
15
50
40
caspase-3
35
25
37
GAPDH
