# Supplementary material for: Brown remodeling of white adipose tissue protects against abdominal aortic aneurysm via batokine FSTL1
Source: EMBO Mol Med. 2025 Oct 9;17(11):3080–109. doi: 10.1038/s44321-025-00318-z (PMC12603302; doi:10.1038/s44321-025-00318-z)
Supplement: Supplementary file 6 — Source data Fig. 5 [file 44321_2025_318_MOESM6_ESM.zip › Figure 5/Figure 5G/README.pptx]

## Slide 1
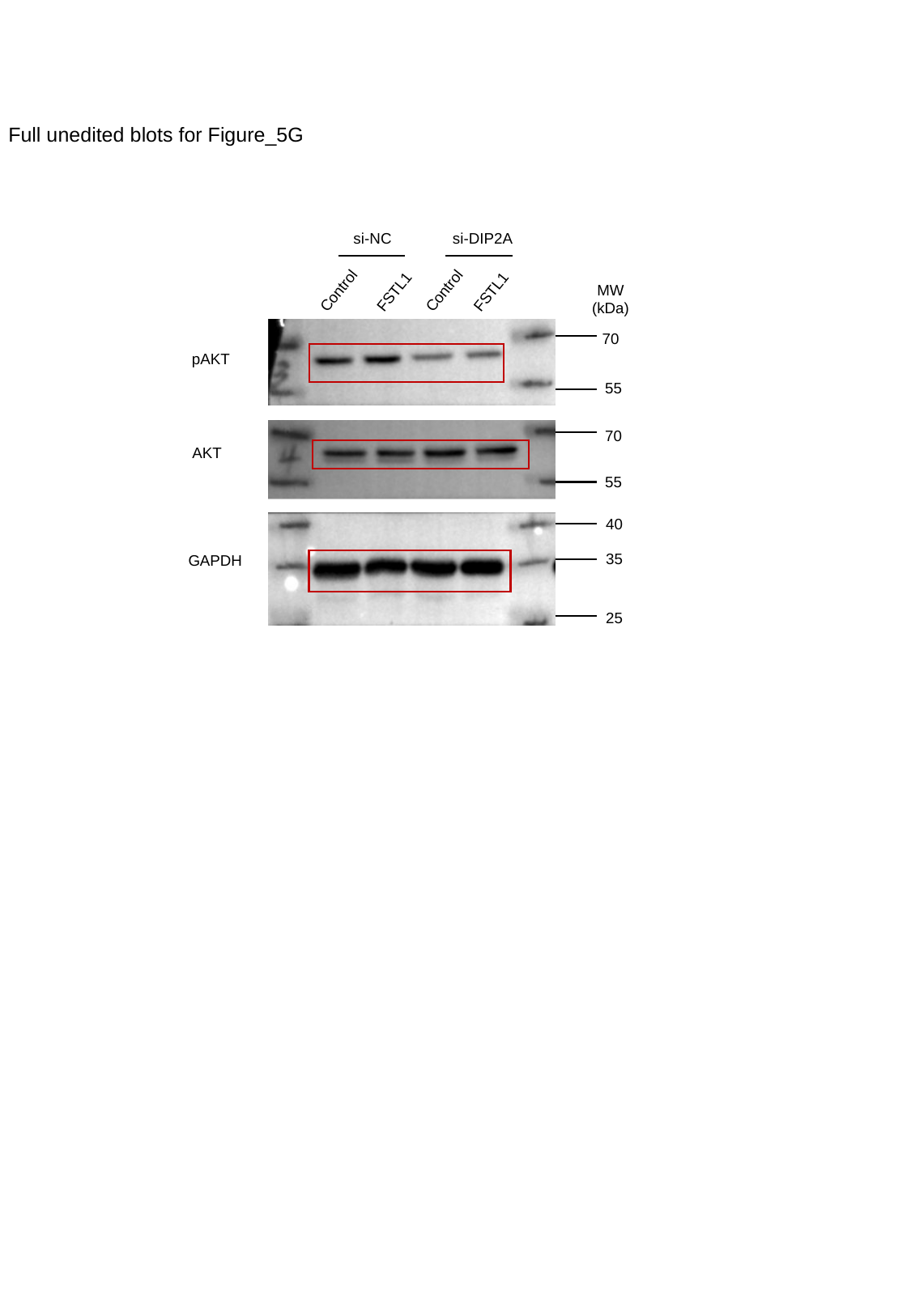

Full unedited blots for Figure_5G
si-NC
si-DIP2A
MW
(kDa)
Control
Control
FSTL1
FSTL1
70
pAKT
55
70
AKT
55
40
35
GAPDH
25
