# Supplementary figures and images for: Brown remodeling of white adipose tissue protects against abdominal aortic aneurysm via batokine FSTL1 (part 2 of 2)
Source: EMBO Mol Med. 2025 Oct 9;17(11):3080–109. doi: 10.1038/s44321-025-00318-z (PMC12603302; doi:10.1038/s44321-025-00318-z)

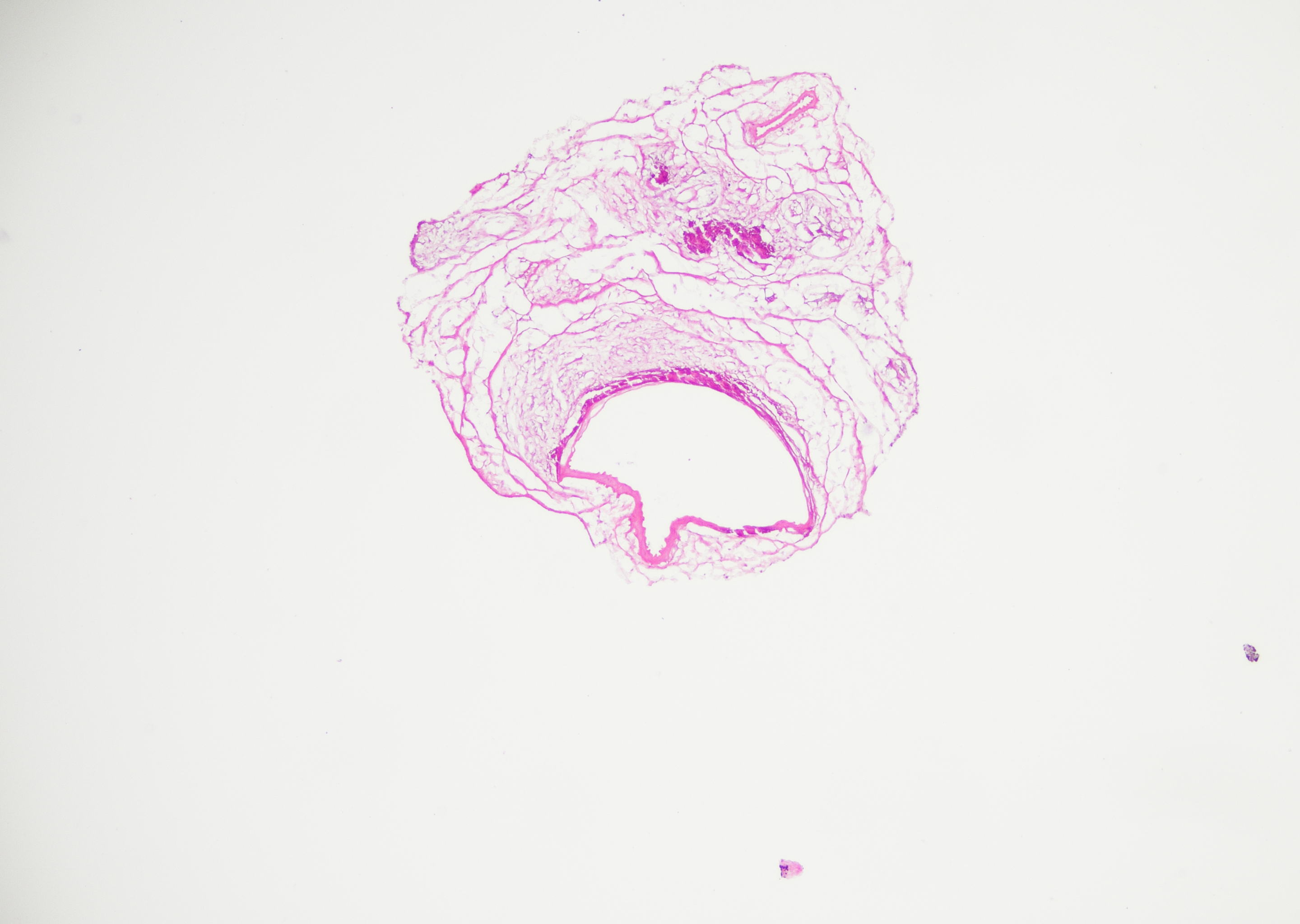

Supplement: Supplementary file 7 — Source data Fig. 6 [file 44321_2025_318_MOESM7_ESM.zip › Figure 6/Figure 6E/HE Staining/CL316,243 AAV-Adipoq-cre 200um.tif]

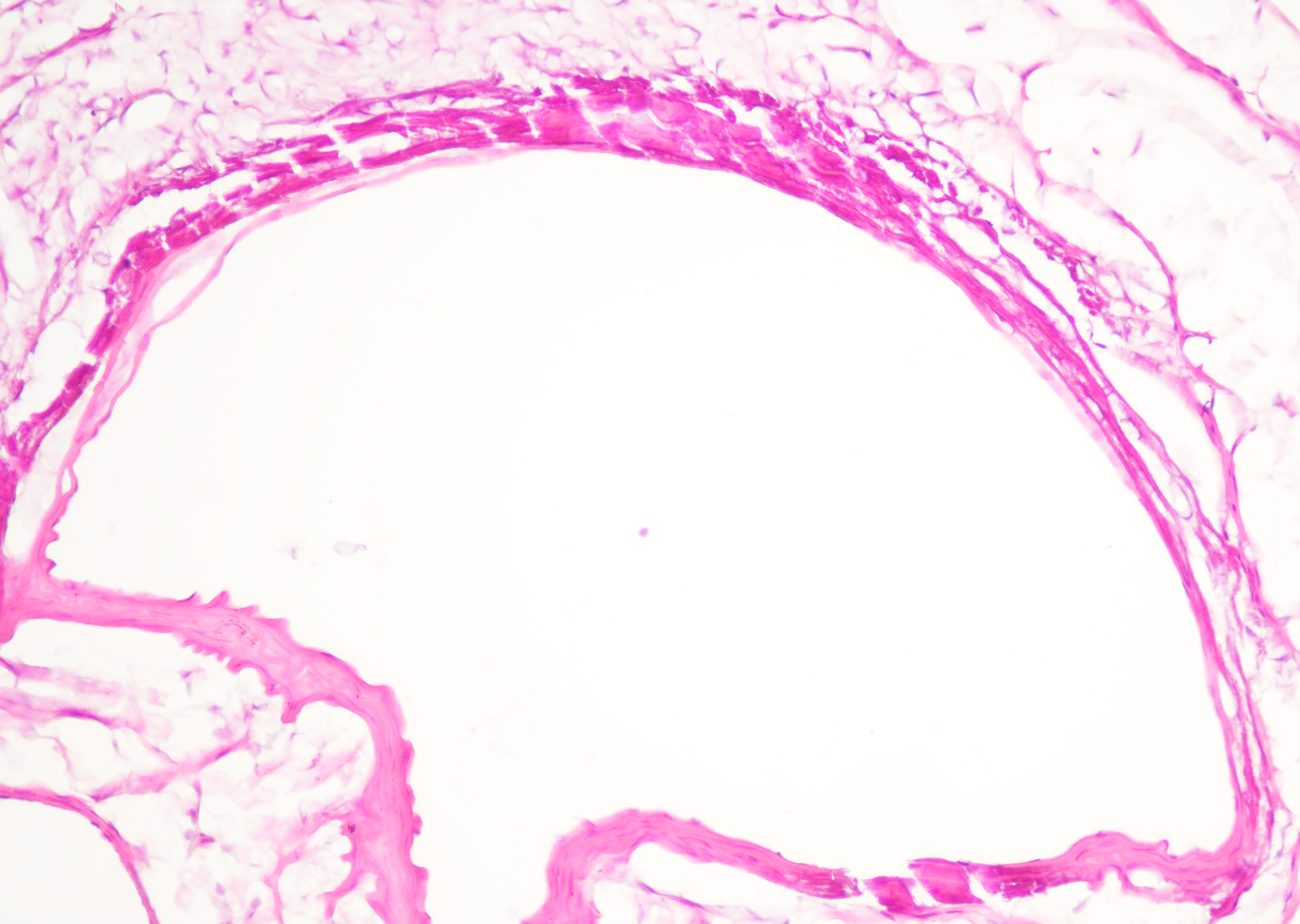

Supplement: Supplementary file 7 — Source data Fig. 6 [file 44321_2025_318_MOESM7_ESM.zip › Figure 6/Figure 6E/HE Staining/CL316,243 AAV-Adipoq-cre 50um.tif]

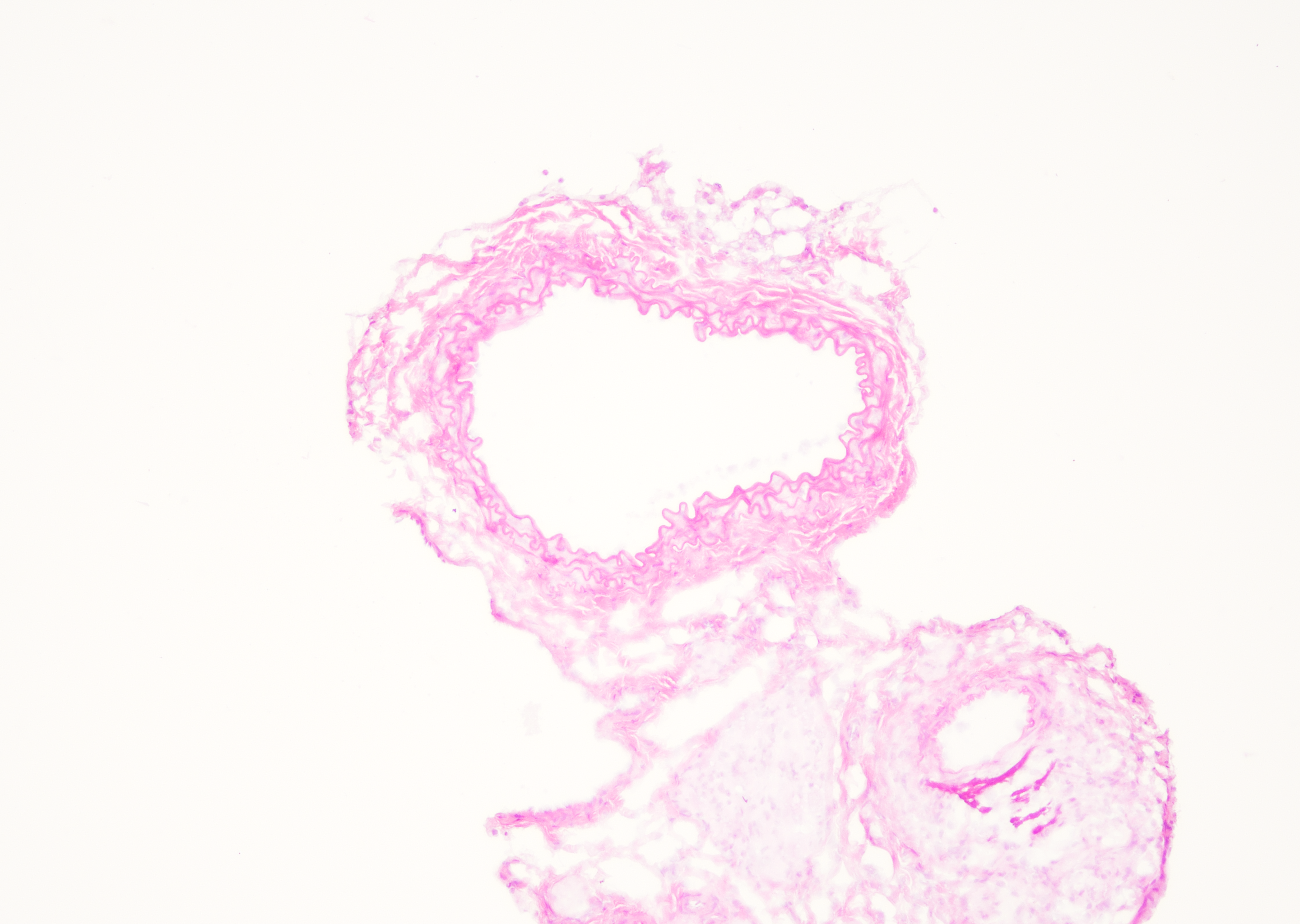

Supplement: Supplementary file 7 — Source data Fig. 6 [file 44321_2025_318_MOESM7_ESM.zip › Figure 6/Figure 6E/HE Staining/CL316,243 AAV Control 100um.tif]

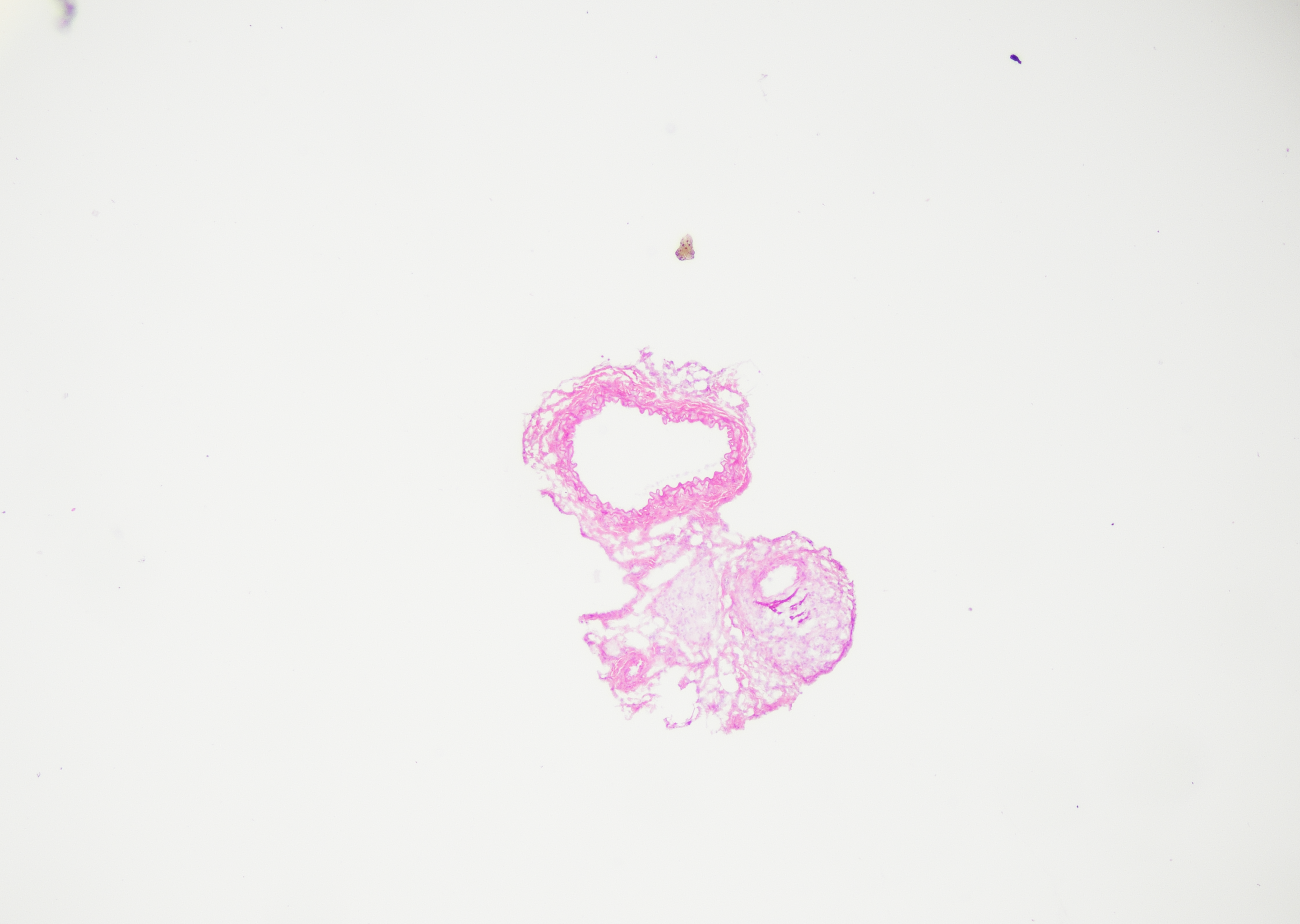

Supplement: Supplementary file 7 — Source data Fig. 6 [file 44321_2025_318_MOESM7_ESM.zip › Figure 6/Figure 6E/HE Staining/CL316,243 AAV Control 200um.tif]

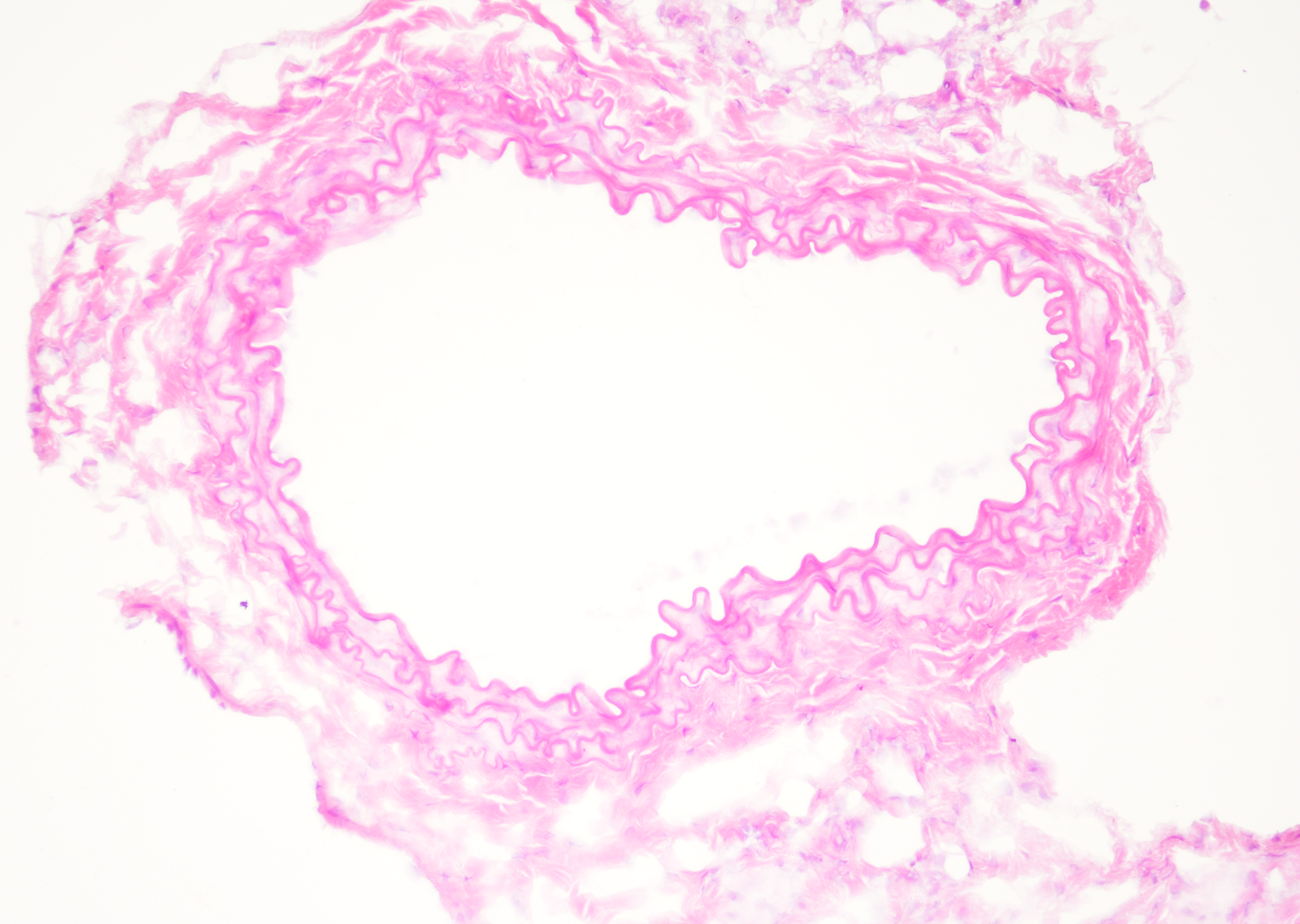

Supplement: Supplementary file 7 — Source data Fig. 6 [file 44321_2025_318_MOESM7_ESM.zip › Figure 6/Figure 6E/HE Staining/CL316,243 AAV Control 50um.tif]

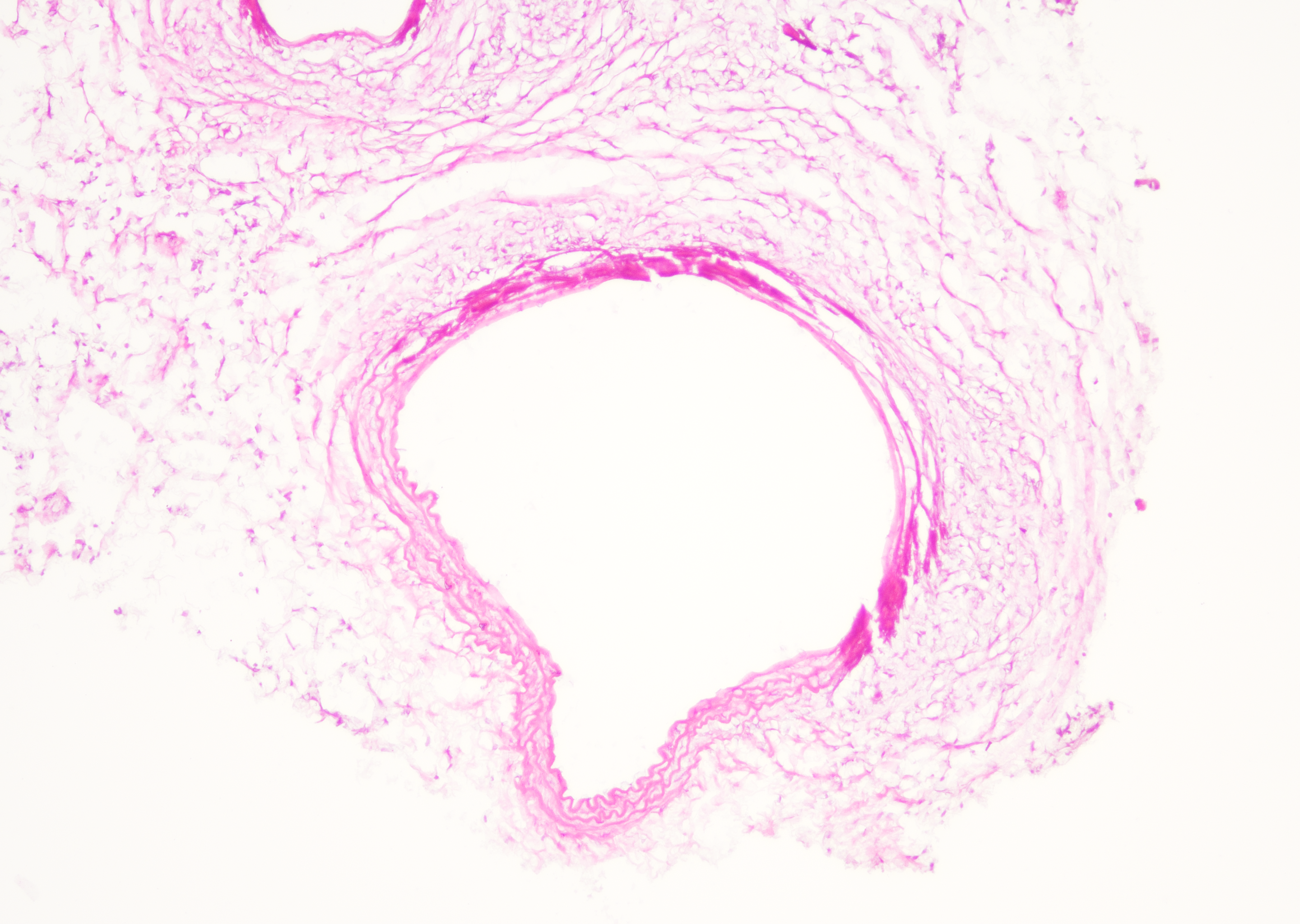

Supplement: Supplementary file 7 — Source data Fig. 6 [file 44321_2025_318_MOESM7_ESM.zip › Figure 6/Figure 6E/HE Staining/Saline AAV-Adipoq-cre 100um.tif]

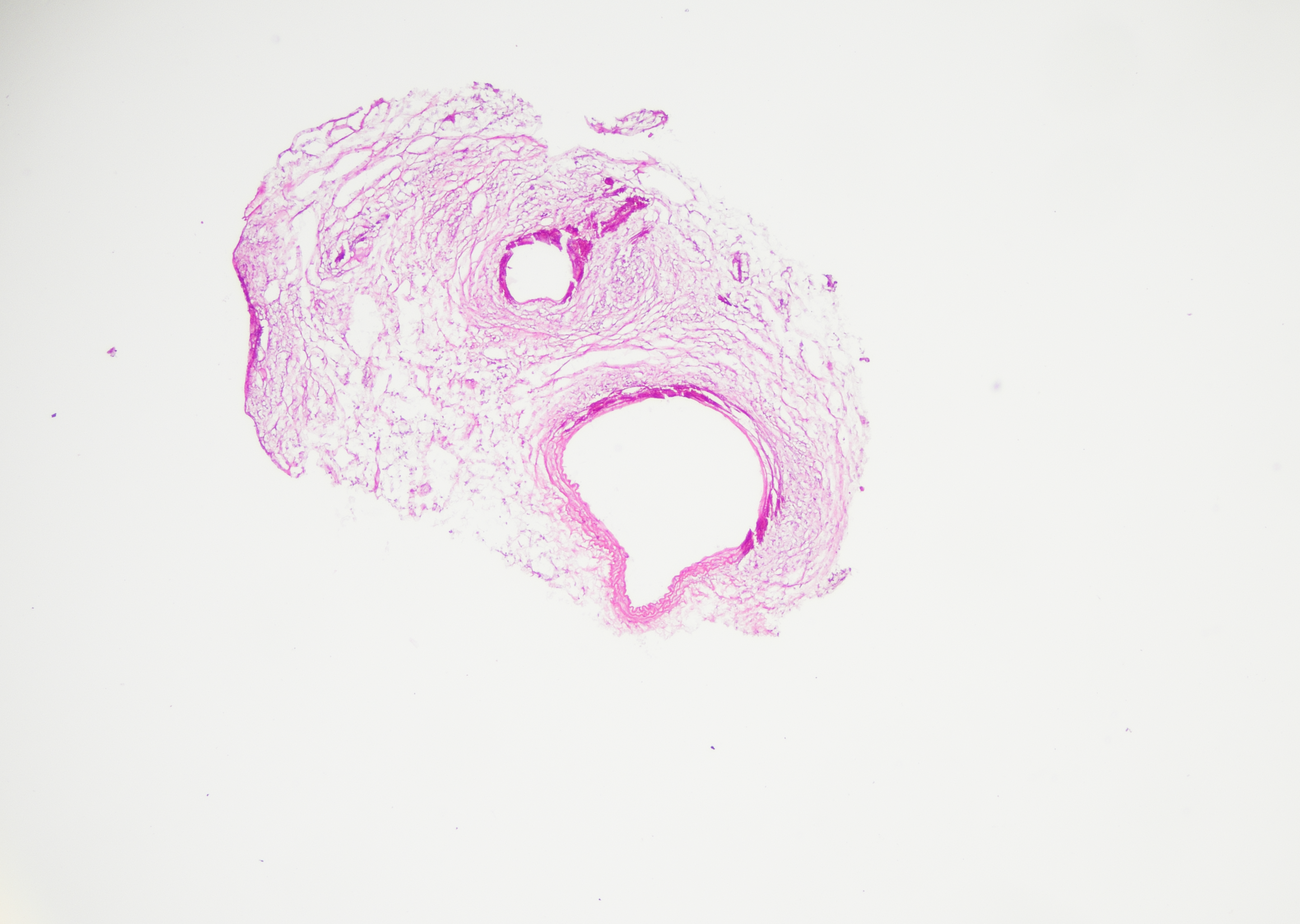

Supplement: Supplementary file 7 — Source data Fig. 6 [file 44321_2025_318_MOESM7_ESM.zip › Figure 6/Figure 6E/HE Staining/Saline AAV-Adipoq-cre 200um.tif]

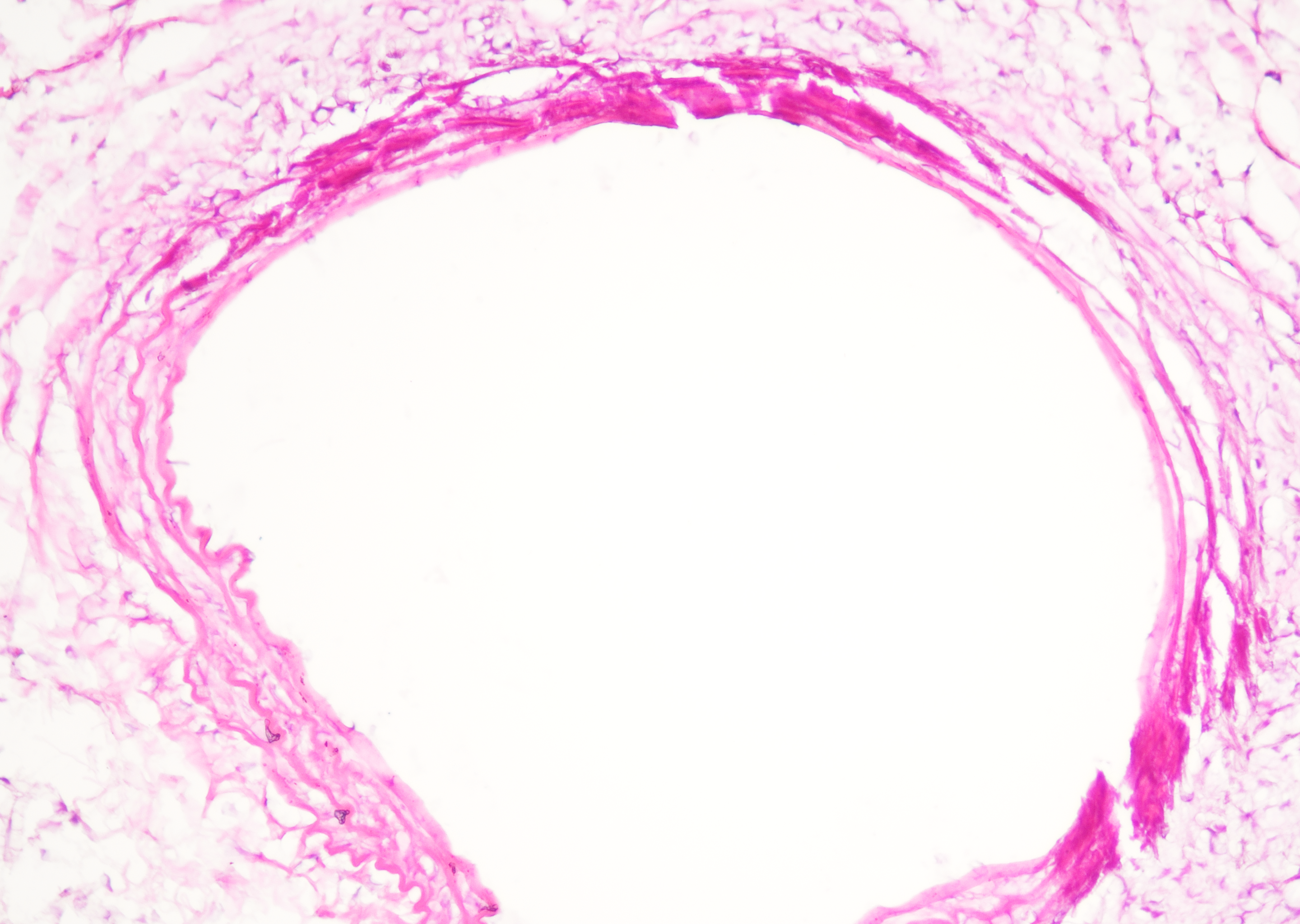

Supplement: Supplementary file 7 — Source data Fig. 6 [file 44321_2025_318_MOESM7_ESM.zip › Figure 6/Figure 6E/HE Staining/Saline AAV-Adipoq-cre 50um.tif]

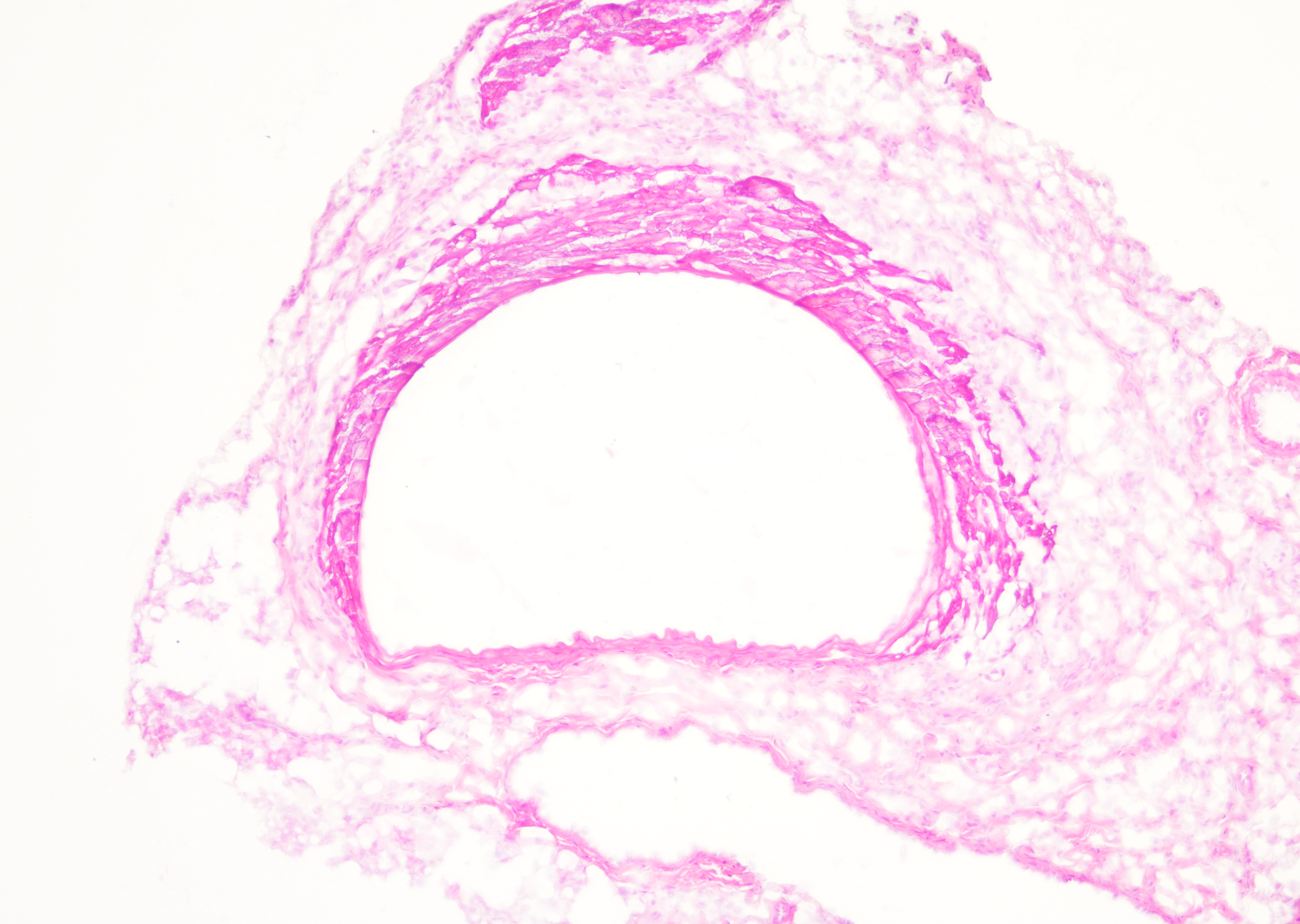

Supplement: Supplementary file 7 — Source data Fig. 6 [file 44321_2025_318_MOESM7_ESM.zip › Figure 6/Figure 6E/HE Staining/Saline AAV-Control 100um.tif]

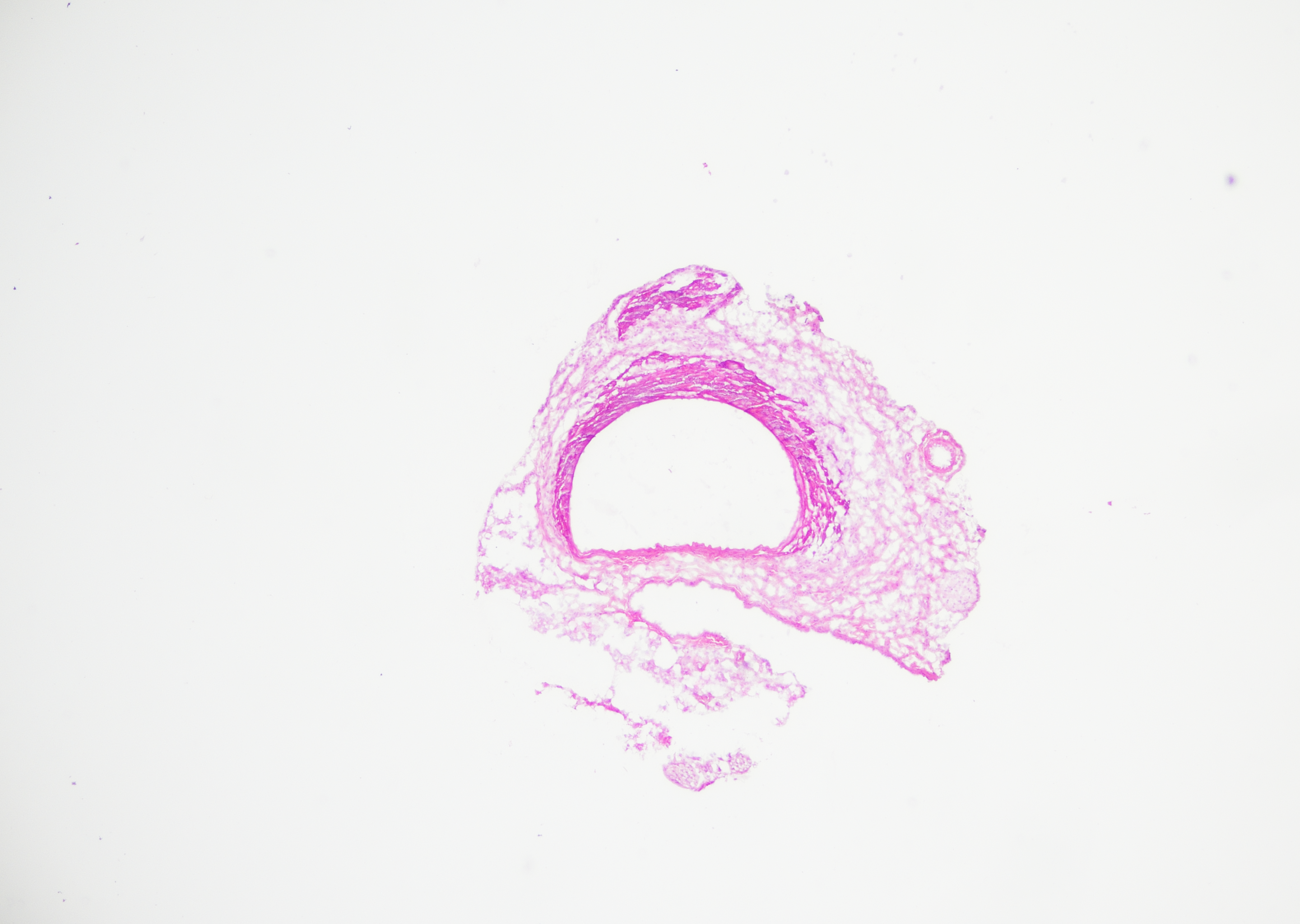

Supplement: Supplementary file 7 — Source data Fig. 6 [file 44321_2025_318_MOESM7_ESM.zip › Figure 6/Figure 6E/HE Staining/Saline AAV-Control 200um.tif]

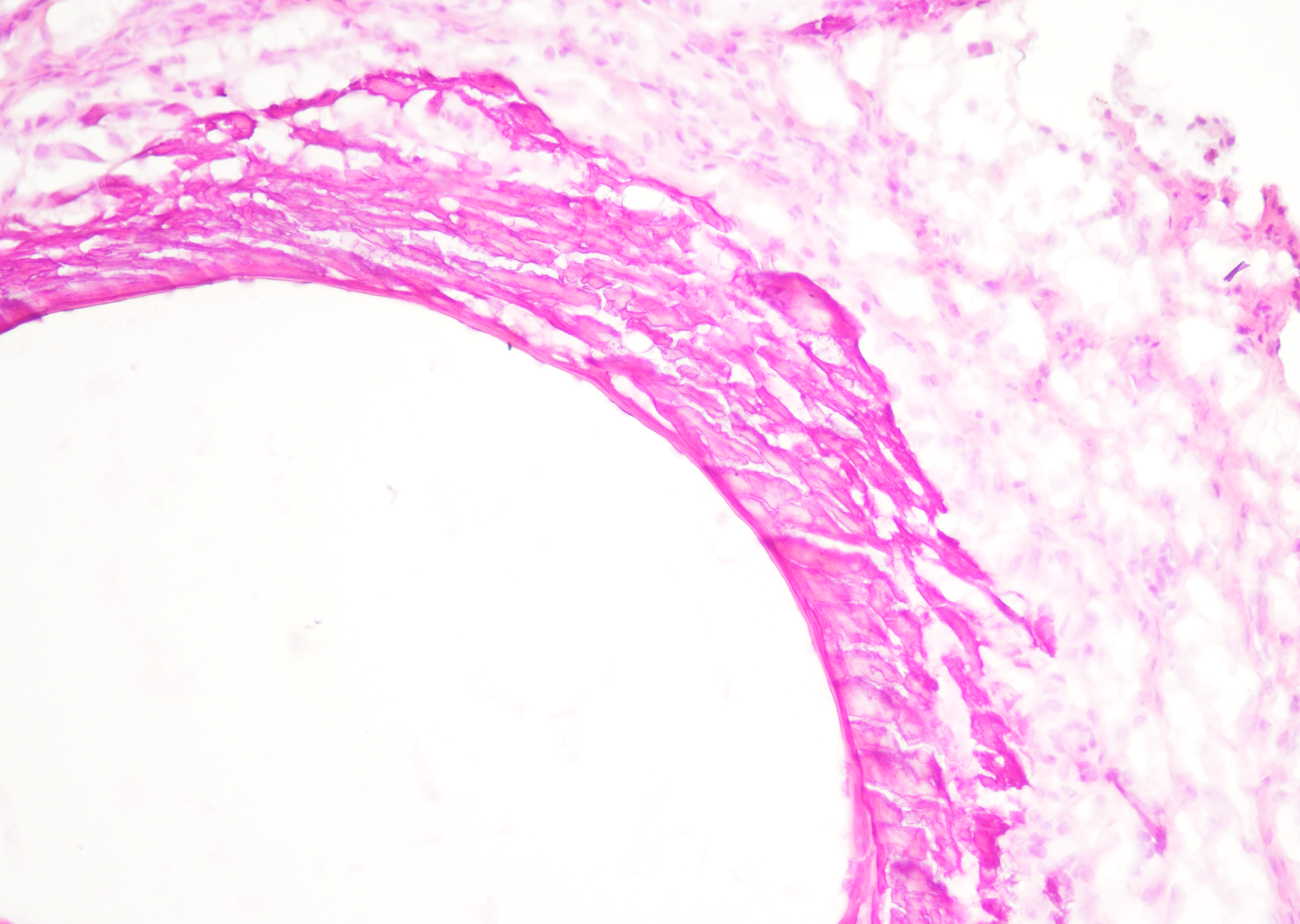

Supplement: Supplementary file 7 — Source data Fig. 6 [file 44321_2025_318_MOESM7_ESM.zip › Figure 6/Figure 6E/HE Staining/Saline AAV-Control 50um.tif]

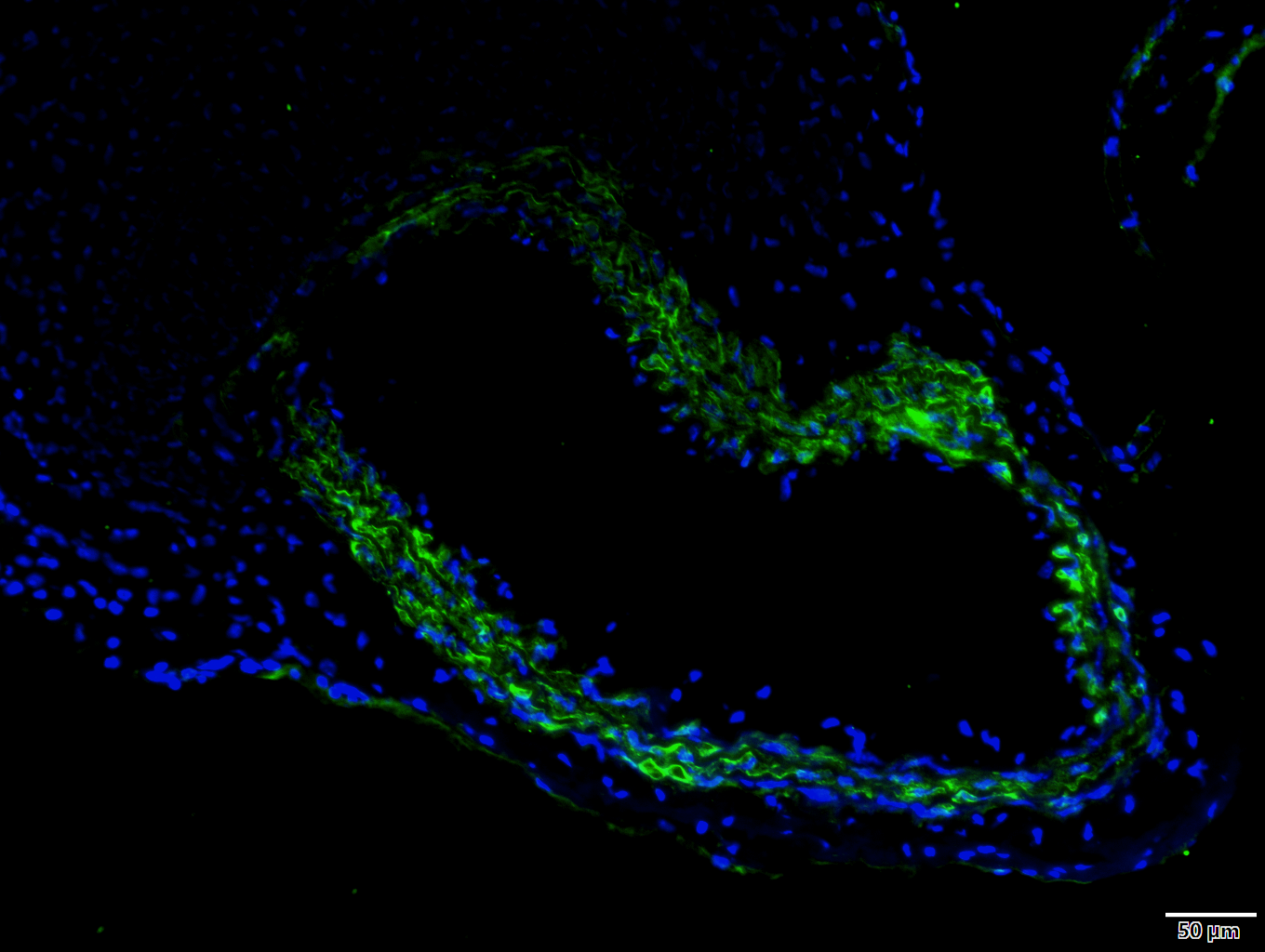

Supplement: Supplementary file 7 — Source data Fig. 6 [file 44321_2025_318_MOESM7_ESM.zip › Figure 6/Figure 6G/AAV-Adipoq-CRE CL316,243.tif]

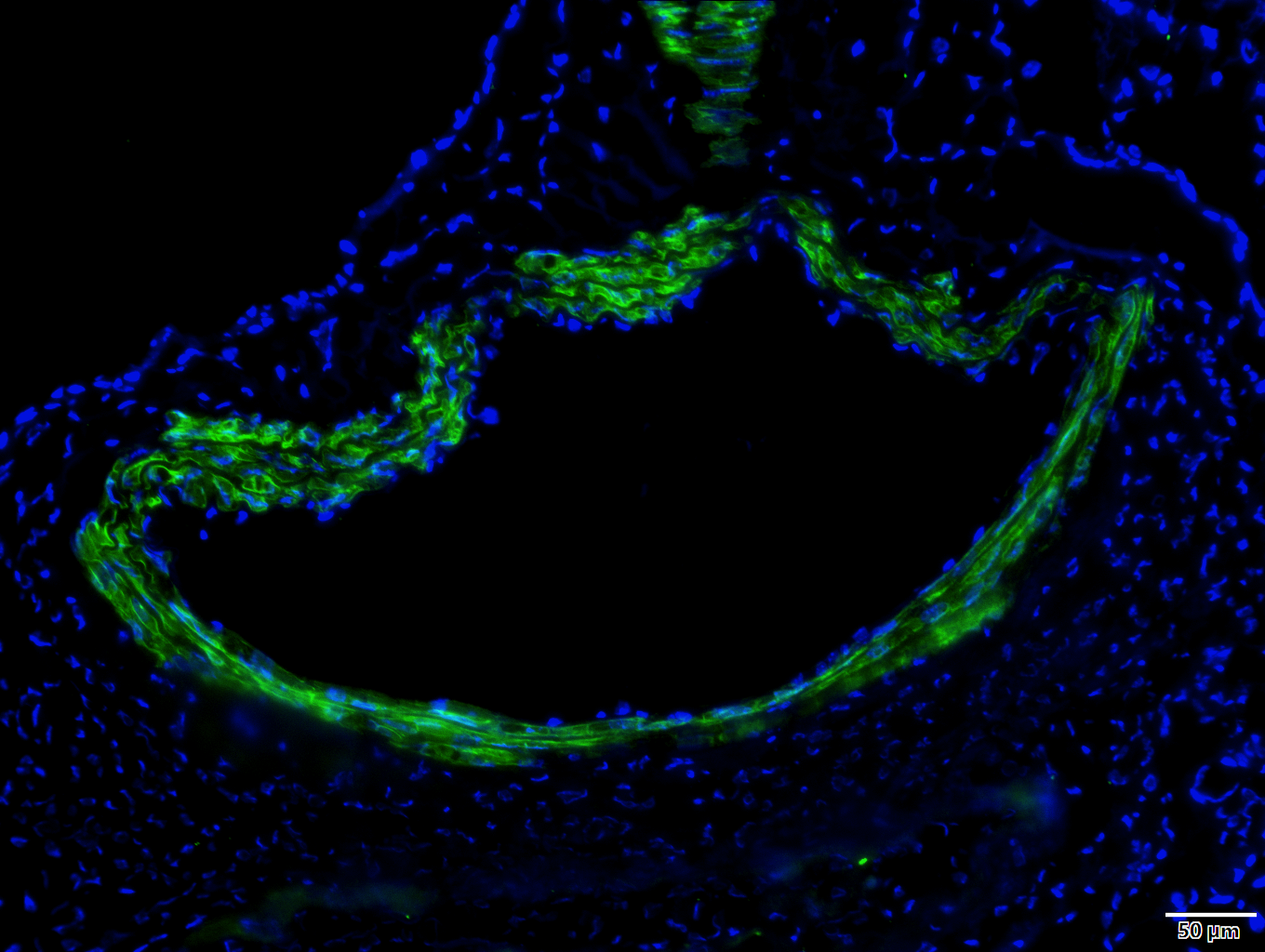

Supplement: Supplementary file 7 — Source data Fig. 6 [file 44321_2025_318_MOESM7_ESM.zip › Figure 6/Figure 6G/AAV-Adipoq-cre Saline.tif]

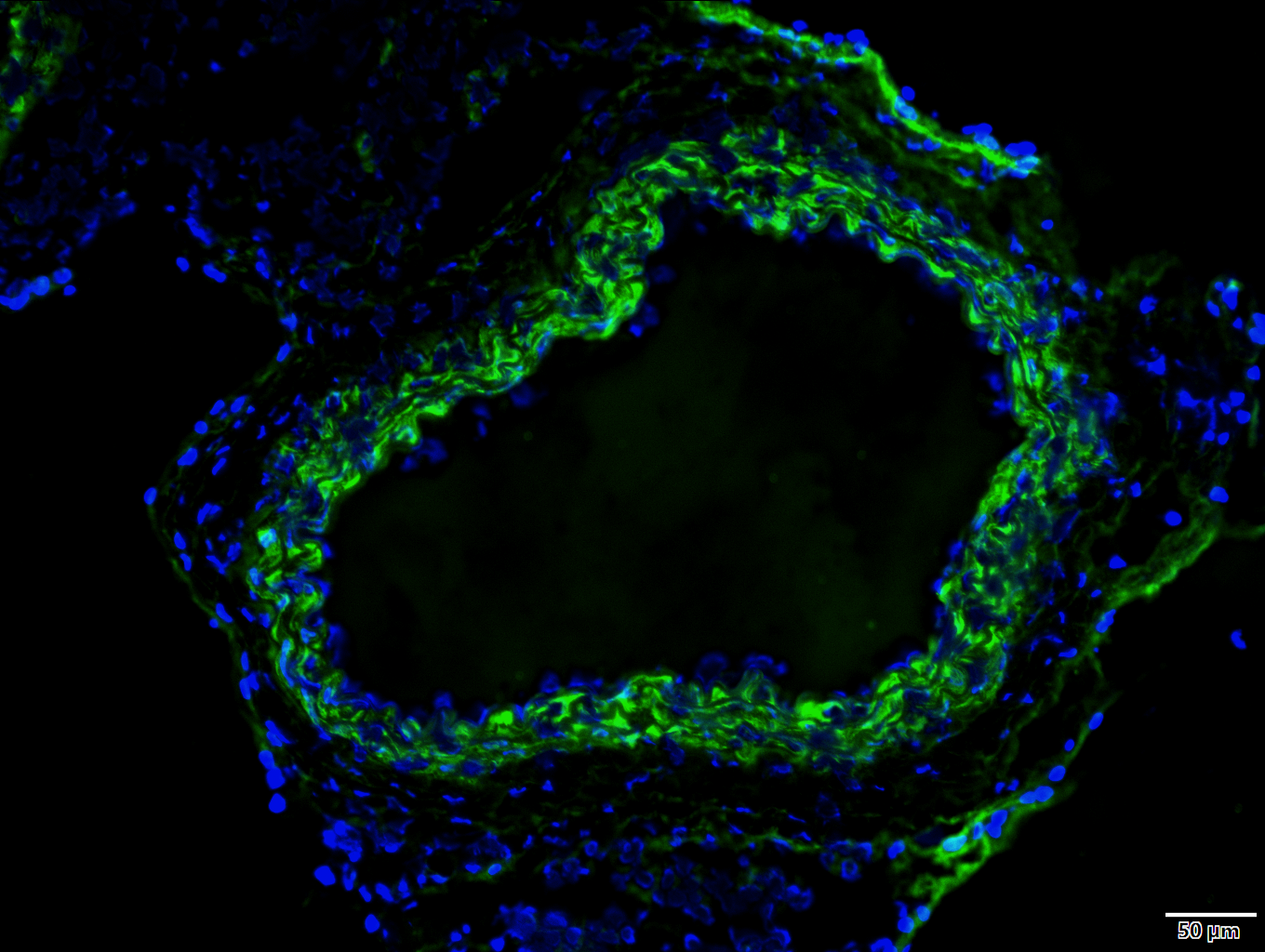

Supplement: Supplementary file 7 — Source data Fig. 6 [file 44321_2025_318_MOESM7_ESM.zip › Figure 6/Figure 6G/AAV-Control CL316,243.tif]

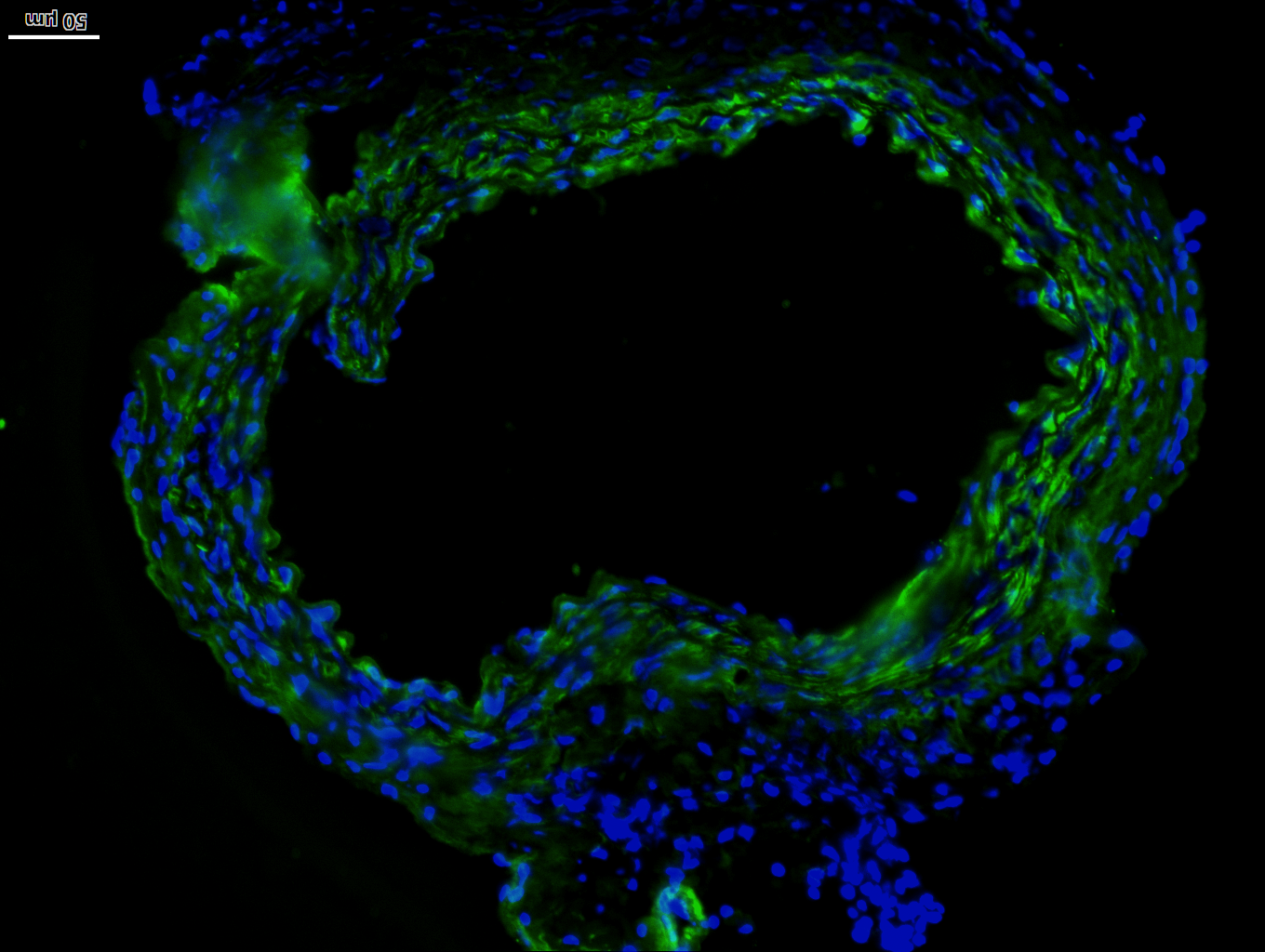

Supplement: Supplementary file 7 — Source data Fig. 6 [file 44321_2025_318_MOESM7_ESM.zip › Figure 6/Figure 6G/AAV-Control Saline.tif]

## Slide 1
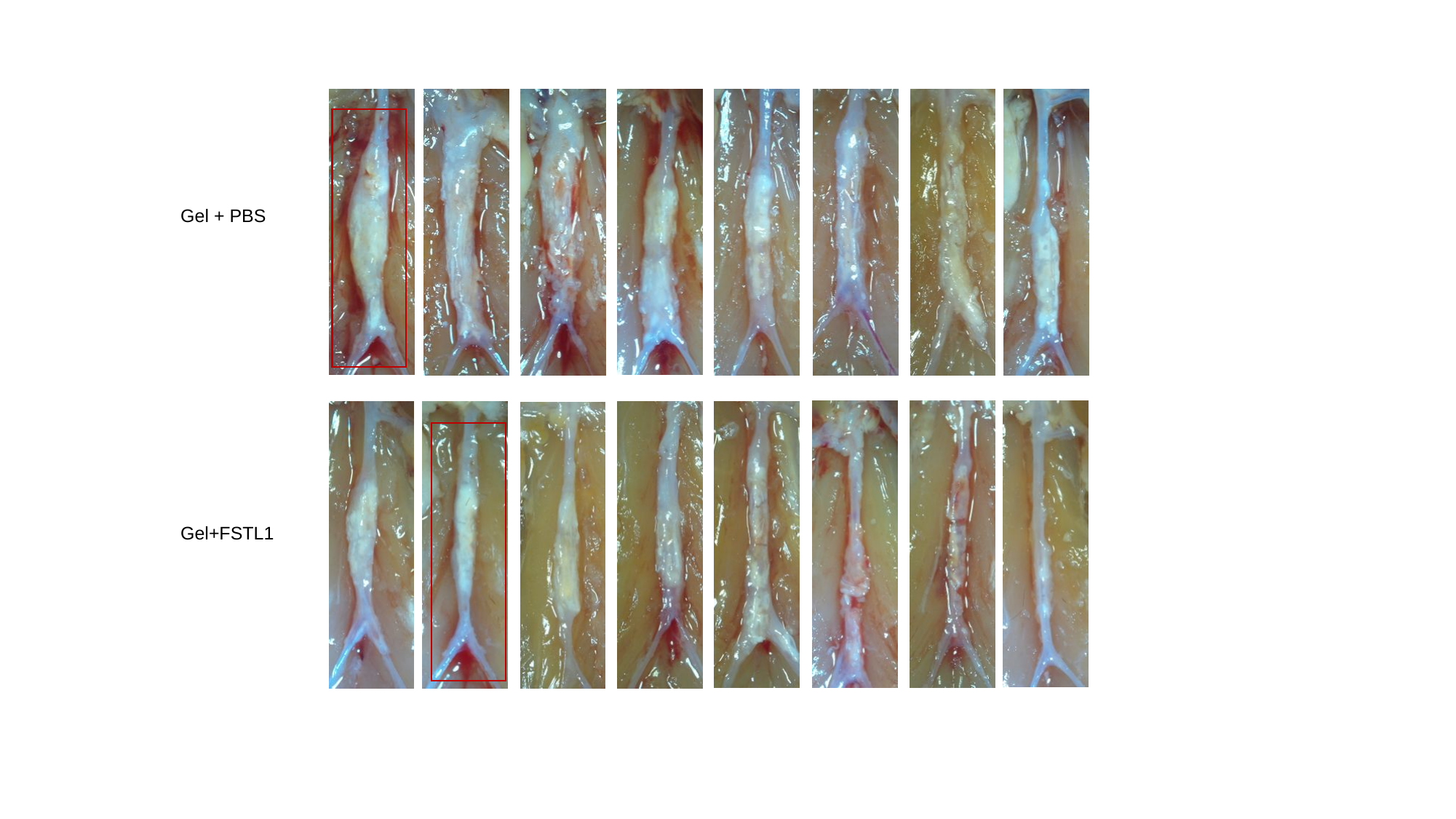

Gel + PBS
Gel+FSTL1

Supplement: Supplementary file 8 — Source data Fig. 7 [file 44321_2025_318_MOESM8_ESM.zip › Figure 7/Figure 7B/Whole mount.pptx]

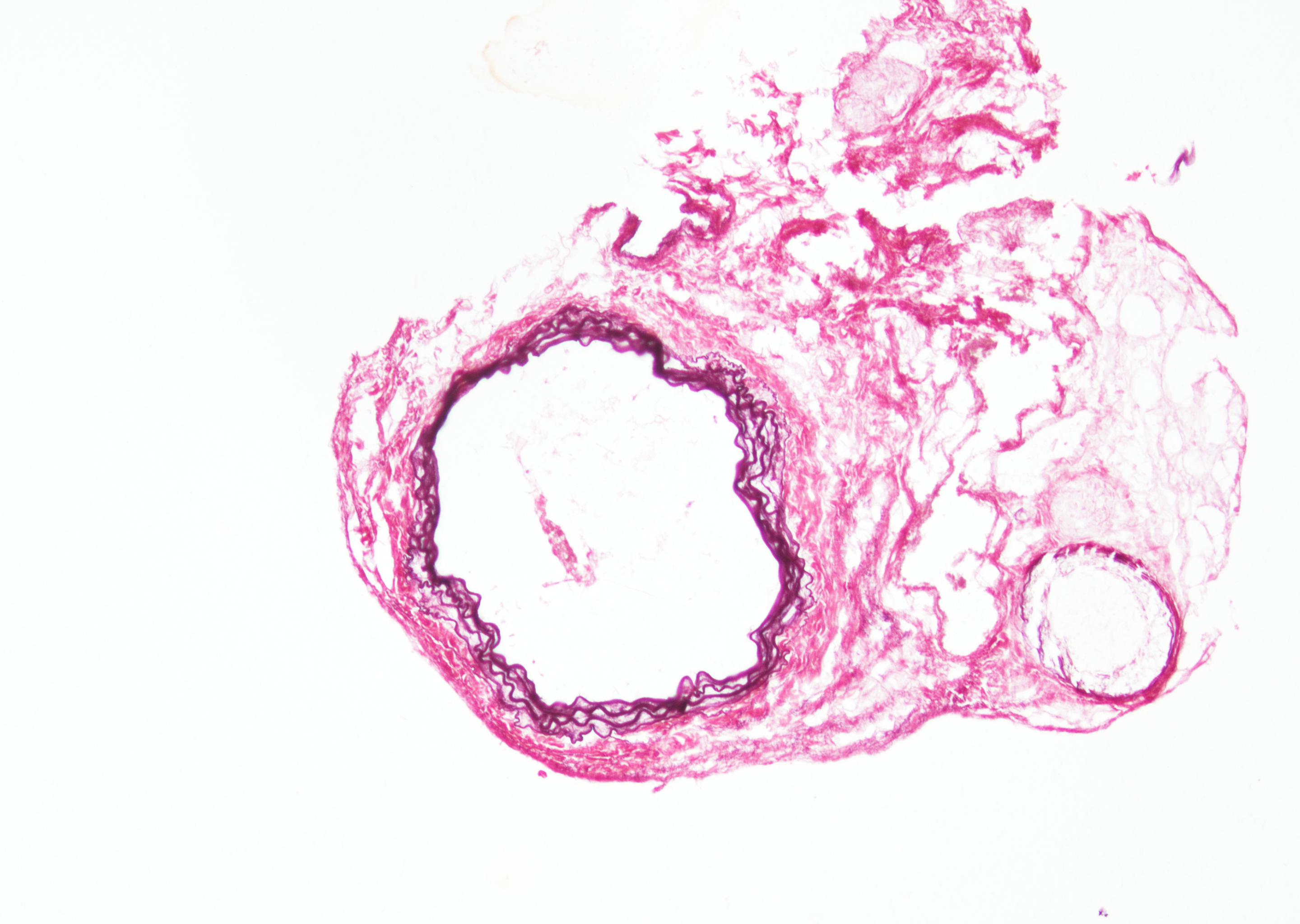

Supplement: Supplementary file 8 — Source data Fig. 7 [file 44321_2025_318_MOESM8_ESM.zip › Figure 7/Figure 7E/EVG Staining/Gel + FSTL1 100um.tiff]

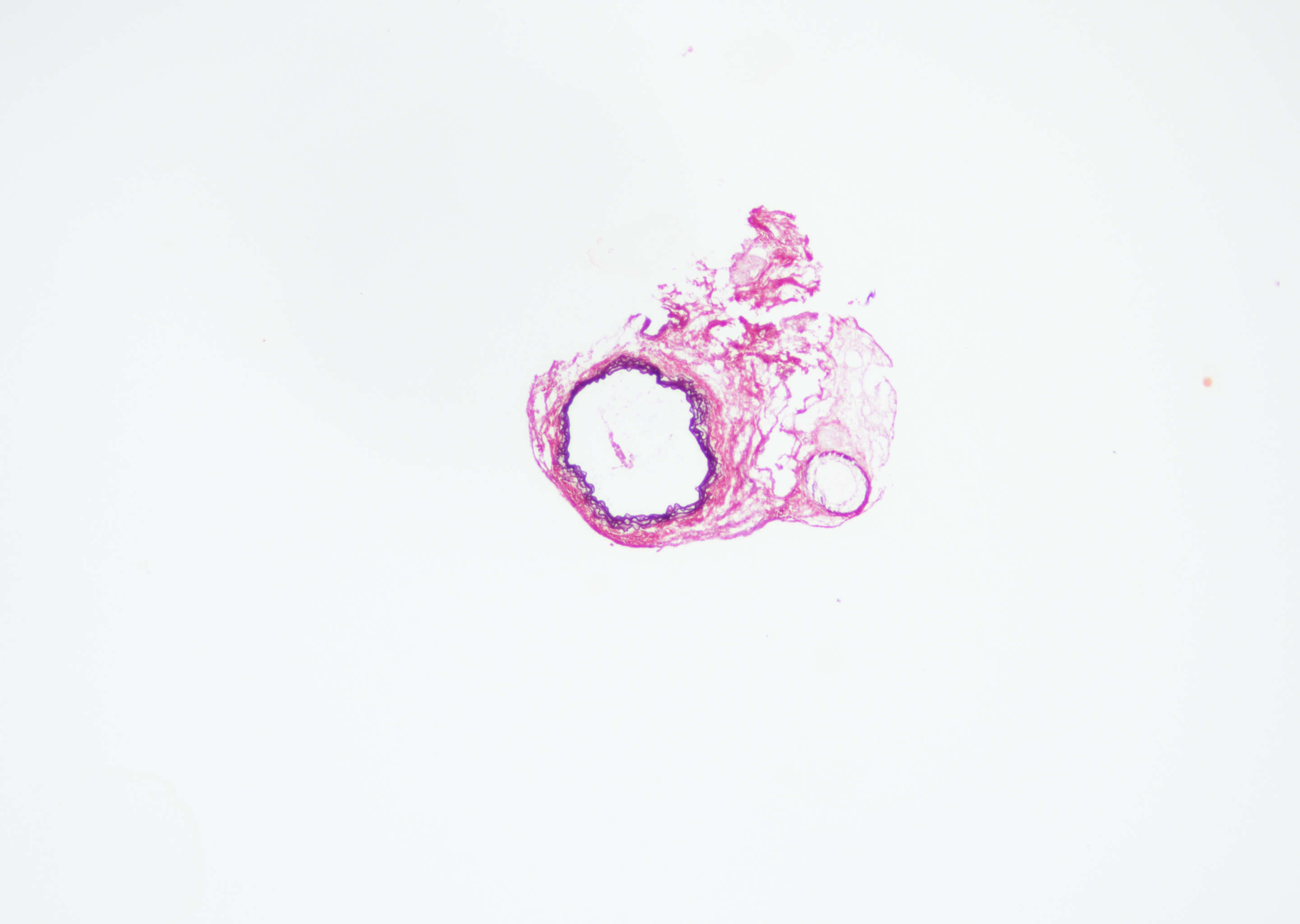

Supplement: Supplementary file 8 — Source data Fig. 7 [file 44321_2025_318_MOESM8_ESM.zip › Figure 7/Figure 7E/EVG Staining/Gel + FSTL1 200um.tiff]

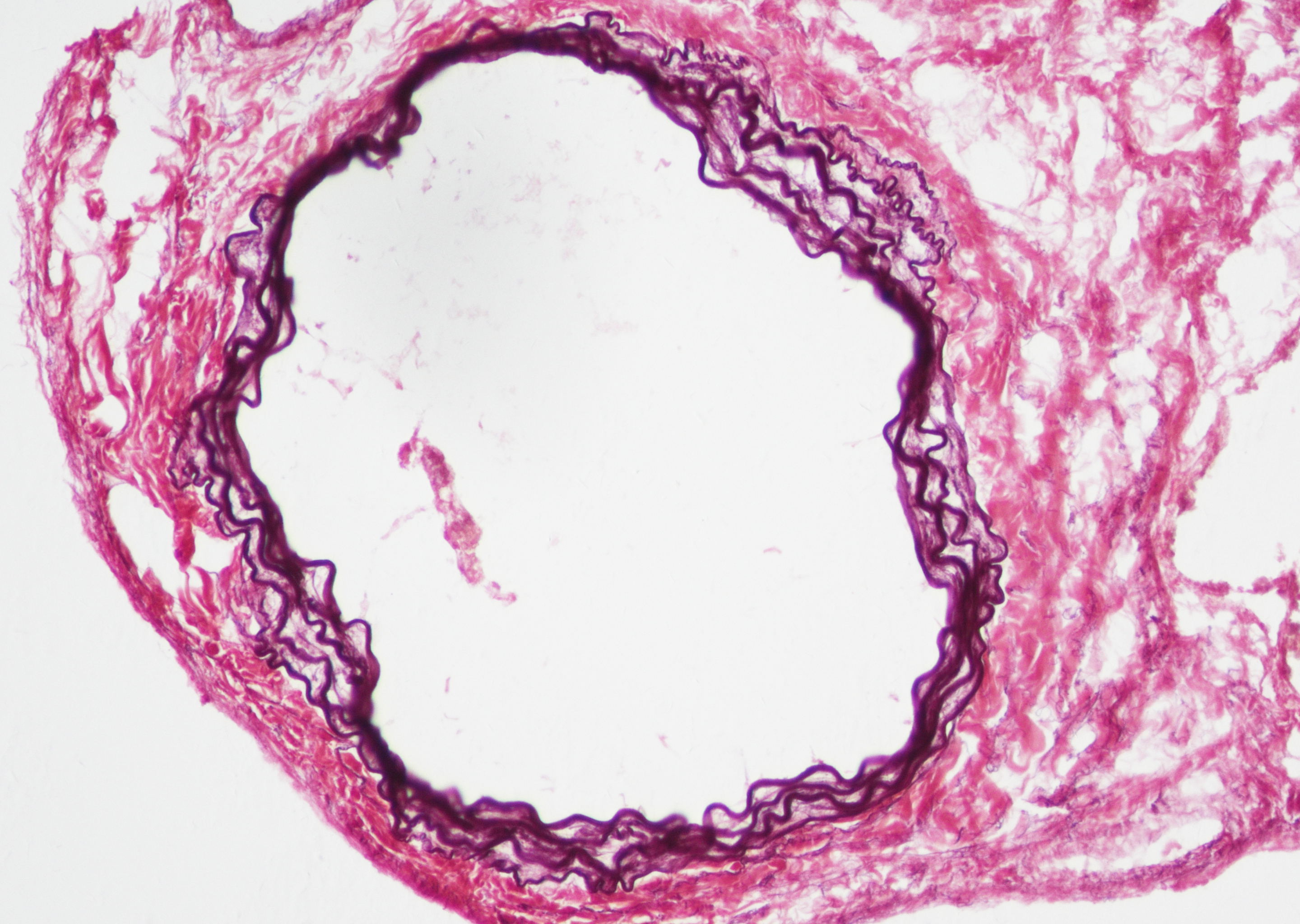

Supplement: Supplementary file 8 — Source data Fig. 7 [file 44321_2025_318_MOESM8_ESM.zip › Figure 7/Figure 7E/EVG Staining/Gel + FSTL1 50um.tiff]

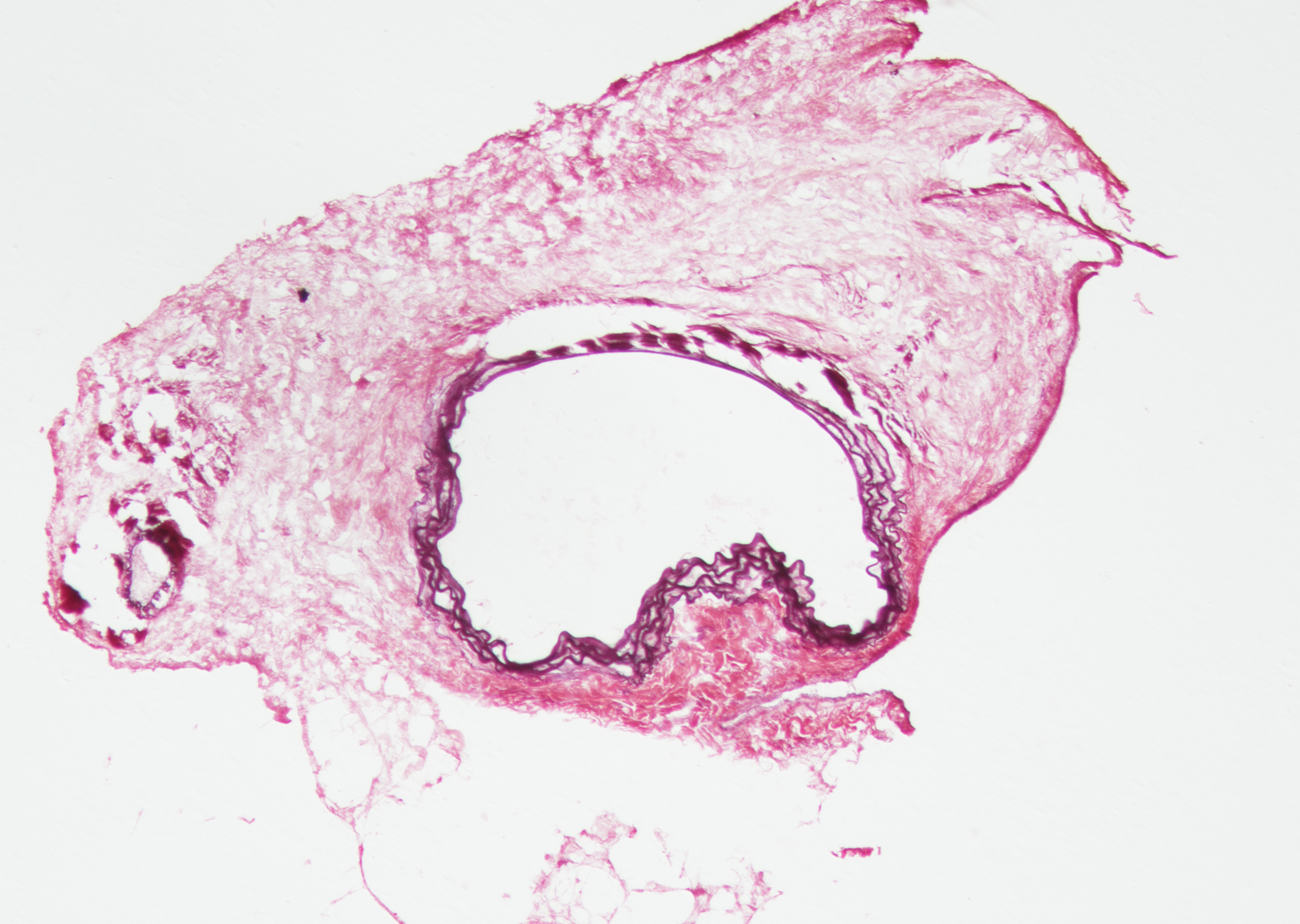

Supplement: Supplementary file 8 — Source data Fig. 7 [file 44321_2025_318_MOESM8_ESM.zip › Figure 7/Figure 7E/EVG Staining/Gel + PBS 100um.tiff]

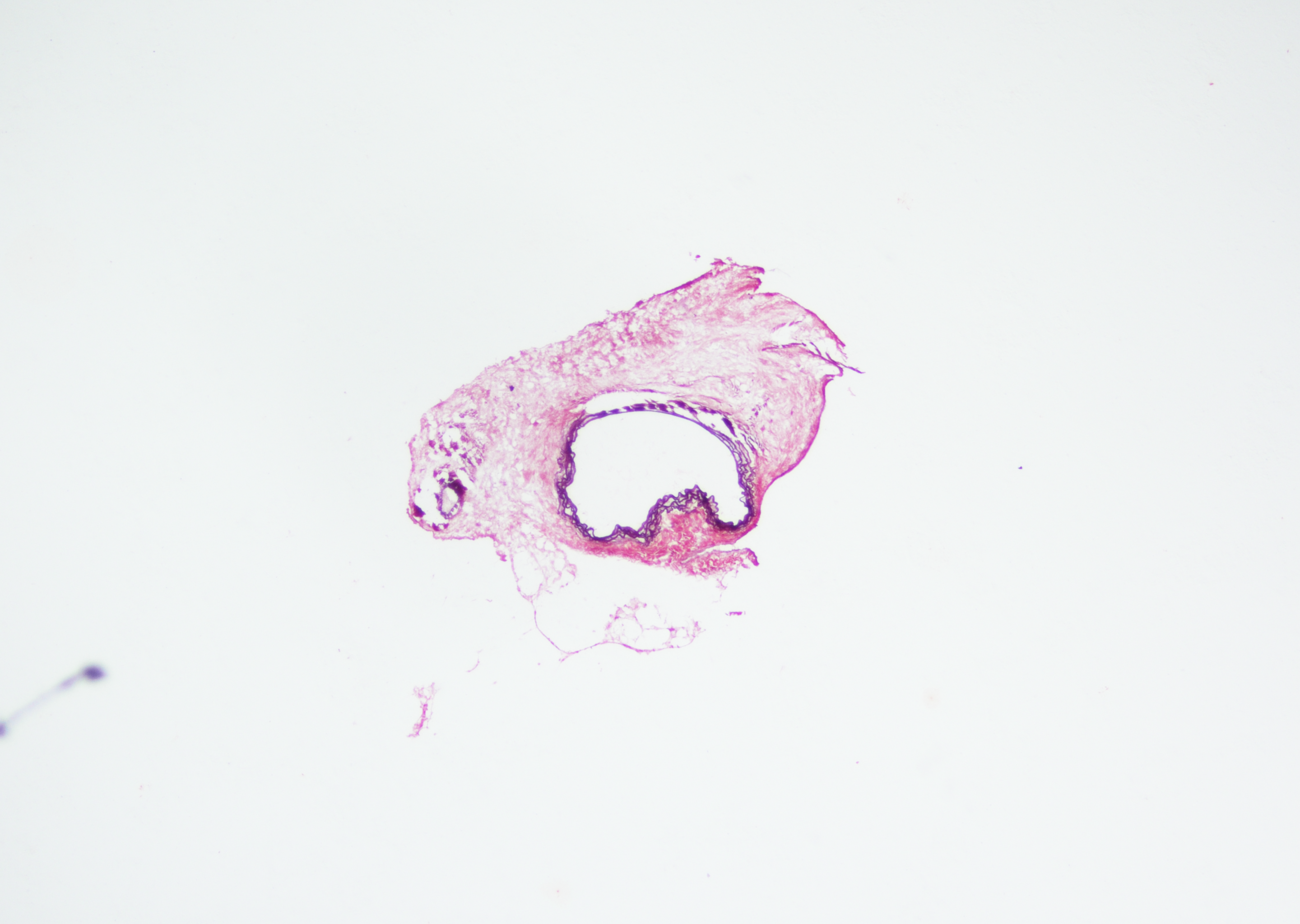

Supplement: Supplementary file 8 — Source data Fig. 7 [file 44321_2025_318_MOESM8_ESM.zip › Figure 7/Figure 7E/EVG Staining/Gel + PBS 200um.tiff]

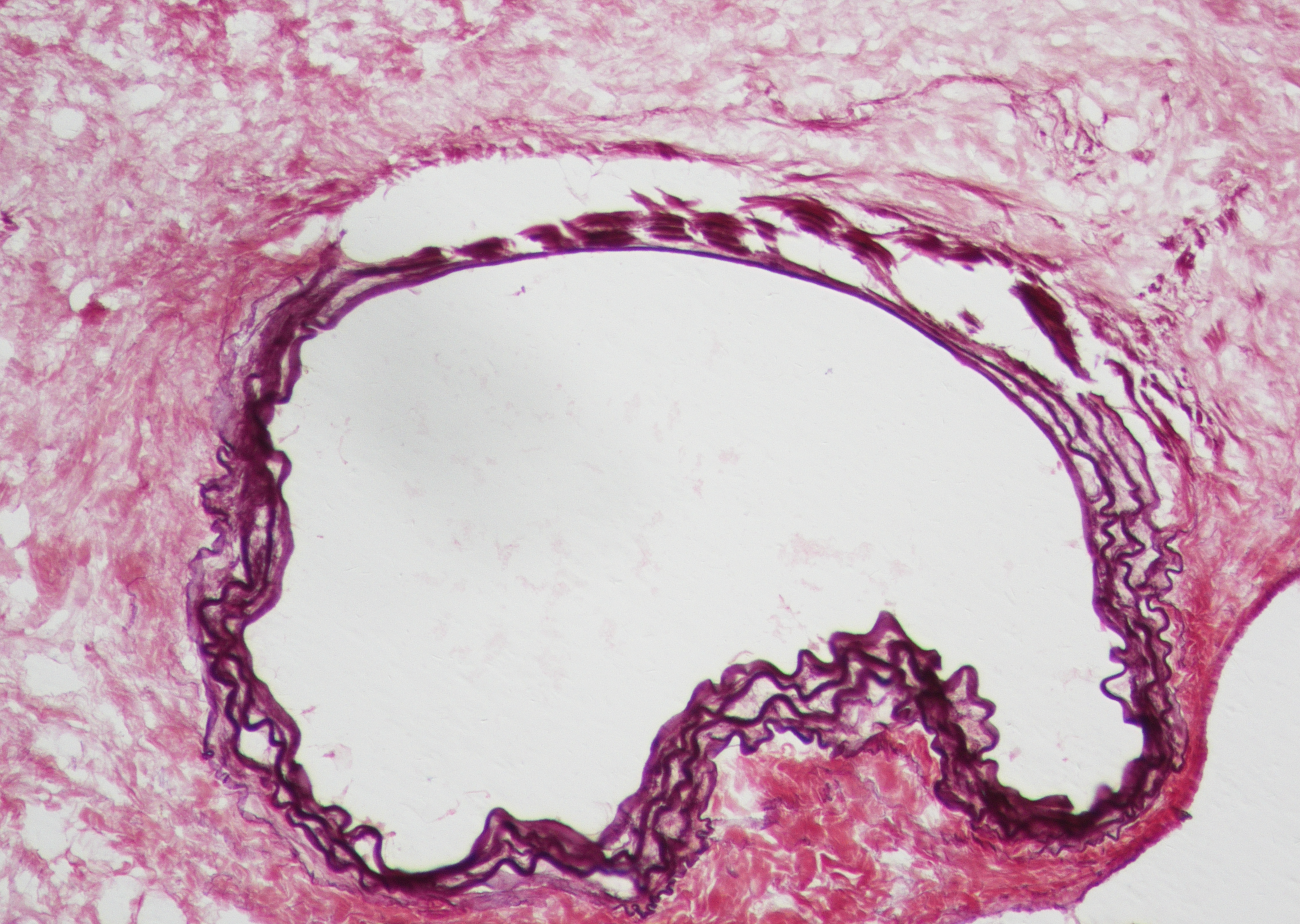

Supplement: Supplementary file 8 — Source data Fig. 7 [file 44321_2025_318_MOESM8_ESM.zip › Figure 7/Figure 7E/EVG Staining/Gel + PBS 50um.tiff]

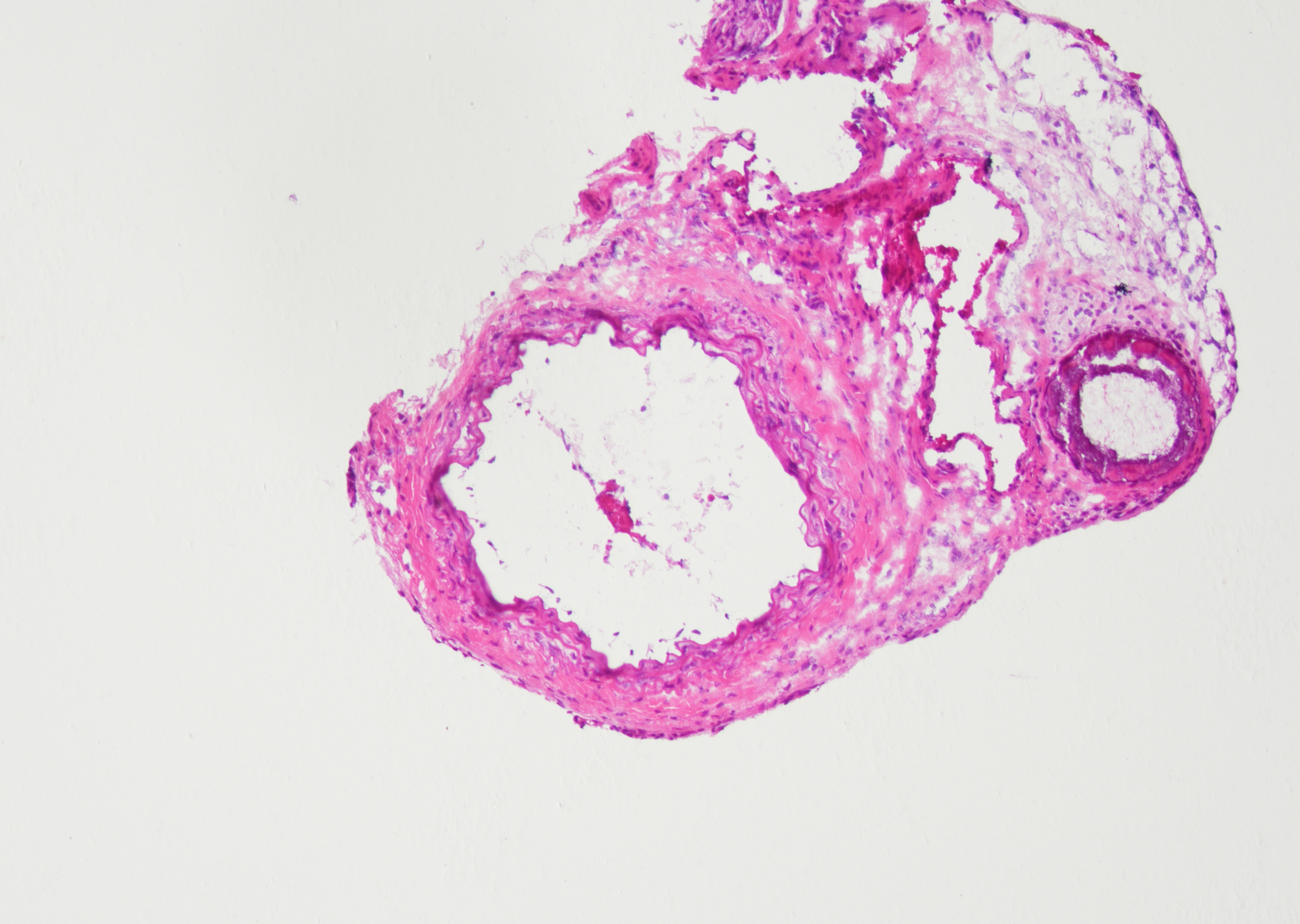

Supplement: Supplementary file 8 — Source data Fig. 7 [file 44321_2025_318_MOESM8_ESM.zip › Figure 7/Figure 7E/HE Staining/Gel+FSTL1 100um.tiff]

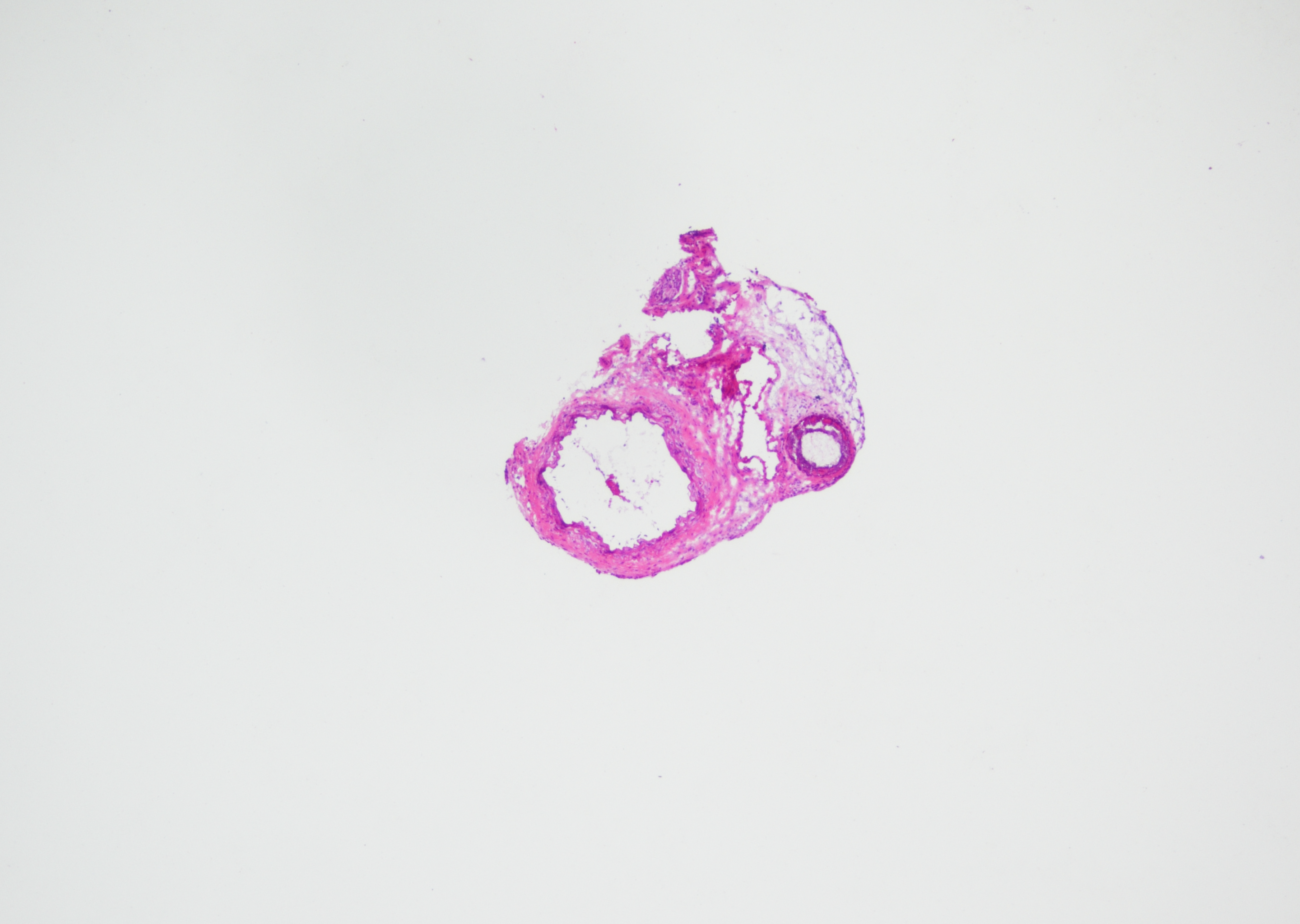

Supplement: Supplementary file 8 — Source data Fig. 7 [file 44321_2025_318_MOESM8_ESM.zip › Figure 7/Figure 7E/HE Staining/Gel+FSTL1 200um.tiff]

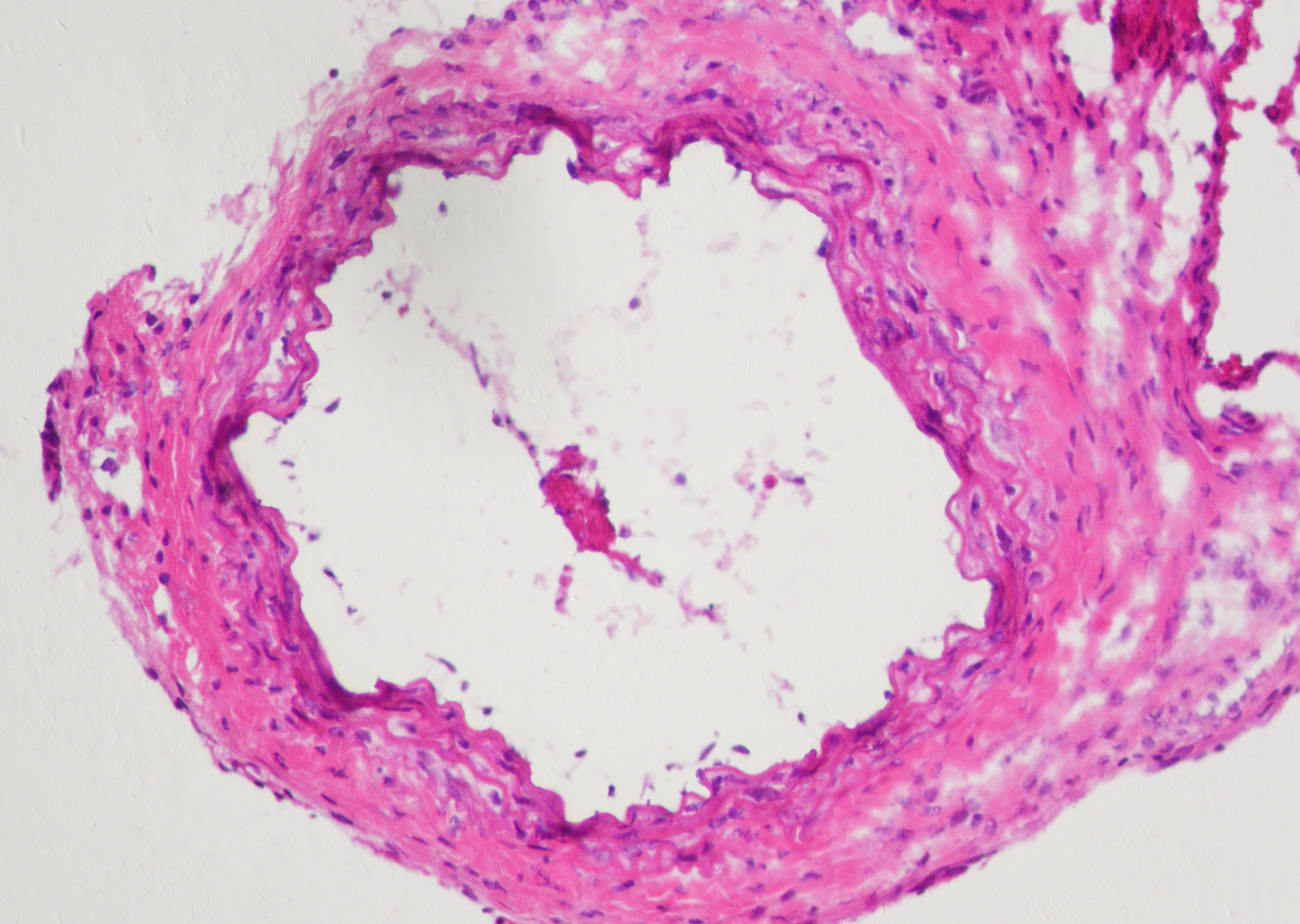

Supplement: Supplementary file 8 — Source data Fig. 7 [file 44321_2025_318_MOESM8_ESM.zip › Figure 7/Figure 7E/HE Staining/Gel+FSTL1 50um.tiff]

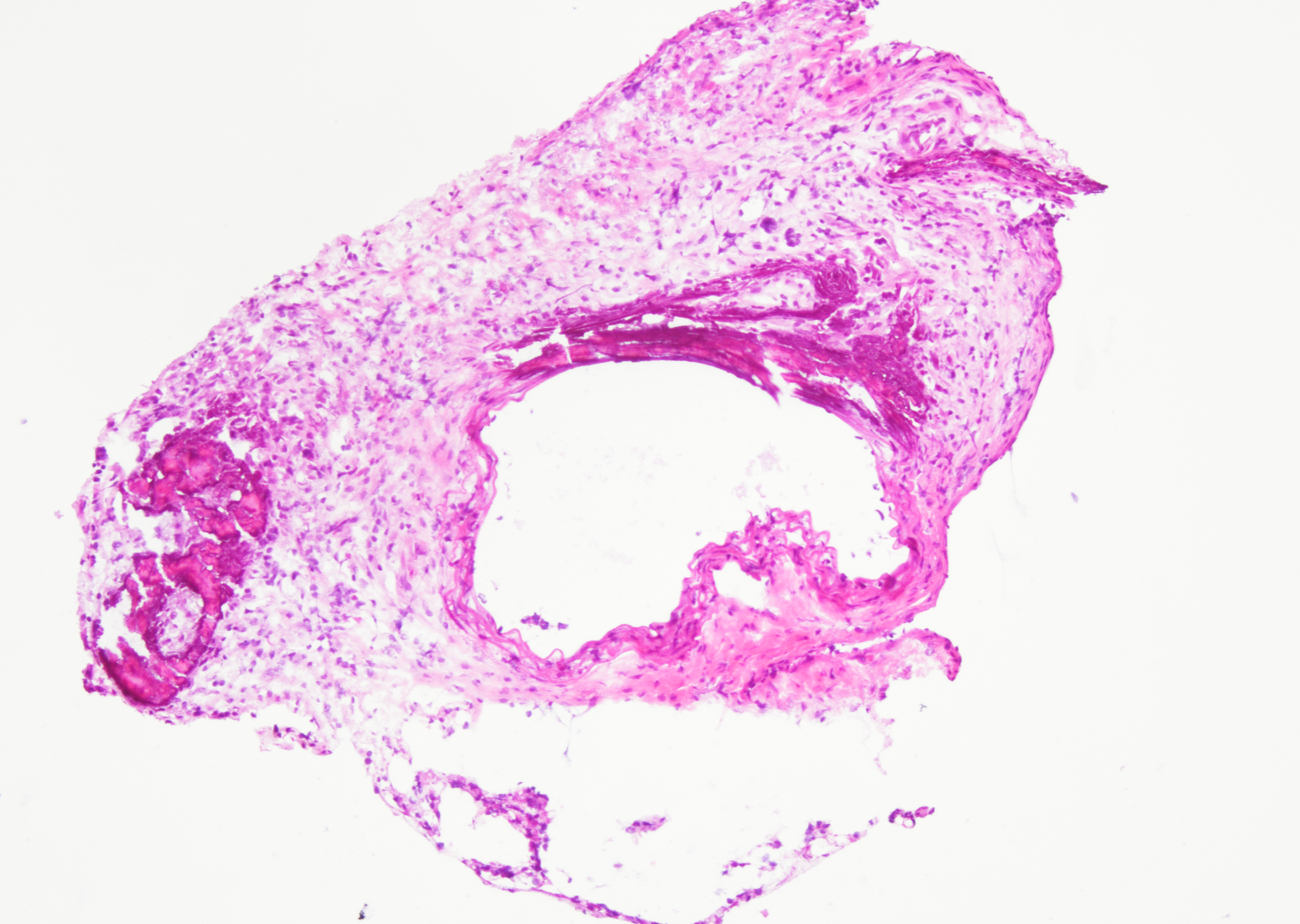

Supplement: Supplementary file 8 — Source data Fig. 7 [file 44321_2025_318_MOESM8_ESM.zip › Figure 7/Figure 7E/HE Staining/Gel+PBS 100um.tiff]

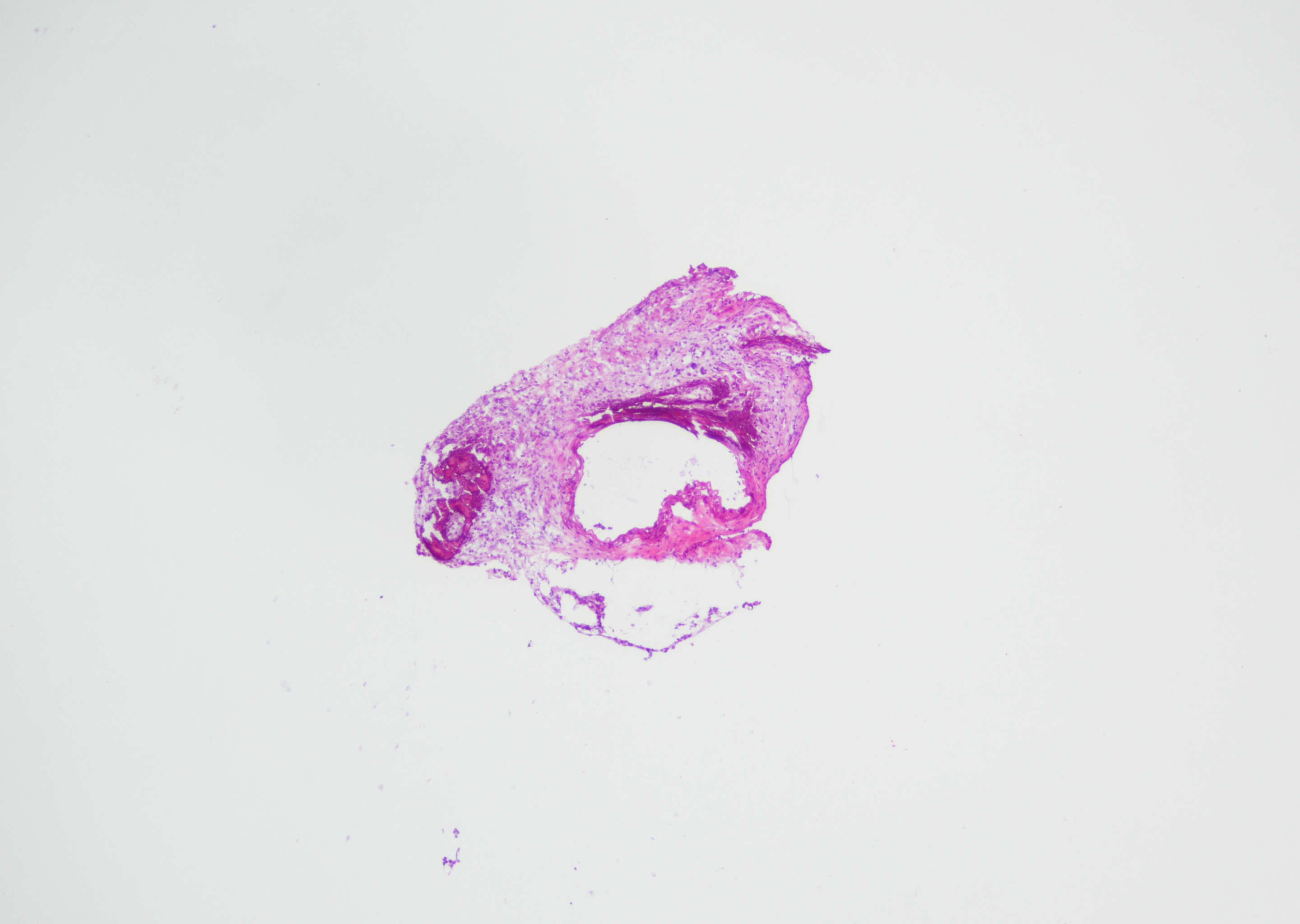

Supplement: Supplementary file 8 — Source data Fig. 7 [file 44321_2025_318_MOESM8_ESM.zip › Figure 7/Figure 7E/HE Staining/Gel+PBS 200um.tiff]

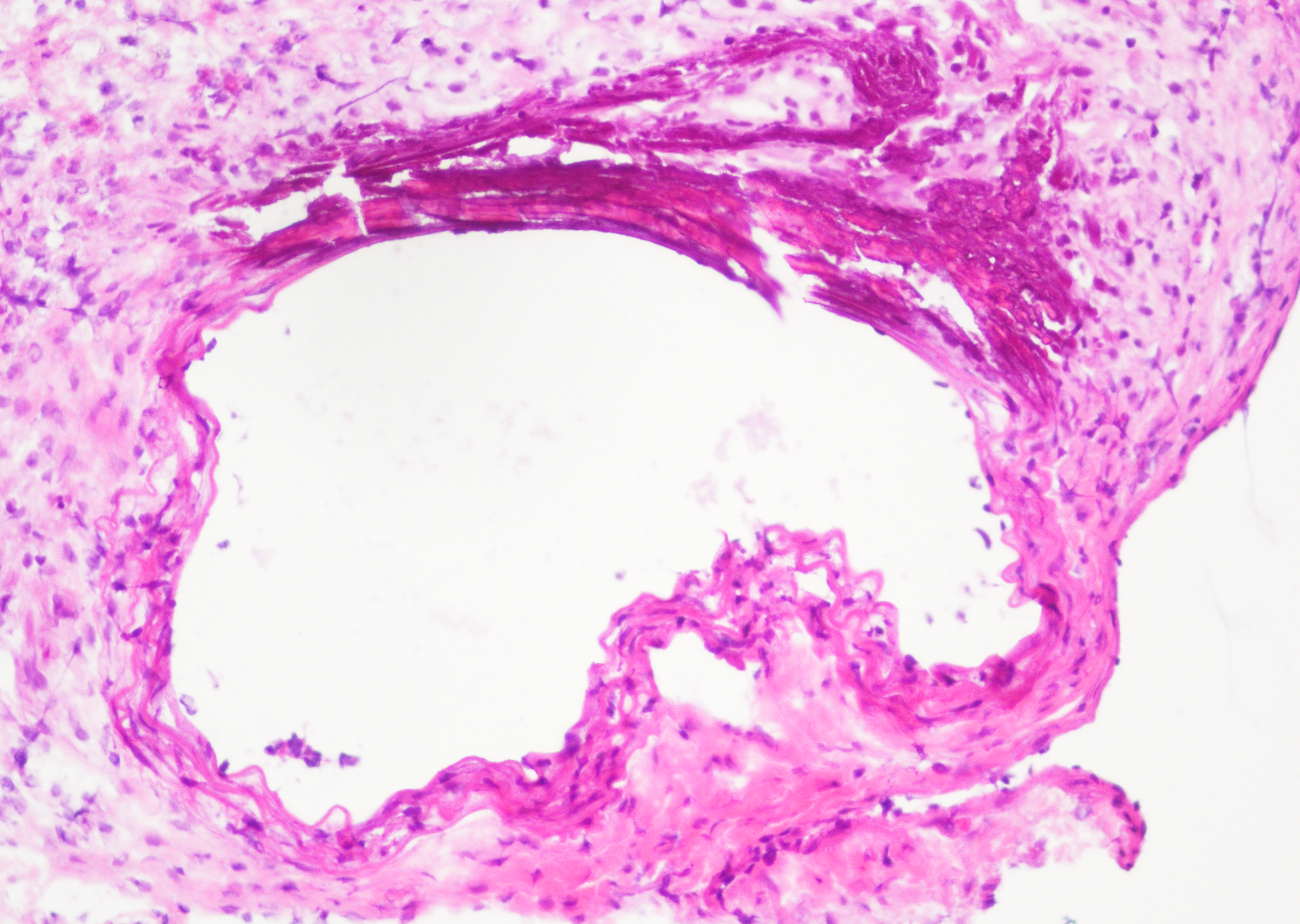

Supplement: Supplementary file 8 — Source data Fig. 7 [file 44321_2025_318_MOESM8_ESM.zip › Figure 7/Figure 7E/HE Staining/Gel+PBS 50um.tiff]

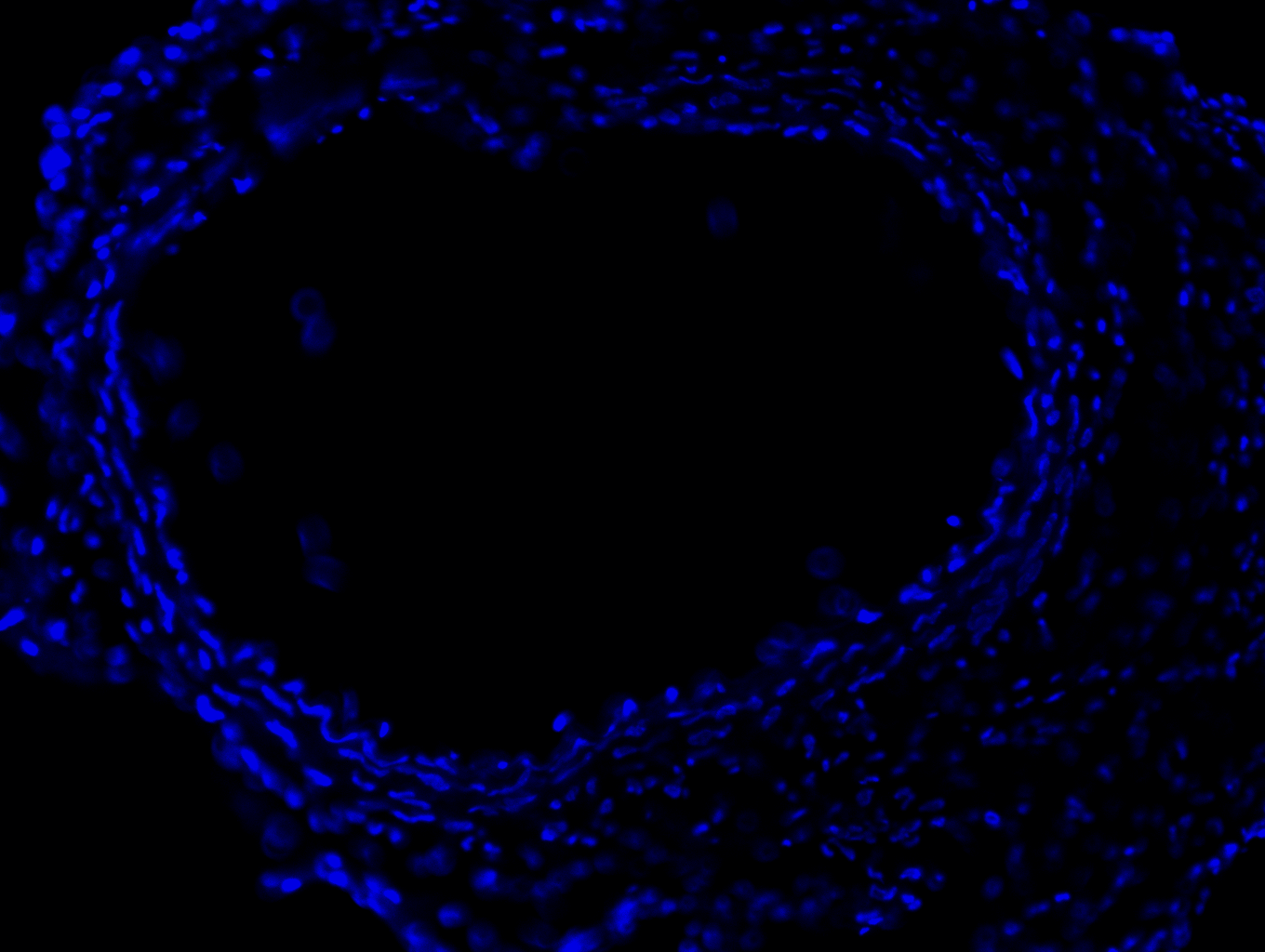

Supplement: Supplementary file 8 — Source data Fig. 7 [file 44321_2025_318_MOESM8_ESM.zip › Figure 7/Figure 7G/Gel +PBS DAPI.tif]

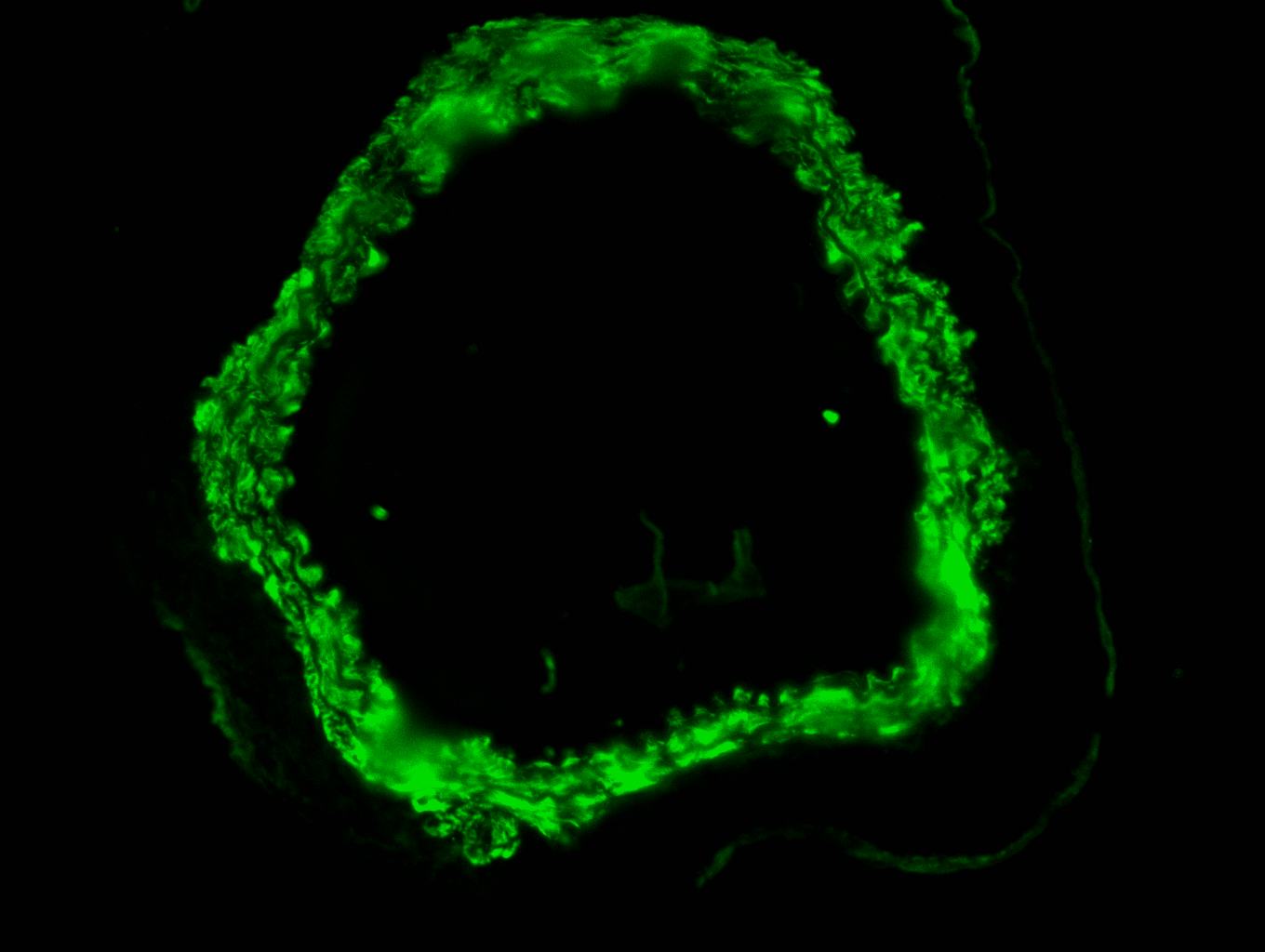

Supplement: Supplementary file 8 — Source data Fig. 7 [file 44321_2025_318_MOESM8_ESM.zip › Figure 7/Figure 7G/Gel+FSTL1 a-SMA.tif]

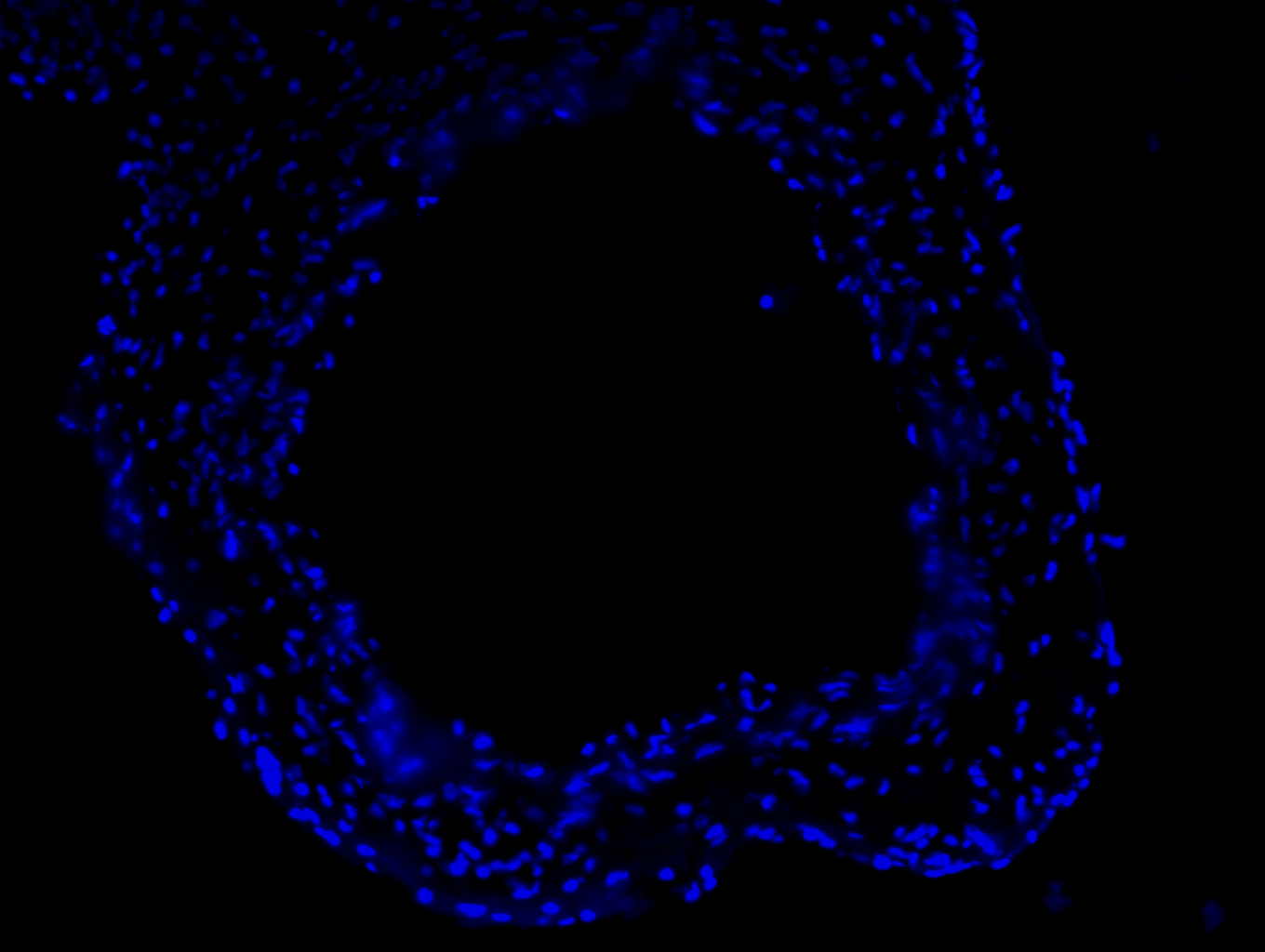

Supplement: Supplementary file 8 — Source data Fig. 7 [file 44321_2025_318_MOESM8_ESM.zip › Figure 7/Figure 7G/Gel+FSTL1 DAPI.tif]

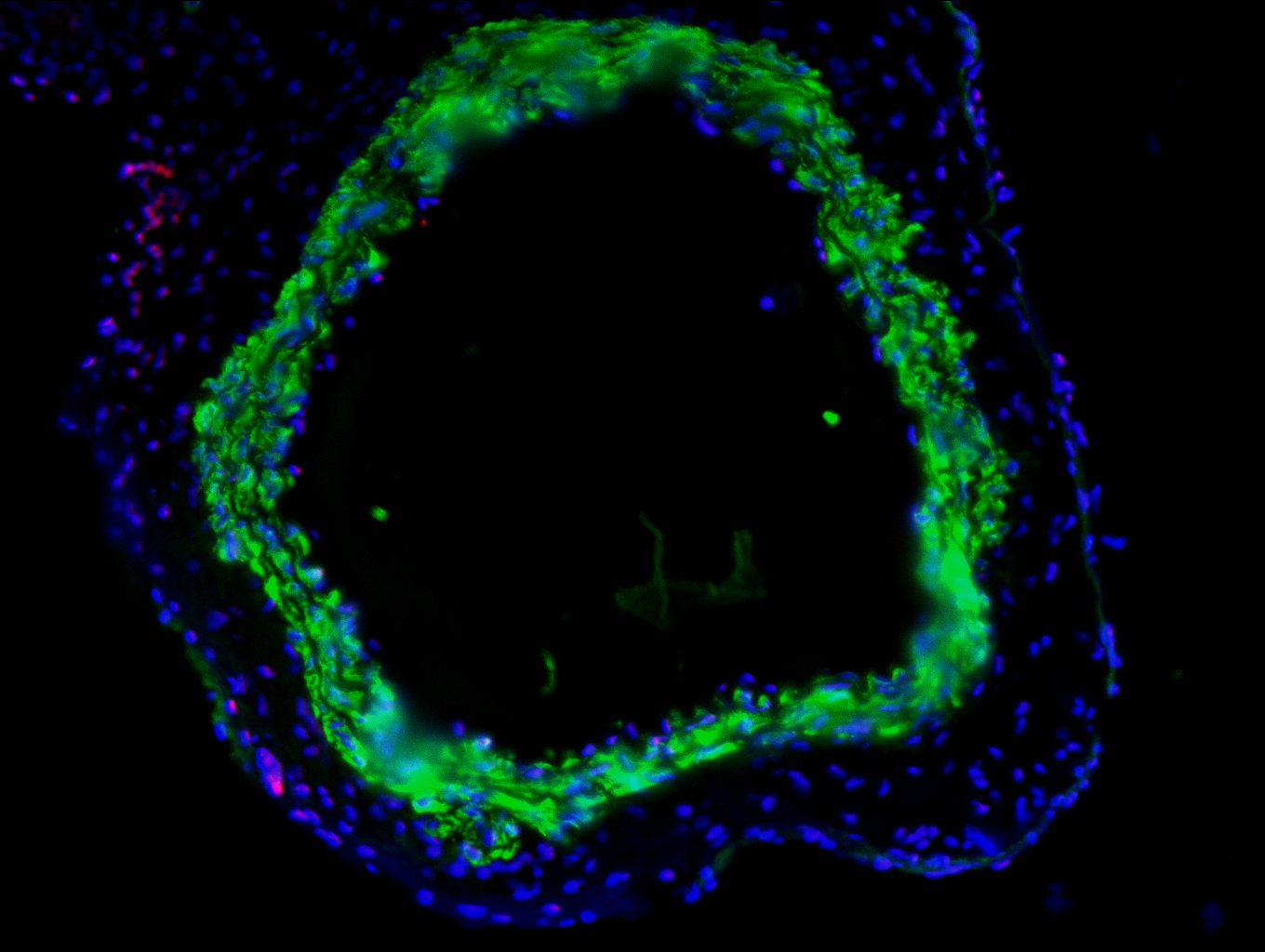

Supplement: Supplementary file 8 — Source data Fig. 7 [file 44321_2025_318_MOESM8_ESM.zip › Figure 7/Figure 7G/Gel+FSTL1 Merge.tif]

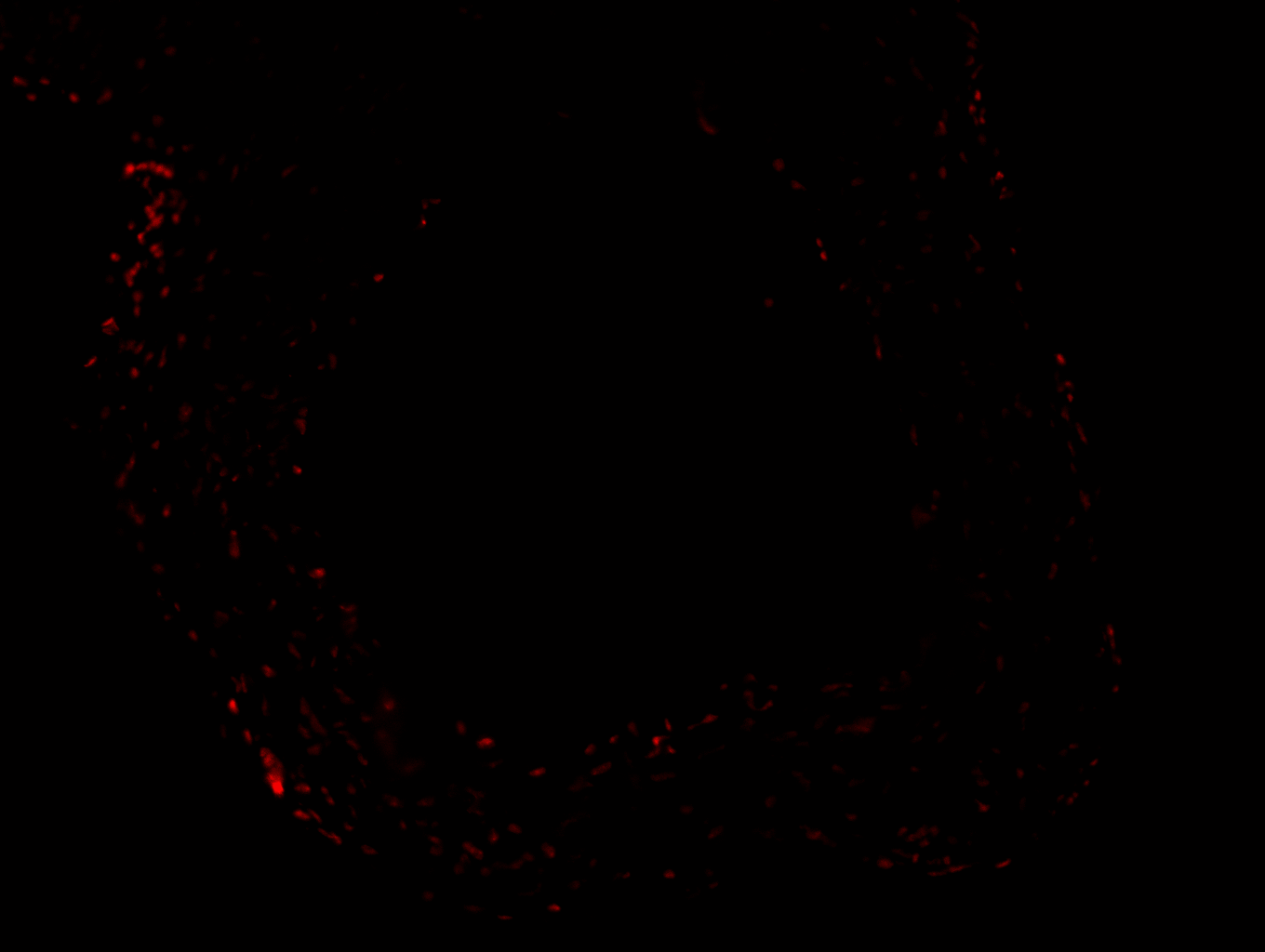

Supplement: Supplementary file 8 — Source data Fig. 7 [file 44321_2025_318_MOESM8_ESM.zip › Figure 7/Figure 7G/Gel+FSTL1 TUNEL.tif]

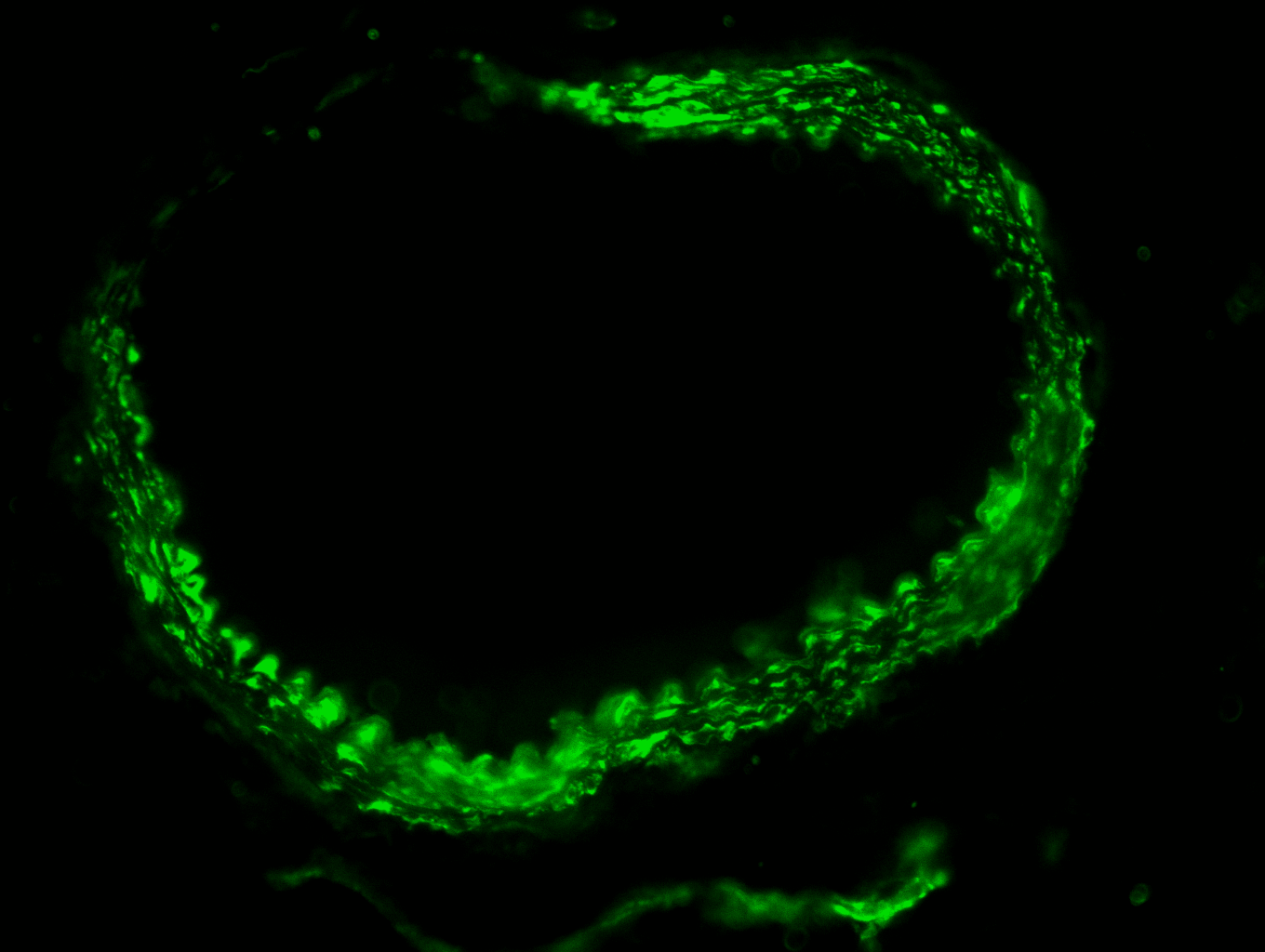

Supplement: Supplementary file 8 — Source data Fig. 7 [file 44321_2025_318_MOESM8_ESM.zip › Figure 7/Figure 7G/Gel+PBS a-SMA.tif]

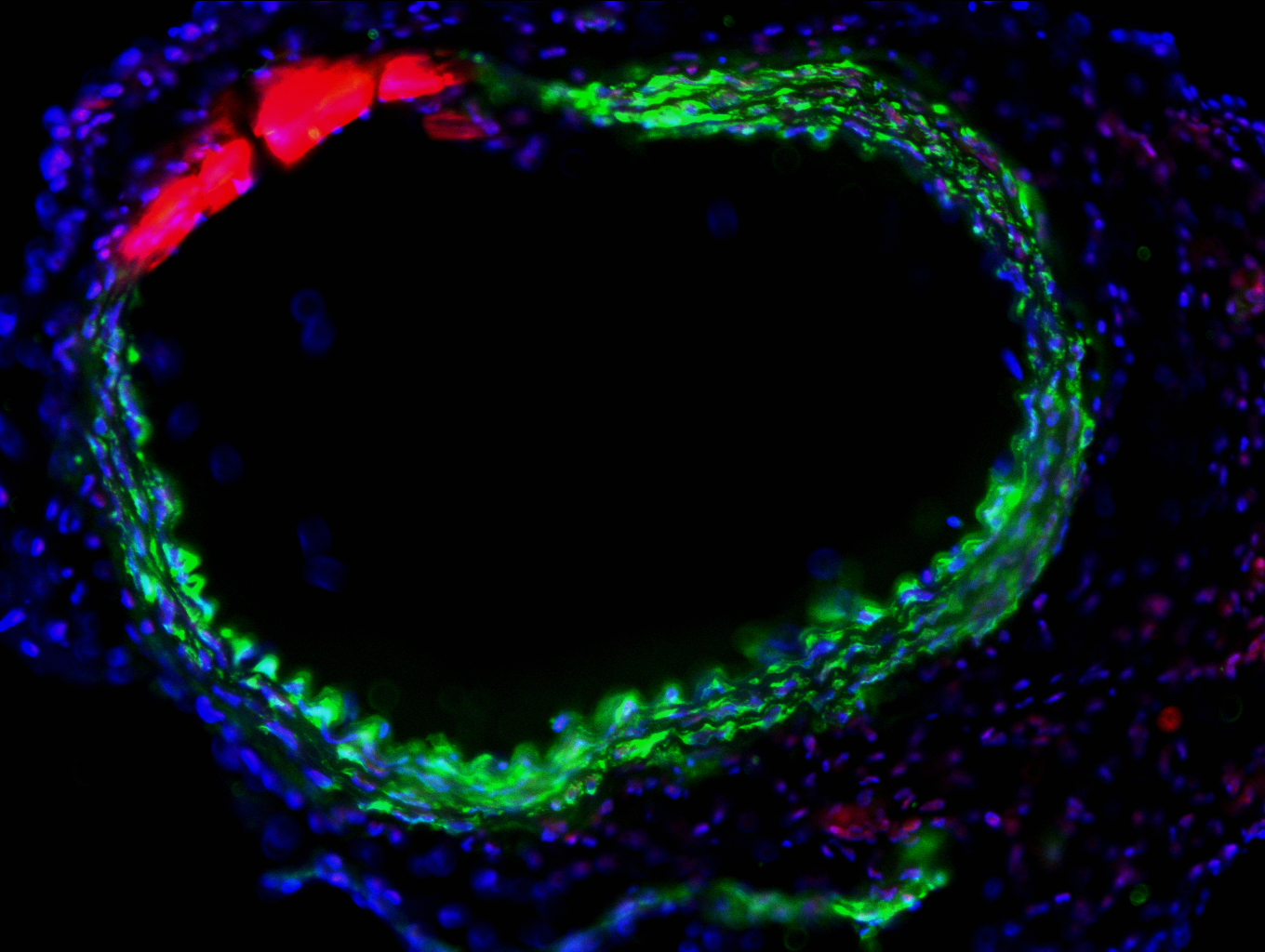

Supplement: Supplementary file 8 — Source data Fig. 7 [file 44321_2025_318_MOESM8_ESM.zip › Figure 7/Figure 7G/Gel+PBS Merge.tif]

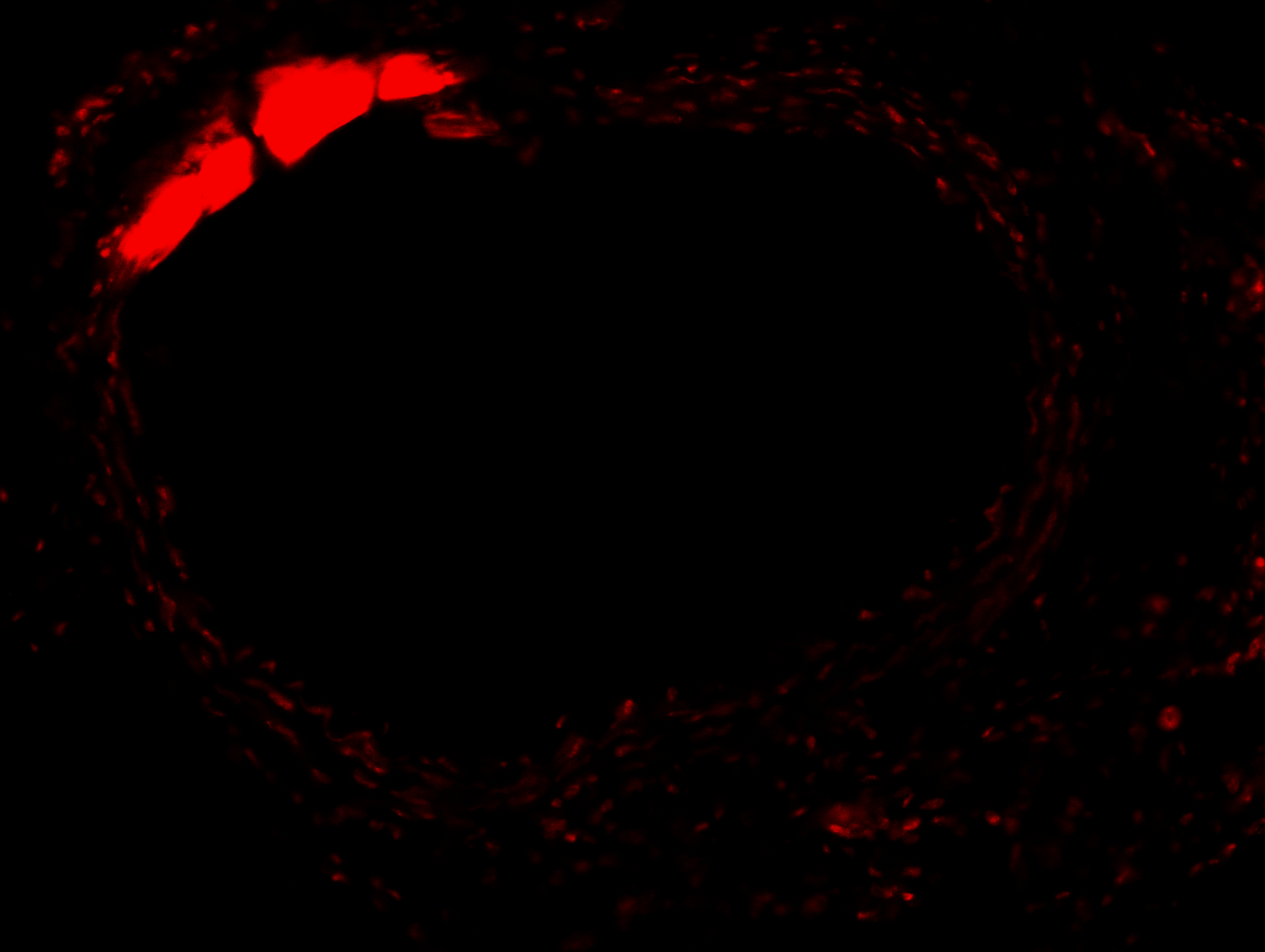

Supplement: Supplementary file 8 — Source data Fig. 7 [file 44321_2025_318_MOESM8_ESM.zip › Figure 7/Figure 7G/Gel+PBS TUNEL.tif]

## Slide 1
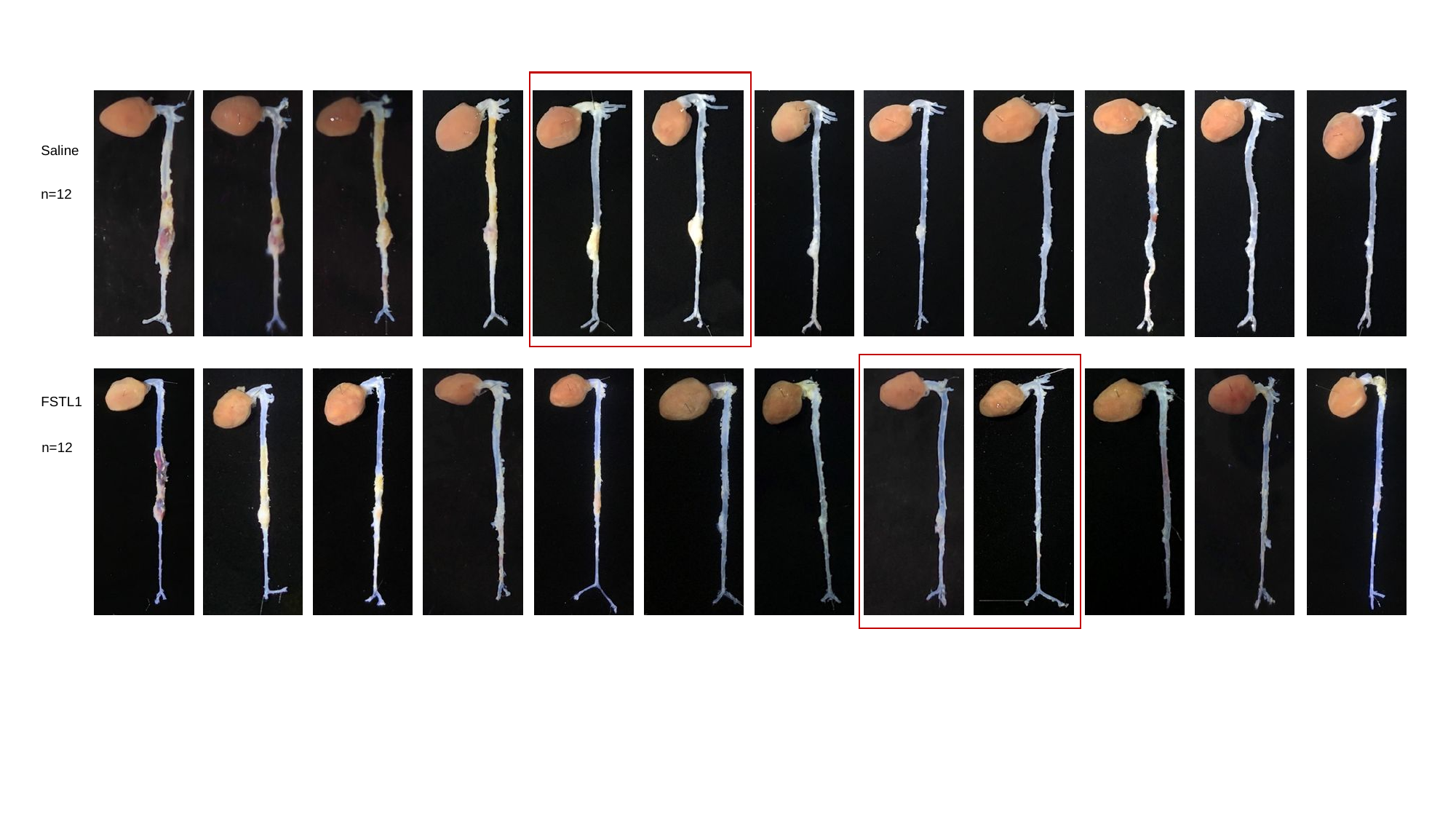

Saline
n=12
FSTL1
n=12

Supplement: Supplementary file 9 — Source data Fig. 8 [file 44321_2025_318_MOESM9_ESM.zip › Figure 8/Figure 8B/Whole mount.pptx]

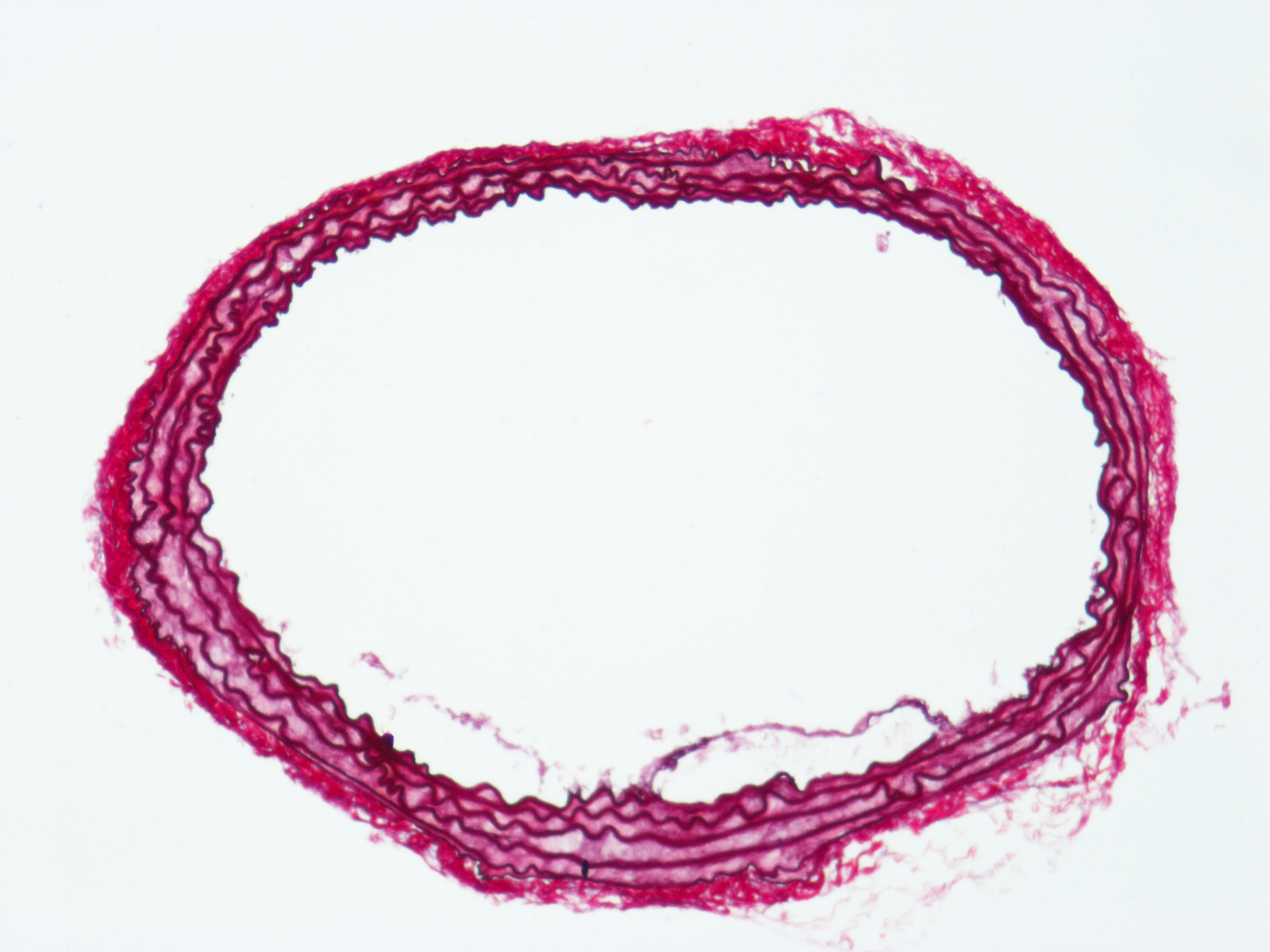

Supplement: Supplementary file 9 — Source data Fig. 8 [file 44321_2025_318_MOESM9_ESM.zip › Figure 8/Figure 8D/EVG Staining/FSTL1 100um.tif]

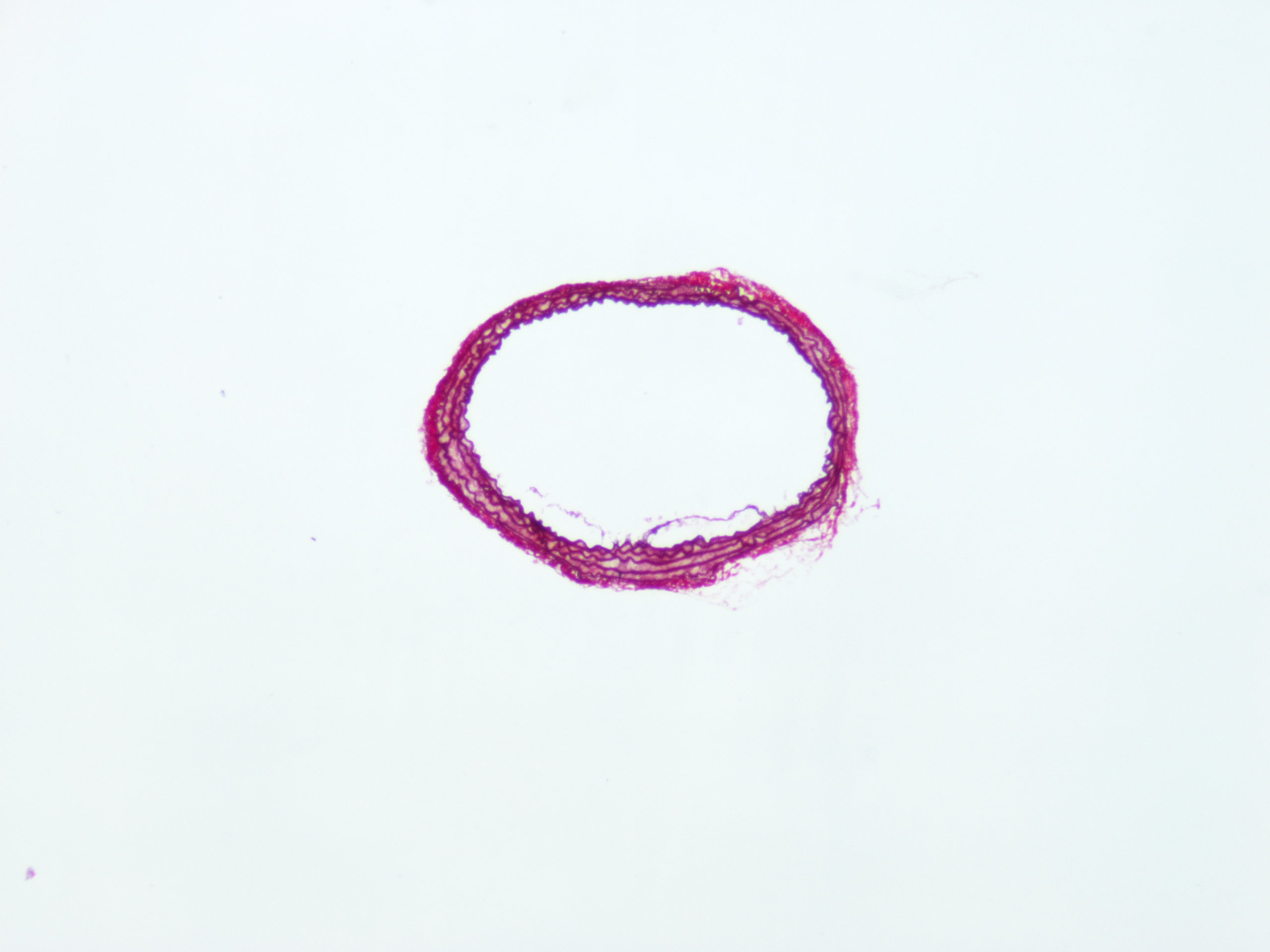

Supplement: Supplementary file 9 — Source data Fig. 8 [file 44321_2025_318_MOESM9_ESM.zip › Figure 8/Figure 8D/EVG Staining/FSTL1 200um.tif]

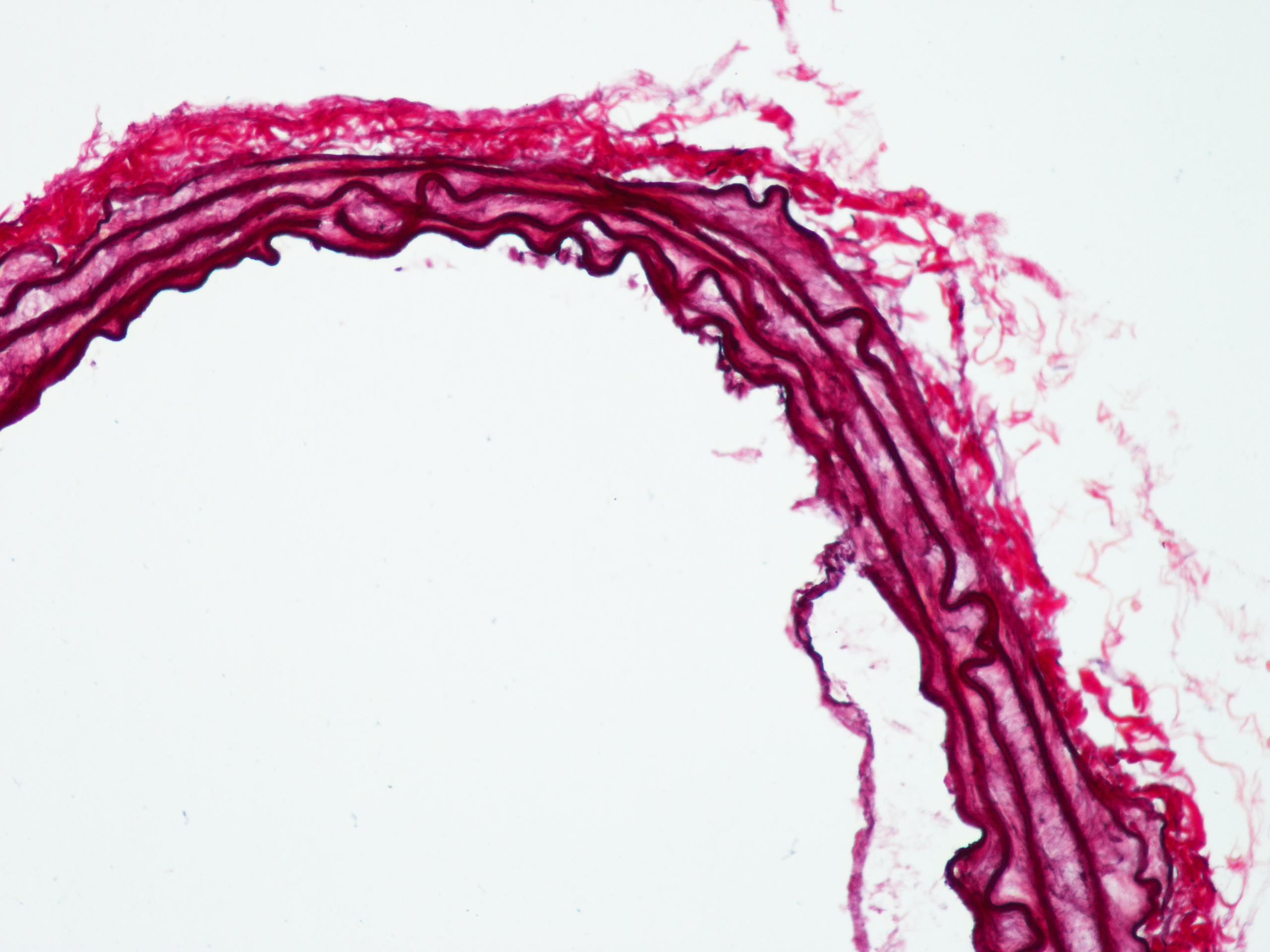

Supplement: Supplementary file 9 — Source data Fig. 8 [file 44321_2025_318_MOESM9_ESM.zip › Figure 8/Figure 8D/EVG Staining/FSTL1 50um.tif]

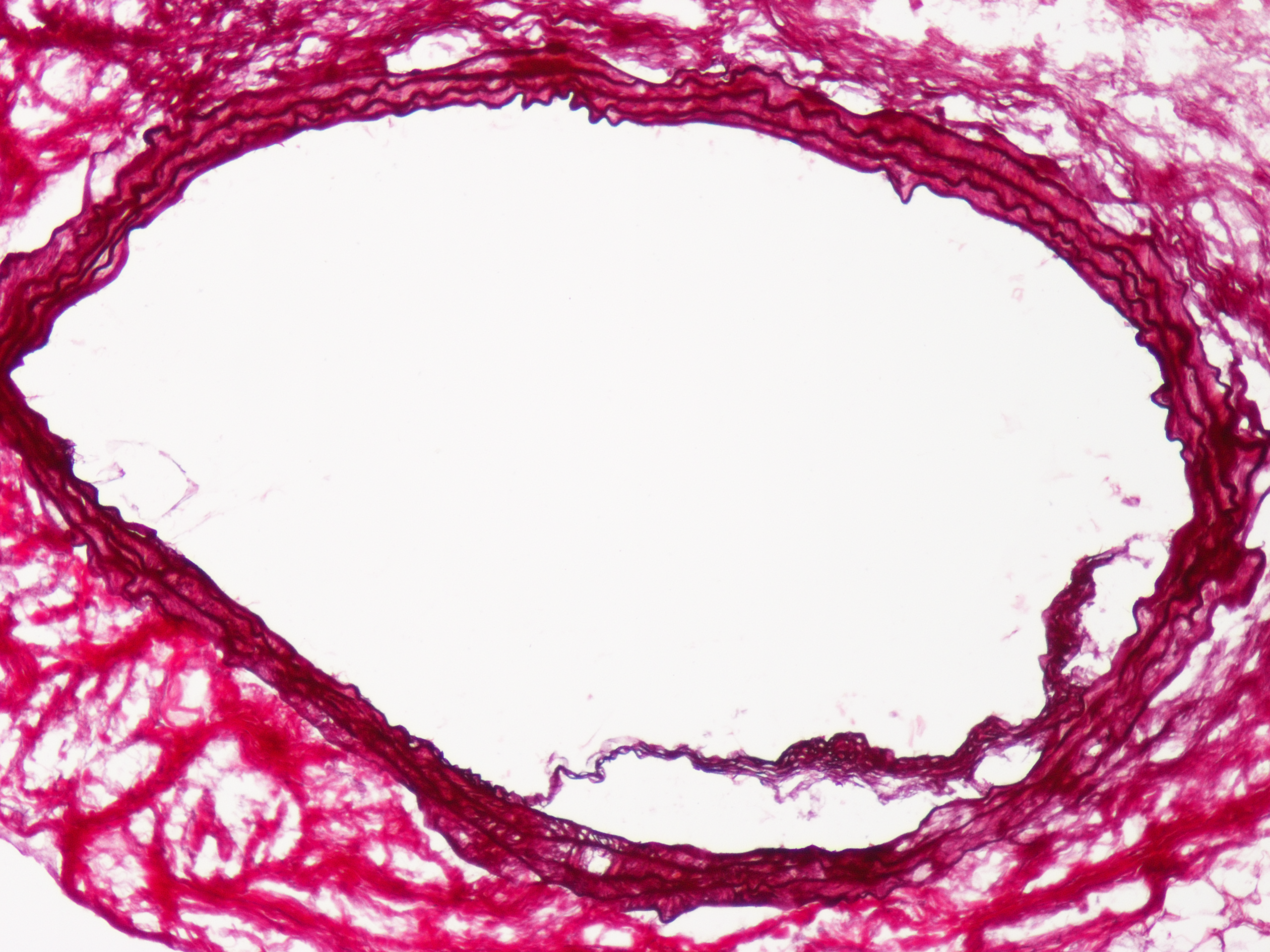

Supplement: Supplementary file 9 — Source data Fig. 8 [file 44321_2025_318_MOESM9_ESM.zip › Figure 8/Figure 8D/EVG Staining/Saline 100um.tif]

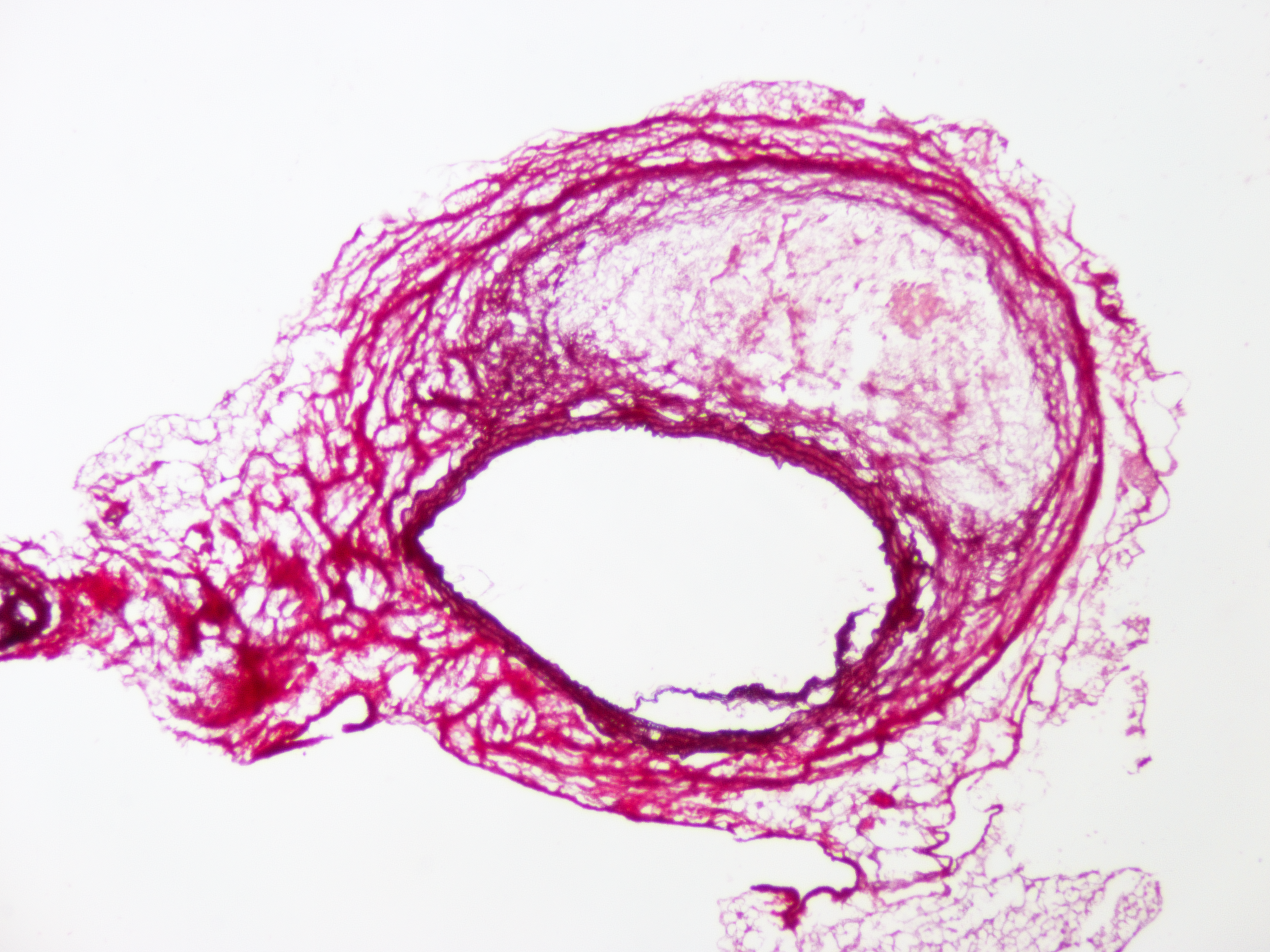

Supplement: Supplementary file 9 — Source data Fig. 8 [file 44321_2025_318_MOESM9_ESM.zip › Figure 8/Figure 8D/EVG Staining/Saline 200um.tif]

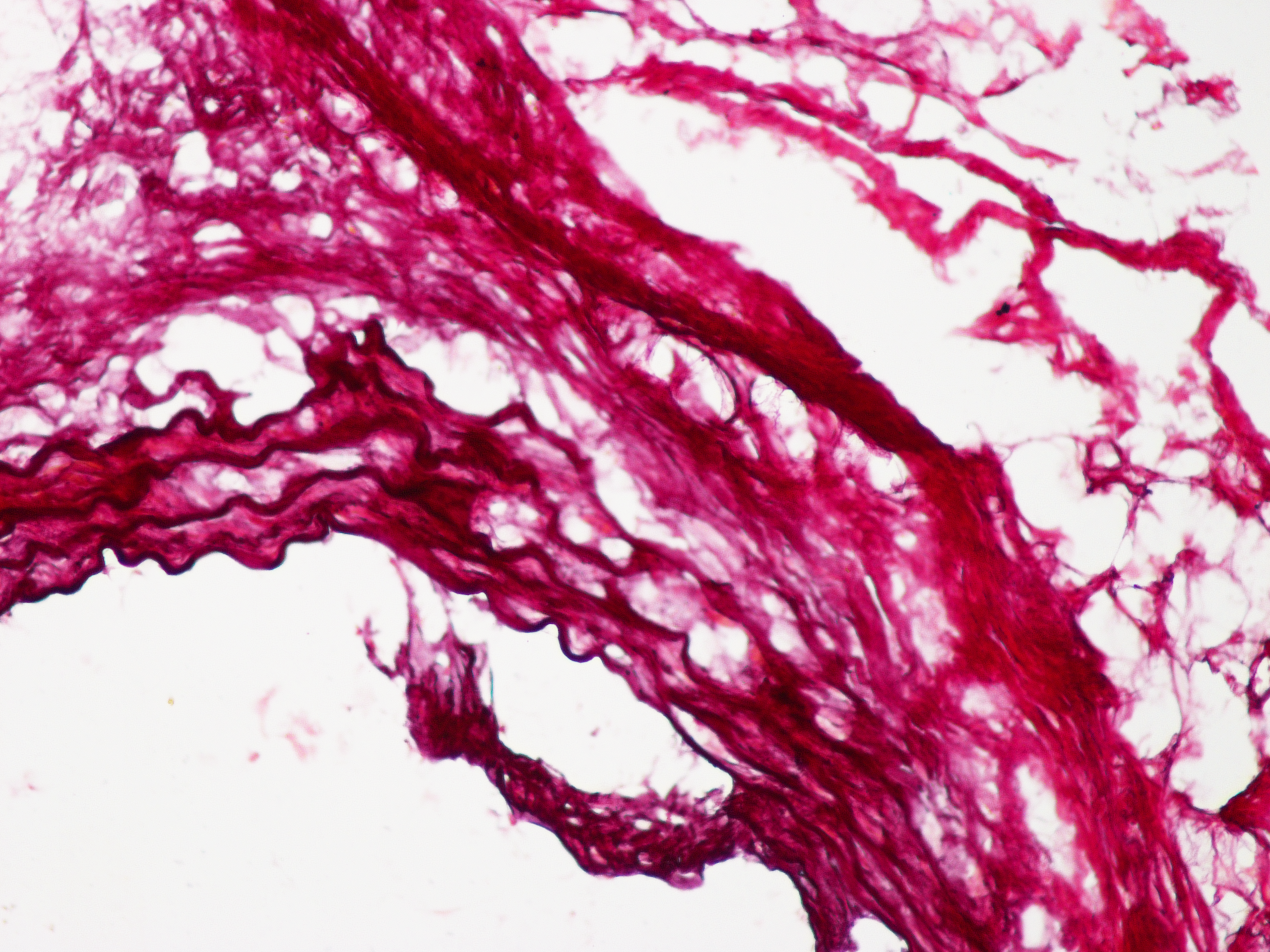

Supplement: Supplementary file 9 — Source data Fig. 8 [file 44321_2025_318_MOESM9_ESM.zip › Figure 8/Figure 8D/EVG Staining/Saline 50um.tif]

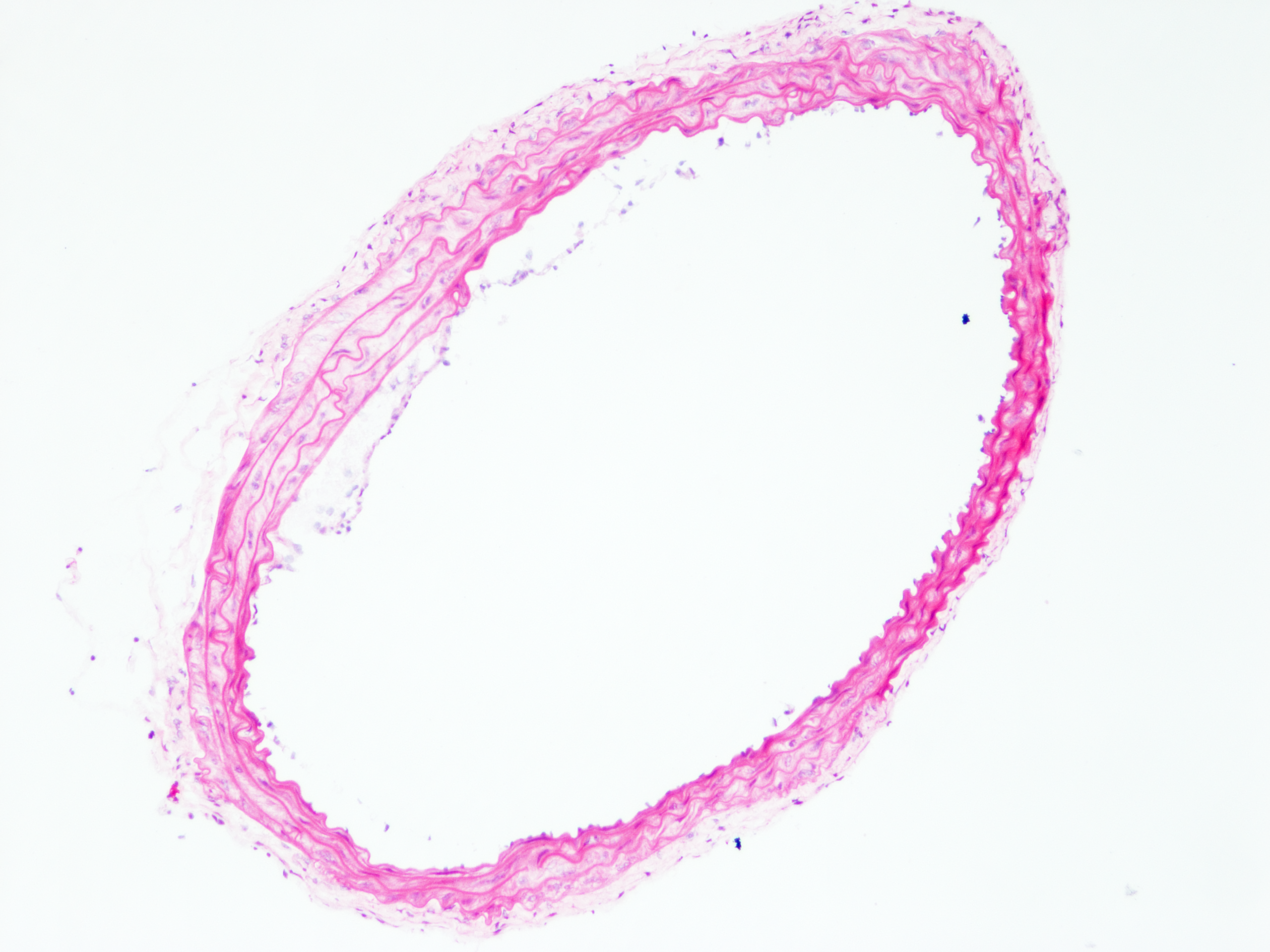

Supplement: Supplementary file 9 — Source data Fig. 8 [file 44321_2025_318_MOESM9_ESM.zip › Figure 8/Figure 8D/HE Staining/FSTL1 100um.tif]

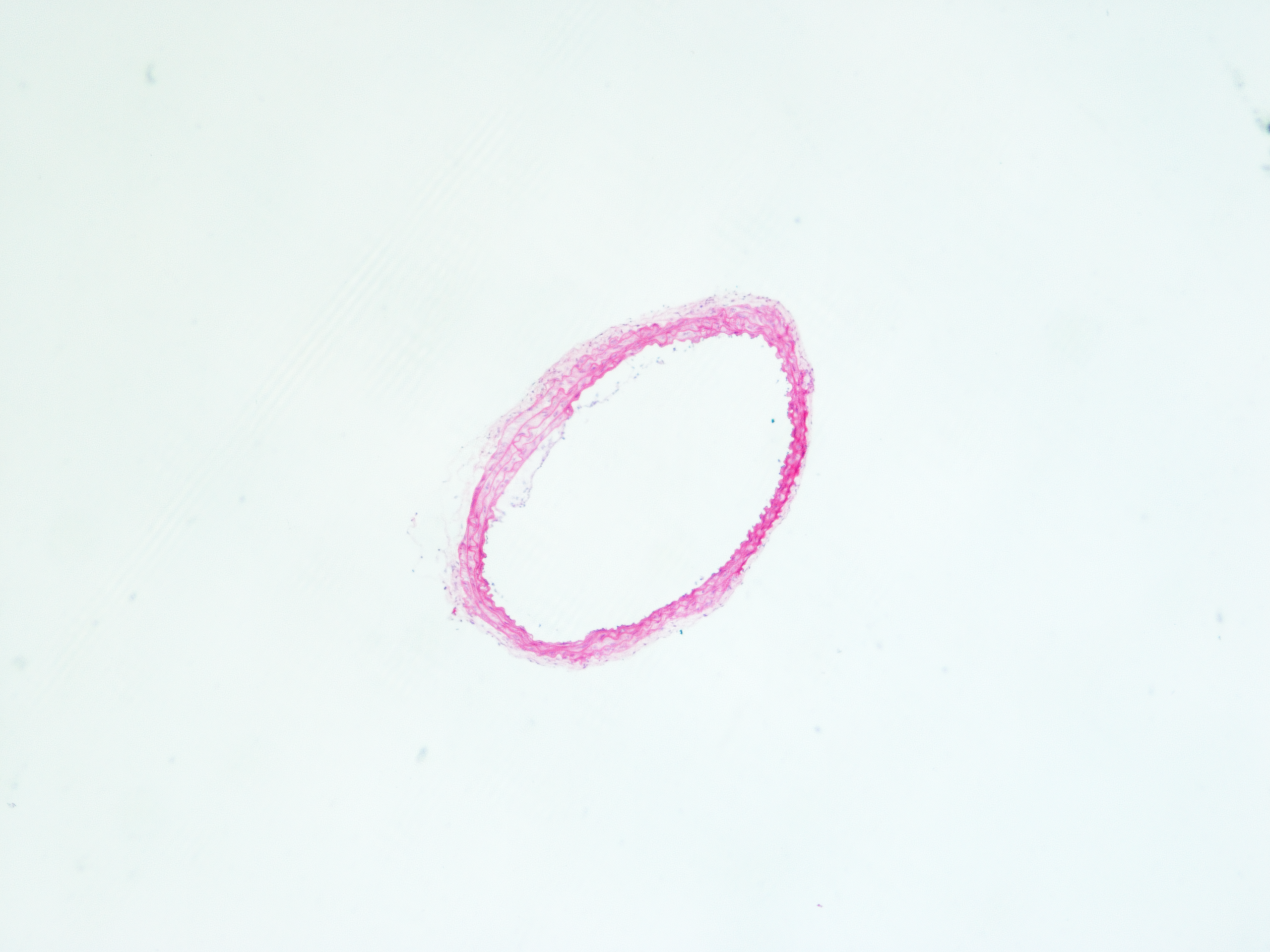

Supplement: Supplementary file 9 — Source data Fig. 8 [file 44321_2025_318_MOESM9_ESM.zip › Figure 8/Figure 8D/HE Staining/FSTL1 200um.tif]

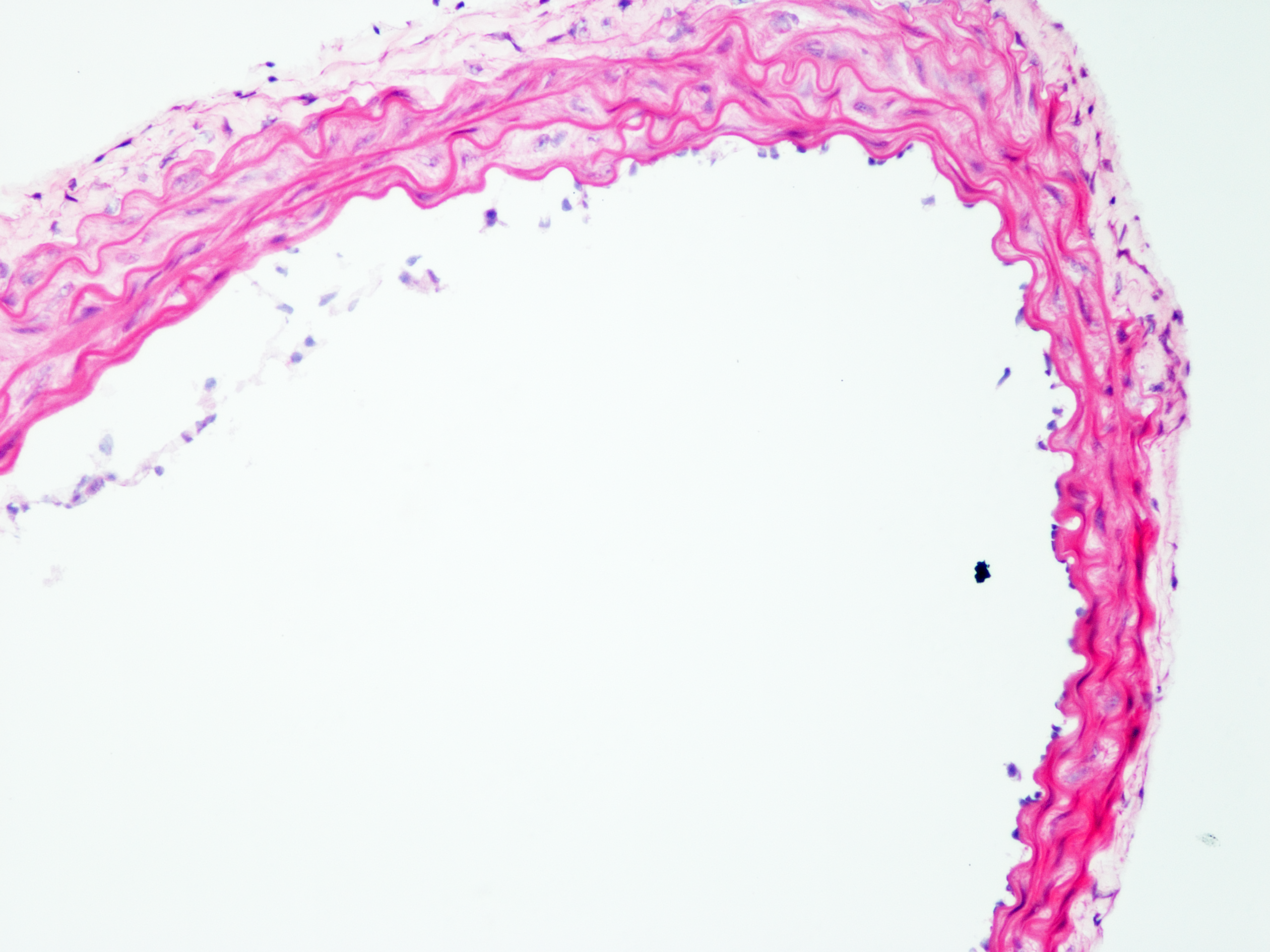

Supplement: Supplementary file 9 — Source data Fig. 8 [file 44321_2025_318_MOESM9_ESM.zip › Figure 8/Figure 8D/HE Staining/FSTL1 50um.tif]

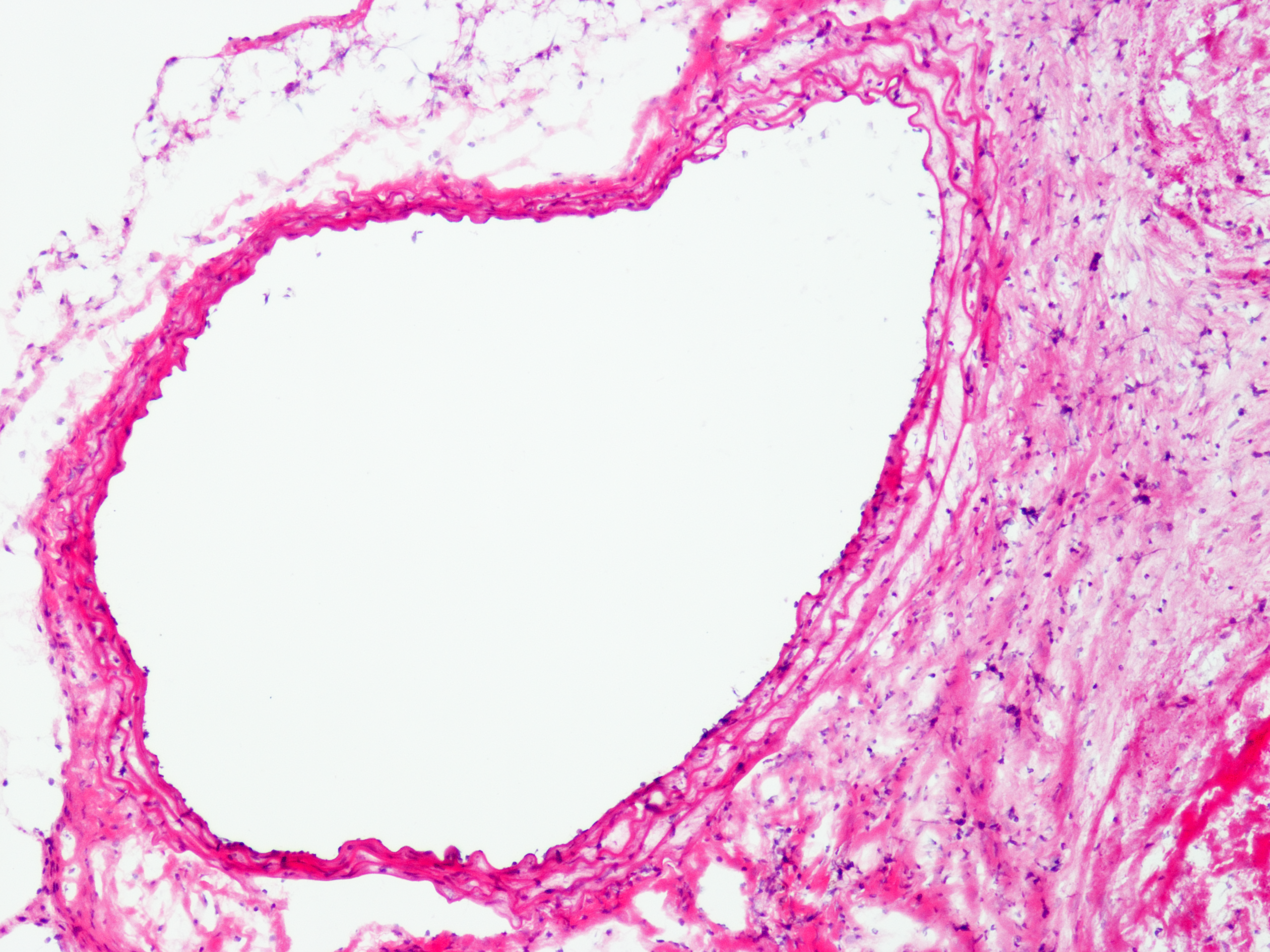

Supplement: Supplementary file 9 — Source data Fig. 8 [file 44321_2025_318_MOESM9_ESM.zip › Figure 8/Figure 8D/HE Staining/Saline 100um.tif]

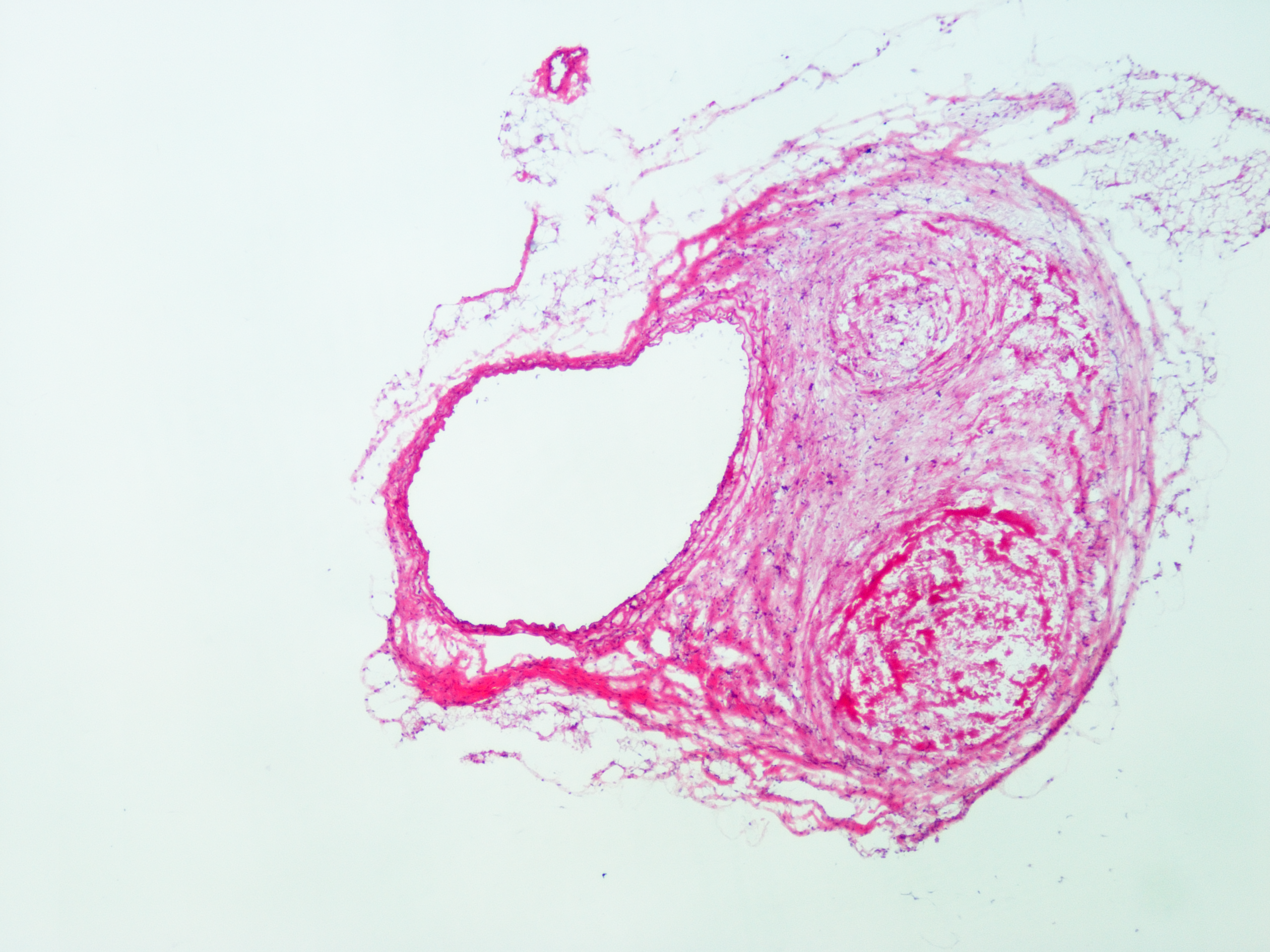

Supplement: Supplementary file 9 — Source data Fig. 8 [file 44321_2025_318_MOESM9_ESM.zip › Figure 8/Figure 8D/HE Staining/Saline 200um.tif]

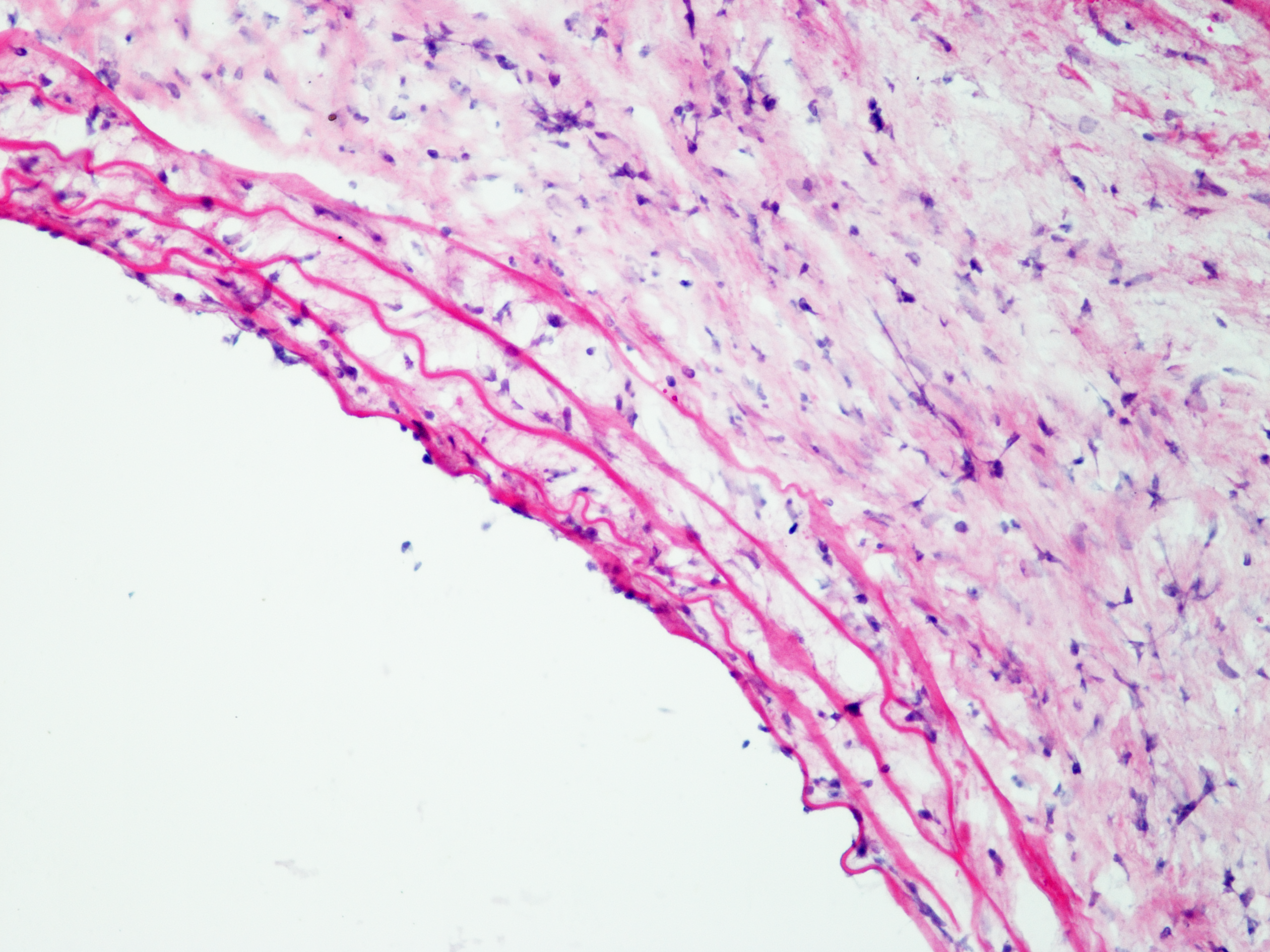

Supplement: Supplementary file 9 — Source data Fig. 8 [file 44321_2025_318_MOESM9_ESM.zip › Figure 8/Figure 8D/HE Staining/Saline 50um.tif]

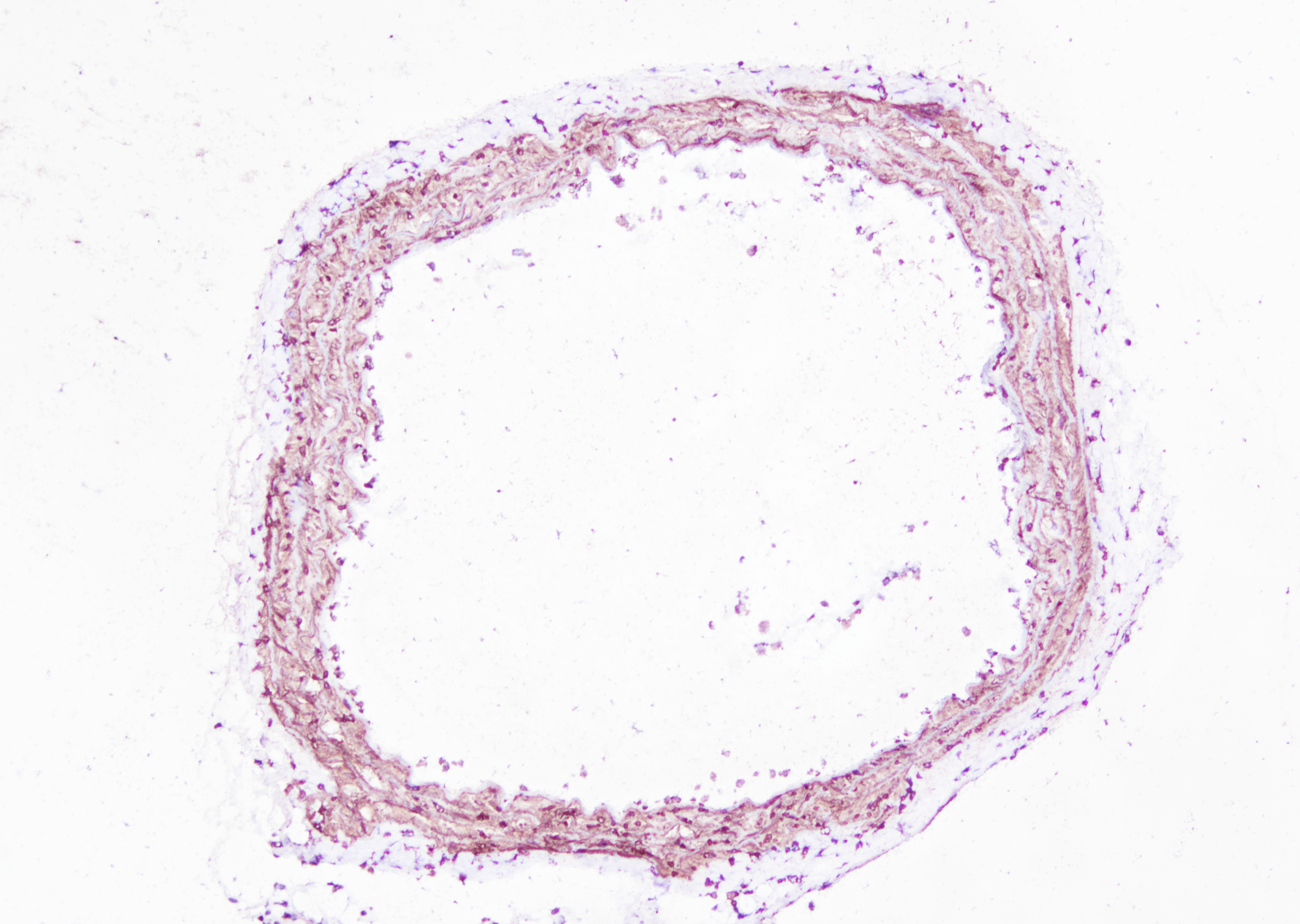

Supplement: Supplementary file 9 — Source data Fig. 8 [file 44321_2025_318_MOESM9_ESM.zip › Figure 8/Figure 8F/FSTL1 a-SMA 100um.tif]

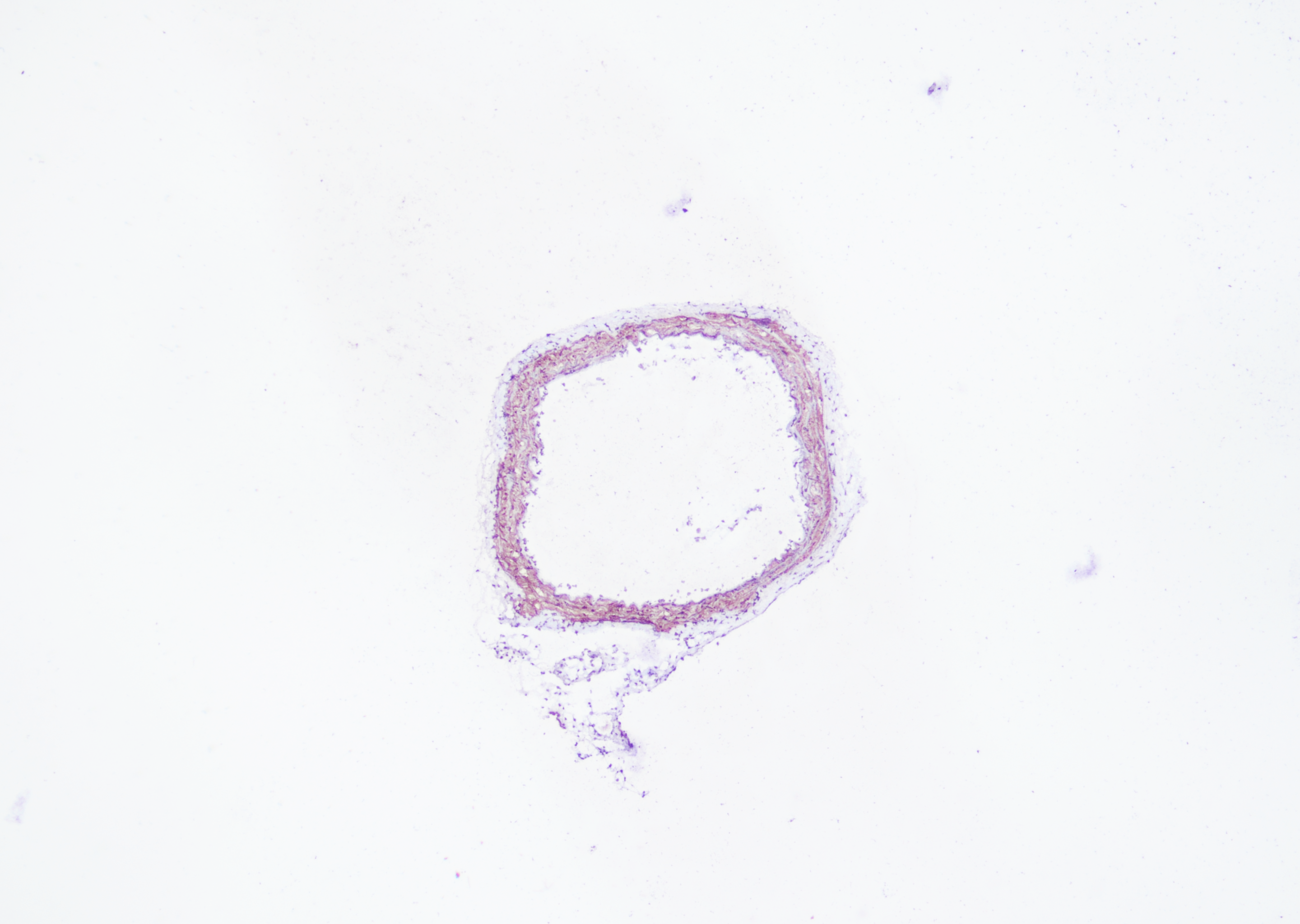

Supplement: Supplementary file 9 — Source data Fig. 8 [file 44321_2025_318_MOESM9_ESM.zip › Figure 8/Figure 8F/FSTL1 a-SMA 200um.tif]

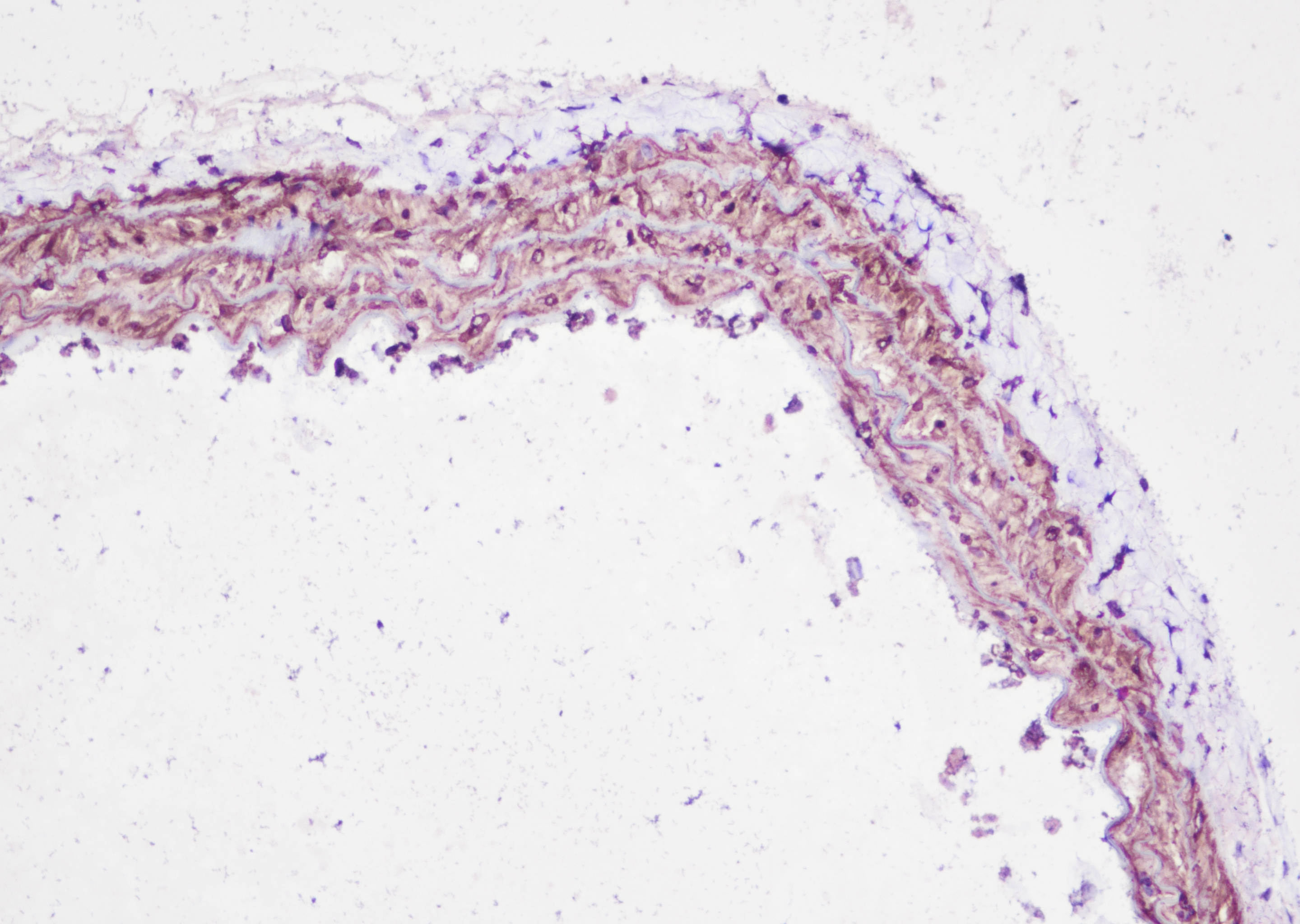

Supplement: Supplementary file 9 — Source data Fig. 8 [file 44321_2025_318_MOESM9_ESM.zip › Figure 8/Figure 8F/FSTL1 a-SMA 50um.tif]

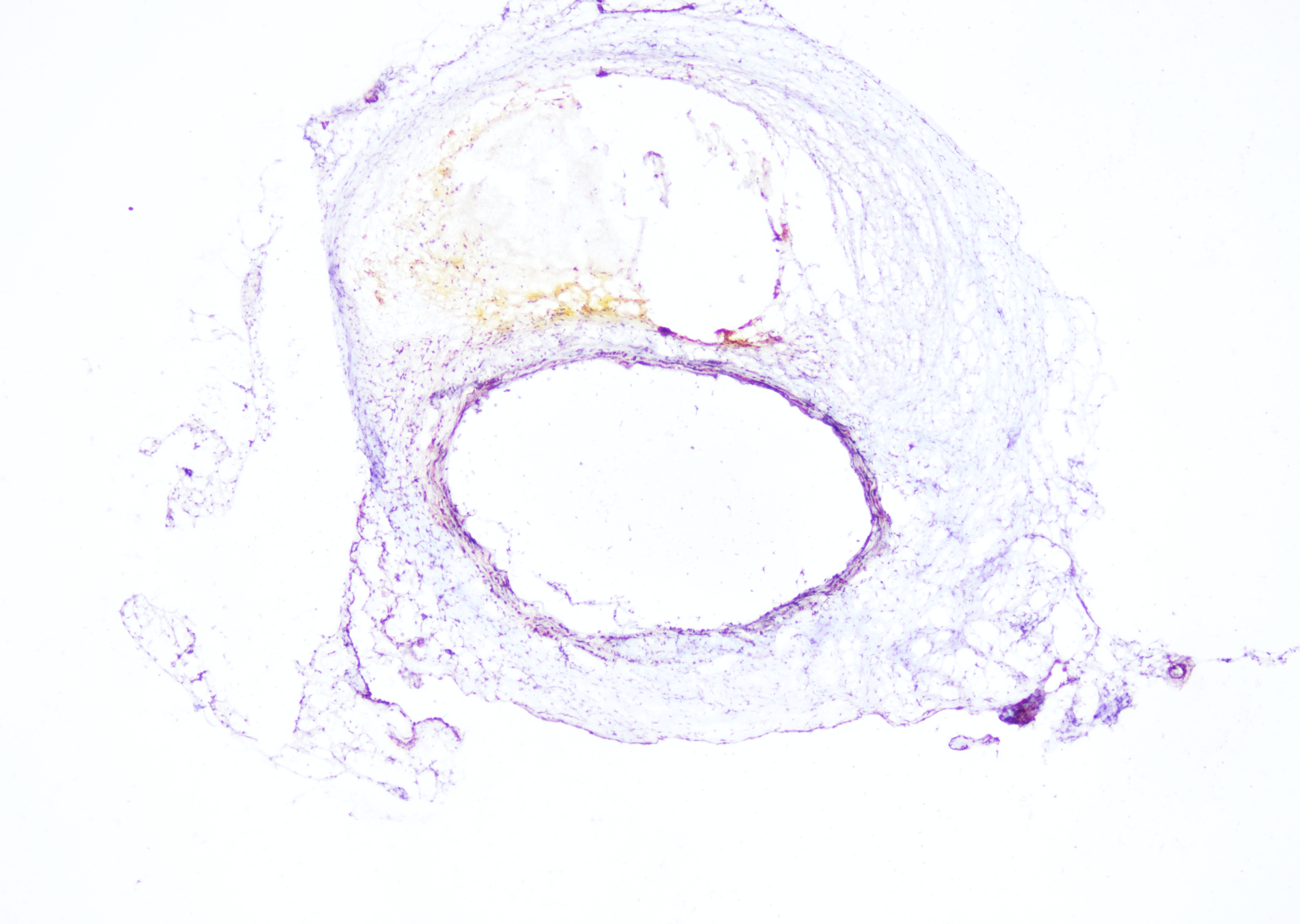

Supplement: Supplementary file 9 — Source data Fig. 8 [file 44321_2025_318_MOESM9_ESM.zip › Figure 8/Figure 8F/Saline a-SMA 200um.tif]

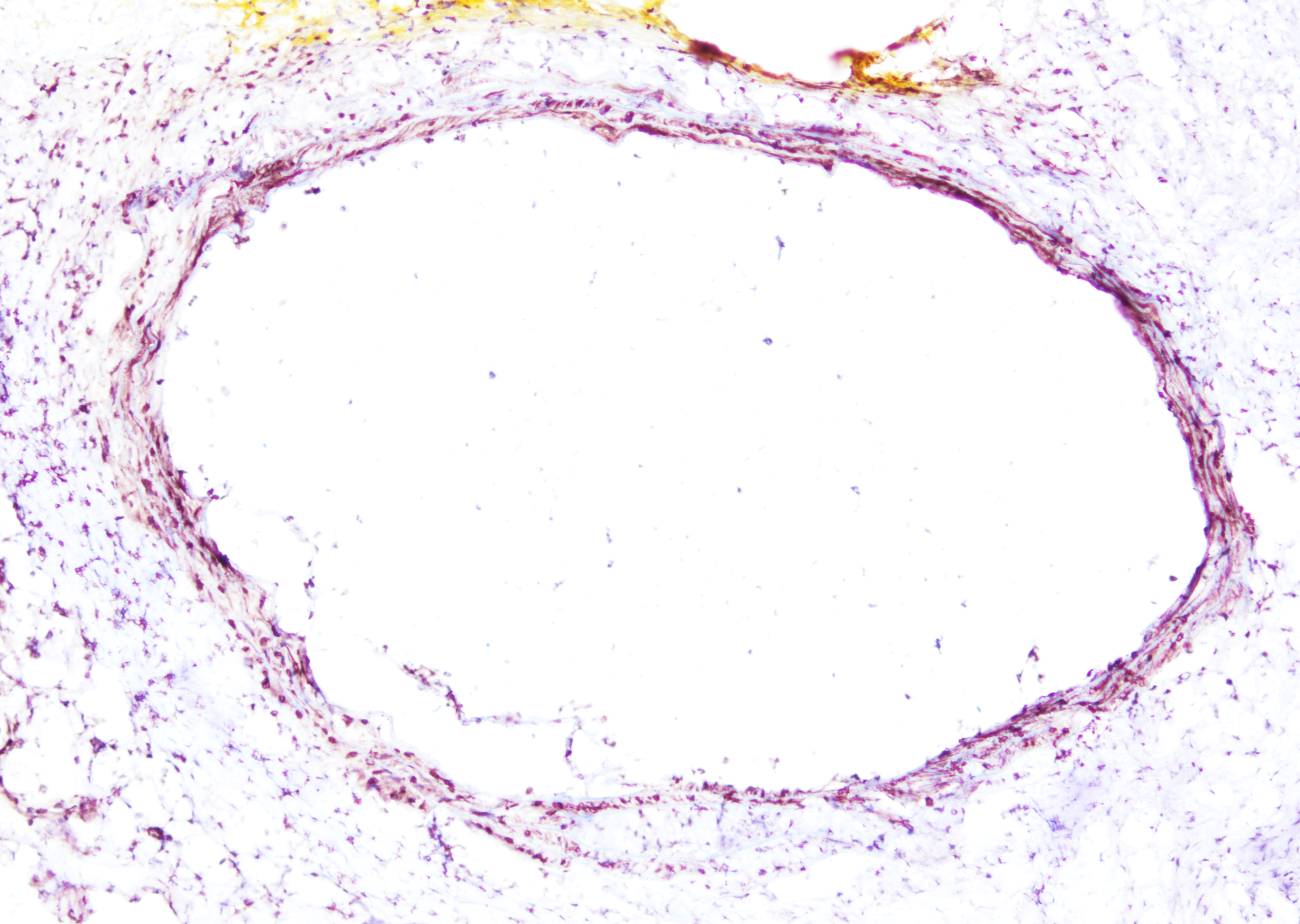

Supplement: Supplementary file 9 — Source data Fig. 8 [file 44321_2025_318_MOESM9_ESM.zip › Figure 8/Figure 8F/Saline a-SMA 100um.tif]

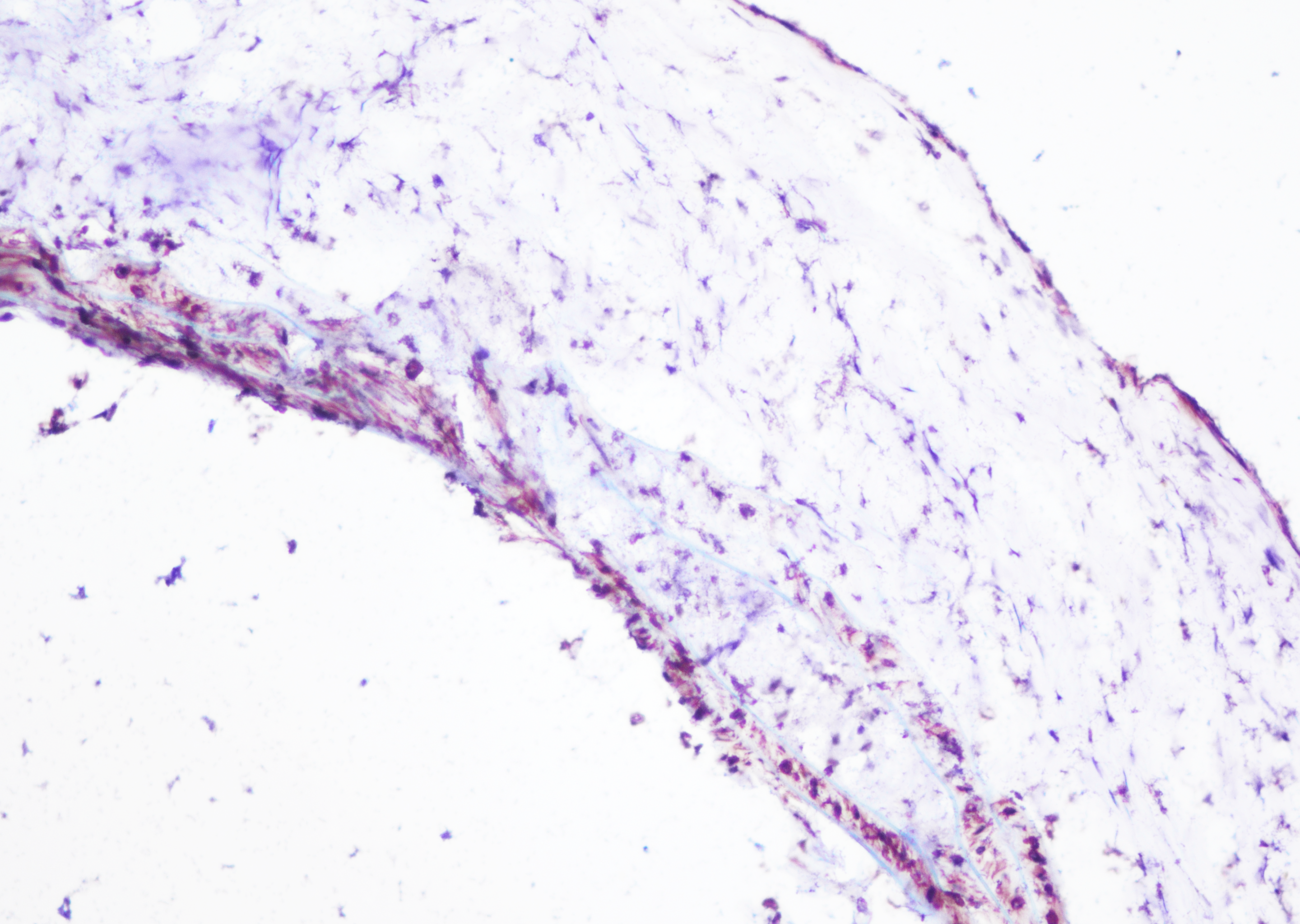

Supplement: Supplementary file 9 — Source data Fig. 8 [file 44321_2025_318_MOESM9_ESM.zip › Figure 8/Figure 8F/Saline a-SMA 50um.tif]

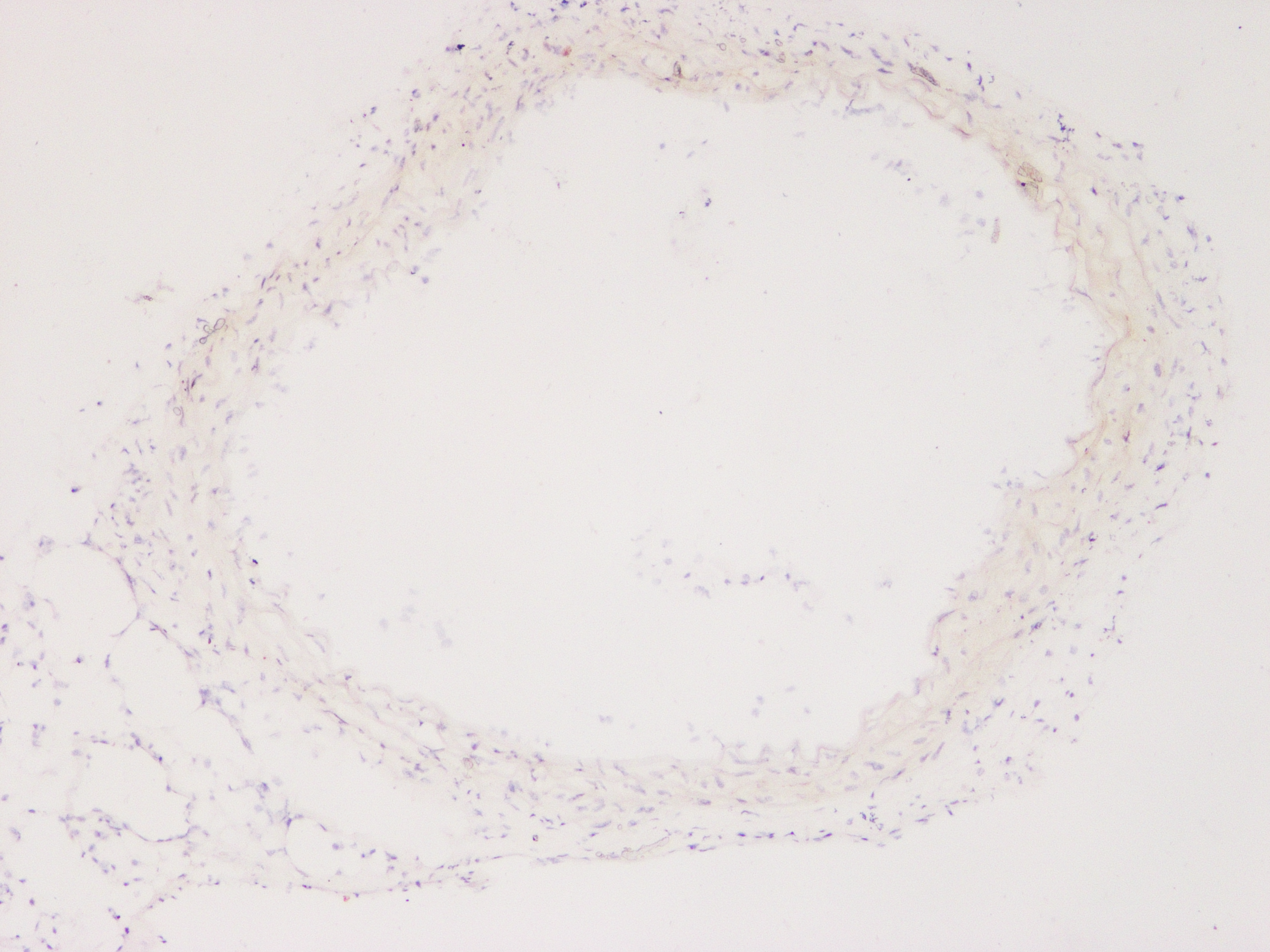

Supplement: Supplementary file 9 — Source data Fig. 8 [file 44321_2025_318_MOESM9_ESM.zip › Figure 8/Figure 8H/FSTL1 Cleaved-Caspase3 100um.tif]

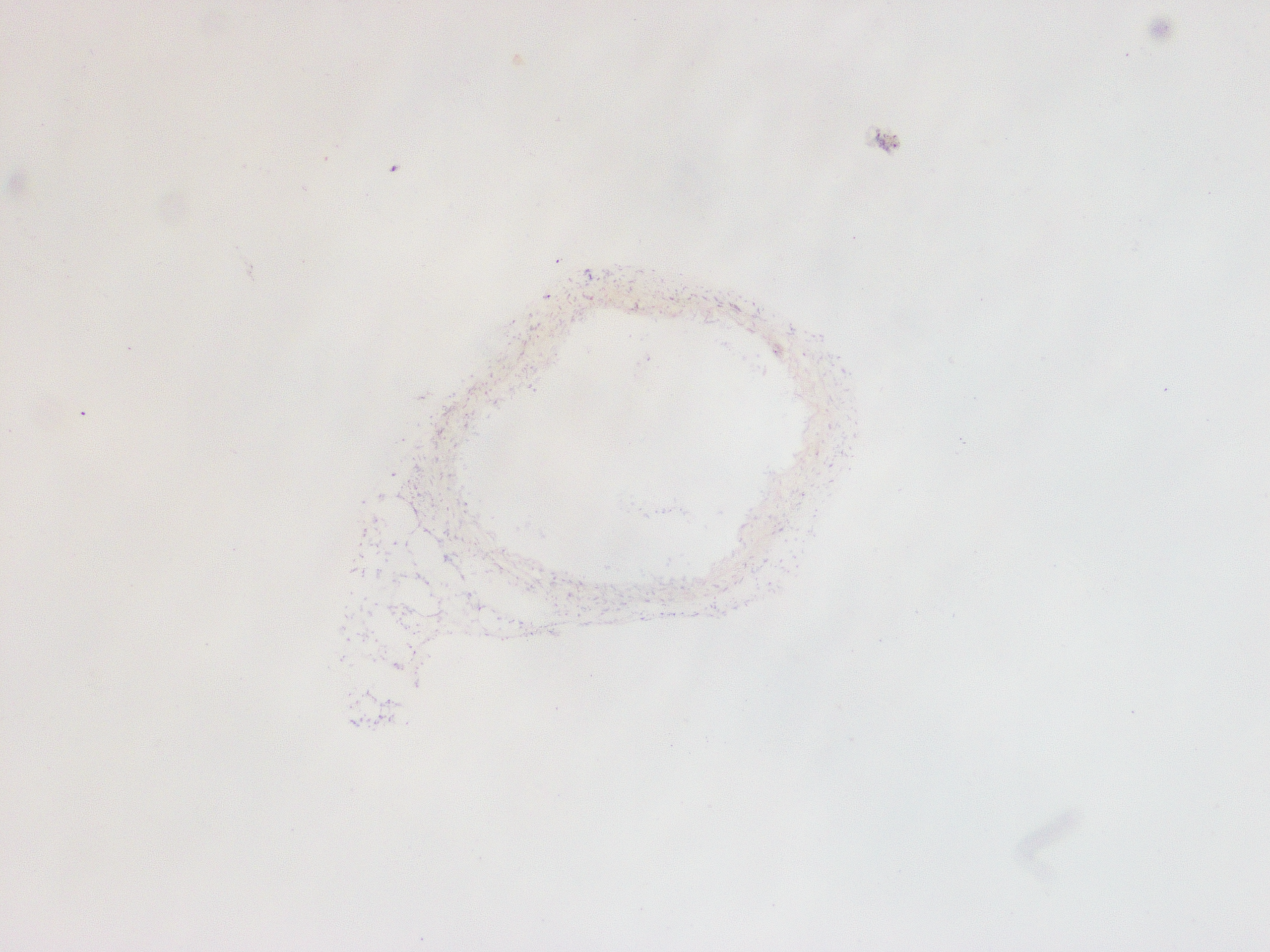

Supplement: Supplementary file 9 — Source data Fig. 8 [file 44321_2025_318_MOESM9_ESM.zip › Figure 8/Figure 8H/FSTL1 Cleaved-caspase3 200um.tif]

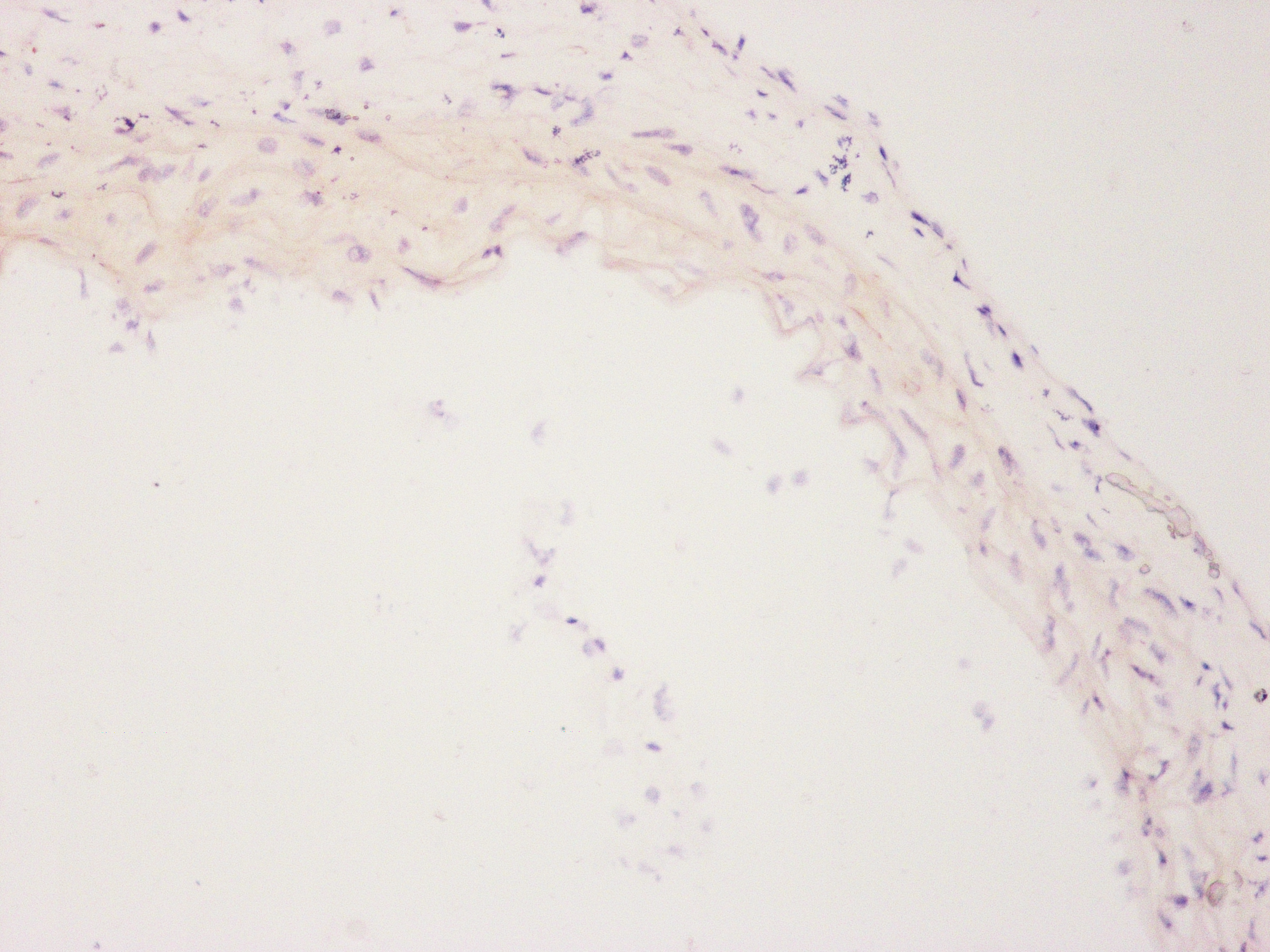

Supplement: Supplementary file 9 — Source data Fig. 8 [file 44321_2025_318_MOESM9_ESM.zip › Figure 8/Figure 8H/FSTL1 Cleaved-caspase3 50um.tif]

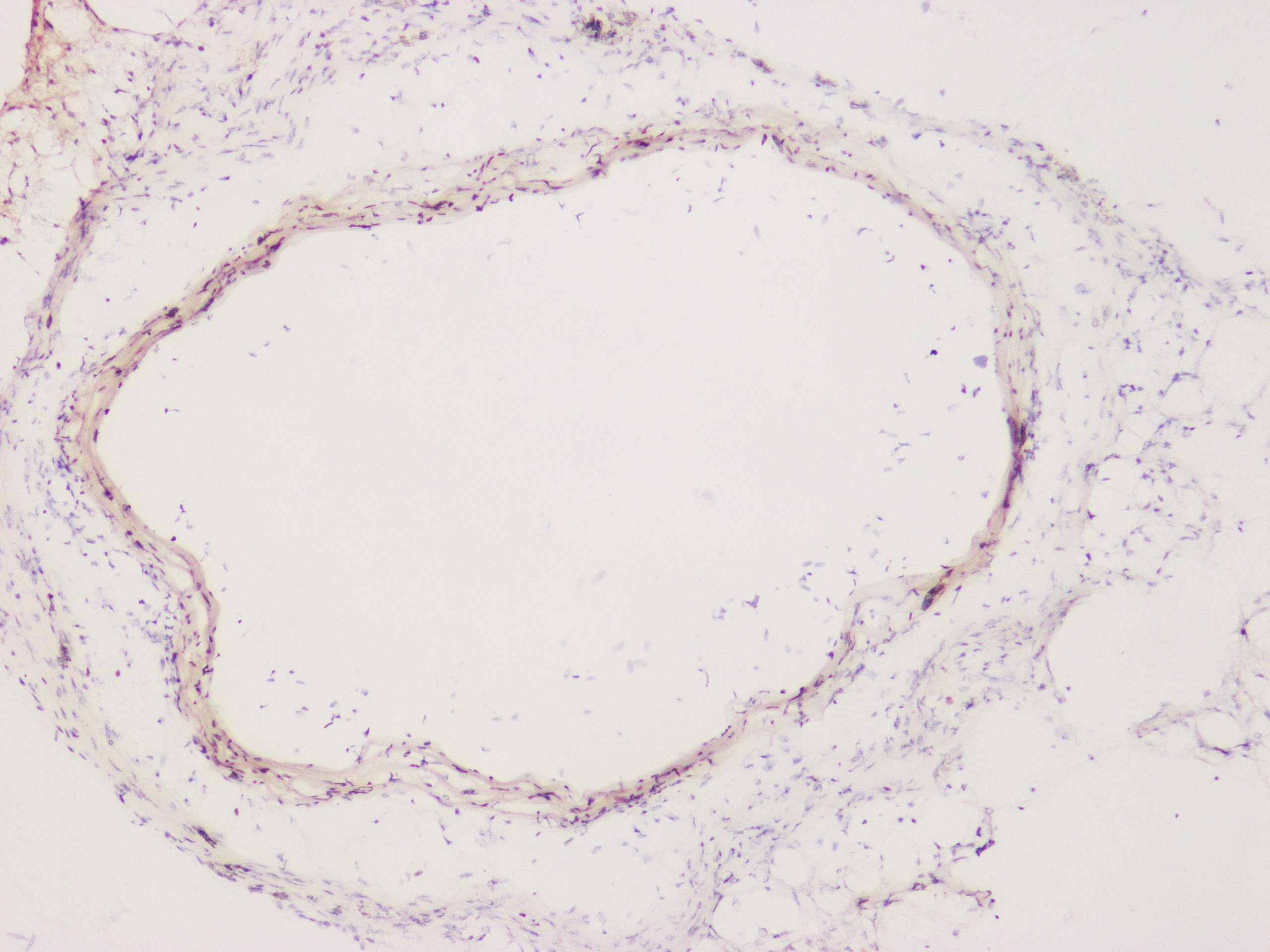

Supplement: Supplementary file 9 — Source data Fig. 8 [file 44321_2025_318_MOESM9_ESM.zip › Figure 8/Figure 8H/Saline Cleaved-caspase3 100um.tif]

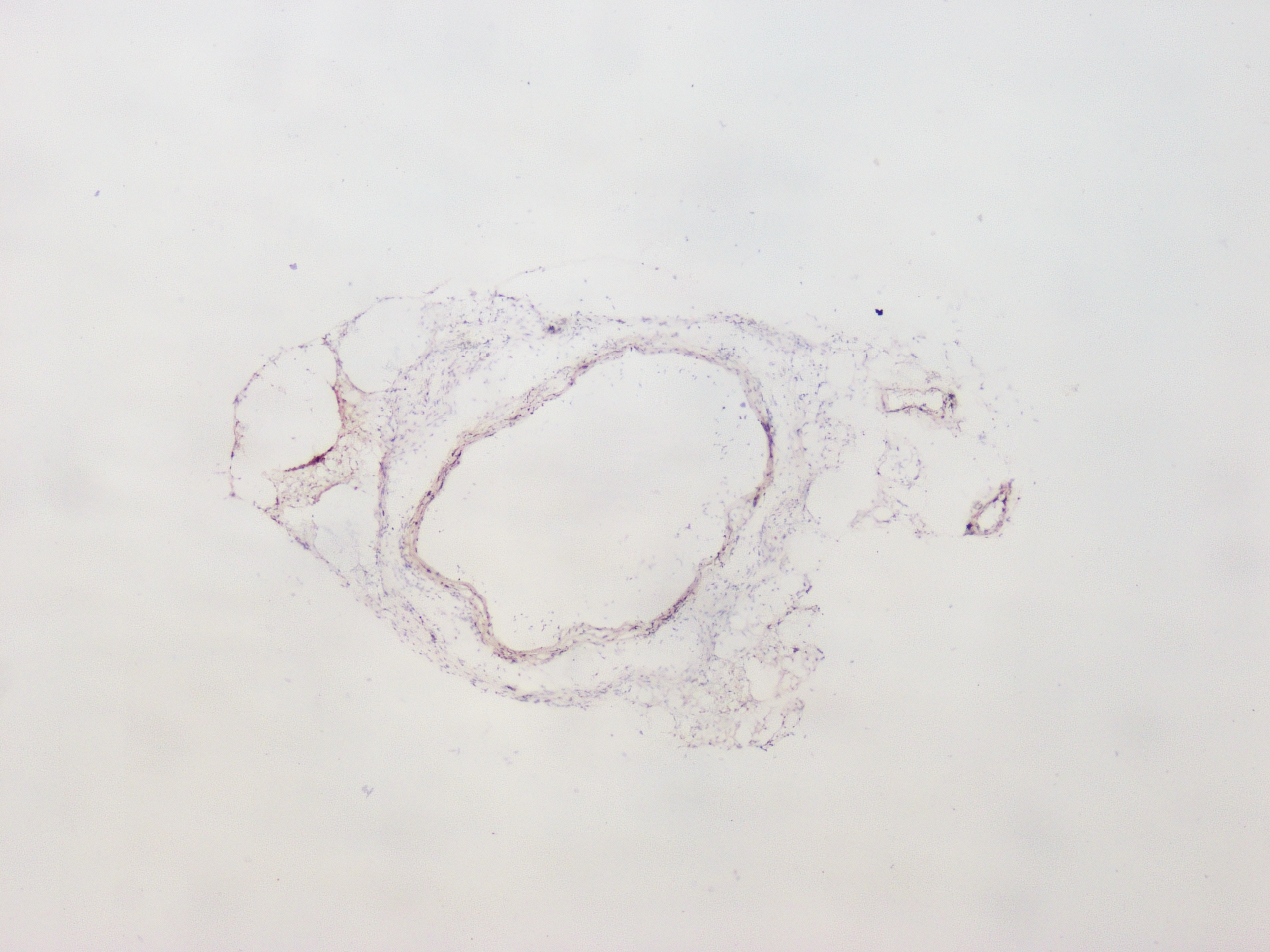

Supplement: Supplementary file 9 — Source data Fig. 8 [file 44321_2025_318_MOESM9_ESM.zip › Figure 8/Figure 8H/Saline Cleaved-caspase3 200um.tif]

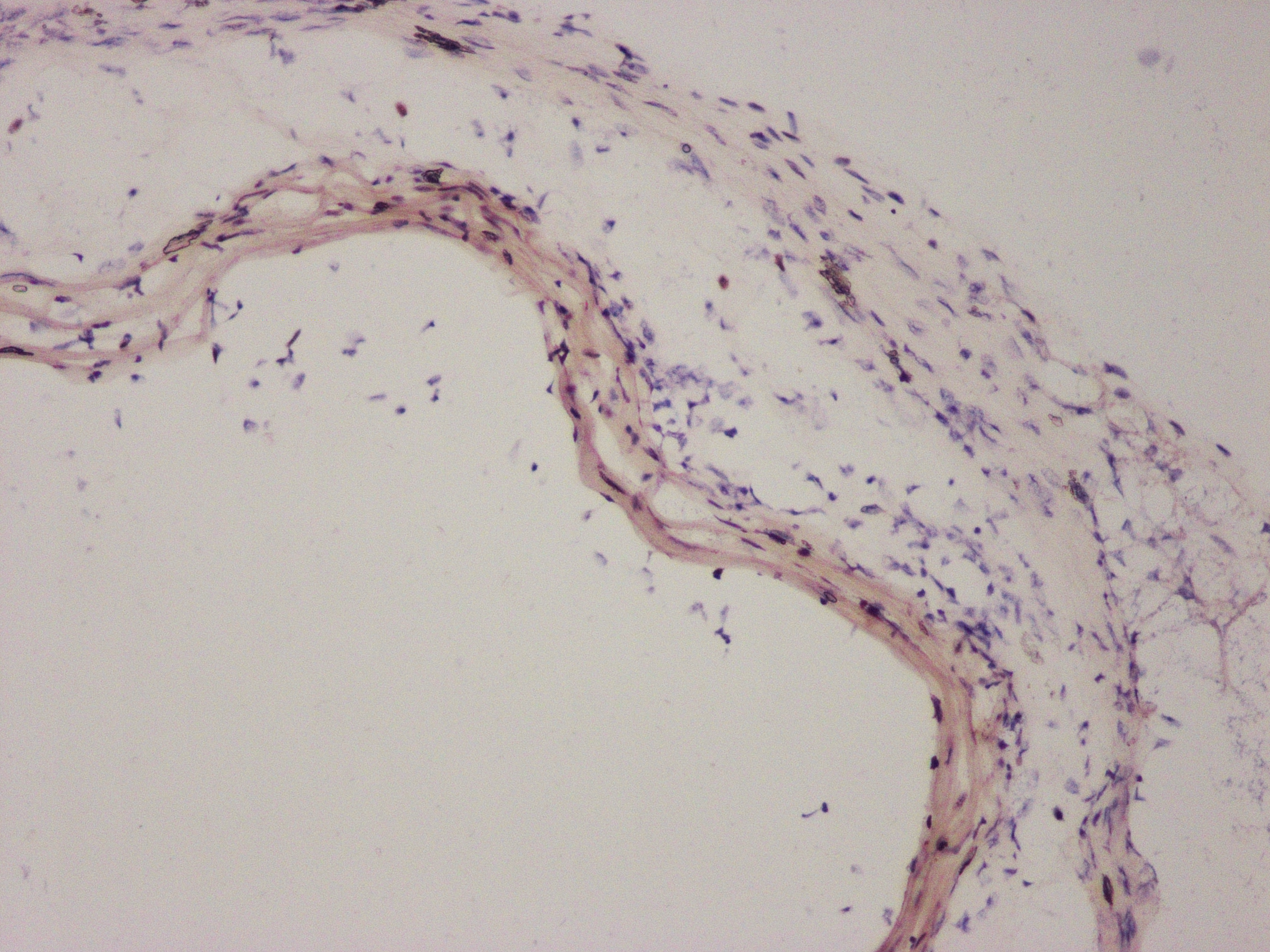

Supplement: Supplementary file 9 — Source data Fig. 8 [file 44321_2025_318_MOESM9_ESM.zip › Figure 8/Figure 8H/Saline Cleaved-caspase3 50um.tif]

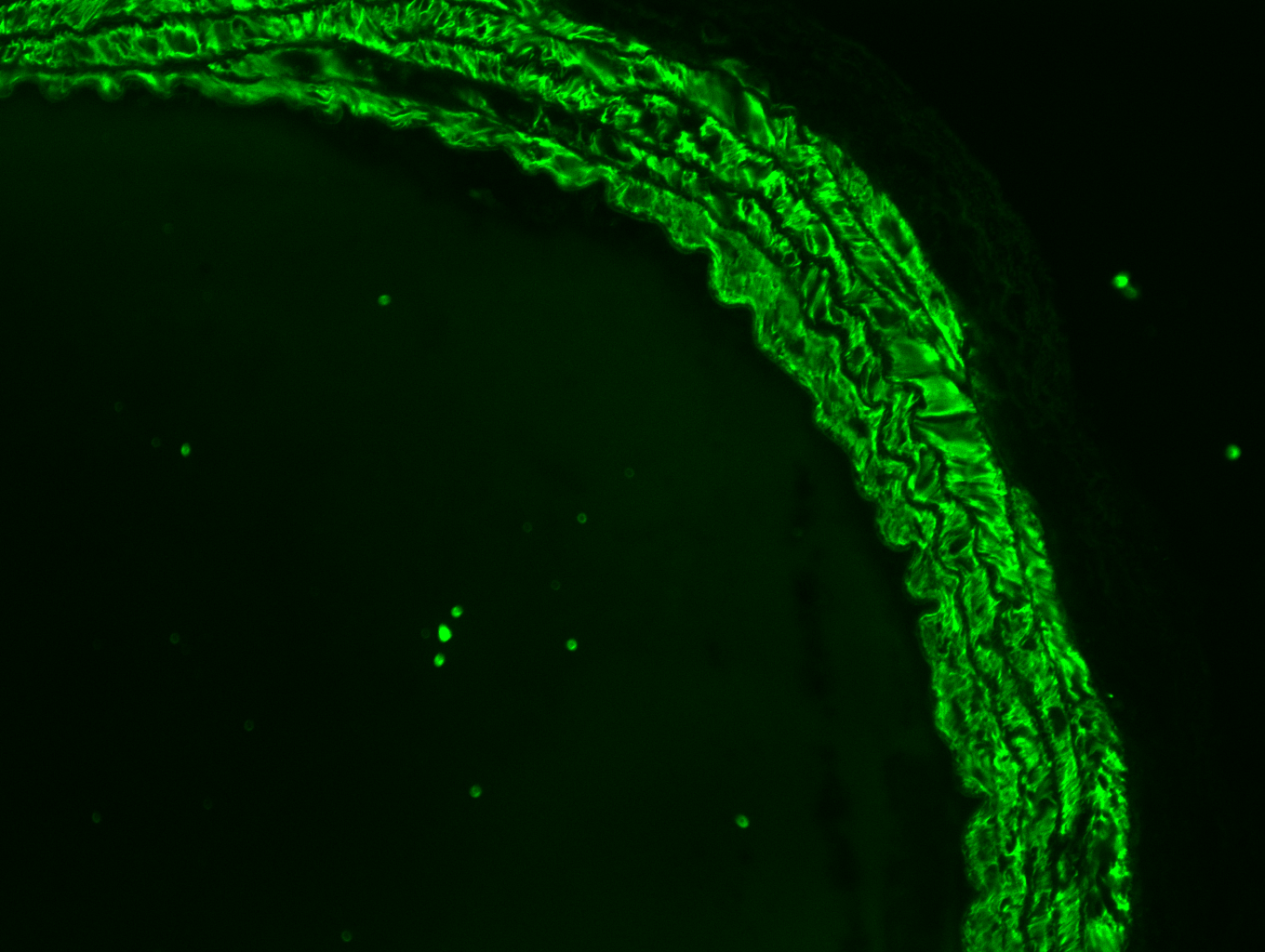

Supplement: Supplementary file 9 — Source data Fig. 8 [file 44321_2025_318_MOESM9_ESM.zip › Figure 8/Figure 8J/FSTL1 aSMA.tif]

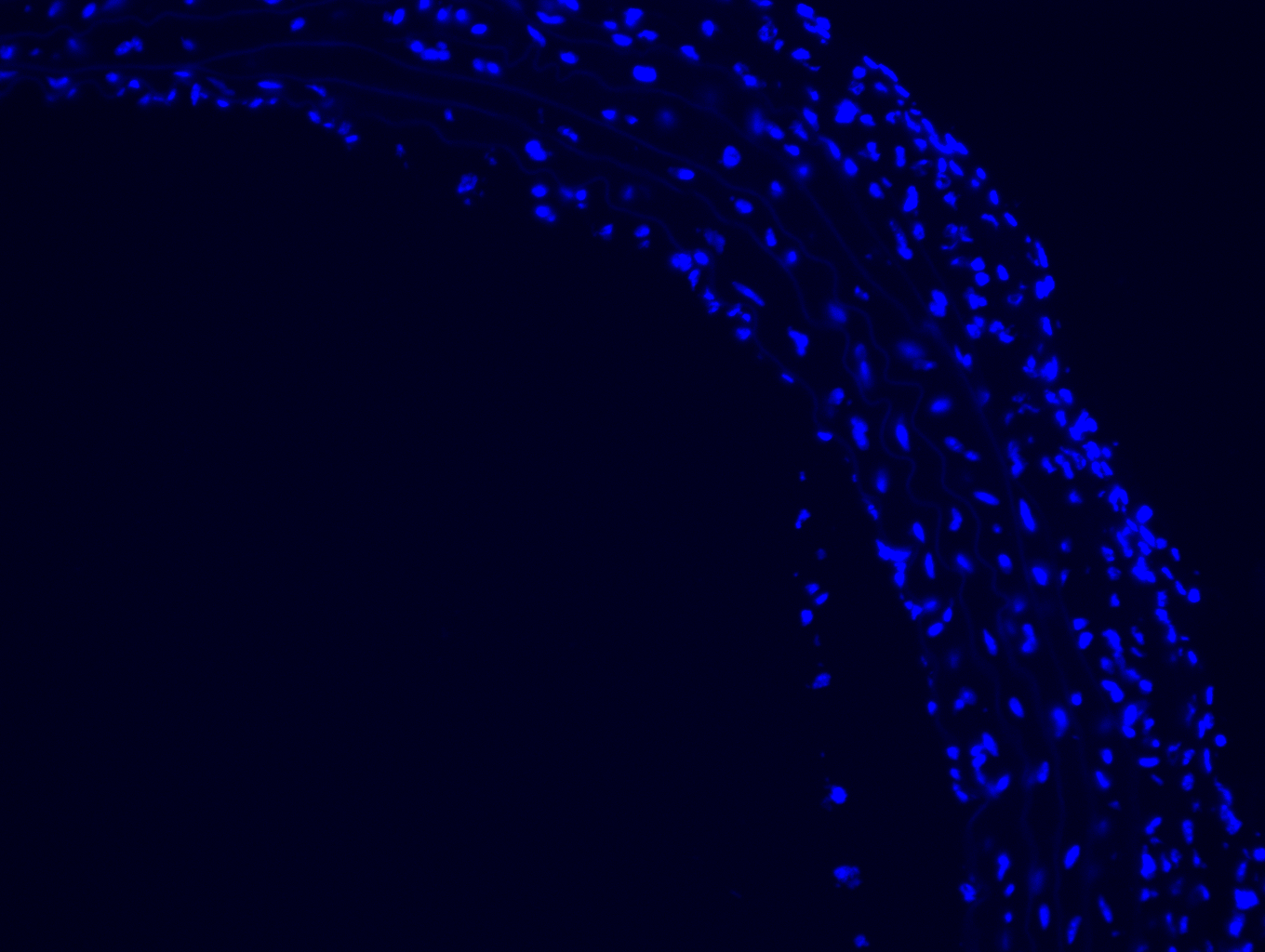

Supplement: Supplementary file 9 — Source data Fig. 8 [file 44321_2025_318_MOESM9_ESM.zip › Figure 8/Figure 8J/FSTL1 DAPI.tif]

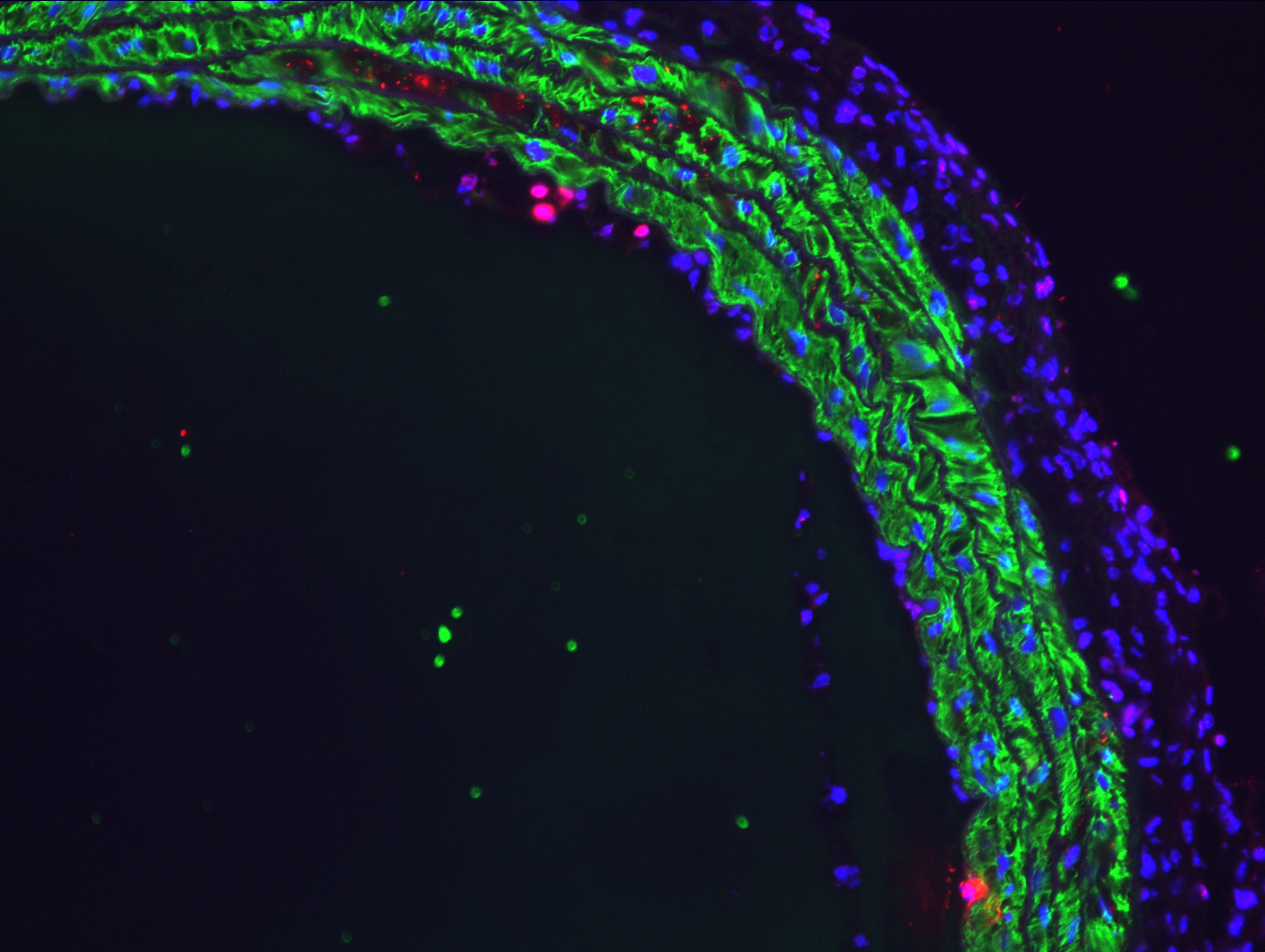

Supplement: Supplementary file 9 — Source data Fig. 8 [file 44321_2025_318_MOESM9_ESM.zip › Figure 8/Figure 8J/FSTL1 Merge.tif]

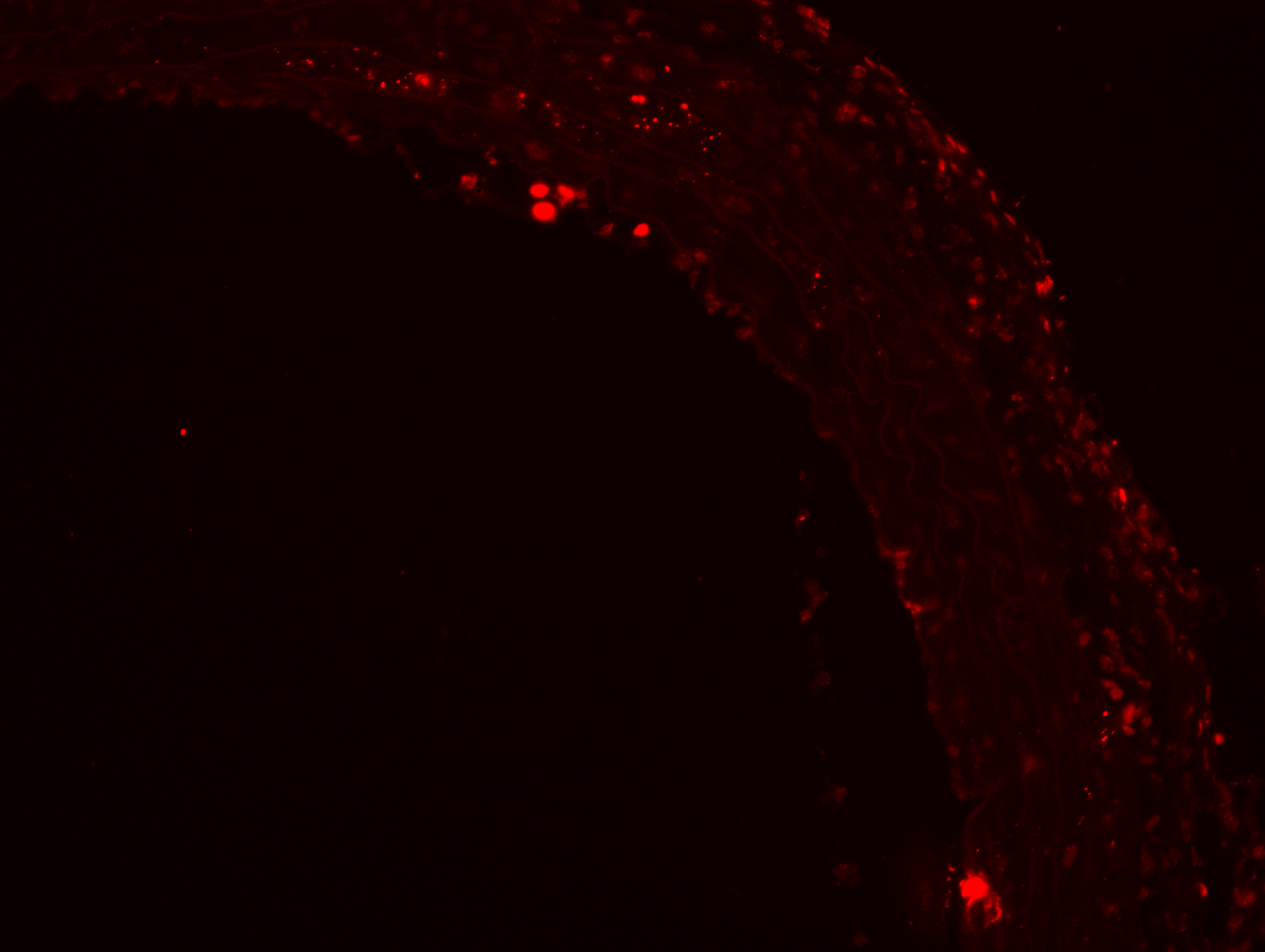

Supplement: Supplementary file 9 — Source data Fig. 8 [file 44321_2025_318_MOESM9_ESM.zip › Figure 8/Figure 8J/FSTL1 Tunel.tif]

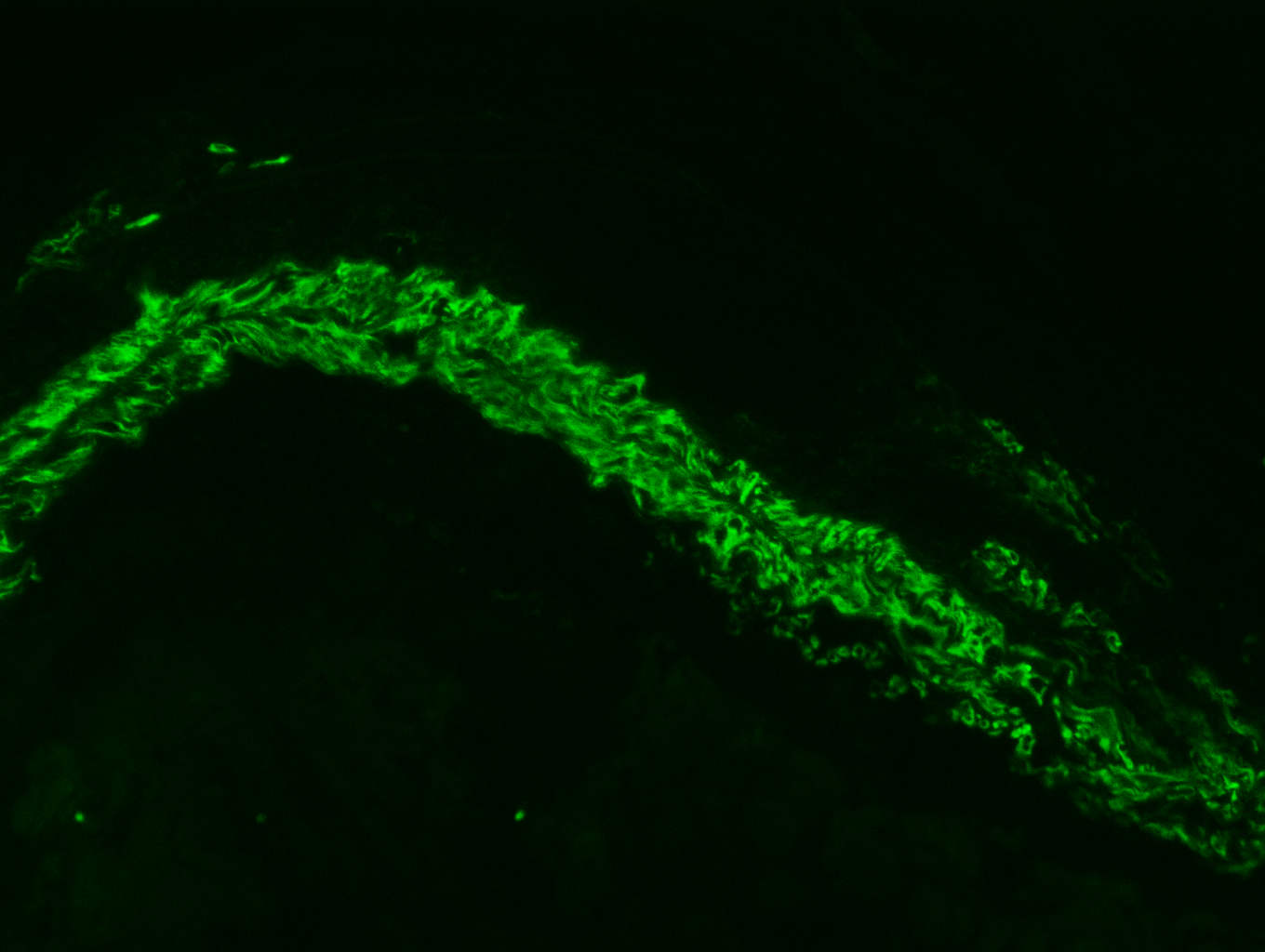

Supplement: Supplementary file 9 — Source data Fig. 8 [file 44321_2025_318_MOESM9_ESM.zip › Figure 8/Figure 8J/Saline aSMA.tif]

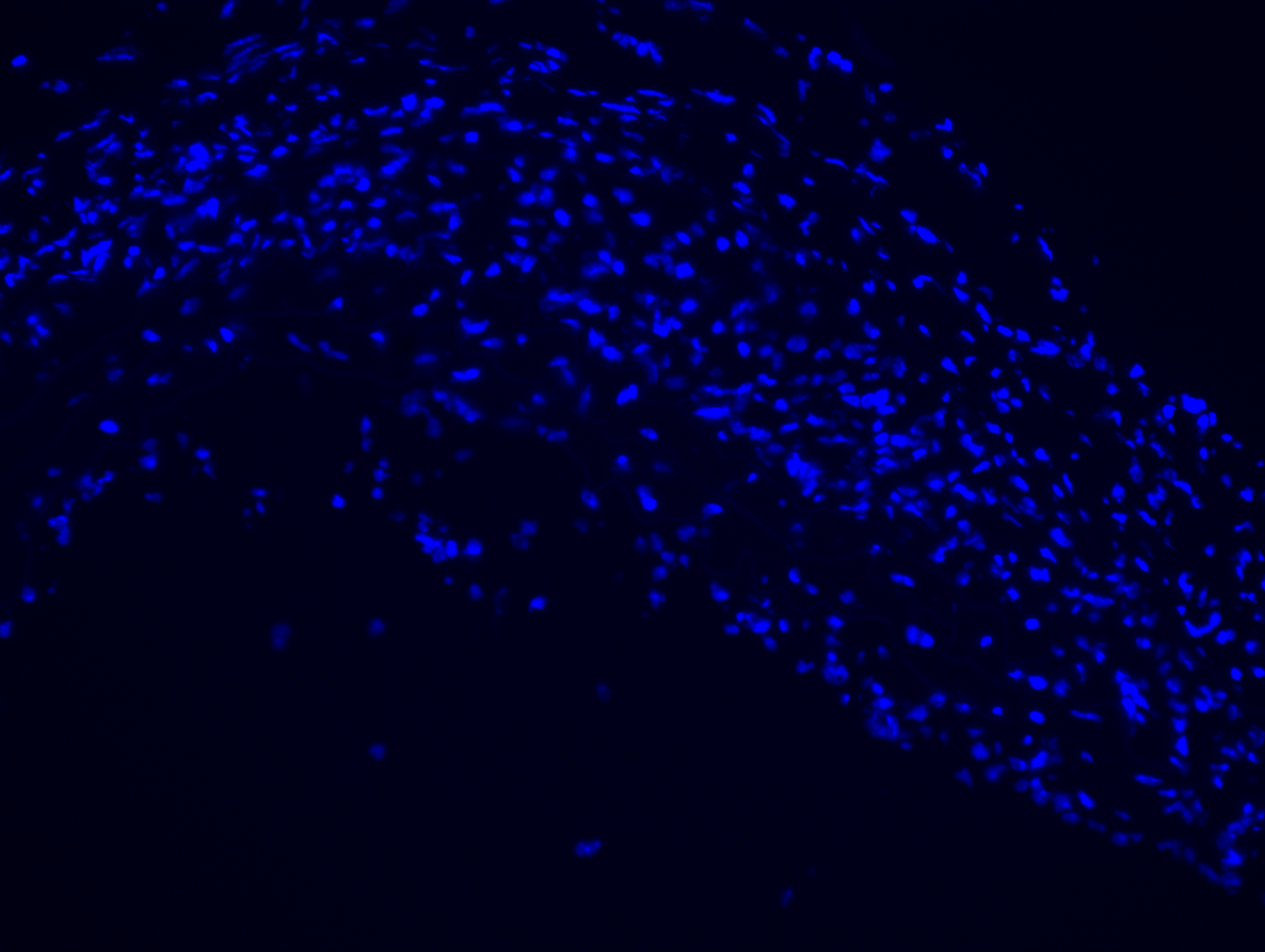

Supplement: Supplementary file 9 — Source data Fig. 8 [file 44321_2025_318_MOESM9_ESM.zip › Figure 8/Figure 8J/Saline DAPI.tif]

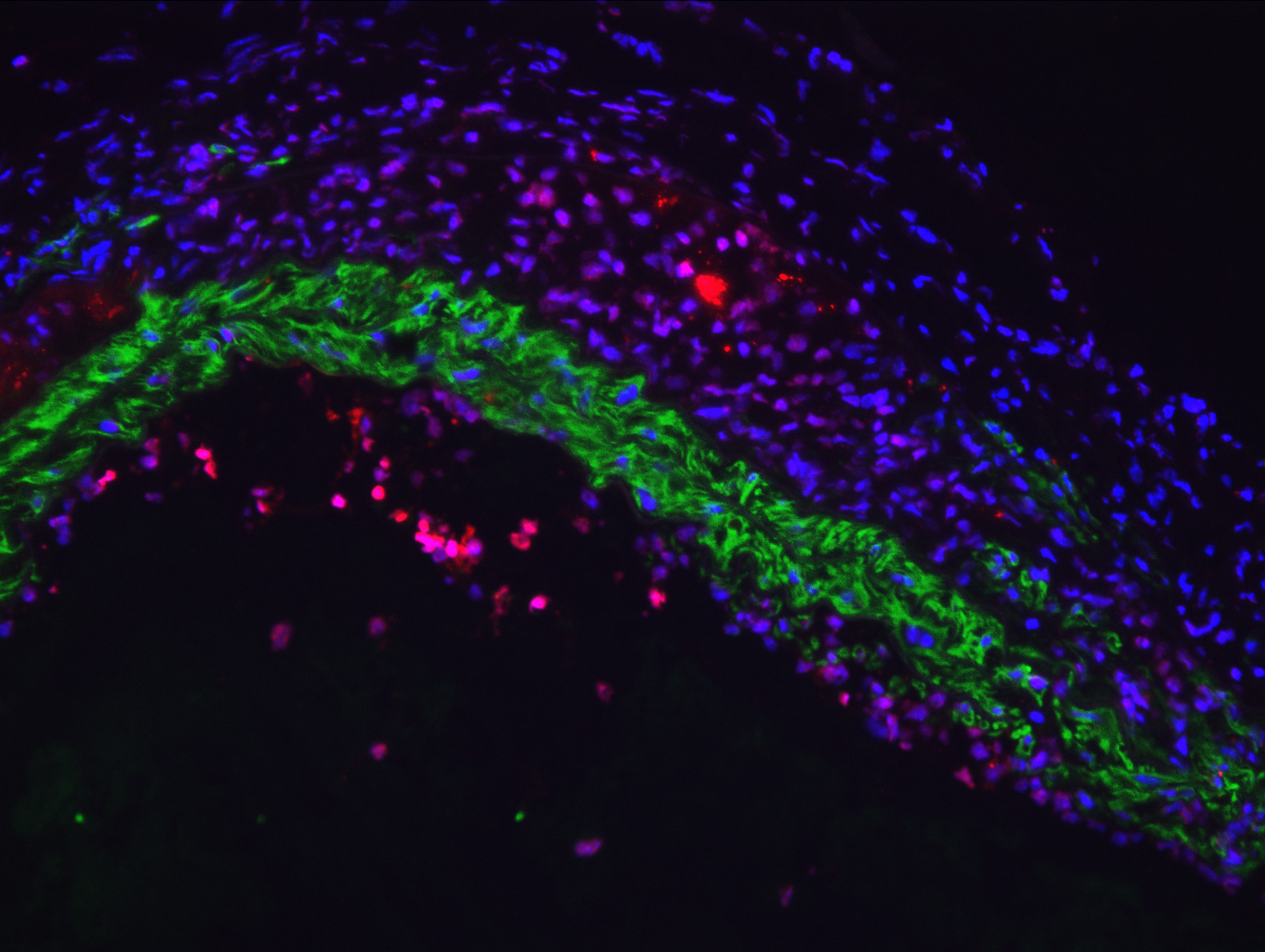

Supplement: Supplementary file 9 — Source data Fig. 8 [file 44321_2025_318_MOESM9_ESM.zip › Figure 8/Figure 8J/Saline Merge.tif]

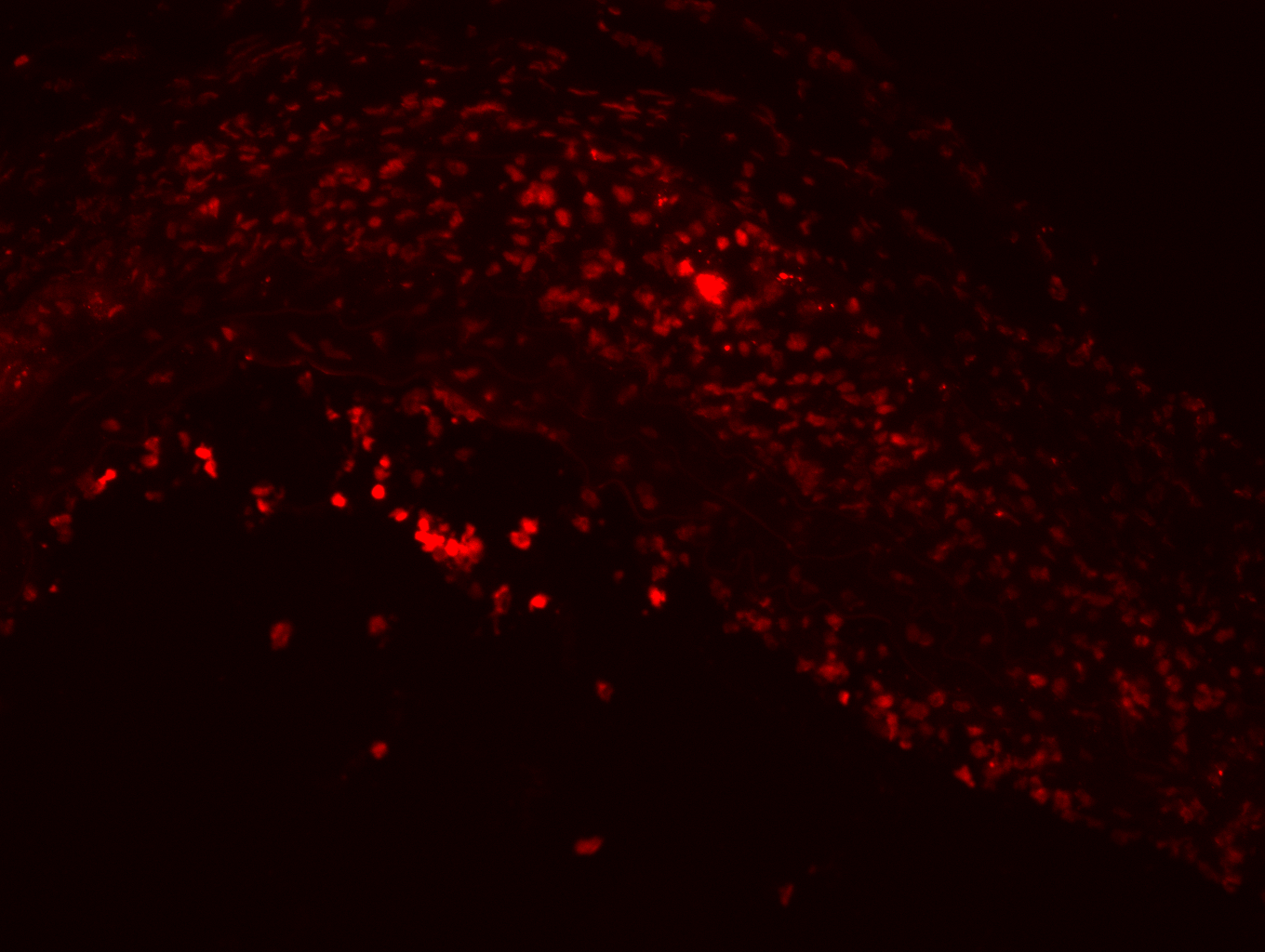

Supplement: Supplementary file 9 — Source data Fig. 8 [file 44321_2025_318_MOESM9_ESM.zip › Figure 8/Figure 8J/Saline Tunel.tif]
