# Supplementary material for: Brown remodeling of white adipose tissue protects against abdominal aortic aneurysm via batokine FSTL1
Source: EMBO Mol Med. 2025 Oct 9;17(11):3080–109. doi: 10.1038/s44321-025-00318-z (PMC12603302; doi:10.1038/s44321-025-00318-z)
Supplement: Supplementary file 10 — Expanded View Figures [file 44321_2025_318_MOESM10_ESM.pdf]

Expanded View Figures

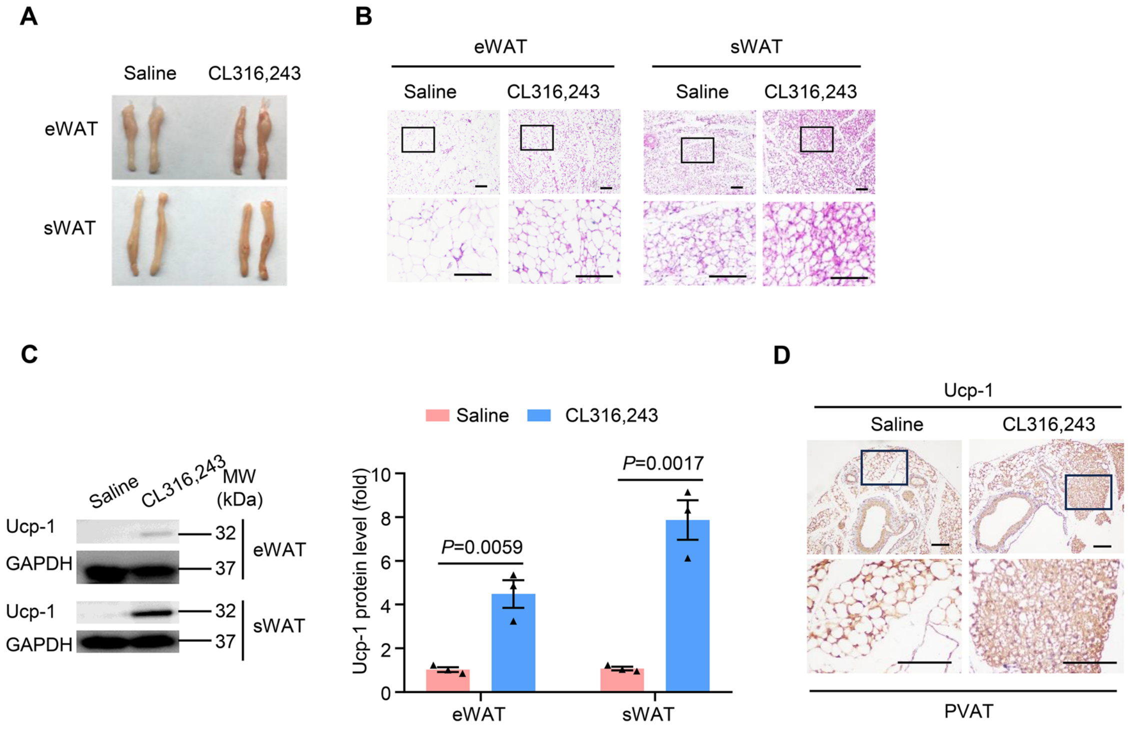

Figure EV1. CL316,243 administration successfully induced adipose tissue browning.

(**A**) Gross appearance of epididymal white adipose tissue (eWAT)and subcutaneous white adipose tissue (sWAT). (**B**) H&E staining. (**C**) Western blot analysis of Ucp-1 expression. (**D**) Immunohistochemical staining of Ucp-1 in perivascular adipose tissue ($n = 3$). Scale bar, 200 μm. Student's *t* test was used to determine statistical difference.

 © The Author(s)

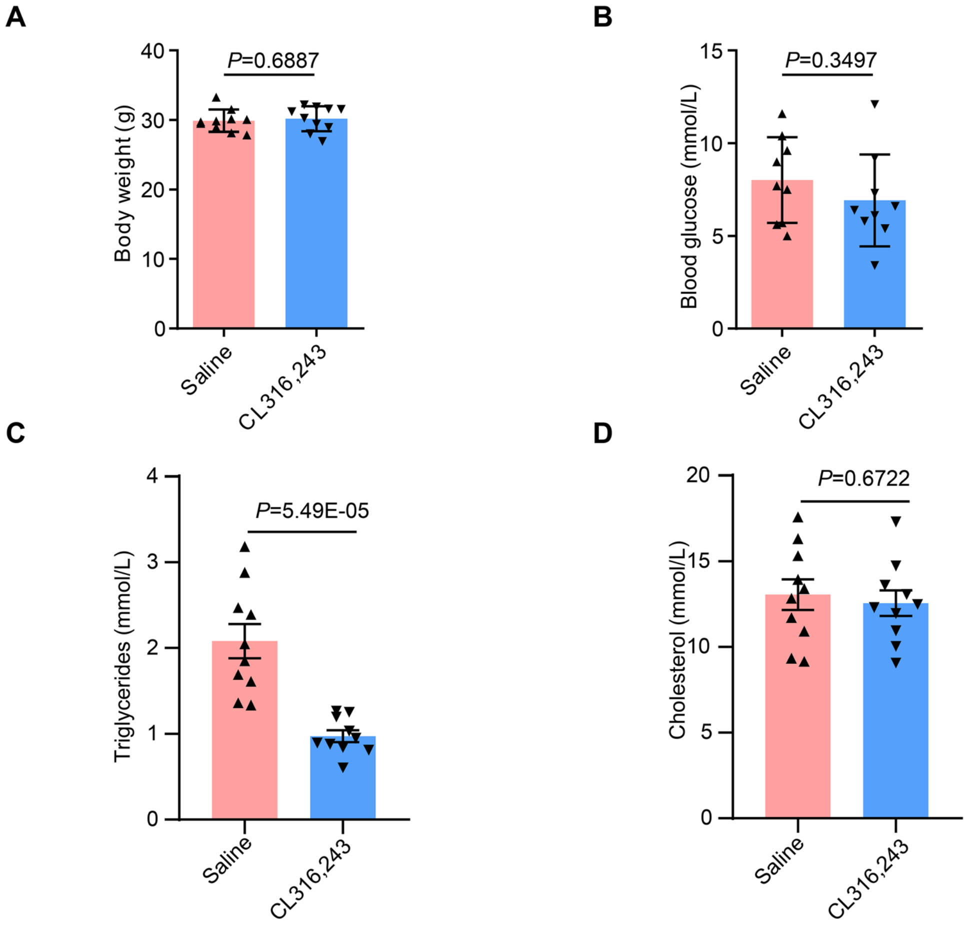

Figure EV2. Characteristics of ApoE$^{-/-}$ mice after CL316,243 treatment.

(**A**) Body weight ($n = 10$). (**B**) Blood glucose levels ($n = 9$). (**C**) Serum triglycerides ($n = 10$). (**D**) Serum cholesterol ($n = 10$). Student's *t* test was used to determine statistical difference.

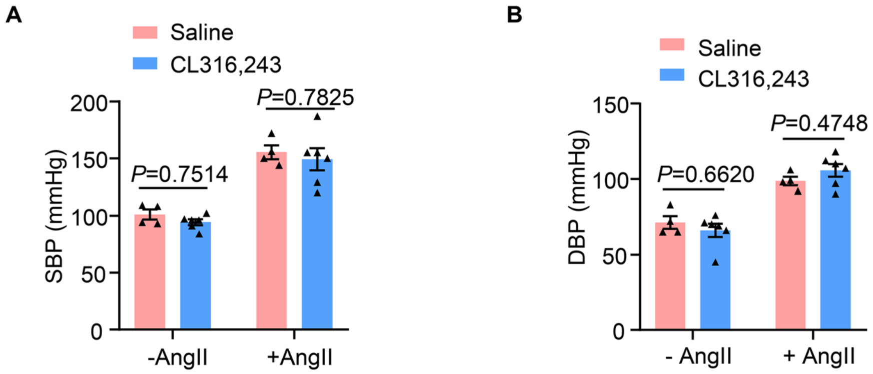

Figure EV3. CL316,243 administration did not significantly change systolic or diastolic blood pressures.

(**A**) Systolic blood pressure. (**B**) Diastolic blood pressure ($n = 4, 6$). Two-way ANOVA was used to determine statistical difference.

 © The Author(s)

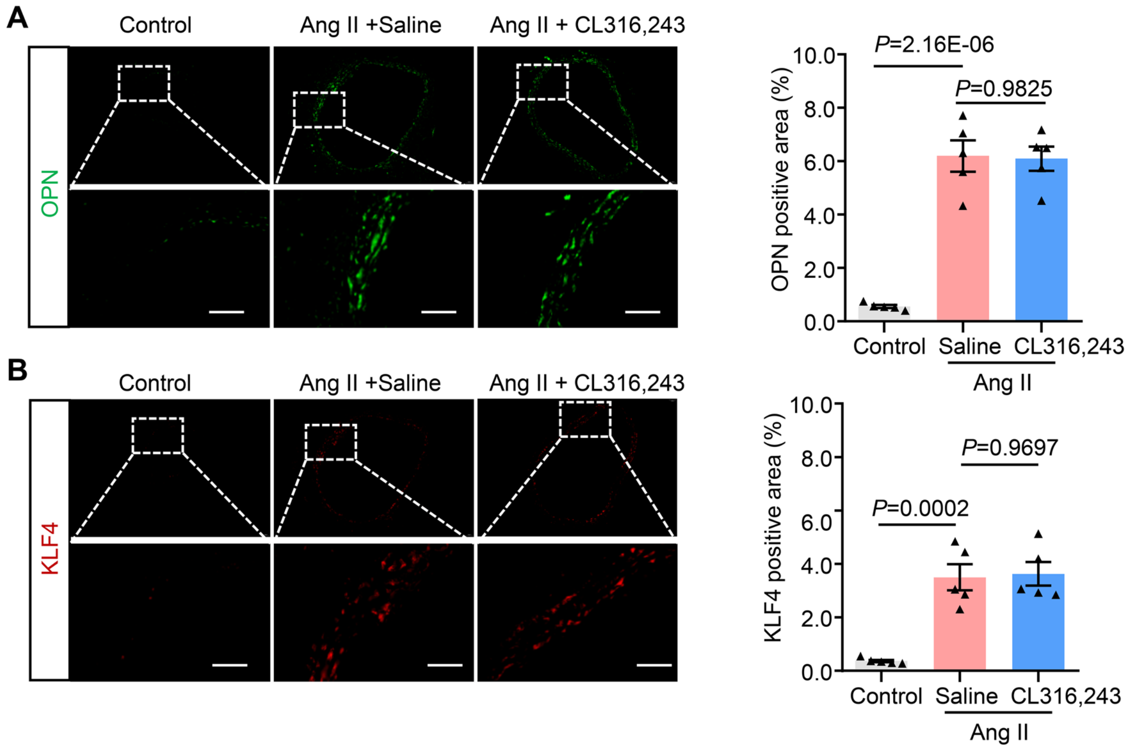

Figure EV4. CL316,243 administration did not alter the expression of VSMC phenotypic switch markers.

Immunofluorescence staining of Osteopontin (OPN) (**A**) and krüppel-like factor 4 (KLF4) (**B**) in aortic tissues from mice treated with saline or CL316,243 infused with Ang II ($n = 5$; scale bar, 25 μm). One-way ANOVA was used to determine statistical difference.

© The Author(s)

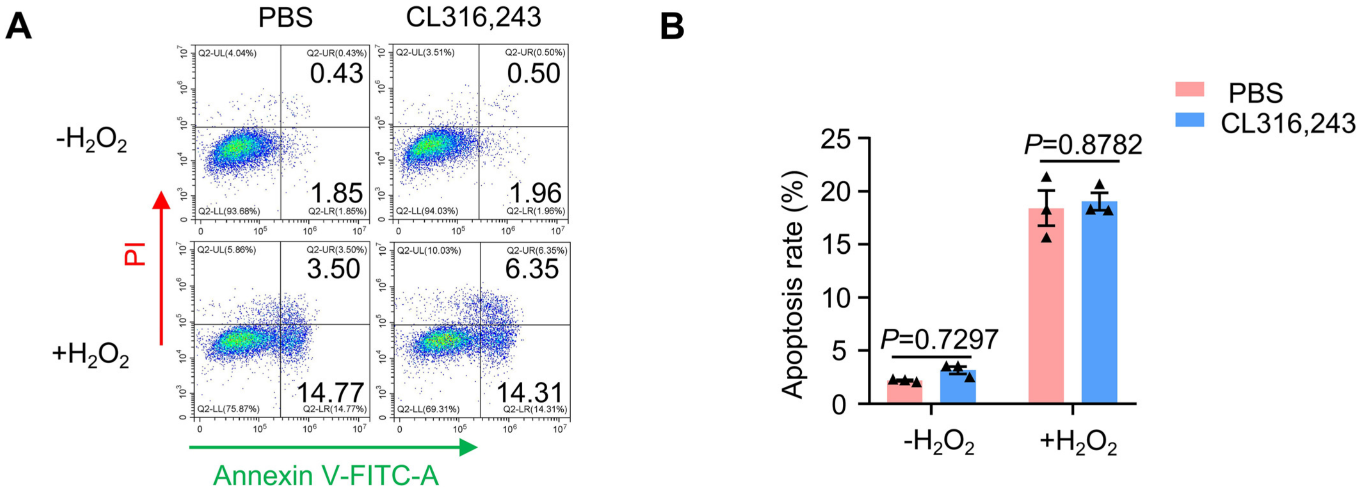

Figure EV5. CL316,243 did not have a direct effect on VSMC apoptosis.

(**A**) Flow cytometric analysis of Annexin V-FITC staining in VSMCs treated with PBS or CL316,243 and then stimulated with H_2O_2 for 24 h. (**B**) Quantification of apoptosis as in (**A**) ($n = 3$). Two-way ANOVA was used to determine statistical difference.

 © The Author(s)

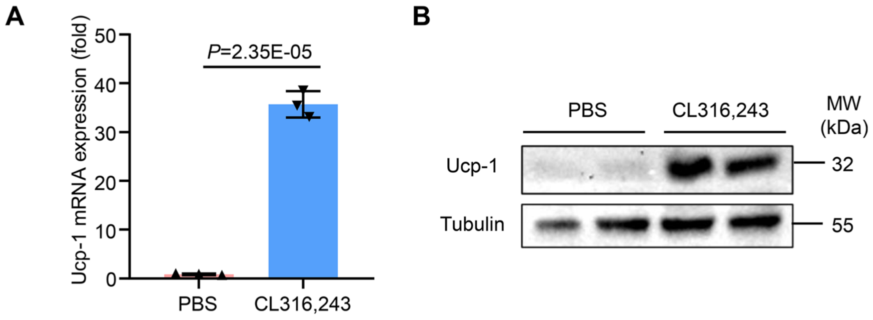

Figure EV6. CL316,243 treatment robustly induced adipocyte browning.

Primary adipocytes were treated with PBS or CL316,243 for 24 h. (**A**) Quantitative RT-PCR analysis of Ucp-1 mRNA level ($n = 3$). (**B**) Western blot analysis of Ucp-1 protein level. Student's *t* test was used to determine statistical difference.

© The Author(s)

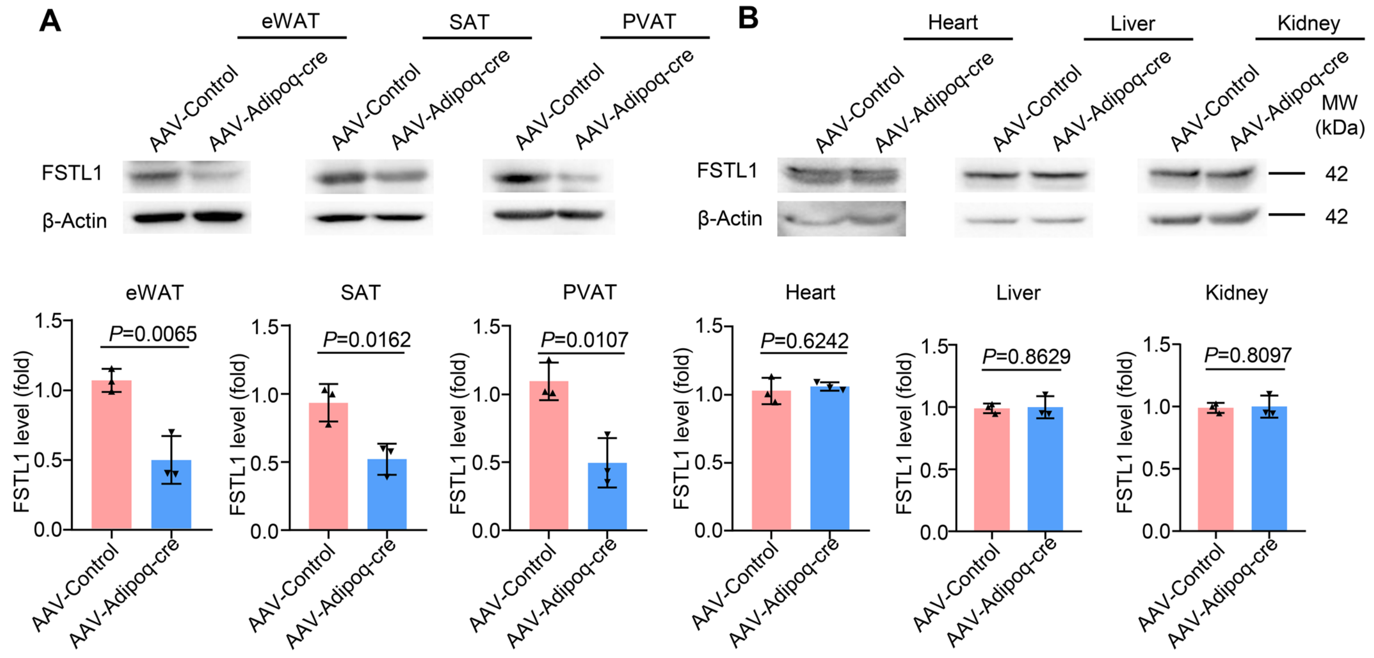

Figure EV7. Western blot analysis of FSTL1 expression after AAV injection and CL316,243 treatment.

(**A**) Expression of FSTL1 in adipose tissues. (**B**) Expression of FSTL1 in other tissues ($n = 3$). Student's t test was used to determine statistical difference.

© The Author(s)

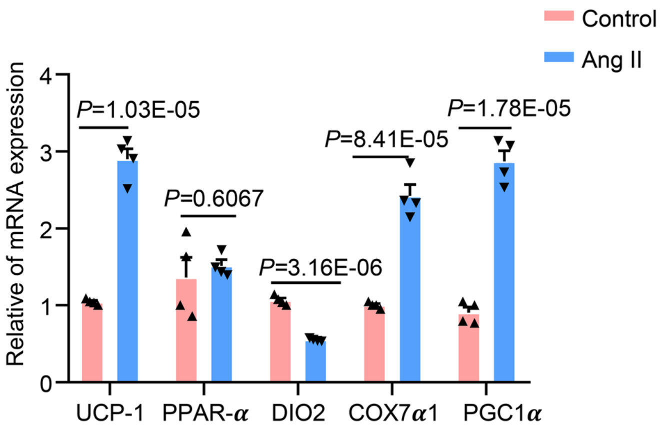

Figure EV8. Quantitative PCR analysis of mRNA expression of browning associated genes in PVAT of ApoE−/− mice treated with control or Ang II (*n* = 4).

Student's *t* test was used to determine statistical difference.

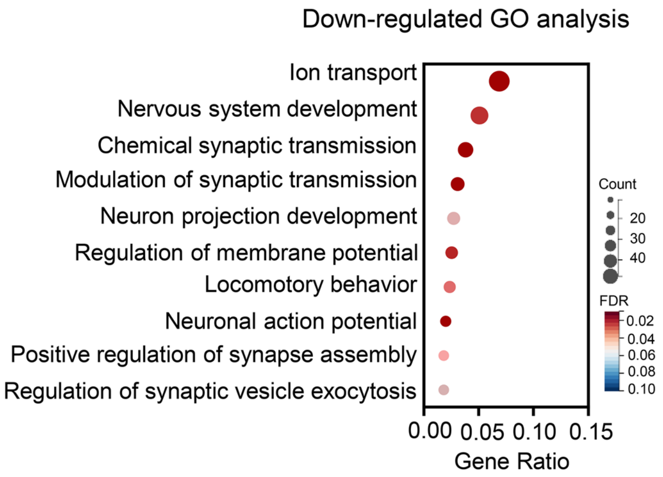

Figure EV9. Gene ontology (GO) analysis of downregulated proteins in aortic tissue from mice treated with saline or CL316,243 infused with Ang II.

 © The Author(s)
